# Draft genome of *Meyerozyma guilliermondii* strain vka1: a yeast strain with composting potential

**DOI:** 10.1186/s43141-020-00074-2

**Published:** 2020-09-29

**Authors:** Ravisankar Valsalan, Deepu Mathew

**Affiliations:** Bioinformatics Centre, Kerala Agricultural University, KAU Post, Thrissur, Kerala State 680 656 India

**Keywords:** Biodegradation, Genome annotation, Genome sequence, Illumina, NGS, Oxford Nanopore

## Abstract

**Background:**

*Meyerozyma guilliermondii* is a yeast which could be isolated from a variety of environments. The vka1 strain isolated and purified from the organic compost was found to have composting potential. To better understand the genes assisting the composting potential in this yeast, whole genome sequencing and sequence annotation were performed.

**Results:**

The genome of *M. guilliermondii* vka1 strain was sequenced using a hybrid approach, on Illumina Hiseq-2500 platform at 100× coverage followed by Nanopore platform at 20× coverage. The de novo assembly using dual-fold approach had given draft genome of 10.8 Mb size. The genome was found to contain 5385 genes. The annotation of the genes was performed, and the enzymes identified to have roles in the degradation of macromolecules are discussed in relation to its composting potential. Annotation of the genome assembly of the related strains had revealed the unique biodegradation related genes in this strain. Phylogenetic analysis using the rDNA region has confirmed the position of this strain in the Ascomycota family. Raw reads are made public, and the genome wide proteome profile is presented to facilitate further studies on this organism.

**Conclusions:**

*Meyerozyma guilliermondii* vka1 strain was sequenced through hybrid approach and the reads were de novo assembled. Draft genome size and the number of genes in the strain were assessed and discussed in relation to the related strains. Scientific insights into the composting potential of this strain are also presented in relation to the unique genes identified in this strain.

## Background

The yeast *Meyerozyma guilliermondii* (Wick.) Kurtzman and M. Suzuki, comb. nov. was first described as *Endomycopsis guilliermondii* by Wickerham [1]. The species was later placed in the genus *Pichia*, as *Pichia guilliermondii* [2] and recently renamed as *Meyerozyma guilliermondii* [3]. This ascomycetous yeast is widely distributed in the natural environment and forms part of the saprophytes on human skin and mucosal microflora*. Meyerozyma caribbica* (anamorph *Candida fermentati*) and *M. guilliermondii* (anamorph *Candida guilliermondii*) are two closely related species [4, 5]. This yeast has been an object of several studies, with a broad bibliography describing its multiple interesting properties and applications [6] and considered ubiquitous as they are found in deep-sea hydrothermal systems of the mid-Atlantic rift [7], wastewater treatment plants [8], maize wounds [9], and insect surfaces [10]. The yeast synthesizes large quantities of riboflavin [11] and has been extensively used in biotechnology industry.

*M. guilliermondii* is involved in xylitol production and polycyclic aromatic hydrocarbon degradation [12, 13]. It has been used industrially for the bioremediation purposes as well as for phosphate solubilizing [14, 15]. This yeast facilitates the process of tannin and saponin degradation also [16, 17].

In this study, we report the draft genome of the strain vka1 involved in the composting of organic waste. Hybrid sequencing followed by de novo assembly of the whole genome was performed. The objective of the study was to identify the number of genes in this strain and to find out and annotate the genes involved in the decomposing process, thus to give scientific back up for the biocomposting capability for this strain.

## Methods

### Yeast isolation and DNA extraction

The *M. guilliermondii* vka1 strain was isolated and purified from the organic compost at Kerala Agricultural University, India, following the standard protocol [18]. DNA was extracted according to the protocol by Dellaporta et al. [19]. For the extraction, pure culture of the yeast was isolated from the conical flask, frozen with liquid nitrogen in a mortar, ground to fine powder, and finally transferred to a tube containing extraction buffer (100 mM Tris pH 8.0, 50 mM EDTA pH 8.0, 500 mM NaCl, 10 mM mercaptoethanol and 1.25% SDS). After mixing, 5 M potassium acetate was added and incubated at 0 °C for 20 min. Supernatant obtained after centrifugation was poured into a clean tube where genomic DNA was allowed to precipitate with isopropanol for 30 min at – 20 °C. After centrifugation, pellet was resuspended in 50 mM Tris, 10 mM EDTA (pH 8.0) and transferred to a 20 μL tube where DNA was precipitated with 80% ethanol and the dried pellet was subsequently dissolved in 10 mM Tris, 1 mM EDTA (pH 8.0).

The 18S rDNA gene of strain vka1 was PCR amplified and Sanger sequenced. Based on the sequence, the species of the yeast was identified. To confirm the species identity, five 18S rDNA gene sequences each from five related species (*M. guilliermondii*, *M. caribbica*, *M. carpophila*, *Candida albicans*, and *C. amylolentus*) were retrieved from NCBI GenBank and subjected to the cluster analysis along with our sequence, with 1000 bootstrap replications.

### Sequencing and assembly

Genomic DNA was sequenced and de novo assembly of the reads was done. Sequencing was performed on Illumina Hiseq-2500 platform at 100× coverage followed by Nanopore sequencing at 20× coverage. Quality of the Illumina and Nanopore raw reads was assessed using FASTQC [20] and Poretools [21], respectively. The de novo assembly was performed using ABySS 2.2.4 [22]. Different k-mer lengths (40–190) were employed to optimize the assembly and the best k-mer value of 144 was chosen for the final output. The assembly was further conducted using SPAdes 3.11.1 [23] employing different k-mer lengths (21, 33, 55, 77, 99, and 111), setting the --cov cutoff parameter to auto, and using the --careful option.

### Gene prediction and annotation

Gene prediction from the scaffolds and annotation of these genes were performed using the following procedure. The assembly was input in to Augustus [24] using *Candida guilliermondii* as the trained dataset. Predicted genes were further analyzed using BLAST+ [25] and classified into different pathways using KAAS (KEGG Automatic Annotation Server) [26]. BBH (bi-directional best hit), the best method for annotating complete genomes in KEGG, was employed for the prediction, with *M. guilliermondii* selected as the organism.

### Analysis of genes in comparison with other strains

To understand the genes imparting the biodegradation capability to vka1 strain, its genome was compared with those of YLG18 and ATCC6260 strains. The assembled genome sequences, ASM975640v1 and ASM694215v1 of YLG18 and ATCC6260 strains, respectively, were downloaded in FASTA format from the SRA database of NCBI. The assembly was then annotated using the same pipeline detailed above.

### Phylogenetic analysis

The rDNA region, comprising the complete sequence of 18S rRNA, ITS1, 5.8S rRNA, ITS2, and 28S rRNA, from vka1 strain was used as query in BLASTn and 10 sequences belonging to various genera of Ascomycota were retrieved. Sequences were then aligned using MAFFT v.7.0 using L-INS-i strategy [27] and the aligned sequences analyzed using MEGA X [28], employing NJ method with 1000 bootstrap replications. The evolutionary distances were computed using the Jukes-Cantor method.

## Results

The yeast has been isolated and purified from the composted material and DNA was extracted using Dellaporta method. Purity of the DNA was good enough for sequencing. Analysis of the 18S rDNA sequence has shown that the organism is *M. guilliermondii*. Cluster analysis with the sequences of the related yeasts had further confirmed the identity of the strain.

The Illumina results had 10,514,805 reads and the Nanopore had 261,366 reads. Raw reads were of high quality and the genome features of the strain are summarized in Table 1. Reads are made available in the NCBI SRA database under the BioProject No. PRJNA598411. Although the genome was sequenced with a combination of long and short reads for obtaining larger number of scaffolds, only a paired-end short read library was employed in de novo assembly. The dual-fold assembly approach has used ABySS 2.2.4 followed by confirmation with SPAdes 3.11.1 assemblers. Different k-mer values were employed for obtaining the assembly and the best value was 144. The final assembly consisted of 36 scaffolds as predicted by the assemblers. After the final assembly, draft genome with 10.8 Mb size was obtained.

**Table 1:** Genome features of *M. guilliermondii* strain vka1

Genome	Features	Value
*M. guilliermondii* strain vka1	Reads	1,05,14,805 (Illumina) 2,61,366 (Nanopore)
	Scaffolds	36
	Max scaffold length	14,99,707
	Min scaffold length	1270
	Genome size	10.8 Mb
	N50	5,67,598
	No. of genes	5385

**Fig. 1:**
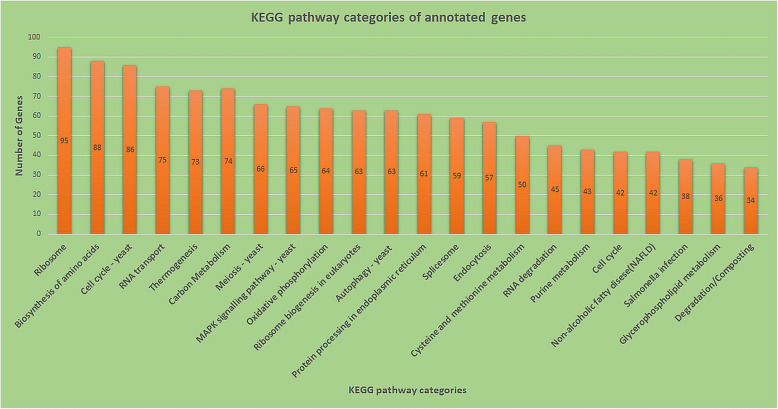
Major KEGG pathway categories of annotated genes from the *M. guilliermondii* strain vka1

Augustus, a gene prediction program that uses a generalized hidden Markov model, was used for prediction of the genes. It has predicted 5385 genes from the vka1 strain (Table 1). KAAS annotation has classified the genes into 380 groups involved in diverse pathways (Fig. 1), of which 20 categories represented the enzymes involved in the degradation of macromolecules. The enzymes with roles in the biodegradation processes along with the list of other proteins identified in the genome are presented in Supplementary Table 1.

Comparison of the genes identified from the vka1, YLG18, and ATCC6260 strains had revealed the unique biodegradation-related genes in vka1. The enzymes S-(hydroxymethyl)glutathione dehydrogenase, phenylacetate 2-hydroxylase, trimethyllysine dioxygenase, 4-aminobutyrate aminotransferase, and delta3-delta2-enoyl-CoA isomerase were found only in vka1 strain.

**Fig. 2:**
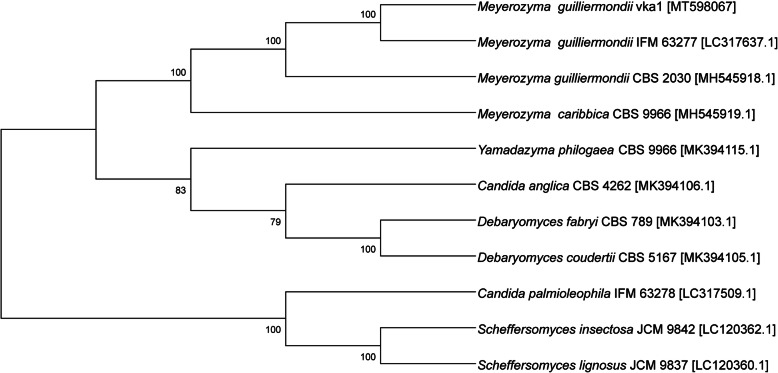
Phylogenetic relationships (cladogram) based on the sequences of rDNA region from selected genomes, inferred using the Neighbour Joining (NJ) method. The percentages of replicate trees in which the associated taxa clustered together in the bootstrap test (1000 replicates) are shown next to the branches

Phylogenetic analysis was carried out using the rDNA sequences of different genera belonging to Ascomycetes phylum. The phylogenetic tree had high bootstrap values for all the branches. Of the two major clades (Fig. 2), the first one had eight accessions divided into two sub-clusters. The first sub-cluster included *Meyerozyma* genus with 100% bootstrap support. Strain vka1 clustered along with *M. guilliermondii* strain IFM 63277. The second sub-cluster had two *Debaryomyces* accessions along with *Candida anglica* and *Yamadazyma philogaea*. Second major clade had three accessions, two belonging to *Scheffersomyces*, and one *Candida palmioleophila* accession forming an outgroup.

## Discussion

The ascomycetous yeast *M. guilliermondii* is widely distributed in the environment including human body. This microbe is also exploited commercially in riboflavin and xylitol production [11]. However, it is well understood to have roles in degradation of macromolecules including polycyclic aromatic hydrocarbons [13], phosphates [15], tannins and saponins [16]. Due to these properties, it is well utilized in bioremediation [14].

Though Illumina sequencing platform offers better read accuracy, short read lengths is a concern [29]. On the other hand, Nanopore platform generates longer reads though comparatively error prone [30]. Method based on the combined analysis of short and long reads generated from different platforms is found to result in comprehensive genome characterization [31].

Several new *de novo* assembly tools have been developed recently to assemble short sequencing reads generated by next-generation sequencing platforms. Different assembly tools have their advantages and disadvantages and hence a dual-fold approach is reported to give better assemblies [32]. Thus, ABySS and SPAdes based de novo assembly was chosen in this study. The genome size shown by the assembly was slightly higher than 10.6 Mb in *M. guilliermondii* strain ATCC6260 and 10.64 Mb in *P. perniciosus*, both sequenced on Illumina HiSeq-2500 platform [33]. The gene calling algorithm Augustus has predicted 5385 genes from the vka1 strain. The number of genes obtained is more than that in a previous study which reported 5275 genes [34], but slightly lower than 5401 genes reported by De Marco et al. [33].

Bi-directional best hit method followed in this study is identified as the best method for annotating the complete genomes [35]. The annotation had shown that the genome accommodates many enzymes which are directly involved in the biodegradation processes, explaining the composting potential of this strain. The enzyme salicylate hydroxylase is identified to be involved in decarboxylative hydroxylation [36]. Similarly, urea carboxylase is involved in carboxylation in the degradation process [37]. Enzymes cyanamide hydratase [38], phenylacetate 2-hydroxylase [39, 40], aldehyde dehydrogenase [41], saccharopine dehydrogenase [42], sarcosine oxidase [43], dihydrolipoamide dehydrogenase [44], trimethyllysine dioxygenase [45], 2-oxoglutarate dehydrogenase [44], malonate-semialdehyde dehydrogenase [46], acyl-CoA dehydrogenase [47], hydroxymethylglutaryl-CoA synthase [48], 3-hydroxyisobutyryl-CoA hydrolase [49], 4-aminobutyrate aminotransferase [50], S-(hydroxymethyl)glutathione dehydrogenase [51], acyl-CoA oxidase [52], delta3-delta2-enoyl-CoA isomerase [53], beta-galactosidase [54], alpha-mannosidase [55], and hexosaminidase [56] are all well understood to have direct involvement in the macromolecule catabolism. This array of macromolecule degrading enzymes, including the unique genes identified, explains the composting potential of this strain. Future studies are required on these enzymes and on this yeast to understand the mechanisms.

The phylogenetic analysis was in agreement with the previous taxonomic studies on *Meyerozyma* and ascomycetes. The analysis had confirmed that vka1 strain belongs to *M. guilliermondii* and bootstrap value of 100% affirms this result. The genera *Debaryomyces*, *Yamadazyma*, and *Candida* which clustered into a single clade, with the bootstrap value of 83.0%, have previously been shown to be close relatives of *Meyerozyma* [3, 57, 58].

## Conclusion

*M. guilliermondii* is an ascomycetous yeast universally distributed in the natural environment and having a wide range of industrial applications. The draft genome of a new strain, vka1, with potential for composting the organic wastes is being reported. Hybrid sequencing followed by dual-fold assembly was used to assess the genome size and the annotation has shown the total number of genes coded. Twenty categories of enzymes which are involved in the macromolecule degradation were identified, and this wide spectrum along with the unique enzymes identified explains the composing potential of this strain. The phylogenetic analysis has confirmed its position in the Ascomycota family.

## Supplementary information

**Additional file 1. **Supplementary Table 1. Detailed results of KEGG (KAAS) analysis of *Meyerozyma guilliermondii* strain vka1 genome, showing the genes for the enzymes annotated and their respective categories (Categories given in boldface).

## Data Availability

The authors declare that sequence reads associated with this article are available in the NCBI SRA database under the BioProject No. PRJNA598411.

## Abbreviations

EDTA: Ethylenediaminetetraacetic acid
ITS: Internal transcribed spacer
KEGG: Kyoto Encyclopedia of Genes and Genomes
MEGA: Molecular Evolutionary Genetics Analysis
NCBI: National Center for Biotechnology Information
SDS: Sodium dodecyl sulfate
