# Supplementary material for: Draft genome of Meyerozyma guilliermondii strain vka1: a yeast strain with composting potential
Source: J Genet Eng Biotechnol. 2020 Sep 29;18:54. doi: 10.1186/s43141-020-00074-2 (PMC7524887; doi:10.1186/s43141-020-00074-2)
Supplement: Supplementary file 1 — Additional file 1. Supplementary Table 1. Detailed results of KEGG (KAAS) analysis of Meyerozyma guilliermondii strain vka1 genome, showing the genes for the enzymes annotated and their respective categories (Categories given in boldface). [file 43141_2020_74_MOESM1_ESM.pdf]

Supplementary Table 1. Detailed results of KEGG (KAAS) analysis of *Meyerozyma guilliermondii* strain vka1 genome, showing the genes for the enzymes annotated and their respective categories (Categories given in boldface)

1. Metabolic pathways

AKR1A1; alcohol dehydrogenase (NADP+) [EC:1.1.1.2]

hom; homoserine dehydrogenase [EC:1.1.1.3]

SORD; L-iditol 2-dehydrogenase [EC:1.1.1.14]

gyaR; glyoxylate reductase [EC:1.1.1.26]

HMGCR; hydroxymethylglutaryl-CoA reductase (NADPH) [EC:1.1.1.34]

MDH2; malate dehydrogenase [EC:1.1.1.37]

IDH3; isocitrate dehydrogenase (NAD+) [EC:1.1.1.41]

IDH1; isocitrate dehydrogenase [EC:1.1.1.42]

PGD; 6-phosphogluconate dehydrogenase [EC:1.1.1.44 1.1.1.343]

G6PD; glucose-6-phosphate 1-dehydrogenase [EC:1.1.1.49 1.1.1.363]

leuB; 3-isopropylmalate dehydrogenase [EC:1.1.1.85]

ilvC; ketol-acid reductoisomerase [EC:1.1.1.86]

serA; D-3-phosphoglycerate dehydrogenase / 2-oxoglutarate reductase [EC:1.1.1.95 1.1.1.399]

fabG; 3-oxoacyl-[acyl-carrier protein] reductase [EC:1.1.1.100]

panE; 2-dehydropantoate 2-reductase [EC:1.1.1.169]

IMPDH; IMP dehydrogenase [EC:1.1.1.205]

lldD; L-lactate dehydrogenase (cytochrome) [EC:1.1.2.3]

LDHD; D-lactate dehydrogenase (cytochrome) [EC:1.1.2.4]

L2HGDH; 2-hydroxyglutarate dehydrogenase [EC:1.1.99.2]

frmA; S-(hydroxymethyl)glutathione dehydrogenase / alcohol dehydrogenase [EC:1.1.1.284 1.1.1.1]

FDH; formate dehydrogenase [EC:1.17.1.9]

ALDH; aldehyde dehydrogenase (NAD+) [EC:1.2.1.3]

E1.2.1.5; aldehyde dehydrogenase (NAD(P)+) [EC:1.2.1.5]

asd; aspartate-semialdehyde dehydrogenase [EC:1.2.1.11]

GAPDH; glyceraldehyde 3-phosphate dehydrogenase [EC:1.2.1.12]

gabD; succinate-semialdehyde dehydrogenase / glutarate-semialdehyde dehydrogenase [EC:1.2.1.16 1.2.1.79 1.2.1.20]

mmsA; malonate-semialdehyde dehydrogenase (acetylating) / methylmalonate-semialdehyde dehydrogenase [EC:1.2.1.18 1.2.1.27]

LYS2; L-2-aminoadipate reductase [EC:1.2.1.95]

proA; glutamate-5-semialdehyde dehydrogenase [EC:1.2.1.41]

PDHA; pyruvate dehydrogenase E1 component alpha subunit [EC:1.2.4.1]

PDHB; pyruvate dehydrogenase E1 component beta subunit [EC:1.2.4.1]

OGDH; 2-oxoglutarate dehydrogenase E1 component [EC:1.2.4.2]

TYR1; prephenate dehydrogenase (NADP+) [EC:1.3.1.13]

TM7SF2; Delta14-sterol reductase [EC:1.3.1.70]

ERG4; Delta24(24(1))-sterol reductase [EC:1.3.1.71]

PPOX; protoporphyrinogen/coproporphyrinogen III oxidase [EC:1.3.3.4 1.3.3.15]

E1.3.3.6; acyl-CoA oxidase [EC:1.3.3.6]

SDHA; succinate dehydrogenase (ubiquinone) flavoprotein subunit [EC:1.3.5.1]

SDHB; succinate dehydrogenase (ubiquinone) iron-sulfur subunit [EC:1.3.5.1]

SDHD; succinate dehydrogenase (ubiquinone) membrane anchor subunit

ACADM; acyl-CoA dehydrogenase [EC:1.3.8.7]

DHODH; dihydroorotate dehydrogenase [EC:1.3.5.2]

E1.4.1.4; glutamate dehydrogenase (NADP+) [EC:1.4.1.4]

DAO; D-amino-acid oxidase [EC:1.4.3.3]

pdxH; pyridoxamine 5'-phosphate oxidase [EC:1.4.3.5]

AOC3; primary-amine oxidase [EC:1.4.3.21]

GLDC; glycine dehydrogenase [EC:1.4.4.2]

proC; pyrroline-5-carboxylate reductase [EC:1.5.1.2]

MTHFD; methylenetetrahydrofolate dehydrogenase (NADP+) / methenyltetrahydrofolate cyclohydrolase / formyltetrahydrofolate synthetase [EC:1.5.1.5 3.5.4.9 6.3.4.3]

LYS1; saccharopine dehydrogenase (NAD+, L-lysine forming) [EC:1.5.1.7]

LYS9; saccharopine dehydrogenase (NADP+, L-glutamate forming) [EC:1.5.1.10]

E1.2.1.88; 1-pyrroline-5-carboxylate dehydrogenase [EC:1.2.1.88]

MTD1; methylenetetrahydrofolate dehydrogenase (NAD+) [EC:1.5.1.15]

metF; methylenetetrahydrofolate reductase (NADPH) [EC:1.5.1.20]

PIPOX; sarcosine oxidase / L-pipecolate oxidase [EC:1.5.3.1 1.5.3.7]

PRODH; proline dehydrogenase [EC:1.5.5.2]

uaZ; urate oxidase [EC:1.7.3.3]

cysJ; sulfite reductase (NADPH) flavoprotein alpha-component [EC:1.8.1.2]

cysI; sulfite reductase (NADPH) hemoprotein beta-component [EC:1.8.1.2]

DLD; dihydrolipoamide dehydrogenase [EC:1.8.1.4]

GSR; glutathione reductase (NADPH) [EC:1.8.1.7]

cysH; phosphoadenosine phosphosulfate reductase [EC:1.8.4.8 1.8.4.10]

UQCRFS1; ubiquinol-cytochrome c reductase iron-sulfur subunit [EC:7.1.1.8]

CYC1; ubiquinol-cytochrome c reductase cytochrome c1 subunit

QCR1; ubiquinol-cytochrome c reductase core subunit 1

QCR2; ubiquinol-cytochrome c reductase core subunit 2

QCR7; ubiquinol-cytochrome c reductase subunit 7

QCR8; ubiquinol-cytochrome c reductase subunit 8

HAAO; 3-hydroxyanthranilate 3,4-dioxygenase [EC:1.13.11.6]

CDO1; cysteine dioxygenase [EC:1.13.11.20]

ncd2; nitronate monooxygenase [EC:1.13.12.16]

IDO; indoleamine 2,3-dioxygenase [EC:1.13.11.52]

TMLHE; trimethyllysine dioxygenase [EC:1.14.11.8]

E1.14.13.1; salicylate hydroxylase [EC:1.14.13.1]

KMO; kynurenine 3-monooxygenase [EC:1.14.13.9]

SCD; stearoyl-CoA desaturase (Delta-9 desaturase) [EC:1.14.19.1]

SQLE; squalene monooxygenase [EC:1.14.14.17]

COMT; catechol O-methyltransferase [EC:2.1.1.6]

mmuM; homocysteine S-methyltransferase [EC:2.1.1.10]

metE; 5-methyltetrahydropteroyltriglutamate--homocysteine methyltransferase [EC:2.1.1.14]

PEMT; phosphatidylethanolamine/phosphatidyl-N-methylethanolamine N-methyltransferase [EC:2.1.1.17 2.1.1.71]

SMT1; sterol 24-C-methyltransferase [EC:2.1.1.41]

thyA; thymidylate synthase [EC:2.1.1.45]

MET1; uroporphyrin-III C-methyltransferase [EC:2.1.1.107]

COQ3; polyprenyldihydroxybenzoate methyltransferase/ 3-demethylubiquinol 3-O-methyltransferase [EC:2.1.1.114 2.1.1.64]

glyA; glycine hydroxymethyltransferase [EC:2.1.2.1]

E2.1.2.2; phosphoribosylglycinamide formyltransferase [EC:2.1.2.2]

purH; phosphoribosylaminoimidazolecarboxamide formyltransferase/ IMP cyclohydrolase [EC:2.1.2.3 3.5.4.10]

gcvT; aminomethyltransferase [EC:2.1.2.10]

panB; 3-methyl-2-oxobutanoate hydroxymethyltransferase [EC:2.1.2.11]

OTC; ornithine carbamoyltransferase [EC:2.1.3.3]

E2.2.1.1; transketolase [EC:2.2.1.1]

E2.2.1.2; transaldolase [EC:2.2.1.2]

ARG2; amino-acid N-acetyltransferase [EC:2.3.1.1]

argJ; glutamate N-acetyltransferase/ amino-acid N-acetyltransferase [EC:2.3.1.35 2.3.1.1]

GNPNAT1; glucosamine-phosphate N-acetyltransferase [EC:2.3.1.4]

ACAT; acetyl-CoA C-acetyltransferase [EC:2.3.1.9]

DLAT; pyruvate dehydrogenase E2 component (dihydrolipoamide acetyltransferase) [EC:2.3.1.12]

metX; homoserine O-acetyltransferase/O-succinyltransferase [EC:2.3.1.31 2.3.1.46]

E2.3.1.37; 5-aminolevulinate synthase [EC:2.3.1.37]

fabD; [acyl-carrier-protein] S-malonyltransferase [EC:2.3.1.39]

SPT; serine palmitoyltransferase [EC:2.3.1.50]

DLST; 2-oxoglutarate dehydrogenase E2 component (dihydrolipoamide succinyltransferase) [EC:2.3.1.61]

FAS2; fatty acid synthase subunit alpha, fungi type [EC:2.3.1.86]

FAS1; fatty acid synthase subunit beta, fungi type [EC:2.3.1.86]

E2.3.1.158; phospholipid:diacylglycerol acyltransferase [EC:2.3.1.158]

ggt; gamma-glutamyltranspeptidase / glutathione hydrolase [EC:2.3.2.2 3.4.19.13]

PYG; glycogen phosphorylase [EC:2.4.1.1]

GYS; glycogen synthase [EC:2.4.1.11]

otsA; trehalose 6-phosphate synthase [EC:2.4.1.15 2.4.1.347]

CHS1; chitin synthase [EC:2.4.1.16]

GBE1; 1,4-alpha-glucan branching enzyme [EC:2.4.1.18]

E2.4.1.34; 1,3-beta-glucan synthase [EC:2.4.1.34]

UGCG; ceramide glucosyltransferase [EC:2.4.1.80]

POMT; dolichyl-phosphate-mannose-protein mannosyltransferase [EC:2.4.1.109]

ALG5; dolichyl-phosphate beta-glucosyltransferase [EC:2.4.1.117]

GYG1; glycogenin [EC:2.4.1.186]

APRT; adenine phosphoribosyltransferase [EC:2.4.2.7]

upp; uracil phosphoribosyltransferase [EC:2.4.2.9]

pyrE; orotate phosphoribosyltransferase [EC:2.4.2.10]

pncB; nicotinate phosphoribosyltransferase [EC:6.3.4.21]

purF; amidophosphoribosyltransferase [EC:2.4.2.14]

trpD; anthranilate phosphoribosyltransferase [EC:2.4.2.18]

nadC; nicotinate-nucleotide pyrophosphorylase (carboxylating) [EC:2.4.2.19]

FDPS; farnesyl diphosphate synthase [EC:2.5.1.1 2.5.1.10]

metK; S-adenosylmethionine synthetase [EC:2.5.1.6]

miaA; tRNA dimethylallyltransferase [EC:2.5.1.75]

ribE; riboflavin synthase [EC:2.5.1.9]

speE; spermidine synthase [EC:2.5.1.16]

GST; glutathione S-transferase [EC:2.5.1.18]

FDFT1; farnesyl-diphosphate farnesyltransferase [EC:2.5.1.21]

SMS; spermine synthase [EC:2.5.1.22]

GGPS1; geranylgeranyl diphosphate synthase, type III [EC:2.5.1.1 2.5.1.10 2.5.1.29]

GPT; alanine transaminase [EC:2.6.1.2]

hisC; histidinol-phosphate aminotransferase [EC:2.6.1.9]

E2.6.1.11; acetylornithine aminotransferase [EC:2.6.1.11]

rocD; ornithine--oxo-acid transaminase [EC:2.6.1.13]

glmS; glutamine---fructose-6-phosphate transaminase (isomerizing) [EC:2.6.1.16]

E2.6.1.42; branched-chain amino acid aminotransferase [EC:2.6.1.42]

AGXT; alanine-glyoxylate transaminase / serine-glyoxylate transaminase / serine-pyruvate transaminase [EC:2.6.1.44 2.6.1.45 2.6.1.51]

serC; phosphoserine aminotransferase [EC:2.6.1.52]

ARO8; aromatic amino acid aminotransferase I/ 2-aminoadipate transaminase [EC:2.6.1.57 2.6.1.39 2.6.1.27 2.6.1.5]

HK; hexokinase [EC:2.7.1.1]

galK; galactokinase [EC:2.7.1.6]

pfkA; 6-phosphofructokinase 1 [EC:2.7.1.11]

E2.7.1.12; gluconokinase [EC:2.7.1.12]

rbsK; ribokinase [EC:2.7.1.15]

xylB; xylulokinase [EC:2.7.1.17]

E2.7.1.20; adenosine kinase [EC:2.7.1.20]

ppnK; NAD+ kinase [EC:2.7.1.23]

coaE; dephospho-CoA kinase [EC:2.7.1.24]

cysC; adenylylsulfate kinase [EC:2.7.1.25]

RFK; riboflavin kinase [EC:2.7.1.26]

DAK; triose/dihydroxyacetone kinase / FAD-AMP lyase (cyclizing) [EC:2.7.1.28 2.7.1.29 4.6.1.15]

CKI1; choline kinase [EC:2.7.1.32]

pdxK; pyridoxine kinase [EC:2.7.1.35]

E2.7.1.36; mevalonate kinase [EC:2.7.1.36]

thrB; homoserine kinase [EC:2.7.1.39]

PK; pyruvate kinase [EC:2.7.1.40]

udk; uridine kinase [EC:2.7.1.48]

THI20; hydroxymethylpyrimidine/phosphomethylpyrimidine kinase / thiaminase [EC:2.7.1.49 2.7.4.7 3.5.99.2]

PI4KA; phosphatidylinositol 4-kinase A [EC:2.7.1.67]

PIP5K; 1-phosphatidylinositol-4-phosphate 5-kinase [EC:2.7.1.68]

ETNK; ethanolamine kinase [EC:2.7.1.82]

E2.7.1.105; 6-phosphofructo-2-kinase [EC:2.7.1.105]

DOLK; dolichol kinase [EC:2.7.1.108]

PIK3C3; phosphatidylinositol 3-kinase [EC:2.7.1.137]

IPMK; inositol-polyphosphate multikinase [EC:2.7.1.140 2.7.1.151]

PIKFYVE; 1-phosphatidylinositol-3-phosphate 5-kinase [EC:2.7.1.150]

PGK; phosphoglycerate kinase [EC:2.7.2.3]

lysC; aspartate kinase [EC:2.7.2.4]

proB; glutamate 5-kinase [EC:2.7.2.11]

E2.7.4.2; phosphomevalonate kinase [EC:2.7.4.2]

adk; adenylate kinase [EC:2.7.4.3]

ndk; nucleoside-diphosphate kinase [EC:2.7.4.6]

E2.7.4.8; guanylate kinase [EC:2.7.4.8]

tmk; dTMP kinase [EC:2.7.4.9]

PRPS; ribose-phosphate pyrophosphokinase [EC:2.7.6.1]

thiN; thiamine pyrophosphokinase [EC:2.7.6.2]

FLAD1; FAD synthetase [EC:2.7.7.2]

sat; sulfate adenylyltransferase [EC:2.7.7.4]

UGP2; UTP--glucose-1-phosphate uridylyltransferase [EC:2.7.7.9]

galT; UDPglucose--hexose-1-phosphate uridylyltransferase [EC:2.7.7.12]

GMPP; mannose-1-phosphate guanylyltransferase [EC:2.7.7.13]

PCYT2; ethanolamine-phosphate cytidylyltransferase [EC:2.7.7.14]

PCYT1; choline-phosphate cytidylyltransferase [EC:2.7.7.15]

APA1_2; sulfate adenylyltransferase (ADP) / ATP adenylyltransferase [EC:2.7.7.5 2.7.7.53]

EPT1; ethanolaminephosphotransferase [EC:2.7.8.1]

pgsA; CDP-diacylglycerol---glycerol-3-phosphate 3-phosphatidyltransferase [EC:2.7.8.5]

CDIPT; CDP-diacylglycerol--inositol 3-phosphatidyltransferase [EC:2.7.8.11]

ALG7; UDP-N-acetylglucosamine--dolichyl-phosphate N-acetylglucosaminephosphotransferase [EC:2.7.8.15]

bioB; biotin synthase [EC:2.8.1.6]

lip; triacylglycerol lipase [EC:3.1.1.3]

MGLL; acylglycerol lipase [EC:3.1.1.23]

PGLS; 6-phosphogluconolactonase [EC:3.1.1.31]

E3.1.1.45; carboxymethylenebutenolidase [EC:3.1.1.45]

E3.1.2.1; acetyl-CoA hydrolase [EC:3.1.2.1]

gloB; hydroxyacylglutathione hydrolase [EC:3.1.2.6]

frmB; S-formylglutathione hydrolase [EC:3.1.2.12]

PPT; palmitoyl-protein thioesterase [EC:3.1.2.22]

E3.1.3.1; alkaline phosphatase [EC:3.1.3.1]

PHO; acid phosphatase [EC:3.1.3.2]

serB; phosphoserine phosphatase [EC:3.1.3.3]

cysQ; 3'(2'), 5'-bisphosphate nucleotidase [EC:3.1.3.7]

GEP4; phosphatidylglycerophosphatase GEP4 [EC:3.1.3.27]

PTEN; phosphatidylinositol-3,4,5-trisphosphate 3-phosphatase and dual-specificity protein phosphatase PTEN [EC:3.1.3.16 3.1.3.48 3.1.3.67]

PLD1_2; phospholipase D1/2 [EC:3.1.4.4]

cpdP; 3',5'-cyclic-nucleotide phosphodiesterase [EC:3.1.4.17]

SGA1; glucoamylase [EC:3.2.1.3]

IMA; oligo-1,6-glucosidase [EC:3.2.1.10]

E3.2.1.14; chitinase [EC:3.2.1.14]

malZ; alpha-glucosidase [EC:3.2.1.20]

lacZ; beta-galactosidase [EC:3.2.1.23]

INV; beta-fructofuranosidase [EC:3.2.1.26]

TREH; alpha,alpha-trehalase [EC:3.2.1.28]

AGL; glycogen debranching enzyme [EC:2.4.1.25 3.2.1.33]

nagZ; beta-N-acetylhexosaminidase [EC:3.2.1.52]

E3.2.1.58; glucan 1,3-beta-glucosidase [EC:3.2.1.58]

MOGS; mannosyl-oligosaccharide glucosidase [EC:3.2.1.106]

URH1; uridine nucleosidase [EC:3.2.2.3]

E3.3.1.1; adenosylhomocysteinase [EC:3.3.1.1]

LTA4H; leukotriene-A4 hydrolase [EC:3.3.2.6]

E3.5.1.1; L-asparaginase [EC:3.5.1.1]

E3.5.1.4; amidase [EC:3.5.1.4]

argE; acetylornithine deacetylase [EC:3.5.1.16]

PNC1; nicotinamidase [EC:3.5.1.19]

nagA; N-acetylglucosamine-6-phosphate deacetylase [EC:3.5.1.25]

E3.5.1.41; chitin deacetylase [EC:3.5.1.41]

E3.5.1.49; formamidase [EC:3.5.1.49]

URA4; dihydroorotase [EC:3.5.2.3]

allB; allantoinase [EC:3.5.2.5]

OPLAH; 5-oxoprolinase (ATP-hydrolysing) [EC:3.5.2.9]

E3.5.3.1; arginase [EC:3.5.3.1]

alc; allantoicase [EC:3.5.3.4]

speB; agmatinase [EC:3.5.3.11]

allA; ureidoglycolate lyase [EC:4.3.2.3]

E3.5.4.3; guanine deaminase [EC:3.5.4.3]

add; adenosine deaminase [EC:3.5.4.4]

AMPD; AMP deaminase [EC:3.5.4.6]

comEB; dCMP deaminase [EC:3.5.4.12]

GCH1; GTP cyclohydrolase IA [EC:3.5.4.16]

ribA; GTP cyclohydrolase II [EC:3.5.4.25]

E3.5.5.1; nitrilase [EC:3.5.5.1]

PRUNE; exopolyphosphatase [EC:3.6.1.11]

nudF; ADP-ribose pyrophosphatase [EC:3.6.1.13]

ITPA; inosine triphosphate pyrophosphatase [EC:3.6.1.-]

dut; dUTP pyrophosphatase [EC:3.6.1.23]

FHIT; bis(5'-adenosyl)-triphosphatase [EC:3.6.1.29]

PMA1; H+-transporting ATPase [EC:7.1.2.1]

TIM11; F-type H+-transporting ATP synthase subunit e

KYNU; kynureninase [EC:3.7.1.3]

FAHD1; acylpyruvate hydrolase [EC:3.7.1.5]

PDC; pyruvate decarboxylase [EC:4.1.1.1]

E4.1.1.15; glutamate decarboxylase [EC:4.1.1.15]

E4.1.1.17; ornithine decarboxylase [EC:4.1.1.17]

pyrF; orotidine-5'-phosphate decarboxylase [EC:4.1.1.23]

PPCDC; phosphopantothenoylcysteine decarboxylase [EC:4.1.1.36]

hemE; uroporphyrinogen decarboxylase [EC:4.1.1.37]

E4.1.1.49; phosphoenolpyruvate carboxykinase (ATP) [EC:4.1.1.49]

speD; S-adenosylmethionine decarboxylase [EC:4.1.1.50]

psd; phosphatidylserine decarboxylase [EC:4.1.1.65]

ltaE; threonine aldolase [EC:4.1.2.48]

FBA; fructose-bisphosphate aldolase, class II [EC:4.1.2.13]

E2.5.1.54; 3-deoxy-7-phosphoheptulonate synthase [EC:2.5.1.54]

SGPL1; sphinganine-1-phosphate aldolase [EC:4.1.2.27]

E4.1.3.1; isocitrate lyase [EC:4.1.3.1]

aceB; malate synthase [EC:2.3.3.9]

E2.3.3.10; hydroxymethylglutaryl-CoA synthase [EC:2.3.3.10]

CS; citrate synthase [EC:2.3.3.1]

leuA; 2-isopropylmalate synthase [EC:2.3.3.13]

E2.2.1.6L; acetolactate synthase I/II/III large subunit [EC:2.2.1.6]

E2.2.1.6S; acetolactate synthase I/III small subunit [EC:2.2.1.6]

LYS21; homocitrate synthase [EC:2.3.3.14]

TRP3; anthranilate synthase / indole-3-glycerol phosphate synthase [EC:4.1.3.27 4.1.1.48]

trpE; anthranilate synthase component I [EC:4.1.3.27]

HIS7; imidazole glycerol-phosphate synthase [EC:4.3.2.10]

cynT; carbonic anhydrase [EC:4.2.1.1]

E4.2.1.2B; fumarate hydratase, class II [EC:4.2.1.2]

ACO; aconitate hydratase [EC:4.2.1.3]

ilvD; dihydroxy-acid dehydratase [EC:4.2.1.9]

ENO; enolase [EC:4.2.1.11]

hisB; imidazoleglycerol-phosphate dehydratase [EC:4.2.1.19]

TRP; tryptophan synthase [EC:4.2.1.20]

CBS; cystathionine beta-synthase [EC:4.2.1.22]

hemB; porphobilinogen synthase [EC:4.2.1.24]

LEU1; 3-isopropylmalate dehydratase [EC:4.2.1.33]

LYS4; homoaconitate hydratase [EC:4.2.1.36]

rfbB; dTDP-glucose 4,6-dehydratase [EC:4.2.1.46]

dapA; 4-hydroxy-tetrahydrodipicolinate synthase [EC:4.3.3.7]

hemD; uroporphyrinogen-III synthase [EC:4.2.1.75]

PCBD; 4a-hydroxytetrahydrobiopterin dehydratase [EC:4.2.1.96]

thrC; threonine synthase [EC:4.2.3.1]

aroC; chorismate synthase [EC:4.2.3.5]

cysK; cysteine synthase [EC:2.5.1.47]
metB; cystathionine gamma-synthase [EC:2.5.1.48]
hemC; hydroxymethylbilane synthase [EC:2.5.1.61]
E4.3.1.19; threonine dehydratase [EC:4.3.1.19]
argH; argininosuccinate lyase [EC:4.3.2.1]
purB; adenylosuccinate lyase [EC:4.3.2.2]
CTH; cystathionine gamma-lyase [EC:4.4.1.1]
GLO1; lactoylglutathione lyase [EC:4.4.1.5]
metC; cysteine-S-conjugate beta-lyase [EC:4.4.1.13]
E4.6.1.1; adenylate cyclase [EC:4.6.1.1]
plc; 1-phosphatidylinositol phosphodiesterase [EC:4.6.1.13]
hemH; protoporphyrin/coproporphyrin ferrochelatase [EC:4.99.1.1 4.99.1.9]
rpe; ribulose-phosphate 3-epimerase [EC:5.1.3.1]
galM; aldose 1-epimerase [EC:5.1.3.3]
E5.1.3.15; glucose-6-phosphate 1-epimerase [EC:5.1.3.15]
TPI; triosephosphate isomerase (TIM) [EC:5.3.1.1]
rpiA; ribose 5-phosphate isomerase A [EC:5.3.1.6]
manA; mannose-6-phosphate isomerase [EC:5.3.1.8]
GPI; glucose-6-phosphate isomerase [EC:5.3.1.9]
hisA; phosphoribosylformimino-5-aminoimidazole carboxamide ribotide isomerase [EC:5.3.1.16]
trpF; phosphoribosylanthranilate isomerase [EC:5.3.1.24]
idi; isopentenyl-diphosphate Delta-isomerase [EC:5.3.3.2]
PGAM; 2,3-bisphosphoglycerate-dependent phosphoglycerate mutase [EC:5.4.2.11]
pgm; phosphoglucomutase [EC:5.4.2.2]
PGM3; phosphoacetylglucosamine mutase [EC:5.4.2.3]
E5.4.99.5; chorismate mutase [EC:5.4.99.5]
LSS; lanosterol synthase [EC:5.4.99.7]
INO1; myo-inositol-1-phosphate synthase [EC:5.5.1.4]
MARS; methionyl-tRNA synthetase [EC:6.1.1.10]
EARS; glutamyl-tRNA synthetase [EC:6.1.1.17]
QARS; glutaminyl-tRNA synthetase [EC:6.1.1.18]

ACSS1_2; acetyl-CoA synthetase [EC:6.2.1.1]

ACSL; long-chain acyl-CoA synthetase [EC:6.2.1.3]

LSC1; succinyl-CoA synthetase alpha subunit [EC:6.2.1.4 6.2.1.5]

LSC2; succinyl-CoA synthetase beta subunit [EC:6.2.1.4 6.2.1.5]

panC; pantoate--beta-alanine ligase [EC:6.3.2.1]

PPCS; phosphopantothenate---cysteine ligase (ATP) [EC:6.3.2.51]

purC; phosphoribosylaminoimidazole-succinocarboxamide synthase [EC:6.3.2.6]

FPGS; folylpolyglutamate synthase [EC:6.3.2.17]

pyrG; CTP synthase [EC:6.3.4.2]

purA; adenylosuccinate synthase [EC:6.3.4.4]

argG; argininosuccinate synthase [EC:6.3.4.5]

E6.3.4.6; urea carboxylase [EC:6.3.4.6]

HLCS; biotin---protein ligase [EC:6.3.4.9 6.3.4.10 6.3.4.11 6.3.4.15]

E6.3.5.1; NAD+ synthase (glutamine-hydrolysing) [EC:6.3.5.1]

guaA; GMP synthase (glutamine-hydrolysing) [EC:6.3.5.2]

PFAS; phosphoribosylformylglycinamidine synthase [EC:6.3.5.3]

asnB; asparagine synthase (glutamine-hydrolysing) [EC:6.3.5.4]

carA; carbamoyl-phosphate synthase small subunit [EC:6.3.5.5]

PC; pyruvate carboxylase [EC:6.4.1.1]

ATPeF0B; F-type H+-transporting ATPase subunit b

ATPeF1A; F-type H+-transporting ATPase subunit alpha

ATPeF1B; F-type H+-transporting ATPase subunit beta [EC:7.1.2.2]

ATPeF1D; F-type H+-transporting ATPase subunit delta

ATPeF1G; F-type H+-transporting ATPase subunit gamma

ATPeF0O; F-type H+-transporting ATPase subunit O

ATPeF0D; F-type H+-transporting ATPase subunit d

ATPeFG; F-type H+-transporting ATPase subunit g

ATPeFK; F-type H+-transporting ATPase subunit k

ATPeV1A; V-type H+-transporting ATPase subunit A [EC:7.1.2.2]

ATPeV0D; V-type H+-transporting ATPase subunit d

ATPeV1B; V-type H+-transporting ATPase subunit B

ATPeV1C; V-type H+-transporting ATPase subunit C

ATPeV1D; V-type H+-transporting ATPase subunit D

ATPeV1G; V-type H+-transporting ATPase subunit G

ATPeV0A; V-type H+-transporting ATPase subunit a

ATPeV0C; V-type H+-transporting ATPase 16kDa proteolipid subunit

E2.7.7.3B; pantetheine-phosphate adenylyltransferase [EC:2.7.7.3]

COX10; heme o synthase [EC:2.5.1.141]

COX11; cytochrome c oxidase assembly protein subunit 11

COX15; cytochrome c oxidase assembly protein subunit 15

COX5B; cytochrome c oxidase subunit 5b

COX6A; cytochrome c oxidase subunit 6a

COX6B; cytochrome c oxidase subunit 6b

COX7C; cytochrome c oxidase subunit 7c

gatA; aspartyl-tRNA(Asn)/glutamyl-tRNA(Gln) amidotransferase subunit A [EC:6.3.5.6 6.3.5.7]

gatB; aspartyl-tRNA(Asn)/glutamyl-tRNA(Gln) amidotransferase subunit B [EC:6.3.5.6 6.3.5.7]

gcvH; glycine cleavage system H protein

nagB; glucosamine-6-phosphate deaminase [EC:3.5.99.6]

ribB; 3,4-dihydroxy 2-butanone 4-phosphate synthase [EC:4.1.99.12]

THI4; cysteine-dependent adenosine diphosphate thiazole synthase [EC:2.4.2.60]

SLC33A1; MFS transporter, PAT family, solute carrier family 33 (acetyl-CoA transportor), member 1 [EC:2.3.1.-]

E3.6.1.22; NAD+ diphosphatase [EC:3.6.1.22]

PIGL; N-acetylglucosaminylphosphatidylinositol deacetylase [EC:3.5.1.89]

lipA; lipoyl synthase [EC:2.8.1.8]

ATPeV0B; V-type H+-transporting ATPase 21kDa proteolipid subunit

katE; catalase [EC:1.11.1.6]

punA; purine-nucleoside phosphorylase [EC:2.4.2.1]

aroQ; 3-dehydroquinate dehydratase II [EC:4.2.1.10]

surE; 5'-nucleotidase [EC:3.1.3.5]

wrbA; NAD(P)H dehydrogenase (quinone) [EC:1.6.5.2]

FBP; fructose-1,6-bisphosphatase I [EC:3.1.3.11]

ALG1; beta-1,4-mannosyltransferase [EC:2.4.1.142]

ALG2; alpha-1,3/alpha-1,6-mannosyltransferase [EC:2.4.1.132 2.4.1.257]

ALG11; alpha-1,2-mannosyltransferase [EC:2.4.1.131]

ALG3; alpha-1,3-mannosyltransferase [EC:2.4.1.258]

ALG9; alpha-1,2-mannosyltransferase [EC:2.4.1.259 2.4.1.261]

ALG12; alpha-1,6-mannosyltransferase [EC:2.4.1.260]

ALG6; alpha-1,3-glucosyltransferase [EC:2.4.1.267]

ALG10; alpha-1,2-glucosyltransferase [EC:2.4.1.256]

PIGA; phosphatidylinositol N-acetylglucosaminyltransferase subunit A [EC:2.4.1.198]

PIGH; phosphatidylinositol N-acetylglucosaminyltransferase subunit H

PIGC; phosphatidylinositol N-acetylglucosaminyltransferase subunit C

PIGQ; phosphatidylinositol N-acetylglucosaminyltransferase subunit Q

GTF1; glutamyl-tRNA(Gln) amidotransferase subunit F [EC:6.3.5.7]

NDUFS1; NADH dehydrogenase (ubiquinone) Fe-S protein 1 [EC:7.1.1.2 1.6.99.3]

NDUFS2; NADH dehydrogenase (ubiquinone) Fe-S protein 2 [EC:7.1.1.2 1.6.99.3]

NDUFS3; NADH dehydrogenase (ubiquinone) Fe-S protein 3 [EC:7.1.1.2 1.6.99.3]

NDUFS4; NADH dehydrogenase (ubiquinone) Fe-S protein 4

NDUFS6; NADH dehydrogenase (ubiquinone) Fe-S protein 6

NDUFS7; NADH dehydrogenase (ubiquinone) Fe-S protein 7 [EC:7.1.1.2 1.6.99.3]

NDUFS8; NADH dehydrogenase (ubiquinone) Fe-S protein 8 [EC:7.1.1.2 1.6.99.3]

NDUFV1; NADH dehydrogenase (ubiquinone) flavoprotein 1 [EC:7.1.1.2 1.6.99.3]

NDUFV2; NADH dehydrogenase (ubiquinone) flavoprotein 2 [EC:7.1.1.2 1.6.99.3]

NDUFA2; NADH dehydrogenase (ubiquinone) 1 alpha subcomplex subunit 2

NDUFA5; NADH dehydrogenase (ubiquinone) 1 alpha subcomplex subunit 5

NDUFA6; NADH dehydrogenase (ubiquinone) 1 alpha subcomplex subunit 6

NDUFA8; NADH dehydrogenase (ubiquinone) 1 alpha subcomplex subunit 8

NDUFA9; NADH dehydrogenase (ubiquinone) 1 alpha subcomplex subunit 9

NDUFAB1; NADH dehydrogenase (ubiquinone) 1 alpha/beta subcomplex 1, acyl-carrier protein

NDUFB8; NADH dehydrogenase (ubiquinone) 1 beta subcomplex subunit 8

NDUFB9; NADH dehydrogenase (ubiquinone) 1 beta subcomplex subunit 9

E3.1.3.15B; histidinol-phosphatase (PHP family) [EC:3.1.3.15]

iscS; cysteine desulfurase [EC:2.8.1.7]

KDSR; 3-dehydrosphinganine reductase [EC:1.1.1.102]

LAG1; Acyl-CoA-dependent ceramide synthase [EC:2.3.1.24]

ACER3; dihydroceramidase [EC:3.5.1.-]

DEGS; sphingolipid 4-desaturase/C4-monooxygenase [EC:1.14.19.17 1.14.18.5]

SUR2; sphinganine C4-monooxygenase [EC:1.14.18.5]

SPHK; sphingosine kinase [EC:2.7.1.91]

E1.1.1.65; pyridoxine 4-dehydrogenase [EC:1.1.1.65]

PIGW; glucosaminylphosphatidylinositol acyltransferase [EC:2.3.-.-]

PIGM; GPI mannosyltransferase 1 subunit M [EC:2.4.1.-]

PIGN; GPI ethanolamine phosphate transferase 1 [EC:2.7.-.-]

PIGB; GPI mannosyltransferase 3 [EC:2.4.1.-]

PIGF; GPI ethanolamine phosphate transferase 2/3 subunit F

PIGO; GPI ethanolamine phosphate transferase 3 subunit O [EC:2.7.-.-]

GAA1; GPI-anchor transamidase subunit GAA1

PIGK; GPI-anchor transamidase subunit K

PIGS; GPI-anchor transamidase subunit S

PIGT; GPI-anchor transamidase subunit T

PIGU; GPI-anchor transamidase subunit U

PGAP1; GPI inositol-deacylase [EC:3.-.-.-]

bglX; beta-glucosidase [EC:3.2.1.21]

E1.1.1.9; D-xylulose reductase [EC:1.1.1.9]

OCH1; alpha 1,6-mannosyltransferase [EC:2.4.1.232]

MNN9; mannan polymerase complexes MNN9 subunit [EC:2.4.1.232]

MNN10; mannan polymerase II complex MNN10 subunit [EC:2.4.1.-]

MNN11; mannan polymerase II complex MNN11 subunit [EC:2.4.1.-]

ANP1; mannan polymerase II complex ANP1 subunit [EC:2.4.1.-]

HOC1; mannan polymerase II complex HOC1 subunit [EC:2.4.1.-]

MNN2; alpha 1,2-mannosyltransferase [EC:2.4.1.-]

MNN1; alpha 1,3-mannosyltransferase [EC:2.4.1.-]

HIBCH; 3-hydroxyisobutyryl-CoA hydrolase [EC:3.1.2.4]

ARO9; aromatic amino acid aminotransferase II [EC:2.6.1.58 2.6.1.28]

LYS12; homoisocitrate dehydrogenase [EC:1.1.1.87]

PLCD; phosphatidylinositol phospholipase C, delta [EC:3.1.4.11]

CYP51; sterol 14alpha-demethylase [EC:1.14.14.154 1.14.15.36]

GPP; glycerol-1-phosphatase [EC:3.1.3.21]

AYR1; 1-acylglycerone phosphate reductase [EC:1.1.1.101]

COQ2; 4-hydroxybenzoate polyprenyltransferase [EC:2.5.1.39]

COQ6; ubiquinone biosynthesis monooxygenase Coq6 [EC:1.14.13.-]

COQ5; 2-methoxy-6-polyprenyl-1,4-benzoquinol methylase [EC:2.1.1.201]

COQ7; 3-demethoxyubiquinol 3-hydroxylase [EC:1.14.99.60]

NMNAT; nicotinamide mononucleotide adenylyltransferase [EC:2.7.7.1 2.7.7.18]

uraH; 5-hydroxyisourate hydrolase [EC:3.5.2.17]

STT3; dolichyl-diphosphooligosaccharide---protein glycosyltransferase [EC:2.4.99.18]

CHAC; glutathione-specific gamma-glutamylcyclotransferase [EC:4.3.2.7]

E3.2.1.22B; alpha-galactosidase [EC:3.2.1.22]

ALG13; beta-1,4-N-acetylglucosaminyltransferase [EC:2.4.1.141]

MECR; mitochondrial enoyl-[acyl-carrier protein] reductase / trans-2-enoyl-CoA reductase [EC:1.3.1.- 1.3.1.38]

ACAA1; acetyl-CoA acyltransferase 1 [EC:2.3.1.16]

PIGV; GPI mannosyltransferase 2 [EC:2.4.1.-]

NSDHL; sterol-4alpha-carboxylate 3-dehydrogenase (decarboxylating) [EC:1.1.1.170]

MESO1; methylsterol monooxygenase [EC:1.14.18.9]

CRLS; cardiolipin synthase (CMP-forming) [EC:2.7.8.41]

mtnA; methylthioribose-1-phosphate isomerase [EC:5.3.1.23]

mtnB; methylthioribulose-1-phosphate dehydratase [EC:4.2.1.109]

mtnD; 1,2-dihydroxy-3-keto-5-methylthiopentene dioxygenase [EC:1.13.11.53 1.13.11.54]

fabF; 3-oxoacyl-[acyl-carrier-protein] synthase II [EC:2.3.1.179]

DPM2; dolichol phosphate-mannose biosynthesis regulatory protein

coaW; type II pantothenate kinase [EC:2.7.1.33]

ERG27; 3-keto steroid reductase [EC:1.1.1.270]

ERG2; C-8 sterol isomerase [EC:5.-.-.-]

ERG5; sterol 22-desaturase [EC:1.14.19.41]

mtnC; enolase-phosphatase E1 [EC:3.1.3.77]

ELO2; fatty acid elongase 2 [EC:2.3.1.199]

ELO3; fatty acid elongase 3 [EC:2.3.1.199]

HSD17B12; 17beta-estradiol 17-dehydrogenase / very-long-chain 3-oxoacyl-CoA reductase [EC:1.1.1.62 1.1.1.330]

FAD2; omega-6 fatty acid desaturase / acyl-lipid omega-6 desaturase (Delta-12 desaturase) [EC:1.14.19.6 1.14.19.22]

TER; very-long-chain enoyl-CoA reductase [EC:1.3.1.93]

PHAA; phenylacetate 2-hydroxylase [EC:1.14.14.54]

NRK1_2; nicotinamide/nicotinate riboside kinase [EC:2.7.1.22 2.7.1.173]

HACD; very-long-chain (3R)-3-hydroxyacyl-CoA dehydratase [EC:4.2.1.134]

RRM1; ribonucleoside-diphosphate reductase subunit M1 [EC:1.17.4.1]

RRM2; ribonucleoside-diphosphate reductase subunit M2 [EC:1.17.4.1]

SIR2; NAD+-dependent protein deacetylase SIR2 [EC:2.3.1.286]

GCLC; glutamate--cysteine ligase catalytic subunit [EC:6.3.2.2]

ACACA; acetyl-CoA carboxylase / biotin carboxylase 1 [EC:6.4.1.2 6.3.4.14 2.1.3.15]

NDUFA12; NADH dehydrogenase (ubiquinone) 1 alpha subcomplex subunit 12

SIRT5; NAD+-dependent protein deacetylase sirtuin 5 [EC:2.3.1.286]

SETD1; [histone H3]-lysine4 N-trimethyltransferase SETD1 [EC:2.1.1.354]

SMYD; [histone H3]-lysine4/36 N-trimethyltransferase SMYD [EC:2.1.1.354 2.1.1.357]

DOT1L; [histone H3]-lysine79 N-trimethyltransferase [EC:2.1.1.360]

HAO; (S)-2-hydroxy-acid oxidase [EC:1.1.3.15]

URA2; carbamoyl-phosphate synthase / aspartate carbamoyltransferase [EC:6.3.5.5 2.1.3.2]

ADE5; phosphoribosylamine--glycine ligase / phosphoribosylformylglycinamidine cyclo-ligase [EC:6.3.4.13 6.3.3.1]

ADE2; phosphoribosylaminoimidazole carboxylase [EC:4.1.1.21]

SMPD2; sphingomyelin phosphodiesterase 2 [EC:3.1.4.12]

HEXA_B; hexosaminidase [EC:3.2.1.52]

ARG56; N-acetyl-gamma-glutamyl-phosphate reductase / acetylglutamate kinase [EC:1.2.1.38 2.7.2.8]

OST1; oligosaccharyltransferase complex subunit alpha (ribophorin I)

SWP1; oligosaccharyltransferase complex subunit delta (ribophorin II)

OST2; oligosaccharyltransferase complex subunit epsilon

OST3; oligosaccharyltransferase complex subunit gamma

WBP1; oligosaccharyltransferase complex subunit beta

ARO10; phenylpyruvate decarboxylase [EC:4.1.1.-]

FMS1; polyamine oxidase [EC:1.5.3.17]

GAT; glycerol-3-phosphate O-acyltransferase / dihydroxyacetone phosphate acyltransferase [EC:2.3.1.15 2.3.1.42]

AGPAT1_2; lysophosphatidate acyltransferase [EC:2.3.1.51]

LPT1; lysophospholipid acyltransferase [EC:2.3.1.51 2.3.1.23 2.3.1.-]

ABAT; 4-aminobutyrate aminotransferase / (S)-3-amino-2-methylpropionate transaminase [EC:2.6.1.19 2.6.1.22]

NIT2; omega-amidase [EC:3.5.1.3]

PI4K2; phosphatidylinositol 4-kinase type 2 [EC:2.7.1.67]

CMPK1; UMP-CMP kinase [EC:2.7.4.14]

ARO1; pentafunctional AROM polypeptide [EC:4.2.3.4 4.2.1.10 1.1.1.25 2.7.1.71 2.5.1.19]

FOL1; dihydroneopterin aldolase / 2-amino-4-hydroxy-6-hydroxymethyldihydropteridine diphosphokinase / dihydropteroate synthase [EC:4.1.2.25 2.7.6.3 2.5.1.15]

adhP; alcohol dehydrogenase, propanol-preferring [EC:1.1.1.1]

HIS4; phosphoribosyl-ATP pyrophosphohydrolase / phosphoribosyl-AMP cyclohydrolase / histidinol dehydrogenase [EC:3.6.1.31 3.5.4.19 1.1.1.23]

THI6; thiamine-phosphate diphosphorylase / hydroxyethylthiazole kinase [EC:2.5.1.3 2.7.1.50]

BNA7; kynurenine formamidase [EC:3.5.1.9]

BNA3; kynurenine aminotransferase [EC:2.6.1.7]

ACP1; low molecular weight phosphotyrosine protein phosphatase [EC:3.1.3.2 3.1.3.48]

GOT1; aspartate aminotransferase, cytoplasmic [EC:2.6.1.1]

GOT2; aspartate aminotransferase, mitochondrial [EC:2.6.1.1]

YND1; golgi apyrase [EC:3.6.1.5]

RIB7; 2,5-diamino-6-(ribosylamino)-4(3H)-pyrimidinone 5'-phosphate reductase [EC:1.1.1.302]

RIB2; tRNA pseudouridine32 synthase / 2,5-diamino-6-(5-phospho-D-ribitylamino)-pyrimidin-4(3H)-one deaminase [EC:5.4.99.28]

TGL4; TAG lipase / steryl ester hydrolase / phospholipase A2 / LPA acyltransferase [EC:3.1.1.3 3.1.1.13 3.1.1.4 2.3.1.51]

TGL3; TAG lipase / lysophosphatidylethanolamine acyltransferase [EC:3.1.1.3 2.3.1.-]

FOX2; multifunctional beta-oxidation protein [EC:4.2.1.- 1.1.1.-]

GDH2; glutamate dehydrogenase [EC:1.4.1.2]

DUG1; Cys-Gly metallodipeptidase DUG1 [EC:3.4.13.-]

LPIN; phosphatidate phosphatase LPIN [EC:3.1.3.4]

GLYK; D-glycerate 3-kinase [EC:2.7.1.31]

TPS; trehalose 6-phosphate synthase/phosphatase [EC:2.4.1.15 3.1.3.12]

ydfG; 3-hydroxy acid dehydrogenase/ malonic semialdehyde reductase [EC:1.1.1.381 1.1.1.-]

psuG; pseudouridylate synthase [EC:4.2.1.70]

CHO2; phosphatidylethanolamine N-methyltransferase [EC:2.1.1.17]

PAFAH1B1; platelet-activating factor acetylhydrolase IB subunit alpha

MET17; O-acetylhomoserine/O-acetylserine sulfhydrylase [EC:2.5.1.49 2.5.1.47]

DAS; dihydroxyacetone synthase [EC:2.2.1.3]

CHO1; CDP-diacylglycerol---serine O-phosphatidyltransferase [EC:2.7.8.8]

ACO2; homoaconitase [EC:4.2.1.-]

PMM; phosphomannomutase [EC:5.4.2.8]

ARD; D-arabinitol 2-dehydrogenase [EC:1.1.1.250]

GRE2; NADPH-dependent methylglyoxal reductase [EC:1.1.1.283]

SOU1; sorbose reductase [EC:1.1.1.289]

XR; D-xylose reductase [EC:1.1.1.307]

ATG15; lipase ATG15 [EC:3.1.1.3]

SDS; L-serine/L-threonine ammonia-lyase [EC:4.3.1.17 4.3.1.19]

MTMR6_7_8; myotubularin-related protein 6/7/8 [EC:3.1.3.64 3.1.3.95]

GCY1; glycerol 2-dehydrogenase (NADP+) [EC:1.1.1.156]

GAAB; L-galactonate dehydratase [EC:4.2.1.146]

THI5; pyrimidine precursor biosynthesis enzyme

LRA4; 2-keto-3-deoxy-L-rhamnonate aldolase [EC:4.1.2.53]

AK6; adenylate kinase [EC:2.7.4.3]

ISN1; IMP and pyridine-specific 5'-nucleotidase [EC:3.1.3.99 3.1.3.-]

CTM1; [cytochrome c]-lysine N-methyltransferase [EC:2.1.1.59]

HPA3; D-amino-acid N-acetyltransferase [EC:2.3.1.36]

PFKFB2; 6-phosphofructo-2-kinase / fructose-2,6-biphosphatase 2 [EC:2.7.1.105 3.1.3.46]

IAL; isopenicillin-N N-acyltransferase like protein

POF1; nicotinamide-nucleotide adenylyltransferase [EC:2.7.7.1]

IPK1; inositol-pentakisphosphate 2-kinase [EC:2.7.1.158]

CARNMT1; carnosine N-methyltransferase [EC:2.1.1.22]

PI4KB; phosphatidylinositol 4-kinase B [EC:2.7.1.67]

HPT1; hypoxanthine phosphoribosyltransferase [EC:2.4.2.8]

EGT2; hercynylcysteine S-oxide lyase [EC:4.4.1.36]

SYNJ; synaptojanin [EC:3.1.3.36]

DHFS; dihydrofolate synthase [EC:6.3.2.12]

DSD1; D-serine ammonia-lyase [EC:4.3.1.18]

GSS; glutathione synthase [EC:6.3.2.3]

DLD3; (R)-2-hydroxyglutarate---pyruvate transhydrogenase [EC:1.1.99.40]

SAC1; phosphatidylinositol 4-phosphatase [EC:3.1.3.-]

GLX3; D-lactate dehydratase [EC:4.2.1.130]

TSL1; trehalose 6-phosphate synthase complex regulatory subunit

HTD2; 3-hydroxyacyl-thioester dehydratase, fungi type [EC:4.2.1.-]

LOA1; 1-acylglycerol-3-phosphate O-acyltransferase [EC:2.3.1.51]

FIG4; phosphatidylinositol 3,5-bisphosphate 5-phosphatase [EC:3.1.3.-]

FAD3; omega-3 fatty acid desaturase (Delta-15 desaturase) [EC:1.14.19.-]

HPD1; 3-hydroxyisobutyrate/3-hydroxypropionate dehydrogenase [EC:1.1.1.31 1.1.1.59]

LCB3; dihydrosphingosine 1-phosphate phosphatase [EC:3.1.3.-]

SET2; [histone H3]-lysine36 N-trimethyltransferase [EC:2.1.1.359]

LIPT2; lipoyl(octanoyl) transferase 2 [EC:2.3.1.181]

MAN1B; endoplasmic reticulum Man9GlcNAc2 1,2-alpha-mannosidase [EC:3.2.1.209]

LIP3; octanoyl-CoA:protein transferase [EC:2.3.1.-]

PRX1; glutaredoxin/glutathione-dependent peroxiredoxin [EC:1.11.1.25 1.11.1.27]

2. Biosynthesis of secondary metabolites

AKR1A1; alcohol dehydrogenase (NADP+) [EC:1.1.1.2]

hom; homoserine dehydrogenase [EC:1.1.1.3]

GPD1; glycerol-3-phosphate dehydrogenase (NAD+) [EC:1.1.1.8]

gyaR; glyoxylate reductase [EC:1.1.1.26]

HMGCR; hydroxymethylglutaryl-CoA reductase (NADPH) [EC:1.1.1.34]

MDH2; malate dehydrogenase [EC:1.1.1.37]

IDH3; isocitrate dehydrogenase (NAD+) [EC:1.1.1.41]

IDH1; isocitrate dehydrogenase [EC:1.1.1.42]

PGD; 6-phosphogluconate dehydrogenase [EC:1.1.1.44 1.1.1.343]

G6PD; glucose-6-phosphate 1-dehydrogenase [EC:1.1.1.49 1.1.1.363]

leuB; 3-isopropylmalate dehydrogenase [EC:1.1.1.85]

ilvC; ketol-acid reductoisomerase [EC:1.1.1.86]

serA; D-3-phosphoglycerate dehydrogenase / 2-oxoglutarate reductase [EC:1.1.1.95 1.1.1.399]

fabG; 3-oxoacyl-[acyl-carrier protein] reductase [EC:1.1.1.100]

panE; 2-dehydropantoate 2-reductase [EC:1.1.1.169]

IMPDH; IMP dehydrogenase [EC:1.1.1.205]

glpA; glycerol-3-phosphate dehydrogenase [EC:1.1.5.3]

frmA; S-(hydroxymethyl)glutathione dehydrogenase / alcohol dehydrogenase [EC:1.1.1.284 1.1.1.1]

ALDH; aldehyde dehydrogenase (NAD+) [EC:1.2.1.3]

E1.2.1.5; aldehyde dehydrogenase (NAD(P)+) [EC:1.2.1.5]

asd; aspartate-semialdehyde dehydrogenase [EC:1.2.1.11]

GAPDH; glyceraldehyde 3-phosphate dehydrogenase [EC:1.2.1.12]

LYS2; L-2-aminoadipate reductase [EC:1.2.1.95]

proA; glutamate-5-semialdehyde dehydrogenase [EC:1.2.1.41]

PDHA; pyruvate dehydrogenase E1 component alpha subunit [EC:1.2.4.1]

PDHB; pyruvate dehydrogenase E1 component beta subunit [EC:1.2.4.1]

OGDH; 2-oxoglutarate dehydrogenase E1 component [EC:1.2.4.2]

TYR1; prephenate dehydrogenase (NADP+) [EC:1.3.1.13]

TM7SF2; Delta14-sterol reductase [EC:1.3.1.70]

ERG4; Delta24(24(1))-sterol reductase [EC:1.3.1.71]

PPOX; protoporphyrinogen/coproporphyrinogen III oxidase [EC:1.3.3.4 1.3.3.15]

E1.3.3.6; acyl-CoA oxidase [EC:1.3.3.6]

SDHA; succinate dehydrogenase (ubiquinone) flavoprotein subunit [EC:1.3.5.1]

SDHB; succinate dehydrogenase (ubiquinone) iron-sulfur subunit [EC:1.3.5.1]

SDHD; succinate dehydrogenase (ubiquinone) membrane anchor subunit

ACADM; acyl-CoA dehydrogenase [EC:1.3.8.7]

DAO; D-amino-acid oxidase [EC:1.4.3.3]

AOC3; primary-amine oxidase [EC:1.4.3.21]

GLDC; glycine dehydrogenase [EC:1.4.4.2]

proC; pyrroline-5-carboxylate reductase [EC:1.5.1.2]

LYS1; saccharopine dehydrogenase (NAD+, L-lysine forming) [EC:1.5.1.7]

LYS9; saccharopine dehydrogenase (NADP+, L-glutamate forming) [EC:1.5.1.10]

PRODH; proline dehydrogenase [EC:1.5.5.2]

DLD; dihydrolipoamide dehydrogenase [EC:1.8.1.4]

SQLE; squalene monooxygenase [EC:1.14.14.17]

mmuM; homocysteine S-methyltransferase [EC:2.1.1.10]

metE; 5-methyltetrahydropteroyltriglutamate--homocysteine methyltransferase [EC:2.1.1.14]

PEMT; phosphatidylethanolamine/phosphatidyl-N-methylethanolamine N-methyltransferase [EC:2.1.1.17 2.1.1.71]

SMT1; sterol 24-C-methyltransferase [EC:2.1.1.41]

ICMT; protein-S-isoprenylcysteine O-methyltransferase [EC:2.1.1.100]

MET1; uroporphyrin-III C-methyltransferase [EC:2.1.1.107]

COQ3; polyprenyldihydroxybenzoate methyltransferase / 3-demethylubiquinol 3-O-methyltransferase [EC:2.1.1.114 2.1.1.64]

glyA; glycine hydroxymethyltransferase [EC:2.1.2.1]

E2.1.2.2; phosphoribosylglycinamide formyltransferase [EC:2.1.2.2]

purH; phosphoribosylaminoimidazolecarboxamide formyltransferase / IMP cyclohydrolase [EC:2.1.2.3 3.5.4.10]

gcvT; aminomethyltransferase [EC:2.1.2.10]

panB; 3-methyl-2-oxobutanoate hydroxymethyltransferase [EC:2.1.2.11]

OTC; ornithine carbamoyltransferase [EC:2.1.3.3]

E2.2.1.1; transketolase [EC:2.2.1.1]

E2.2.1.2; transaldolase [EC:2.2.1.2]

ARG2; amino-acid N-acetyltransferase [EC:2.3.1.1]

argJ; glutamate N-acetyltransferase / amino-acid N-acetyltransferase [EC:2.3.1.35 2.3.1.1]

ACAT; acetyl-CoA C-acetyltransferase [EC:2.3.1.9]

DLAT; pyruvate dehydrogenase E2 component (dihydrolipoamide acetyltransferase) [EC:2.3.1.12]

metX; homoserine O-acetyltransferase/O-succinyltransferase [EC:2.3.1.31 2.3.1.46]

E2.3.1.37; 5-aminolevulinate synthase [EC:2.3.1.37]

fabD; [acyl-carrier-protein] S-malonyltransferase [EC:2.3.1.39]

DLST; 2-oxoglutarate dehydrogenase E2 component (dihydrolipoamide succinyltransferase) [EC:2.3.1.61]

PYG; glycogen phosphorylase [EC:2.4.1.1]

GYS; glycogen synthase [EC:2.4.1.11]

otsA; trehalose 6-phosphate synthase [EC:2.4.1.15 2.4.1.347]

GBE1; 1,4-alpha-glucan branching enzyme [EC:2.4.1.18]

GYG1; glycogenin [EC:2.4.1.186]

purF; amidophosphoribosyltransferase [EC:2.4.2.14]

trpD; anthranilate phosphoribosyltransferase [EC:2.4.2.18]

FDPS; farnesyl diphosphate synthase [EC:2.5.1.1 2.5.1.10]

metK; S-adenosylmethionine synthetase [EC:2.5.1.6]

miaA; tRNA dimethylallyltransferase [EC:2.5.1.75]

ribE; riboflavin synthase [EC:2.5.1.9]

FDFT1; farnesyl-diphosphate farnesyltransferase [EC:2.5.1.21]

GGPS1; geranylgeranyl diphosphate synthase, type III [EC:2.5.1.1 2.5.1.10 2.5.1.29]

hisC; histidinol-phosphate aminotransferase [EC:2.6.1.9]

E2.6.1.11; acetylornithine aminotransferase [EC:2.6.1.11]

rocD; ornithine--oxo-acid transaminase [EC:2.6.1.13]

E2.6.1.42; branched-chain amino acid aminotransferase [EC:2.6.1.42]

AGXT; alanine-glyoxylate transaminase / serine-glyoxylate transaminase / serine-pyruvate transaminase [EC:2.6.1.44 2.6.1.45 2.6.1.51]

serC; phosphoserine aminotransferase [EC:2.6.1.52]

ARO8; aromatic amino acid aminotransferase I / 2-aminoadipate transaminase [EC:2.6.1.57 2.6.1.39 2.6.1.27 2.6.1.5]

HK; hexokinase [EC:2.7.1.1]

pfkA; 6-phosphofructokinase 1 [EC:2.7.1.11]

E2.7.1.12; gluconokinase [EC:2.7.1.12]

RFK; riboflavin kinase [EC:2.7.1.26]

E2.7.1.36; mevalonate kinase [EC:2.7.1.36]

thrB; homoserine kinase [EC:2.7.1.39]

PK; pyruvate kinase [EC:2.7.1.40]

PGK; phosphoglycerate kinase [EC:2.7.2.3]

lysC; aspartate kinase [EC:2.7.2.4]

proB; glutamate 5-kinase [EC:2.7.2.11]

E2.7.4.2; phosphomevalonate kinase [EC:2.7.4.2]

adk; adenylate kinase [EC:2.7.4.3]

ndk; nucleoside-diphosphate kinase [EC:2.7.4.6]

PRPS; ribose-phosphate pyrophosphokinase [EC:2.7.6.1]

FLAD1; FAD synthetase [EC:2.7.7.2]

sat; sulfate adenylyltransferase [EC:2.7.7.4]

UGP2; UTP--glucose-1-phosphate uridylyltransferase [EC:2.7.7.9]

GMPP; mannose-1-phosphate guanylyltransferase [EC:2.7.7.13]

EPT1; ethanolaminephosphotransferase [EC:2.7.8.1]

PGLS; 6-phosphogluconolactonase [EC:3.1.1.31]

serB; phosphoserine phosphatase [EC:3.1.3.3]

PLD1_2; phospholipase D1/2 [EC:3.1.4.4]

malZ; alpha-glucosidase [EC:3.2.1.20]

INV; beta-fructofuranosidase [EC:3.2.1.26]

TREH; alpha,alpha-trehalase [EC:3.2.1.28]

AGL; glycogen debranching enzyme [EC:2.4.1.25 3.2.1.33]

E3.5.1.1; L-asparaginase [EC:3.5.1.1]

argE; acetylornithine deacetylase [EC:3.5.1.16]

E3.5.3.1; arginase [EC:3.5.3.1]

AMPD; AMP deaminase [EC:3.5.4.6]

ribA; GTP cyclohydrolase II [EC:3.5.4.25]

PDC; pyruvate decarboxylase [EC:4.1.1.1]

E4.1.1.15; glutamate decarboxylase [EC:4.1.1.15]

E4.1.1.17; ornithine decarboxylase [EC:4.1.1.17]

hemE; uroporphyrinogen decarboxylase [EC:4.1.1.37]

E4.1.1.49; phosphoenolpyruvate carboxykinase (ATP) [EC:4.1.1.49]

psd; phosphatidylserine decarboxylase [EC:4.1.1.65]

ltaE; threonine aldolase [EC:4.1.2.48]

FBA; fructose-bisphosphate aldolase, class II [EC:4.1.2.13]

E2.5.1.54; 3-deoxy-7-phosphoheptulonate synthase [EC:2.5.1.54]

E4.1.3.1; isocitrate lyase [EC:4.1.3.1]

aceB; malate synthase [EC:2.3.3.9]

E2.3.3.10; hydroxymethylglutaryl-CoA synthase [EC:2.3.3.10]

CS; citrate synthase [EC:2.3.3.1]

leuA; 2-isopropylmalate synthase [EC:2.3.3.13]

E2.2.1.6L; acetolactate synthase I/II/III large subunit [EC:2.2.1.6]

E2.2.1.6S; acetolactate synthase I/III small subunit [EC:2.2.1.6]

LYS21; homocitrate synthase [EC:2.3.3.14]

TRP3; anthranilate synthase / indole-3-glycerol phosphate synthase [EC:4.1.3.27 4.1.1.48]

trpE; anthranilate synthase component I [EC:4.1.3.27]

HIS7; imidazole glycerol-phosphate synthase [EC:4.3.2.10]

E4.2.1.2B; fumarate hydratase, class II [EC:4.2.1.2]

ACO; aconitate hydratase [EC:4.2.1.3]

ilvD; dihydroxy-acid dehydratase [EC:4.2.1.9]

ENO; enolase [EC:4.2.1.11]

hisB; imidazoleglycerol-phosphate dehydratase [EC:4.2.1.19]

TRP; tryptophan synthase [EC:4.2.1.20]

CBS; cystathionine beta-synthase [EC:4.2.1.22]

hemB; porphobilinogen synthase [EC:4.2.1.24]

LEU1; 3-isopropylmalate dehydratase [EC:4.2.1.33]

LYS4; homoaconitate hydratase [EC:4.2.1.36]

rfbB; dTDP-glucose 4,6-dehydratase [EC:4.2.1.46]

dapA; 4-hydroxy-tetrahydrodipicolinate synthase [EC:4.3.3.7]

hemD; uroporphyrinogen-III synthase [EC:4.2.1.75]

thrC; threonine synthase [EC:4.2.3.1]

aroC; chorismate synthase [EC:4.2.3.5]

cysK; cysteine synthase [EC:2.5.1.47]

metB; cystathionine gamma-synthase [EC:2.5.1.48]

hemC; hydroxymethylbilane synthase [EC:2.5.1.61]

E4.3.1.19; threonine dehydratase [EC:4.3.1.19]

argH; argininosuccinate lyase [EC:4.3.2.1]

purB; adenylosuccinate lyase [EC:4.3.2.2]

CTH; cystathionine gamma-lyase [EC:4.4.1.1]

metC; cysteine-S-conjugate beta-lyase [EC:4.4.1.13]

hemH; protoporphyrin/coproporphyrin ferrochelatase [EC:4.99.1.1 4.99.1.9]

rpe; ribulose-phosphate 3-epimerase [EC:5.1.3.1]

galM; aldose 1-epimerase [EC:5.1.3.3]

E5.1.3.15; glucose-6-phosphate 1-epimerase [EC:5.1.3.15]

TPI; triosephosphate isomerase (TIM) [EC:5.3.1.1]

rpiA; ribose 5-phosphate isomerase A [EC:5.3.1.6]

manA; mannose-6-phosphate isomerase [EC:5.3.1.8]

GPI; glucose-6-phosphate isomerase [EC:5.3.1.9]

hisA; phosphoribosylformimino-5-aminoimidazole carboxamide ribotide isomerase [EC:5.3.1.16]

trpF; phosphoribosylanthranilate isomerase [EC:5.3.1.24]

idi; isopentenyl-diphosphate Delta-isomerase [EC:5.3.3.2]

PGAM; 2,3-bisphosphoglycerate-dependent phosphoglycerate mutase [EC:5.4.2.11]

pgm; phosphoglucomutase [EC:5.4.2.2]

E5.4.99.5; chorismate mutase [EC:5.4.99.5]

LSS; lanosterol synthase [EC:5.4.99.7]

INO1; myo-inositol-1-phosphate synthase [EC:5.5.1.4]

EARS; glutamyl-tRNA synthetase [EC:6.1.1.17]

ACSS1_2; acetyl-CoA synthetase [EC:6.2.1.1]

LSC1; succinyl-CoA synthetase alpha subunit [EC:6.2.1.4 6.2.1.5]

LSC2; succinyl-CoA synthetase beta subunit [EC:6.2.1.4 6.2.1.5]

panC; pantoate--beta-alanine ligase [EC:6.3.2.1]

purC; phosphoribosylaminoimidazole-succinocarboxamide synthase [EC:6.3.2.6]

argG; argininosuccinate synthase [EC:6.3.4.5]

PFAS; phosphoribosylformylglycinamidine synthase [EC:6.3.5.3]

asnB; asparagine synthase (glutamine-hydrolysing) [EC:6.3.5.4]

COX10; heme o synthase [EC:2.5.1.141]

COX15; cytochrome c oxidase assembly protein subunit 15

gcvH; glycine cleavage system H protein

ribB; 3,4-dihydroxy 2-butanone 4-phosphate synthase [EC:4.1.99.12]

katE; catalase [EC:1.11.1.6]

punA; purine-nucleoside phosphorylase [EC:2.4.2.1]

aroQ; 3-dehydroquinate dehydratase II [EC:4.2.1.10]

surE; 5'-nucleotidase [EC:3.1.3.5]

wrbA; NAD(P)H dehydrogenase (quinone) [EC:1.6.5.2]

FBP; fructose-1,6-bisphosphatase I [EC:3.1.3.11]

E3.1.3.15B; histidinol-phosphatase (PHP family) [EC:3.1.3.15]

bglX; beta-glucosidase [EC:3.2.1.21]

hexPS; hexaprenyl-diphosphate synthase [EC:2.5.1.82 2.5.1.83]

ARO9; aromatic amino acid aminotransferase II [EC:2.6.1.58 2.6.1.28]

LYS12; homoisocitrate dehydrogenase [EC:1.1.1.87]

CYP51; sterol 14alpha-demethylase [EC:1.14.14.154 1.14.15.36]

FNTB; protein farnesyltransferase subunit beta [EC:2.5.1.58]

FNTA; protein farnesyltransferase/geranylgeranyltransferase type-1 subunit alpha [EC:2.5.1.58 2.5.1.59]

STE24; STE24 endopeptidase [EC:3.4.24.84]

COQ2; 4-hydroxybenzoate polyprenyltransferase [EC:2.5.1.39]

COQ6; ubiquinone biosynthesis monooxygenase Coq6 [EC:1.14.13.-]

COQ5; 2-methoxy-6-polyprenyl-1,4-benzoquinol methylase [EC:2.1.1.201]

COQ7; 3-demethoxyubiquinol 3-hydroxylase [EC:1.14.99.60]

ACAA1; acetyl-CoA acyltransferase 1 [EC:2.3.1.16]

NSDHL; sterol-4alpha-carboxylate 3-dehydrogenase (decarboxylating) [EC:1.1.1.170]

MESO1; methylsterol monooxygenase [EC:1.14.18.9]

ERG27; 3-keto steroid reductase [EC:1.1.1.270]

ERG2; C-8 sterol isomerase [EC:5.-.-.-]

ERG5; sterol 22-desaturase [EC:1.14.19.41]

ELO2; fatty acid elongase 2 [EC:2.3.1.199]

ELO3; fatty acid elongase 3 [EC:2.3.1.199]

HSD17B12; 17beta-estradiol 17-dehydrogenase / very-long-chain 3-oxoacyl-CoA reductase [EC:1.1.1.62 1.1.1.330]

TER; very-long-chain enoyl-CoA reductase [EC:1.3.1.93]

HACD; very-long-chain (3R)-3-hydroxyacyl-CoA dehydratase [EC:4.2.1.134]

ACACA; acetyl-CoA carboxylase / biotin carboxylase 1 [EC:6.4.1.2 6.3.4.14 2.1.3.15]

HAO; (S)-2-hydroxy-acid oxidase [EC:1.1.3.15]

DHDDS; ditrans,polycis-polyprenyl diphosphate synthase [EC:2.5.1.87]

ADE5; phosphoribosylamine--glycine ligase / phosphoribosylformylglycinamidine cyclo-ligase [EC:6.3.4.13 6.3.3.1]

ADE2; phosphoribosylaminoimidazole carboxylase [EC:4.1.1.21]

ARG56; N-acetyl-gamma-glutamyl-phosphate reductase / acetylglutamate kinase [EC:1.2.1.38 2.7.2.8]

GAT; glycerol-3-phosphate O-acyltransferase / dihydroxyacetone phosphate acyltransferase [EC:2.3.1.15 2.3.1.42]

AGPAT1_2; lysophosphatidate acyltransferase [EC:2.3.1.51]

LPT1; lysophospholipid acyltransferase [EC:2.3.1.51 2.3.1.23 2.3.1.-]

ARO1; pentafunctional AROM polypeptide [EC:4.2.3.4 4.2.1.10 1.1.1.25 2.7.1.71 2.5.1.19]

adhP; alcohol dehydrogenase, propanol-preferring [EC:1.1.1.1]

HIS4; phosphoribosyl-ATP pyrophosphohydrolase / phosphoribosyl-AMP cyclohydrolase / histidinol dehydrogenase [EC:3.6.1.31 3.5.4.19 1.1.1.23]

GOT1; aspartate aminotransferase, cytoplasmic [EC:2.6.1.1]

GOT2; aspartate aminotransferase, mitochondrial [EC:2.6.1.1]

TGL4; TAG lipase / steryl ester hydrolase / phospholipase A2 / LPA acyltransferase [EC:3.1.1.3 3.1.1.13 3.1.1.4 2.3.1.51]

LPIN; phosphatidate phosphatase LPIN [EC:3.1.3.4]

GLYK; D-glycerate 3-kinase [EC:2.7.1.31]

TPS; trehalose 6-phosphate synthase/phosphatase [EC:2.4.1.15 3.1.3.12]

CHO2; phosphatidylethanolamine N-methyltransferase [EC:2.1.1.17]

MET17; O-acetylhomoserine/O-acetylserine sulfhydrylase [EC:2.5.1.49 2.5.1.47]

CHO1; CDP-diacylglycerol---serine O-phosphatidyltransferase [EC:2.7.8.8]

ACO2; homoaconitase [EC:4.2.1.-]

PMM; phosphomannomutase [EC:5.4.2.8]

SDS; L-serine/L-threonine ammonia-lyase [EC:4.3.1.17 4.3.1.19]

AK6; adenylate kinase [EC:2.7.4.3]

DPP1; diacylglycerol diphosphate phosphatase / phosphatidate phosphatase [EC:3.1.3.81 3.1.3.4]

NUS1; dehydrodolichyl diphosphate syntase complex subunit NUS1 [EC:2.5.1.87]

IAL; isopenicillin-N N-acyltransferase like protein

HPT1; hypoxanthine phosphoribosyltransferase [EC:2.4.2.8]

TSL1; trehalose 6-phosphate synthase complex regulatory subunit

LOA1; 1-acylglycerol-3-phosphate O-acyltransferase [EC:2.3.1.51]

3. Microbial metabolism in diverse environments

AKR1A1; alcohol dehydrogenase (NADP+) [EC:1.1.1.2]

hom; homoserine dehydrogenase [EC:1.1.1.3]

gyaR; glyoxylate reductase [EC:1.1.1.26]

MDH2; malate dehydrogenase [EC:1.1.1.37]

IDH3; isocitrate dehydrogenase (NAD+) [EC:1.1.1.41]

IDH1; isocitrate dehydrogenase [EC:1.1.1.42]

PGD; 6-phosphogluconate dehydrogenase [EC:1.1.1.44 1.1.1.343]

G6PD; glucose-6-phosphate 1-dehydrogenase [EC:1.1.1.49 1.1.1.363]

serA; D-3-phosphoglycerate dehydrogenase / 2-oxoglutarate reductase [EC:1.1.1.95 1.1.1.399]

frmA; S-(hydroxymethyl)glutathione dehydrogenase / alcohol dehydrogenase [EC:1.1.1.284 1.1.1.1]

FDH; formate dehydrogenase [EC:1.17.1.9]

ALDH; aldehyde dehydrogenase (NAD+) [EC:1.2.1.3]

E1.2.1.5; aldehyde dehydrogenase (NAD(P)+) [EC:1.2.1.5]

asd; aspartate-semialdehyde dehydrogenase [EC:1.2.1.11]

GAPDH; glyceraldehyde 3-phosphate dehydrogenase [EC:1.2.1.12]

gabD; succinate-semialdehyde dehydrogenase / glutarate-semialdehyde dehydrogenase [EC:1.2.1.16 1.2.1.79 1.2.1.20]

PDHA; pyruvate dehydrogenase E1 component alpha subunit [EC:1.2.4.1]

PDHB; pyruvate dehydrogenase E1 component beta subunit [EC:1.2.4.1]

OGDH; 2-oxoglutarate dehydrogenase E1 component [EC:1.2.4.2]

SDHA; succinate dehydrogenase (ubiquinone) flavoprotein subunit [EC:1.3.5.1]

SDHB; succinate dehydrogenase (ubiquinone) iron-sulfur subunit [EC:1.3.5.1]

SDHD; succinate dehydrogenase (ubiquinone) membrane anchor subunit

E1.4.1.4; glutamate dehydrogenase (NADP+) [EC:1.4.1.4]

pdxH; pyridoxamine 5'-phosphate oxidase [EC:1.4.3.5]

MTHFD; methylenetetrahydrofolate dehydrogenase (NADP+) / methenyltetrahydrofolate cyclohydrolase / formyltetrahydrofolate synthetase [EC:1.5.1.5 3.5.4.9 6.3.4.3]

metF; methylenetetrahydrofolate reductase (NADPH) [EC:1.5.1.20]

uaZ; urate oxidase [EC:1.7.3.3]

cysJ; sulfite reductase (NADPH) flavoprotein alpha-component [EC:1.8.1.2]

cysI; sulfite reductase (NADPH) hemoprotein beta-component [EC:1.8.1.2]

DLD; dihydrolipoamide dehydrogenase [EC:1.8.1.4]

cysH; phosphoadenosine phosphosulfate reductase [EC:1.8.4.8 1.8.4.10]

E1.14.13.1; salicylate hydroxylase [EC:1.14.13.1]

MET1; uroporphyrin-III C-methyltransferase [EC:2.1.1.107]

glyA; glycine hydroxymethyltransferase [EC:2.1.2.1]

E2.2.1.1; transketolase [EC:2.2.1.1]

E2.2.1.2; transaldolase [EC:2.2.1.2]

ACAT; acetyl-CoA C-acetyltransferase [EC:2.3.1.9]

DLAT; pyruvate dehydrogenase E2 component (dihydrolipoamide acetyltransferase) [EC:2.3.1.12]

DLST; 2-oxoglutarate dehydrogenase E2 component (dihydrolipoamide succinyltransferase) [EC:2.3.1.61]

GPT; alanine transaminase [EC:2.6.1.2]

AGXT; alanine-glyoxylate transaminase / serine-glyoxylate transaminase / serine-pyruvate transaminase [EC:2.6.1.44 2.6.1.45 2.6.1.51]

serC; phosphoserine aminotransferase [EC:2.6.1.52]

HK; hexokinase [EC:2.7.1.1]

pfkA; 6-phosphofructokinase 1 [EC:2.7.1.11]

E2.7.1.12; gluconokinase [EC:2.7.1.12]

cysC; adenylylsulfate kinase [EC:2.7.1.25]

DAK; triose/dihydroxyacetone kinase / FAD-AMP lyase (cyclizing) [EC:2.7.1.28 2.7.1.29 4.6.1.15]

thrB; homoserine kinase [EC:2.7.1.39]

PK; pyruvate kinase [EC:2.7.1.40]

PGK; phosphoglycerate kinase [EC:2.7.2.3]

lysC; aspartate kinase [EC:2.7.2.4]

PRPS; ribose-phosphate pyrophosphokinase [EC:2.7.6.1]

sat; sulfate adenylyltransferase [EC:2.7.7.4]

APA1_2; sulfate adenylyltransferase (ADP) / ATP adenylyltransferase [EC:2.7.7.5 2.7.7.53]

PGLS; 6-phosphogluconolactonase [EC:3.1.1.31]

E3.1.1.45; carboxymethylenebutenolidase [EC:3.1.1.45]

frmB; S-formylglutathione hydrolase [EC:3.1.2.12]

serB; phosphoserine phosphatase [EC:3.1.3.3]

cysQ; 3'(2'), 5'-bisphosphate nucleotidase [EC:3.1.3.7]

E3.1.3.41; 4-nitrophenyl phosphatase [EC:3.1.3.41]

E3.5.1.4; amidase [EC:3.5.1.4]

allB; allantoinase [EC:3.5.2.5]

alc; allantoicase [EC:3.5.3.4]

E3.5.5.1; nitrilase [EC:3.5.5.1]

FAHD1; acylpyruvate hydrolase [EC:3.7.1.5]

E4.1.1.15; glutamate decarboxylase [EC:4.1.1.15]

E4.1.1.49; phosphoenolpyruvate carboxykinase (ATP) [EC:4.1.1.49]

ltaE; threonine aldolase [EC:4.1.2.48]

FBA; fructose-bisphosphate aldolase, class II [EC:4.1.2.13]

E4.1.3.1; isocitrate lyase [EC:4.1.3.1]

aceB; malate synthase [EC:2.3.3.9]

CS; citrate synthase [EC:2.3.3.1]

LYS21; homocitrate synthase [EC:2.3.3.14]

E4.2.1.2B; fumarate hydratase, class II [EC:4.2.1.2]

ACO; aconitate hydratase [EC:4.2.1.3]

ENO; enolase [EC:4.2.1.11]

hemB; porphobilinogen synthase [EC:4.2.1.24]

LYS4; homoaconitate hydratase [EC:4.2.1.36]

dapA; 4-hydroxy-tetrahydrodipicolinate synthase [EC:4.3.3.7]

hemD; uroporphyrinogen-III synthase [EC:4.2.1.75]

thrC; threonine synthase [EC:4.2.3.1]

cysK; cysteine synthase [EC:2.5.1.47]

hemC; hydroxymethylbilane synthase [EC:2.5.1.61]

rpe; ribulose-phosphate 3-epimerase [EC:5.1.3.1]

galM; aldose 1-epimerase [EC:5.1.3.3]

E5.1.3.15; glucose-6-phosphate 1-epimerase [EC:5.1.3.15]

TPI; triosephosphate isomerase (TIM) [EC:5.3.1.1]

rpiA; ribose 5-phosphate isomerase A [EC:5.3.1.6]

GPI; glucose-6-phosphate isomerase [EC:5.3.1.9]

PGAM; 2,3-bisphosphoglycerate-dependent phosphoglycerate mutase [EC:5.4.2.11]
pgm; phosphoglucomutase [EC:5.4.2.2]
EARS; glutamyl-tRNA synthetase [EC:6.1.1.17]
ACSS1_2; acetyl-CoA synthetase [EC:6.2.1.1]
LSC1; succinyl-CoA synthetase alpha subunit [EC:6.2.1.4 6.2.1.5]
LSC2; succinyl-CoA synthetase beta subunit [EC:6.2.1.4 6.2.1.5]
PC; pyruvate carboxylase [EC:6.4.1.1]
FBP; fructose-1,6-bisphosphatase I [EC:3.1.3.11]
E1.1.1.65; pyridoxine 4-dehydrogenase [EC:1.1.1.65]
LYS12; homoisocitrate dehydrogenase [EC:1.1.1.87]
DDI2_3; cyanamide hydratase [EC:4.2.1.69]
uraH; 5-hydroxyisourate hydrolase [EC:3.5.2.17]
PHAA; phenylacetate 2-hydroxylase [EC:1.14.14.54]
ACACA; acetyl-CoA carboxylase / biotin carboxylase 1 [EC:6.4.1.2 6.3.4.14 2.1.3.15]
HAO; (S)-2-hydroxy-acid oxidase [EC:1.1.3.15]
ABAT; 4-aminobutyrate aminotransferase / (S)-3-amino-2-methylpropionate transaminase [EC:2.6.1.19 2.6.1.22]
adhP; alcohol dehydrogenase, propanol-preferring [EC:1.1.1.1]
GOT1; aspartate aminotransferase, cytoplasmic [EC:2.6.1.1]
GOT2; aspartate aminotransferase, mitochondrial [EC:2.6.1.1]
GDH2; glutamate dehydrogenase [EC:1.4.1.2]
MET17; O-acetylhomoserine/O-acetylserine sulfhydrylase [EC:2.5.1.49 2.5.1.47]
DAS; dihydroxyacetone synthase [EC:2.2.1.3]
ACO2; homoaconitase [EC:4.2.1.-]
LRA4; 2-keto-3-deoxy-L-rhamnonate aldolase [EC:4.1.2.53]
GLX3; D-lactate dehydratase [EC:4.2.1.130]

4. Huntington disease

SDHA; succinate dehydrogenase (ubiquinone) flavoprotein subunit [EC:1.3.5.1]
SDHB; succinate dehydrogenase (ubiquinone) iron-sulfur subunit [EC:1.3.5.1]
SDHD; succinate dehydrogenase (ubiquinone) membrane anchor subunit

UQCRFS1; ubiquinol-cytochrome c reductase iron-sulfur subunit [EC:7.1.1.8]

CYC1; ubiquinol-cytochrome c reductase cytochrome c1 subunit

QCR1; ubiquinol-cytochrome c reductase core subunit 1

QCR2; ubiquinol-cytochrome c reductase core subunit 2

QCR7; ubiquinol-cytochrome c reductase subunit 7

QCR8; ubiquinol-cytochrome c reductase subunit 8

PIK3C3; phosphatidylinositol 3-kinase [EC:2.7.1.137]

ATPeF0B; F-type H+-transporting ATPase subunit b

ATPeF1A; F-type H+-transporting ATPase subunit alpha

ATPeF1B; F-type H+-transporting ATPase subunit beta [EC:7.1.2.2]

ATPeF1D; F-type H+-transporting ATPase subunit delta

ATPeF1G; F-type H+-transporting ATPase subunit gamma

ATPeF0O; F-type H+-transporting ATPase subunit O

ATPeF0D; F-type H+-transporting ATPase subunit d

COX5B; cytochrome c oxidase subunit 5b

COX6A; cytochrome c oxidase subunit 6a

COX6B; cytochrome c oxidase subunit 6b

COX7C; cytochrome c oxidase subunit 7c

PSMA1; 20S proteasome subunit alpha 6 [EC:3.4.25.1]

PSMA3; 20S proteasome subunit alpha 7 [EC:3.4.25.1]

PSMA4; 20S proteasome subunit alpha 3 [EC:3.4.25.1]

PSMA5; 20S proteasome subunit alpha 5 [EC:3.4.25.1]

PSMA6; 20S proteasome subunit alpha 1 [EC:3.4.25.1]

PSMA7; 20S proteasome subunit alpha 4 [EC:3.4.25.1]

PSMB1; 20S proteasome subunit beta 6 [EC:3.4.25.1]

PSMB2; 20S proteasome subunit beta 4 [EC:3.4.25.1]

PSMB3; 20S proteasome subunit beta 3 [EC:3.4.25.1]

PSMB4; 20S proteasome subunit beta 7 [EC:3.4.25.1]

PSMB5; 20S proteasome subunit beta 5 [EC:3.4.25.1]

PSMB6; 20S proteasome subunit beta 1 [EC:3.4.25.1]

PSMB7; 20S proteasome subunit beta 2 [EC:3.4.25.1]

RPB1; DNA-directed RNA polymerase II subunit RPB1 [EC:2.7.7.6]

RPB10; DNA-directed RNA polymerases I, II, and III subunit RPABC5

RPB2; DNA-directed RNA polymerase II subunit RPB2 [EC:2.7.7.6]

RPB3; DNA-directed RNA polymerase II subunit RPB3

RPB5; DNA-directed RNA polymerases I, II, and III subunit RPABC1

RPB6; DNA-directed RNA polymerases I, II, and III subunit RPABC2

RPB7; DNA-directed RNA polymerase II subunit RPB7

RPB8; DNA-directed RNA polymerases I, II, and III subunit RPABC3

PSMD2; 26S proteasome regulatory subunit N1

PSMD4; 26S proteasome regulatory subunit N10

PSMD14; 26S proteasome regulatory subunit N11

PSMD8; 26S proteasome regulatory subunit N12

PSMD1; 26S proteasome regulatory subunit N2

PSMD3; 26S proteasome regulatory subunit N3

PSMD12; 26S proteasome regulatory subunit N5

PSMD6; 26S proteasome regulatory subunit N7

PSMD7; 26S proteasome regulatory subunit N8

PSMD13; 26S proteasome regulatory subunit N9

PSMC2; 26S proteasome regulatory subunit T1

PSMC1; 26S proteasome regulatory subunit T2

PSMC4; 26S proteasome regulatory subunit T3

PSMC6; 26S proteasome regulatory subunit T4

PSMC3; 26S proteasome regulatory subunit T5

PSMC5; 26S proteasome regulatory subunit T6

TBP; transcription initiation factor TFIID TATA-box-binding protein

NDUFS1; NADH dehydrogenase (ubiquinone) Fe-S protein 1 [EC:7.1.1.2 1.6.99.3]

NDUFS2; NADH dehydrogenase (ubiquinone) Fe-S protein 2 [EC:7.1.1.2 1.6.99.3]

NDUFS3; NADH dehydrogenase (ubiquinone) Fe-S protein 3 [EC:7.1.1.2 1.6.99.3]

NDUFS4; NADH dehydrogenase (ubiquinone) Fe-S protein 4

NDUFS6; NADH dehydrogenase (ubiquinone) Fe-S protein 6

NDUFS7; NADH dehydrogenase (ubiquinone) Fe-S protein 7 [EC:7.1.1.2 1.6.99.3]

NDUFS8; NADH dehydrogenase (ubiquinone) Fe-S protein 8 [EC:7.1.1.2 1.6.99.3]

NDUFV1; NADH dehydrogenase (ubiquinone) flavoprotein 1 [EC:7.1.1.2 1.6.99.3]

NDUFV2; NADH dehydrogenase (ubiquinone) flavoprotein 2 [EC:7.1.1.2 1.6.99.3]

NDUFA2; NADH dehydrogenase (ubiquinone) 1 alpha subcomplex subunit 2

NDUFA5; NADH dehydrogenase (ubiquinone) 1 alpha subcomplex subunit 5

NDUFA6; NADH dehydrogenase (ubiquinone) 1 alpha subcomplex subunit 6

NDUFA8; NADH dehydrogenase (ubiquinone) 1 alpha subcomplex subunit 8

NDUFA9; NADH dehydrogenase (ubiquinone) 1 alpha subcomplex subunit 9

NDUFAB1; NADH dehydrogenase (ubiquinone) 1 alpha/beta subcomplex 1, acyl-carrier protein

NDUFB8; NADH dehydrogenase (ubiquinone) 1 beta subcomplex subunit 8

NDUFB9; NADH dehydrogenase (ubiquinone) 1 beta subcomplex subunit 9

SOD2; superoxide dismutase, Fe-Mn family [EC:1.15.1.1]

SOD1; superoxide dismutase, Cu-Zn family [EC:1.15.1.1]

CLTC; clathrin heavy chain

DCTN1; dynactin 1

SLC25A4S; solute carrier family 25 (mitochondrial adenine nucleotide translocator), member 4/5/6/31

HDAC1_2; histone deacetylase 1/2 [EC:3.5.1.98]

RPN13; 26S proteasome regulatory subunit N13

PSMD9; 26S proteasome regulatory subunit N4

MTOR; serine/threonine-protein kinase mTOR [EC:2.7.11.1]

ULK2; serine/threonine-protein kinase ULK2 [EC:2.7.11.1]

ATG13; autophagy-related protein 13

PIK3R4; phosphoinositide-3-kinase, regulatory subunit 4 [EC:2.7.11.1]

BECN; beclin

CYC; cytochrome c

ERN1; serine/threonine-protein kinase/endoribonuclease IRE1 [EC:2.7.11.1 3.1.26.-]

KIF5; kinesin family member 5

SHFM1; 26 proteasome complex subunit DSS1

NDUFA12; NADH dehydrogenase (ubiquinone) 1 alpha subcomplex subunit 12

SIN3A; paired amphipathic helix protein Sin3a

AP2A; AP-2 complex subunit alpha

AP2B1; AP-2 complex subunit beta-1

AP2M1; AP-2 complex subunit mu-1
AP2S1; AP-2 complex subunit sigma-1
VDAC2; voltage-dependent anion channel protein 2
ACTR1; centractin
ATG2; autophagy-related protein 2
WIPI1_2; autophagy-related protein 18

5. Alzheimer disease

APDH; glyceraldehyde 3-phosphate dehydrogenase [EC:1.2.1.12]
DHA; succinate dehydrogenase (ubiquinone) flavoprotein subunit [EC:1.3.5.1]
DHB; succinate dehydrogenase (ubiquinone) iron-sulfur subunit [EC:1.3.5.1]
DHD; succinate dehydrogenase (ubiquinone) membrane anchor subunit
QCRFS1; ubiquinol-cytochrome c reductase iron-sulfur subunit [EC:7.1.1.8]
YC1; ubiquinol-cytochrome c reductase cytochrome c1 subunit
CR1; ubiquinol-cytochrome c reductase core subunit 1
CR2; ubiquinol-cytochrome c reductase core subunit 2
CR7; ubiquinol-cytochrome c reductase subunit 7
CR8; ubiquinol-cytochrome c reductase subunit 8
IK3C3; phosphatidylinositol 3-kinase [EC:2.7.1.137]
DE; insulysin [EC:3.4.24.56]
TPeF0B; F-type H+-transporting ATPase subunit b
TPeF1A; F-type H+-transporting ATPase subunit alpha
TPeF1B; F-type H+-transporting ATPase subunit beta [EC:7.1.2.2]
TPeF1D; F-type H+-transporting ATPase subunit delta
TPeF1G; F-type H+-transporting ATPase subunit gamma
TPeF0O; F-type H+-transporting ATPase subunit O
TPeF0D; F-type H+-transporting ATPase subunit d
ALM; calmodulin
OX5B; cytochrome c oxidase subunit 5b
OX6A; cytochrome c oxidase subunit 6a
OX6B; cytochrome c oxidase subunit 6b

OX7C; cytochrome c oxidase subunit 7c

SMA1; 20S proteasome subunit alpha 6 [EC:3.4.25.1]

SMA3; 20S proteasome subunit alpha 7 [EC:3.4.25.1]

SMA4; 20S proteasome subunit alpha 3 [EC:3.4.25.1]

SMA5; 20S proteasome subunit alpha 5 [EC:3.4.25.1]

SMA6; 20S proteasome subunit alpha 1 [EC:3.4.25.1]

SMA7; 20S proteasome subunit alpha 4 [EC:3.4.25.1]

SMB1; 20S proteasome subunit beta 6 [EC:3.4.25.1]

SMB2; 20S proteasome subunit beta 4 [EC:3.4.25.1]

SMB3; 20S proteasome subunit beta 3 [EC:3.4.25.1]

SMB4; 20S proteasome subunit beta 7 [EC:3.4.25.1]

SMB5; 20S proteasome subunit beta 5 [EC:3.4.25.1]

SMB6; 20S proteasome subunit beta 1 [EC:3.4.25.1]

SMB7; 20S proteasome subunit beta 2 [EC:3.4.25.1]

SMD2; 26S proteasome regulatory subunit N1

SMD4; 26S proteasome regulatory subunit N10

SMD14; 26S proteasome regulatory subunit N11

SMD8; 26S proteasome regulatory subunit N12

SMD1; 26S proteasome regulatory subunit N2

SMD3; 26S proteasome regulatory subunit N3

SMD12; 26S proteasome regulatory subunit N5

SMD6; 26S proteasome regulatory subunit N7

SMD7; 26S proteasome regulatory subunit N8

SMD13; 26S proteasome regulatory subunit N9

SMC2; 26S proteasome regulatory subunit T1

SMC1; 26S proteasome regulatory subunit T2

SMC4; 26S proteasome regulatory subunit T3

SMC6; 26S proteasome regulatory subunit T4

SMC3; 26S proteasome regulatory subunit T5

SMC5; 26S proteasome regulatory subunit T6

SK3B; glycogen synthase kinase 3 beta [EC:2.7.11.26]

SNK2A; casein kinase II subunit alpha [EC:2.7.11.1]

SNK2B; casein kinase II subunit beta

IF2S1; translation initiation factor 2 subunit 1

DUFS1; NADH dehydrogenase (ubiquinone) Fe-S protein 1 [EC:7.1.1.2 1.6.99.3]

DUFS2; NADH dehydrogenase (ubiquinone) Fe-S protein 2 [EC:7.1.1.2 1.6.99.3]

DUFS3; NADH dehydrogenase (ubiquinone) Fe-S protein 3 [EC:7.1.1.2 1.6.99.3]

DUFS4; NADH dehydrogenase (ubiquinone) Fe-S protein 4

DUFS6; NADH dehydrogenase (ubiquinone) Fe-S protein 6

DUFS7; NADH dehydrogenase (ubiquinone) Fe-S protein 7 [EC:7.1.1.2 1.6.99.3]

DUFS8; NADH dehydrogenase (ubiquinone) Fe-S protein 8 [EC:7.1.1.2 1.6.99.3]

DUFV1; NADH dehydrogenase (ubiquinone) flavoprotein 1 [EC:7.1.1.2 1.6.99.3]

DUFV2; NADH dehydrogenase (ubiquinone) flavoprotein 2 [EC:7.1.1.2 1.6.99.3]

DUFA2; NADH dehydrogenase (ubiquinone) 1 alpha subcomplex subunit 2

DUFA5; NADH dehydrogenase (ubiquinone) 1 alpha subcomplex subunit 5

DUFA6; NADH dehydrogenase (ubiquinone) 1 alpha subcomplex subunit 6

DUFA8; NADH dehydrogenase (ubiquinone) 1 alpha subcomplex subunit 8

DUFA9; NADH dehydrogenase (ubiquinone) 1 alpha subcomplex subunit 9

DUFAB1; NADH dehydrogenase (ubiquinone) 1 alpha/beta subcomplex 1, acyl-carrier protein

DUFB8; NADH dehydrogenase (ubiquinone) 1 beta subcomplex subunit 8

DUFB9; NADH dehydrogenase (ubiquinone) 1 beta subcomplex subunit 9

PP3C; serine/threonine-protein phosphatase 2B catalytic subunit [EC:3.1.3.16]

RK; mitogen-activated protein kinase 1/3 [EC:2.7.11.24]

AE1; amyloid beta precursor protein binding protein 1

LC25A4S; solute carrier family 25 (mitochondrial adenine nucleotide translocator), member 4/5/6/31

PID; peptidyl-prolyl isomerase D [EC:5.2.1.8]

PP3R; serine/threonine-protein phosphatase 2B regulatory subunit

PN13; 26S proteasome regulatory subunit N13

SMD9; 26S proteasome regulatory subunit N4

TOR; serine/threonine-protein kinase mTOR [EC:2.7.11.1]

RAS; GTPase KRas

LK2; serine/threonine-protein kinase ULK2 [EC:2.7.11.1]

TG13; autophagy-related protein 13

IK3R4; phosphoinositide-3-kinase, regulatory subunit 4 [EC:2.7.11.1]

ECN; beclin

YC; cytochrome c

RN1; serine/threonine-protein kinase/endoribonuclease IRE1 [EC:2.7.11.1 3.1.26.-]

IF5; kinesin family member 5

HFM1; 26 proteasome complex subunit DSS1

DUFA12; NADH dehydrogenase (ubiquinone) 1 alpha subcomplex subunit 12

DAC2; voltage-dependent anion channel protein 2

TG2; autophagy-related protein 2

IPI1_2; autophagy-related protein 18

6. Biosynthesis of amino acids

hom; homoserine dehydrogenase [EC:1.1.1.3]

IDH3; isocitrate dehydrogenase (NAD+) [EC:1.1.1.41]

IDH1; isocitrate dehydrogenase [EC:1.1.1.42]

leuB; 3-isopropylmalate dehydrogenase [EC:1.1.1.85]

ilvC; ketol-acid reductoisomerase [EC:1.1.1.86]

serA; D-3-phosphoglycerate dehydrogenase / 2-oxoglutarate reductase [EC:1.1.1.95 1.1.1.399]

asd; aspartate-semialdehyde dehydrogenase [EC:1.2.1.11]

GAPDH; glyceraldehyde 3-phosphate dehydrogenase [EC:1.2.1.12]

LYS2; L-2-aminoadipate reductase [EC:1.2.1.95]

proA; glutamate-5-semialdehyde dehydrogenase [EC:1.2.1.41]

proC; pyrroline-5-carboxylate reductase [EC:1.5.1.2]

LYS1; saccharopine dehydrogenase (NAD+, L-lysine forming) [EC:1.5.1.7]

LYS9; saccharopine dehydrogenase (NADP+, L-glutamate forming) [EC:1.5.1.10]

metE; 5-methyltetrahydropteroyltriglutamate--homocysteine methyltransferase [EC:2.1.1.14]

glyA; glycine hydroxymethyltransferase [EC:2.1.2.1]

OTC; ornithine carbamoyltransferase [EC:2.1.3.3]

E2.2.1.1; transketolase [EC:2.2.1.1]

E2.2.1.2; transaldolase [EC:2.2.1.2]

ARG2; amino-acid N-acetyltransferase [EC:2.3.1.1]

argJ; glutamate N-acetyltransferase / amino-acid N-acetyltransferase [EC:2.3.1.35 2.3.1.1]

metX; homoserine O-acetyltransferase/O-succinyltransferase [EC:2.3.1.31 2.3.1.46]

trpD; anthranilate phosphoribosyltransferase [EC:2.4.2.18]

metK; S-adenosylmethionine synthetase [EC:2.5.1.6]

GPT; alanine transaminase [EC:2.6.1.2]

hisC; histidinol-phosphate aminotransferase [EC:2.6.1.9]

E2.6.1.11; acetylornithine aminotransferase [EC:2.6.1.11]

E2.6.1.42; branched-chain amino acid aminotransferase [EC:2.6.1.42]

serC; phosphoserine aminotransferase [EC:2.6.1.52]

ARO8; aromatic amino acid aminotransferase I / 2-aminoadipate transaminase [EC:2.6.1.57 2.6.1.39 2.6.1.27 2.6.1.5]

pfkA; 6-phosphofructokinase 1 [EC:2.7.1.11]

thrB; homoserine kinase [EC:2.7.1.39]

PK; pyruvate kinase [EC:2.7.1.40]

PGK; phosphoglycerate kinase [EC:2.7.2.3]

lysC; aspartate kinase [EC:2.7.2.4]

proB; glutamate 5-kinase [EC:2.7.2.11]

PRPS; ribose-phosphate pyrophosphokinase [EC:2.7.6.1]

serB; phosphoserine phosphatase [EC:3.1.3.3]

argE; acetylornithine deacetylase [EC:3.5.1.16]

E3.5.3.1; arginase [EC:3.5.3.1]

ltaE; threonine aldolase [EC:4.1.2.48]

FBA; fructose-bisphosphate aldolase, class II [EC:4.1.2.13]

E2.5.1.54; 3-deoxy-7-phosphoheptulonate synthase [EC:2.5.1.54]

CS; citrate synthase [EC:2.3.3.1]

leuA; 2-isopropylmalate synthase [EC:2.3.3.13]

E2.2.1.6L; acetolactate synthase I/II/III large subunit [EC:2.2.1.6]

E2.2.1.6S; acetolactate synthase I/III small subunit [EC:2.2.1.6]

LYS21; homocitrate synthase [EC:2.3.3.14]

TRP3; anthranilate synthase / indole-3-glycerol phosphate synthase [EC:4.1.3.27 4.1.1.48]

trpE; anthranilate synthase component I [EC:4.1.3.27]

HIS7; imidazole glycerol-phosphate synthase [EC:4.3.2.10]

ACO; aconitate hydratase [EC:4.2.1.3]

ilvD; dihydroxy-acid dehydratase [EC:4.2.1.9]

ENO; enolase [EC:4.2.1.11]

hisB; imidazoleglycerol-phosphate dehydratase [EC:4.2.1.19]

TRP; tryptophan synthase [EC:4.2.1.20]

CBS; cystathionine beta-synthase [EC:4.2.1.22]

LEU1; 3-isopropylmalate dehydratase [EC:4.2.1.33]

LYS4; homoaconitate hydratase [EC:4.2.1.36]

dapA; 4-hydroxy-tetrahydrodipicolinate synthase [EC:4.3.3.7]

thrC; threonine synthase [EC:4.2.3.1]

aroC; chorismate synthase [EC:4.2.3.5]

cysK; cysteine synthase [EC:2.5.1.47]

metB; cystathionine gamma-synthase [EC:2.5.1.48]

E4.3.1.19; threonine dehydratase [EC:4.3.1.19]

argH; argininosuccinate lyase [EC:4.3.2.1]

CTH; cystathionine gamma-lyase [EC:4.4.1.1]

metC; cysteine-S-conjugate beta-lyase [EC:4.4.1.13]

rpe; ribulose-phosphate 3-epimerase [EC:5.1.3.1]

TPI; triosephosphate isomerase (TIM) [EC:5.3.1.1]

rpiA; ribose 5-phosphate isomerase A [EC:5.3.1.6]

hisA; phosphoribosylformimino-5-aminoimidazole carboxamide ribotide isomerase [EC:5.3.1.16]

trpF; phosphoribosylanthranilate isomerase [EC:5.3.1.24]

PGAM; 2,3-bisphosphoglycerate-dependent phosphoglycerate mutase [EC:5.4.2.11]

E5.4.99.5; chorismate mutase [EC:5.4.99.5]

argG; argininosuccinate synthase [EC:6.3.4.5]

asnB; asparagine synthase (glutamine-hydrolysing) [EC:6.3.5.4]

PC; pyruvate carboxylase [EC:6.4.1.1]

aroQ; 3-dehydroquinate dehydratase II [EC:4.2.1.10]

E3.1.3.15B; histidinol-phosphatase (PHP family) [EC:3.1.3.15]

LYS12; homoisocitrate dehydrogenase [EC:1.1.1.87]

ARG56; N-acetyl-gamma-glutamyl-phosphate reductase / acetylglutamate kinase [EC:1.2.1.38 2.7.2.8]

ARO1; pentafunctional AROM polypeptide [EC:4.2.3.4 4.2.1.10 1.1.1.25 2.7.1.71 2.5.1.19]

HIS4; phosphoribosyl-ATP pyrophosphohydrolase / phosphoribosyl-AMP cyclohydrolase / histidinol dehydrogenase [EC:3.6.1.31 3.5.4.19 1.1.1.23]

GOT1; aspartate aminotransferase, cytoplasmic [EC:2.6.1.1]

GOT2; aspartate aminotransferase, mitochondrial [EC:2.6.1.1]

MET17; O-acetylhomoserine/O-acetylserine sulfhydrylase [EC:2.5.1.49 2.5.1.47]

ACO2; homoaconitase [EC:4.2.1.-]

SDS; L-serine/L-threonine ammonia-lyase [EC:4.3.1.17 4.3.1.19]

7. Cell cycle – yeast

BUB1; checkpoint serine/threonine-protein kinase [EC:2.7.11.1]

BUB3; cell cycle arrest protein BUB3

MCM5; DNA replication licensing factor MCM5 [EC:3.6.4.12]

MCM7; DNA replication licensing factor MCM7 [EC:3.6.4.12]

MCM4; DNA replication licensing factor MCM4 [EC:3.6.4.12]

CDC6; cell division control protein 6

CDC7; cell division control protein 7 [EC:2.7.11.1]

CKS1; cyclin-dependent kinase regulatory subunit CKS1

DAM1; DASH complex subunit DAM1

DBF4; regulatory subunit for Cdc7p protein kinase

ESP1; separase [EC:3.4.22.49]

HSL1; serine/threonine-protein kinase HSL1, negative regulator of Swe1 kinase [EC:2.7.11.-]

PRMT5; type II protein arginine methyltransferase [EC:2.1.1.320]

MCM2; DNA replication licensing factor MCM2 [EC:3.6.4.12]

MCM3; DNA replication licensing factor MCM3 [EC:3.6.4.12]

MCM6; DNA replication licensing factor MCM6 [EC:3.6.4.12]

MEC1; cell cycle checkpoint protein MEC1

MEC3; G2-specific checkpoint protein

MIH1; M-phase inducer tyrosine phosphatase [EC:3.1.3.48]

ORC1; origin recognition complex subunit 1

ORC2; origin recognition complex subunit 2

ORC4; origin recognition complex subunit 4

ORC5; origin recognition complex subunit 5

ORC6; origin recognition complex subunit 6

PHO2; regulatory protein PHO2

HRAD1; cell cycle checkpoint protein [EC:3.1.11.2]

SKP1; S-phase kinase-associated protein 1

SWE1; mitosis inhibitor protein kinase SWE1 [EC:2.7.11.1]

CUL1; cullin 1

APC1; anaphase-promoting complex subunit 1

APC2; anaphase-promoting complex subunit 2

APC3; anaphase-promoting complex subunit 3

APC4; anaphase-promoting complex subunit 4

APC5; anaphase-promoting complex subunit 5

APC6; anaphase-promoting complex subunit 6

APC8; anaphase-promoting complex subunit 8

APC10; anaphase-promoting complex subunit 10

APC11; anaphase-promoting complex subunit 11

GRR1; F-box and leucine-rich repeat protein GRR1

CDC4; F-box and WD-40 domain protein CDC4

CDC20; cell division cycle 20, cofactor of APC complex

CDH1; cell division cycle 20-like protein 1, cofactor of APC complex

PPP2R1; serine/threonine-protein phosphatase 2A regulatory subunit A

PPP2R2; serine/threonine-protein phosphatase 2A regulatory subunit B

ERK; mitogen-activated protein kinase 1/3 [EC:2.7.11.24]

CDC45; cell division control protein 45

SMC1; structural maintenance of chromosome 1

CDC14; cell division cycle 14 [EC:3.1.3.16 3.1.3.48]

CLN3; G1/S-specific cyclin CLN3

MBP1; transcription factor MBP1

SWI6; regulatory protein SWI6

SWI4; regulatory protein SWI4

FAR1; cyclin-dependent kinase inhibitor FAR1

PHO81; CDK inhibitor PHO81

PHO85; negative regulator of the PHO system [EC:2.7.11.22]

PCL1; PHO85 cyclin-1

PCL2_9; PHO85 cyclin-2/9

CLB3_4; G2/mitotic-specific cyclin 3/4

CDC5; cell cycle serine/threonine-protein kinase CDC5/MSD2 [EC:2.7.11.21]

RAD9; DNA repair protein RAD9

DDC1; DNA damage checkpoint protein

SSN6; general transcriptional corepressor CYC8

TUP1; general transcriptional corepressor TUP1

CAK1; serine/threonine-protein kinase CAK1 [EC:2.7.11.22]

GIN4; serine/threonine-protein kinase GIN4 [EC:2.7.11.-]

SMC3; structural maintenance of chromosome 3 (chondroitin sulfate proteoglycan 6)

SCC1; cohesin complex subunit SCC1

STAG1_2; cohesin complex subunit SA-1/2

SCC2; cohesin loading factor subunit SCC2

SMC2; structural maintenance of chromosome 2

BRRN1; condensin complex subunit 2

YCS4; condensin complex subunit 1

YCG1; condensin complex subunit 3

MAD1; mitotic spindle assembly checkpoint protein MAD1

LTE1; Gdp/GTP exchange factor required for growth at low temperatures

TEM1; Gtp-binding protein of the ras superfamily involved in termination of M-phase

CDC15; cell division control protein CDC15 [EC:2.7.11.1]

DBF2; cell cycle protein kinase DBF2 [EC:2.7.11.-]

MOB1; MOB kinase activator 1

TTK; serine/threonine-protein kinase TTK/MPS1 [EC:2.7.12.1]

RFXX; regulatory factor X, other

MET30; F-box and WD-40 domain protein MET30

MRC1; mediator of replication checkpoint protein 1

YOX1; homeobox protein YOX1/YHP1

MCM1; pheromone receptor transcription factor

LCD1; DNA damage checkpoint protein LCD1

CLB2; G2/mitotic-specific cyclin 2

8. Parkinson disease

SDHA; succinate dehydrogenase (ubiquinone) flavoprotein subunit [EC:1.3.5.1]

SDHB; succinate dehydrogenase (ubiquinone) iron-sulfur subunit [EC:1.3.5.1]

SDHD; succinate dehydrogenase (ubiquinone) membrane anchor subunit

UQCRFS1; ubiquinol-cytochrome c reductase iron-sulfur subunit [EC:7.1.1.8]

CYC1; ubiquinol-cytochrome c reductase cytochrome c1 subunit

QCR1; ubiquinol-cytochrome c reductase core subunit 1

QCR2; ubiquinol-cytochrome c reductase core subunit 2

QCR7; ubiquinol-cytochrome c reductase subunit 7

QCR8; ubiquinol-cytochrome c reductase subunit 8

ATPeF0B; F-type H+-transporting ATPase subunit b

ATPeF1A; F-type H+-transporting ATPase subunit alpha

ATPeF1B; F-type H+-transporting ATPase subunit beta [EC:7.1.2.2]

ATPeF1D; F-type H+-transporting ATPase subunit delta

ATPeF1G; F-type H+-transporting ATPase subunit gamma

ATPeF0O; F-type H+-transporting ATPase subunit O

ATPeF0D; F-type H+-transporting ATPase subunit d

CALM; calmodulin

COX5B; cytochrome c oxidase subunit 5b

COX6A; cytochrome c oxidase subunit 6a

COX6B; cytochrome c oxidase subunit 6b

COX7C; cytochrome c oxidase subunit 7c

PSMA1; 20S proteasome subunit alpha 6 [EC:3.4.25.1]

PSMA3; 20S proteasome subunit alpha 7 [EC:3.4.25.1]

PSMA4; 20S proteasome subunit alpha 3 [EC:3.4.25.1]

PSMA5; 20S proteasome subunit alpha 5 [EC:3.4.25.1]

PSMA6; 20S proteasome subunit alpha 1 [EC:3.4.25.1]

PSMA7; 20S proteasome subunit alpha 4 [EC:3.4.25.1]

PSMB1; 20S proteasome subunit beta 6 [EC:3.4.25.1]

PSMB2; 20S proteasome subunit beta 4 [EC:3.4.25.1]

PSMB3; 20S proteasome subunit beta 3 [EC:3.4.25.1]

PSMB4; 20S proteasome subunit beta 7 [EC:3.4.25.1]

PSMB5; 20S proteasome subunit beta 5 [EC:3.4.25.1]

PSMB6; 20S proteasome subunit beta 1 [EC:3.4.25.1]

PSMB7; 20S proteasome subunit beta 2 [EC:3.4.25.1]

RP-S27Ae; ubiquitin-small subunit ribosomal protein S27Ae

PSMD2; 26S proteasome regulatory subunit N1

PSMD4; 26S proteasome regulatory subunit N10

PSMD14; 26S proteasome regulatory subunit N11

PSMD8; 26S proteasome regulatory subunit N12

PSMD1; 26S proteasome regulatory subunit N2

PSMD3; 26S proteasome regulatory subunit N3

PSMD12; 26S proteasome regulatory subunit N5

PSMD6; 26S proteasome regulatory subunit N7

PSMD7; 26S proteasome regulatory subunit N8

PSMD13; 26S proteasome regulatory subunit N9

PSMC2; 26S proteasome regulatory subunit T1

PSMC1; 26S proteasome regulatory subunit T2

PSMC4; 26S proteasome regulatory subunit T3

PSMC6; 26S proteasome regulatory subunit T4

PSMC3; 26S proteasome regulatory subunit T5

PSMC5; 26S proteasome regulatory subunit T6

UBE1; ubiquitin-activating enzyme E1 [EC:6.2.1.45]

EIF2S1; translation initiation factor 2 subunit 1

trxA; thioredoxin 1

NDUFS1; NADH dehydrogenase (ubiquinone) Fe-S protein 1 [EC:7.1.1.2 1.6.99.3]

NDUFS2; NADH dehydrogenase (ubiquinone) Fe-S protein 2 [EC:7.1.1.2 1.6.99.3]

NDUFS3; NADH dehydrogenase (ubiquinone) Fe-S protein 3 [EC:7.1.1.2 1.6.99.3]

NDUFS4; NADH dehydrogenase (ubiquinone) Fe-S protein 4

NDUFS6; NADH dehydrogenase (ubiquinone) Fe-S protein 6

NDUFS7; NADH dehydrogenase (ubiquinone) Fe-S protein 7 [EC:7.1.1.2 1.6.99.3]

NDUFS8; NADH dehydrogenase (ubiquinone) Fe-S protein 8 [EC:7.1.1.2 1.6.99.3]

NDUFV1; NADH dehydrogenase (ubiquinone) flavoprotein 1 [EC:7.1.1.2 1.6.99.3]

NDUFV2; NADH dehydrogenase (ubiquinone) flavoprotein 2 [EC:7.1.1.2 1.6.99.3]

NDUFA2; NADH dehydrogenase (ubiquinone) 1 alpha subcomplex subunit 2

NDUFA5; NADH dehydrogenase (ubiquinone) 1 alpha subcomplex subunit 5

NDUFA6; NADH dehydrogenase (ubiquinone) 1 alpha subcomplex subunit 6

NDUFA8; NADH dehydrogenase (ubiquinone) 1 alpha subcomplex subunit 8

NDUFA9; NADH dehydrogenase (ubiquinone) 1 alpha subcomplex subunit 9

NDUFAB1; NADH dehydrogenase (ubiquinone) 1 alpha/beta subcomplex 1, acyl-carrier protein

NDUFB8; NADH dehydrogenase (ubiquinone) 1 beta subcomplex subunit 8

NDUFB9; NADH dehydrogenase (ubiquinone) 1 beta subcomplex subunit 9

PKA; protein kinase A [EC:2.7.11.11]

UBE2J2; ubiquitin-conjugating enzyme E2 J2 [EC:2.3.2.23]

UBE2G2; ubiquitin-conjugating enzyme E2 G2 [EC:2.3.2.23]

GNAI; guanine nucleotide-binding protein G(i) subunit alpha

SLC25A4S; solute carrier family 25 (mitochondrial adenine nucleotide translocator), member 4/5/6/31

MFN2; mitofusin 2 [EC:3.6.5.-]

RPN13; 26S proteasome regulatory subunit N13

PSMD9; 26S proteasome regulatory subunit N4

CYC; cytochrome c

UBC; ubiquitin C

ERN1; serine/threonine-protein kinase/endoribonuclease IRE1 [EC:2.7.11.1 3.1.26.-]
KIF5; kinesin family member 5
SHFM1; 26 proteasome complex subunit DSS1
NDUFA12; NADH dehydrogenase (ubiquinone) 1 alpha subcomplex subunit 12
VDAC2; voltage-dependent anion channel protein 2

9. Ribosome

RP-L1; large subunit ribosomal protein L1
RP-L10; large subunit ribosomal protein L10
RP-L10Ae; large subunit ribosomal protein L10Ae
RP-L10e; large subunit ribosomal protein L10e
RP-L11; large subunit ribosomal protein L11
RP-L11e; large subunit ribosomal protein L11e
RP-L13; large subunit ribosomal protein L13
RP-L13Ae; large subunit ribosomal protein L13Ae
RP-L13e; large subunit ribosomal protein L13e
RP-L14e; large subunit ribosomal protein L14e
RP-L15; large subunit ribosomal protein L15
RP-L15e; large subunit ribosomal protein L15e
RP-L17e; large subunit ribosomal protein L17e
RP-L19e; large subunit ribosomal protein L19e
RP-L21; large subunit ribosomal protein L21
RP-L21e; large subunit ribosomal protein L21e
RP-L23; large subunit ribosomal protein L23
RP-L23e; large subunit ribosomal protein L23e
RP-L24; large subunit ribosomal protein L24
RP-L24e; large subunit ribosomal protein L24e
RP-L26e; large subunit ribosomal protein L26e
RP-L27; large subunit ribosomal protein L27
RP-L27Ae; large subunit ribosomal protein L27Ae
RP-L28; large subunit ribosomal protein L28

RP-L29e; large subunit ribosomal protein L29e

RP-L3; large subunit ribosomal protein L3

RP-L30; large subunit ribosomal protein L30

RP-L30e; large subunit ribosomal protein L30e

RP-L31e; large subunit ribosomal protein L31e

RP-L32; large subunit ribosomal protein L32

RP-L37e; large subunit ribosomal protein L37e

RP-L38e; large subunit ribosomal protein L38e

RP-L3e; large subunit ribosomal protein L3e

RP-L4; large subunit ribosomal protein L4

RP-L4e; large subunit ribosomal protein L4e

RP-L5; large subunit ribosomal protein L5

RP-L5e; large subunit ribosomal protein L5e

RP-L7; large subunit ribosomal protein L7/L12

RP-L7Ae; large subunit ribosomal protein L7Ae

RP-L7e; large subunit ribosomal protein L7e

RP-L9e; large subunit ribosomal protein L9e

RP-LP0; large subunit ribosomal protein LP0

RP-LP1; large subunit ribosomal protein LP1

RP-LP2; large subunit ribosomal protein LP2

RP-S10; small subunit ribosomal protein S10

RP-S11; small subunit ribosomal protein S11

RP-S12; small subunit ribosomal protein S12

RP-S12e; small subunit ribosomal protein S12e

RP-S14; small subunit ribosomal protein S14

RP-S15; small subunit ribosomal protein S15

RP-S15Ae; small subunit ribosomal protein S15Ae

RP-S15e; small subunit ribosomal protein S15e

RP-S16; small subunit ribosomal protein S16

RP-S17; small subunit ribosomal protein S17

RP-S17e; small subunit ribosomal protein S17e

RP-S18; small subunit ribosomal protein S18

RP-S18e; small subunit ribosomal protein S18e

RP-S2; small subunit ribosomal protein S2

RP-S20e; small subunit ribosomal protein S20e

RP-S23e; small subunit ribosomal protein S23e

RP-S25e; small subunit ribosomal protein S25e

RP-S26e; small subunit ribosomal protein S26e

RP-S27Ae; ubiquitin-small subunit ribosomal protein S27Ae

RP-S27e; small subunit ribosomal protein S27e

RP-S28e; small subunit ribosomal protein S28e

RP-S2e; small subunit ribosomal protein S2e

RP-S3Ae; small subunit ribosomal protein S3Ae

RP-S3e; small subunit ribosomal protein S3e

RP-S4e; small subunit ribosomal protein S4e

RP-S5; small subunit ribosomal protein S5

RP-S5e; small subunit ribosomal protein S5e

RP-S6; small subunit ribosomal protein S6

RP-S7; small subunit ribosomal protein S7

RP-S7e; small subunit ribosomal protein S7e

RP-S8e; small subunit ribosomal protein S8e

RP-S9; small subunit ribosomal protein S9

RP-SAe; small subunit ribosomal protein Sae

10. Carbon metabolism

MDH2; malate dehydrogenase [EC:1.1.1.37]

ME2; malate dehydrogenase (oxaloacetate-decarboxylating) [EC:1.1.1.38]

IDH3; isocitrate dehydrogenase (NAD+) [EC:1.1.1.41]

IDH1; isocitrate dehydrogenase [EC:1.1.1.42]

PGD; 6-phosphogluconate dehydrogenase [EC:1.1.1.44 1.1.1.343]

G6PD; glucose-6-phosphate 1-dehydrogenase [EC:1.1.1.49 1.1.1.363]

serA; D-3-phosphoglycerate dehydrogenase / 2-oxoglutarate reductase [EC:1.1.1.95 1.1.1.399]

frmA; S-(hydroxymethyl)glutathione dehydrogenase / alcohol dehydrogenase [EC:1.1.1.284 1.1.1.1]

FDH; formate dehydrogenase [EC:1.17.1.9]

GAPDH; glyceraldehyde 3-phosphate dehydrogenase [EC:1.2.1.12]

mmsA; malonate-semialdehyde dehydrogenase (acetylating) / methylmalonate-semialdehyde dehydrogenase [EC:1.2.1.18 1.2.1.27]

PDHA; pyruvate dehydrogenase E1 component alpha subunit [EC:1.2.4.1]

PDHB; pyruvate dehydrogenase E1 component beta subunit [EC:1.2.4.1]

OGDH; 2-oxoglutarate dehydrogenase E1 component [EC:1.2.4.2]

E1.3.3.6; acyl-CoA oxidase [EC:1.3.3.6]

SDHA; succinate dehydrogenase (ubiquinone) flavoprotein subunit [EC:1.3.5.1]

SDHB; succinate dehydrogenase (ubiquinone) iron-sulfur subunit [EC:1.3.5.1]

SDHD; succinate dehydrogenase (ubiquinone) membrane anchor subunit

GLDC; glycine dehydrogenase [EC:1.4.4.2]

metF; methylenetetrahydrofolate reductase (NADPH) [EC:1.5.1.20]

DLD; dihydrolipoamide dehydrogenase [EC:1.8.1.4]

glyA; glycine hydroxymethyltransferase [EC:2.1.2.1]

gcvT; aminomethyltransferase [EC:2.1.2.10]

E2.2.1.1; transketolase [EC:2.2.1.1]

E2.2.1.2; transaldolase [EC:2.2.1.2]

ACAT; acetyl-CoA C-acetyltransferase [EC:2.3.1.9]

DLAT; pyruvate dehydrogenase E2 component (dihydrolipoamide acetyltransferase) [EC:2.3.1.12]

DLST; 2-oxoglutarate dehydrogenase E2 component (dihydrolipoamide succinyltransferase) [EC:2.3.1.61]

GPT; alanine transaminase [EC:2.6.1.2]

AGXT; alanine-glyoxylate transaminase / serine-glyoxylate transaminase / serine-pyruvate transaminase [EC:2.6.1.44 2.6.1.45 2.6.1.51]

serC; phosphoserine aminotransferase [EC:2.6.1.52]

HK; hexokinase [EC:2.7.1.1]

pfkA; 6-phosphofructokinase 1 [EC:2.7.1.11]

E2.7.1.12; gluconokinase [EC:2.7.1.12]

DAK; triose/dihydroxyacetone kinase / FAD-AMP lyase (cyclizing) [EC:2.7.1.28 2.7.1.29 4.6.1.15]

PK; pyruvate kinase [EC:2.7.1.40]

PGK; phosphoglycerate kinase [EC:2.7.2.3]

PRPS; ribose-phosphate pyrophosphokinase [EC:2.7.6.1]

PGLS; 6-phosphogluconolactonase [EC:3.1.1.31]

frmB; S-formylglutathione hydrolase [EC:3.1.2.12]

serB; phosphoserine phosphatase [EC:3.1.3.3]

E3.5.1.49; formamidase [EC:3.5.1.49]

E4.1.1.49; phosphoenolpyruvate carboxykinase (ATP) [EC:4.1.1.49]

FBA; fructose-bisphosphate aldolase, class II [EC:4.1.2.13]

E4.1.3.1; isocitrate lyase [EC:4.1.3.1]

aceB; malate synthase [EC:2.3.3.9]

CS; citrate synthase [EC:2.3.3.1]

E4.2.1.2B; fumarate hydratase, class II [EC:4.2.1.2]

ACO; aconitate hydratase [EC:4.2.1.3]

ENO; enolase [EC:4.2.1.11]

cysK; cysteine synthase [EC:2.5.1.47]

E4.3.1.19; threonine dehydratase [EC:4.3.1.19]

rpe; ribulose-phosphate 3-epimerase [EC:5.1.3.1]

TPI; triosephosphate isomerase (TIM) [EC:5.3.1.1]

rpiA; ribose 5-phosphate isomerase A [EC:5.3.1.6]

GPI; glucose-6-phosphate isomerase [EC:5.3.1.9]

PGAM; 2,3-bisphosphoglycerate-dependent phosphoglycerate mutase [EC:5.4.2.11]

ACSS1_2; acetyl-CoA synthetase [EC:6.2.1.1]

LSC1; succinyl-CoA synthetase alpha subunit [EC:6.2.1.4 6.2.1.5]

LSC2; succinyl-CoA synthetase beta subunit [EC:6.2.1.4 6.2.1.5]

PC; pyruvate carboxylase [EC:6.4.1.1]

gcvH; glycine cleavage system H protein

katE; catalase [EC:1.11.1.6]

FBP; fructose-1,6-bisphosphatase I [EC:3.1.3.11]

HIBCH; 3-hydroxyisobutyryl-CoA hydrolase [EC:3.1.2.4]

HAO; (S)-2-hydroxy-acid oxidase [EC:1.1.3.15]

GOT1; aspartate aminotransferase, cytoplasmic [EC:2.6.1.1]

GOT2; aspartate aminotransferase, mitochondrial [EC:2.6.1.1]

FOX2; multifunctional beta-oxidation protein [EC:4.2.1.- 1.1.1.-]

GLYK; D-glycerate 3-kinase [EC:2.7.1.31]

MET17; O-acetylhomoserine/O-acetylserine sulfhydrylase [EC:2.5.1.49 2.5.1.47]

DAS; dihydroxyacetone synthase [EC:2.2.1.3]

SDS; L-serine/L-threonine ammonia-lyase [EC:4.3.1.17 4.3.1.19]

HPD1; 3-hydroxyisobutyrate/3-hydroxypropionate dehydrogenase [EC:1.1.1.31 1.1.1.59]

11. RNA transport

rnz; ribonuclease Z [EC:3.1.26.11]

cca; tRNA nucleotidyltransferase (CCA-adding enzyme) [EC:2.7.7.72 3.1.3.- 3.1.4.-]

POP1; ribonuclease P/MRP protein subunit POP1 [EC:3.1.26.5]

PRMT5; type II protein arginine methyltransferase [EC:2.1.1.320]

EEF1A; elongation factor 1-alpha

EIF1A; translation initiation factor 1A

EIF2S1; translation initiation factor 2 subunit 1

EIF2S2; translation initiation factor 2 subunit 2

EIF2B1; translation initiation factor eIF-2B subunit alpha

EIF2B5; translation initiation factor eIF-2B subunit epsilon

EIF2B3; translation initiation factor eIF-2B subunit gamma

EIF2S3; translation initiation factor 2 subunit 3

EIF5B; translation initiation factor 5B

EIF3J; translation initiation factor 3 subunit J

EIF3I; translation initiation factor 3 subunit I

EIF3H; translation initiation factor 3 subunit H

EIF3G; translation initiation factor 3 subunit G

EIF3F; translation initiation factor 3 subunit F

EIF3C; translation initiation factor 3 subunit C

EIF3B; translation initiation factor 3 subunit B

EIF3A; translation initiation factor 3 subunit A

EIF4A; translation initiation factor 4A

EIF4B; translation initiation factor 4B

EIF4E; translation initiation factor 4E

EIF4G; translation initiation factor 4G

EIF5; translation initiation factor 5

POP4; ribonuclease P protein subunit POP4 [EC:3.1.26.5]

RPP1; ribonuclease P/MRP protein subunit RPP1 [EC:3.1.26.5]

EIF2B4; translation initiation factor eIF-2B subunit delta

EIF2B2; translation initiation factor eIF-2B subunit beta

NMD3; nonsense-mediated mRNA decay protein 3

RAN; GTP-binding nuclear protein Ran

TPR; nucleoprotein TPR

UBE2I; ubiquitin-conjugating enzyme E2 I

DDX39B; ATP-dependent RNA helicase UAP56/SUB2 [EC:3.6.4.13]

THOC2; THO complex subunit 2

THOC3; THO complex subunit 3

NCBP1; nuclear cap-binding protein subunit 1

NCBP2; nuclear cap-binding protein subunit 2

EIF4A3; ATP-dependent RNA helicase [EC:3.6.4.13]

PABPC; polyadenylate-binding protein

GEMIN2; gem associated protein 2

SRRM1; serine/arginine repetitive matrix protein 1

SEC13; protein transport protein SEC13

NXF; nuclear RNA export factor

XPOT; exportin-T

XPO5; exportin-5

XPO1; exportin-1

TGS1; trimethylguanosine synthase [EC:2.1.1.-]

KPNB1; importin subunit beta-1

NUP98; nuclear pore complex protein Nup98-Nup96

RAE1; mRNA export factor

SEH1; nucleoporin SEH1

NUP133; nuclear pore complex protein Nup133

NUP160; nuclear pore complex protein Nup160

NUP85; nuclear pore complex protein Nup85

NUP62; nuclear pore complex protein Nup62

NUPL1; nucleoporin p58/p45

NUP54; nuclear pore complex protein Nup54

NUP93; nuclear pore complex protein Nup93

NUP205; nuclear pore complex protein Nup205

NUP188; nuclear pore complex protein Nup188

NUP155; nuclear pore complex protein Nup155

NDC1; nucleoporin NDC1

RANGAP1; Ran GTPase-activating protein 1

UPF1; regulator of nonsense transcripts 1 [EC:3.6.4.-]

UPF2; regulator of nonsense transcripts 2

UPF3; regulator of nonsense transcripts 3

POP3; ribonuclease P/MRP protein subunit POP3 [EC:3.1.26.5]

POP7; ribonuclease P/MRP protein subunit POP7 [EC:3.1.26.5]

RPM2; ribonuclease P protein component, mitochondrial [EC:3.1.26.5]

12. MAPK signaling pathway – yeast

GPD1; glycerol-3-phosphate dehydrogenase (NAD+) [EC:1.1.1.8]

E2.4.1.34; 1,3-beta-glucan synthase [EC:2.4.1.34]

PI4KA; phosphatidylinositol 4-kinase A [EC:2.7.1.67]

PIP5K; 1-phosphatidylinositol-4-phosphate 5-kinase [EC:2.7.1.68]

CSNK1; casein kinase 1 [EC:2.7.11.1]

HSL1; serine/threonine-protein kinase HSL1, negative regulator of Swe1 kinase [EC:2.7.11.-]

PRMT5; type II protein arginine methyltransferase [EC:2.1.1.320]

MIH1; M-phase inducer tyrosine phosphatase [EC:3.1.3.48]

PRKCA; classical protein kinase C alpha type [EC:2.7.11.13]

SWE1; mitosis inhibitor protein kinase SWE1 [EC:2.7.11.1]

katE; catalase [EC:1.11.1.6]

ERK; mitogen-activated protein kinase 1/3 [EC:2.7.11.24]

CDC42; cell division control protein 42

PAK1; p21-activated kinase 1 [EC:2.7.11.1]

P38; p38 MAP kinase [EC:2.7.11.24]

MAPK7; mitogen-activated protein kinase 7 [EC:2.7.11.24]

RHOA; Ras homolog gene family, member A

GNB1; guanine nucleotide-binding protein G(I)/G(S)/G(T) subunit beta-1

YPS1_2; yapsin 1/2 [EC:3.4.23.41]

PDPK1; 3-phosphoinositide dependent protein kinase-1 [EC:2.7.11.1]

YWHAE; 14-3-3 protein epsilon

SWI6; regulatory protein SWI6

SWI4; regulatory protein SWI4

FAR1; cyclin-dependent kinase inhibitor FAR1

SSN6; general transcriptional corepressor CYC8

TUP1; general transcriptional corepressor TUP1

MKK1_2; mitogen-activated protein kinase kinase [EC:2.7.12.2]

SKO1; ATF/CREB family transcription factor

TEAD; transcriptional enhancer factor

MSN2_4; zinc finger protein MSN2/4

NEDD4; E3 ubiquitin-protein ligase NEDD4 [EC:2.3.2.26]

STE2; pheromone alpha factor receptor

STE12; transcription factor STE12

STE7; mitogen-activated protein kinase kinase [EC:2.7.12.2]

PBS2; mitogen-activated protein kinase kinase [EC:2.7.12.2]

STE11; mitogen-activated protein kinase kinase kinase [EC:2.7.11.25]

BCK1; mitogen-activated protein kinase kinase kinase [EC:2.7.11.25]

SSK2; mitogen-activated protein kinase kinase kinase [EC:2.7.11.25]

SLN1; osomolarity two-component system, sensor histidine kinase SLN1 [EC:2.7.13.3]

YPD1; osomolarity two-component system, phosphorelay intermediate protein YPD1

SSK1; osomolarity two-component system, response regulator SSK1

CDC24; cell division control protein 24

BEM1; bud emergence protein 1

BNI1; cytokinesis protein

STE5; pheromone-response scaffold protein

MSG5; tyrosine-protein phosphatase MSG5 [EC:3.1.3.48]

WSC; cell wall integrity and stress response component

SHO1; SHO1 osmosensor

MCM1; pheromone receptor transcription factor

PAF1; RNA polymerase II-associated factor 1

GRE2; NADPH-dependent methylglyoxal reductase [EC:1.1.1.283]

BNR1; BNI1-related protein 1

PTC1; protein phosphatase PTC1 [EC:3.1.3.16]

PTP2_3; tyrosine-protein phosphatase 2/3 [EC:3.1.3.48]

CLA4; serine/threonine-protein kinase CLA4 [EC:2.7.11.1]

SST2; GTPase-activating protein SST2

RGA1_2; Rho-type GTPase-activating protein 1/2

BEM3; Rho-type GTPase-activating protein

STE50; protein STE50

ROM1_2; RHO1 GDP-GTP exchange protein 1/2

TUS1; Rho1 guanine nucleotide exchange factor TUS1

BEM2; GTPase-activating protein BEM2

SAC7; GTPase-activating protein SAC7

SPA2; protein SPA2

OPY2; protein OPY2

GPA1; guanine nucleotide-binding protein alpha-1 subunit

CLB2; G2/mitotic-specific cyclin 2

13. Thermogenesis

SDHA; succinate dehydrogenase (ubiquinone) flavoprotein subunit [EC:1.3.5.1]

SDHB; succinate dehydrogenase (ubiquinone) iron-sulfur subunit [EC:1.3.5.1]

SDHD; succinate dehydrogenase (ubiquinone) membrane anchor subunit

UQCRFS1; ubiquinol-cytochrome c reductase iron-sulfur subunit [EC:7.1.1.8]

CYC1; ubiquinol-cytochrome c reductase cytochrome c1 subunit

QCR1; ubiquinol-cytochrome c reductase core subunit 1

QCR2; ubiquinol-cytochrome c reductase core subunit 2

QCR7; ubiquinol-cytochrome c reductase subunit 7

QCR8; ubiquinol-cytochrome c reductase subunit 8

MGLL; acylglycerol lipase [EC:3.1.1.23]

ACSL; long-chain acyl-CoA synthetase [EC:6.2.1.3]

ATPeF0B; F-type H+-transporting ATPase subunit b

ATPeF1A; F-type H+-transporting ATPase subunit alpha

ATPeF1B; F-type H+-transporting ATPase subunit beta [EC:7.1.2.2]

ATPeF1D; F-type H+-transporting ATPase subunit delta

ATPeF1G; F-type H+-transporting ATPase subunit gamma

ATPeF0O; F-type H+-transporting ATPase subunit O

ATPeF0D; F-type H+-transporting ATPase subunit d

ATPeFG; F-type H+-transporting ATPase subunit g

COX10; heme o synthase [EC:2.5.1.141]

COX11; cytochrome c oxidase assembly protein subunit 11

COX15; cytochrome c oxidase assembly protein subunit 15

COX5B; cytochrome c oxidase subunit 5b

COX6A; cytochrome c oxidase subunit 6a

COX6B; cytochrome c oxidase subunit 6b

COX7C; cytochrome c oxidase subunit 7c

SOS; son of sevenless

NDUFS1; NADH dehydrogenase (ubiquinone) Fe-S protein 1 [EC:7.1.1.2 1.6.99.3]

NDUFS2; NADH dehydrogenase (ubiquinone) Fe-S protein 2 [EC:7.1.1.2 1.6.99.3]

NDUFS3; NADH dehydrogenase (ubiquinone) Fe-S protein 3 [EC:7.1.1.2 1.6.99.3]

NDUFS4; NADH dehydrogenase (ubiquinone) Fe-S protein 4

NDUFS6; NADH dehydrogenase (ubiquinone) Fe-S protein 6

NDUFS7; NADH dehydrogenase (ubiquinone) Fe-S protein 7 [EC:7.1.1.2 1.6.99.3]

NDUFS8; NADH dehydrogenase (ubiquinone) Fe-S protein 8 [EC:7.1.1.2 1.6.99.3]

NDUFV1; NADH dehydrogenase (ubiquinone) flavoprotein 1 [EC:7.1.1.2 1.6.99.3]

NDUFV2; NADH dehydrogenase (ubiquinone) flavoprotein 2 [EC:7.1.1.2 1.6.99.3]

NDUFA2; NADH dehydrogenase (ubiquinone) 1 alpha subcomplex subunit 2

NDUFA5; NADH dehydrogenase (ubiquinone) 1 alpha subcomplex subunit 5

NDUFA6; NADH dehydrogenase (ubiquinone) 1 alpha subcomplex subunit 6

NDUFA8; NADH dehydrogenase (ubiquinone) 1 alpha subcomplex subunit 8

NDUFA9; NADH dehydrogenase (ubiquinone) 1 alpha subcomplex subunit 9

NDUFAB1; NADH dehydrogenase (ubiquinone) 1 alpha/beta subcomplex 1, acyl-carrier protein

NDUFB8; NADH dehydrogenase (ubiquinone) 1 beta subcomplex subunit 8

NDUFB9; NADH dehydrogenase (ubiquinone) 1 beta subcomplex subunit 9

PKA; protein kinase A [EC:2.7.11.11]

P38; p38 MAP kinase [EC:2.7.11.24]

PRKAB; 5'-AMP-activated protein kinase, regulatory beta subunit

PRKAG; 5'-AMP-activated protein kinase, regulatory gamma subunit

MTOR; serine/threonine-protein kinase mTOR [EC:2.7.11.1]

RAPTOR; regulatory associated protein of mTOR

RHEB; Ras homolog enriched in brain

KRAS; GTPase KRas

MLST8; target of rapamycin complex subunit LST8

NDUFAF3; NADH dehydrogenase [ubiquinone] 1 alpha subcomplex assembly factor 3

NDUFA12; NADH dehydrogenase (ubiquinone) 1 alpha subcomplex subunit 12

SMARCB1; SWI/SNF-related matrix-associated actin-dependent regulator of chromatin subfamily B member 1

SLC25A20_29; solute carrier family 25 (mitochondrial carnitine/acylcarnitine transporter), member 20/29

COX18; mitochondrial inner membrane protein COX18

NDUFAF1; NADH dehydrogenase [ubiquinone] 1 alpha subcomplex assembly factor 1

NDUFAF2; NADH dehydrogenase [ubiquinone] 1 alpha subcomplex assembly factor 2

NDUFAF5; NADH dehydrogenase [ubiquinone] 1 alpha subcomplex assembly factor 5 [EC:2.1.1.-]

NDUFAF6; NADH dehydrogenase [ubiquinone] 1 alpha subcomplex assembly factor 6

NDUFAF7; NADH dehydrogenase [ubiquinone] 1 alpha subcomplex assembly factor 7

COA1; cytochrome c oxidase assembly factor 1

COA6; cytochrome c oxidase assembly factor 6

COX16; cytochrome c oxidase assembly protein subunit 16

COX20; cytochrome c oxidase assembly protein subunit 20

14. Meiosis - yeast

E4.6.1.1; adenylate cyclase [EC:4.6.1.1]

BUB1; checkpoint serine/threonine-protein kinase [EC:2.7.11.1]

MCM5; DNA replication licensing factor MCM5 [EC:3.6.4.12]

MCM7; DNA replication licensing factor MCM7 [EC:3.6.4.12]

MCM4; DNA replication licensing factor MCM4 [EC:3.6.4.12]

CDC6; cell division control protein 6

CDC7; cell division control protein 7 [EC:2.7.11.1]

DBF4; regulatory subunit for Cdc7p protein kinase

ESP1; separase [EC:3.4.22.49]

MCM2; DNA replication licensing factor MCM2 [EC:3.6.4.12]

MCM3; DNA replication licensing factor MCM3 [EC:3.6.4.12]

MCM6; DNA replication licensing factor MCM6 [EC:3.6.4.12]

MEC1; cell cycle checkpoint protein MEC1

MEC3; G2-specific checkpoint protein

ORC1; origin recognition complex subunit 1

ORC2; origin recognition complex subunit 2

ORC4; origin recognition complex subunit 4

ORC5; origin recognition complex subunit 5

ORC6; origin recognition complex subunit 6

HRAD1; cell cycle checkpoint protein [EC:3.1.11.2]

SWE1; mitosis inhibitor protein kinase SWE1 [EC:2.7.11.1]

APC1; anaphase-promoting complex subunit 1

APC2; anaphase-promoting complex subunit 2

APC3; anaphase-promoting complex subunit 3

APC4; anaphase-promoting complex subunit 4

APC5; anaphase-promoting complex subunit 5

APC6; anaphase-promoting complex subunit 6

APC8; anaphase-promoting complex subunit 8

APC10; anaphase-promoting complex subunit 10

APC11; anaphase-promoting complex subunit 11

CDC20; cell division cycle 20, cofactor of APC complex

PPP2R1; serine/threonine-protein phosphatase 2A regulatory subunit A

PKA; protein kinase A [EC:2.7.11.11]

GNAI; guanine nucleotide-binding protein G(i) subunit alpha

PPP1C; serine/threonine-protein phosphatase PP1 catalytic subunit [EC:3.1.3.16]

CDC45; cell division control protein 45

SMC1; structural maintenance of chromosome 1

CDC14; cell division cycle 14 [EC:3.1.3.16 3.1.3.48]

CLN3; G1/S-specific cyclin CLN3

SWI6; regulatory protein SWI6

SWI4; regulatory protein SWI4

CLB3_4; G2/mitotic-specific cyclin 3/4

CDC5; cell cycle serine/threonine-protein kinase CDC5/MSD2 [EC:2.7.11.21]

RAD9; DNA repair protein RAD9

DDC1; DNA damage checkpoint protein

SMC3; structural maintenance of chromosome 3 (chondroitin sulfate proteoglycan 6)

STAG1_2; cohesin complex subunit SA-1/2

MAD1; mitotic spindle assembly checkpoint protein MAD1

CDC15; cell division control protein CDC15 [EC:2.7.11.1]

MTOR; serine/threonine-protein kinase mTOR [EC:2.7.11.1]

KRAS; GTPase KRas

HXT; MFS transporter, SP family, sugar:H+ symporter

RME1; zinc finger protein RME1

MSN2_4; zinc finger protein MSN2/4

SPO11; meiotic recombination protein SPO11

SGO1; shugoshin

PPP2R5; serine/threonine-protein phosphatase 2A regulatory subunit B'

SNF1; carbon catabolite-derepressing protein kinase [EC:2.7.11.1]

GPR1; G protein-coupled receptor GPR1

EFG1; enhanced filamentous growth protein 1

IME2; meiosis induction protein kinase IME2/SME1 [EC:2.7.11.1]

RIM15; serine/threonine-protein kinase RIM15 [EC:2.7.11.1]

NDT80; meiosis-specific transcription factor NDT80

SPS1; sporulation-specific protein 1 [EC:2.7.11.1]

REC8; meiotic recombination protein REC8, fungi type

AMA1; meiosis-specific APC/C activator protein AMA1

15. Protein processing in endoplasmic reticulum

MOGS; mannosyl-oligosaccharide glucosidase [EC:3.2.1.106]

E3.5.1.52; peptide-N4-(N-acetyl-beta-glucosaminyl)asparagine amidase [EC:3.5.1.52]

SKP1; S-phase kinase-associated protein 1

EIF2S1; translation initiation factor 2 subunit 1

HSPA1s; heat shock 70kDa protein 1/2/6/8

CUL1; cullin 1

HSP90A; molecular chaperone HtpG

UBQLN; ubiquilin

UBE2J2; ubiquitin-conjugating enzyme E2 J2 [EC:2.3.2.23]

UBE2G2; ubiquitin-conjugating enzyme E2 G2 [EC:2.3.2.23]

UBE2D; ubiquitin-conjugating enzyme E2 D [EC:2.3.2.23]

STT3; dolichyl-diphosphooligosaccharide---protein glycosyltransferase [EC:2.4.99.18]

SAR1; GTP-binding protein SAR1 [EC:3.6.5.-]

CANX; calnexin

PRKCSH; protein kinase C substrate 80K-H

ERN1; serine/threonine-protein kinase/endoribonuclease IRE1 [EC:2.7.11.1 3.1.26.-]

HSP110; heat shock protein 110kDa

HYOU1; hypoxia up-regulated 1

DNAJA2; DnaJ homolog subfamily A member 2

DNAJB12; DnaJ homolog subfamily B member 12

DNAJC3; DnaJ homolog subfamily C member 3

SEC63; translocation protein SEC63

STUB1; STIP1 homology and U-box containing protein 1 [EC:2.3.2.27]

HSPBP1; hsp70-interacting protein

PDIA1; protein disulfide-isomerase A1 [EC:5.3.4.1]

PDIA6; protein disulfide-isomerase A6 [EC:5.3.4.1]

LMAN1; lectin, mannose-binding 1

LMAN2; lectin, mannose-binding 2

OS9; protein OS-9

UBE4B; ubiquitin conjugation factor E4 B [EC:2.3.2.27]

SYVN1; E3 ubiquitin-protein ligase synoviolin [EC:2.3.2.27]

MARCH6; E3 ubiquitin-protein ligase MARCH6 [EC:2.3.2.27]

RAD23; UV excision repair protein RAD23

SEC61A; protein transport protein SEC61 subunit alpha

ERO1LB; ERO1-like protein beta [EC:1.8.4.-]

HUGT; UDP-glucose:glycoprotein glucosyltransferase [EC:2.4.1.-]

SEC62; translocation protein SEC62

OST1; oligosaccharyltransferase complex subunit alpha (ribophorin I)

SWP1; oligosaccharyltransferase complex subunit delta (ribophorin II)

OST2; oligosaccharyltransferase complex subunit epsilon

OST3; oligosaccharyltransferase complex subunit gamma

WBP1; oligosaccharyltransferase complex subunit beta

OTU1; ubiquitin thioesterase OTU1 [EC:3.1.2.-]

DERL2_3; Derlin-2/3

EPS1; protein disulfide-isomerase [EC:5.3.4.1]

SIL1; nucleotide exchange factor SIL1

SCJ1; DnaJ-related protein SCJ1

PREB; prolactin regulatory element-binding protein

SEC13; protein transport protein SEC13

SEC31; protein transport protein SEC31

SEC23; protein transport protein SEC23

SEC24; protein transport protein SEC24

BCAP31; B-cell receptor-associated protein 31

SHP1; UBX domain-containing protein 1

UBX2; UBX domain-containing protein 2

NPLOC4; nuclear protein localization protein 4 homolog

UFD1; ubiquitin fusion degradation protein 1

SNL1; HSP70 co-chaperone SNL1

PLAA; phospholipase A-2-activating protein

CUE1; coupling of ubiquitin conjugation to ER degradation protein 1

HRD3; ERAD-associated E3 ubiquitin-protein ligase component HRD3

USA1; U1 SNP1-associating protein 1

EIF2AK4; eukaryotic translation initiation factor 2-alpha kinase 4 [EC:2.7.11.1]

MAN1B; endoplasmic reticulum Man9GlcNAc2 1,2-alpha-mannosidase [EC:3.2.1.209]

16. Ribosome biogenesis in eukaryotes

POP1; ribonuclease P/MRP protein subunit POP1 [EC:3.1.26.5]

CSNK2A; casein kinase II subunit alpha [EC:2.7.11.1]

CSNK2B; casein kinase II subunit beta

EIF6; translation initiation factor 6

POP4; ribonuclease P protein subunit POP4 [EC:3.1.26.5]

RPP1; ribonuclease P/MRP protein subunit RPP1 [EC:3.1.26.5]

rnc; ribonuclease III [EC:3.1.26.3]

NOG1; nucleolar GTP-binding protein

RIOK1; RIO kinase 1 [EC:2.7.11.1]

RIOK2; RIO kinase 2 [EC:2.7.11.1]

NMD3; nonsense-mediated mRNA decay protein 3

RAN; GTP-binding nuclear protein Ran

RCL1; RNA 3'-terminal phosphate cyclase-like protein

GAR1; H/ACA ribonucleoprotein complex subunit 1

NHP2; H/ACA ribonucleoprotein complex subunit 2

DKC1; H/ACA ribonucleoprotein complex subunit 4 [EC:5.4.99.-]

NOB1; RNA-binding protein NOB1

XRN1; 5'-3' exoribonuclease 1 [EC:3.1.13.-]

XRN2; 5'-3' exoribonuclease 2 [EC:3.1.13.-]

SNU13; U4/U6 small nuclear ribonucleoprotein SNU13

orn; oligoribonuclease [EC:3.1.-.-]

NXF; nuclear RNA export factor

XPO1; exportin-1

NAT10; N-acetyltransferase 10 [EC:2.3.1.-]

POP3; ribonuclease P/MRP protein subunit POP3 [EC:3.1.26.5]

POP7; ribonuclease P/MRP protein subunit POP7 [EC:3.1.26.5]

SNM1; ribonuclease MRP protein subunit SNM1

RMP1; ribonuclease MRP protein subunit RMP1

RIA1; ribosome assembly protein 1 [EC:3.6.5.-]

NUG2; nuclear GTP-binding protein

NUG1; nuclear GTP-binding protein

LSG1; large subunit GTPase 1 [EC:3.6.1.-]

UTP22; U3 small nucleolar RNA-associated protein 22

RRP7; ribosomal RNA-processing protein 7

UTP5; U3 small nucleolar RNA-associated protein 5

UTP8; U3 small nucleolar RNA-associated protein 8

UTP4; U3 small nucleolar RNA-associated protein 4

UTP15; U3 small nucleolar RNA-associated protein 15

UTP10; U3 small nucleolar RNA-associated protein 10

UTP9; U3 small nucleolar RNA-associated protein 9

NAN1; NET1-associated nuclear protein 1 (U3 small nucleolar RNA-associated protein 17)

UTP18; U3 small nucleolar RNA-associated protein 18

UTP13; U3 small nucleolar RNA-associated protein 13

DIP2; U3 small nucleolar RNA-associated protein 12

UTP6; U3 small nucleolar RNA-associated protein 6

PWP2; periodic tryptophan protein 2

MPP10; U3 small nucleolar RNA-associated protein MPP10

IMP3; U3 small nucleolar ribonucleoprotein protein IMP3

IMP4; U3 small nucleolar ribonucleoprotein protein IMP4

NOP1; rRNA 2'-O-methyltransferase fibrillarin [EC:2.1.1.-]

NOP56; nucleolar protein 56

NOP58; nucleolar protein 58

UTP24; U3 small nucleolar RNA-associated protein 24

UTP14; U3 small nucleolar RNA-associated protein 14

BMS1; ribosome biogenesis protein BMS1

REX1; RNA exonuclease 1 [EC:3.1.-.-]

RIX7; ribosome biogenesis ATPase

MDN1; midasin

NOP4; nucleolar protein 4

SDO1; ribosome maturation protein SDO1

AFG2; AAA family ATPase

HRR25; casein kinase I homolog HRR25 [EC:2.7.11.1]

AK6; adenylate kinase [EC:2.7.4.3]

17. Autophagy – yeast

SPT; serine palmitoyltransferase [EC:2.3.1.50]

PIK3C3; phosphatidylinositol 3-kinase [EC:2.7.1.137]

IPMK; inositol-polyphosphate multikinase [EC:2.7.1.140 2.7.1.151]

E3.4.21.48; cerevisin [EC:3.4.21.48]

PEP4; saccharopepsin [EC:3.4.23.25]

EIF2S1; translation initiation factor 2 subunit 1

PKA; protein kinase A [EC:2.7.11.11]

MAPK7; mitogen-activated protein kinase 7 [EC:2.7.11.24]

NSF; vesicle-fusing ATPase [EC:3.6.4.6]

PHO85; negative regulator of the PHO system [EC:2.7.11.22]

MTOR; serine/threonine-protein kinase mTOR [EC:2.7.11.1]

RAPTOR; regulatory associated protein of mTOR

IP6K; inositol-hexakisphosphate 5-kinase [EC:2.7.4.21]

KRAS; GTPase KRas

RAB7A; Ras-related protein Rab-7A

MLST8; target of rapamycin complex subunit LST8

ULK2; serine/threonine-protein kinase ULK2 [EC:2.7.11.1]

ATG17; autophagy-related protein 17

ATG11; autophagy-related protein 11

ATG13; autophagy-related protein 13

PIK3R4; phosphoinositide-3-kinase, regulatory subunit 4 [EC:2.7.11.1]

BECN; beclin

ATG7; ubiquitin-like modifier-activating enzyme ATG7

ATG5; autophagy-related protein 5

ATG16; autophagy-related protein 16

GABARAP; GABA(A) receptor-associated protein

ATG4; cysteine protease ATG4 [EC:3.4.22.-]

ATG3; ubiquitin-like-conjugating enzyme ATG3

VTI1; vesicle transport through interaction with t-SNAREs 1

VAM7; regulator of vacuolar morphogenesis

YKT6; synaptobrevin homolog YKT6

GCN4; general control protein GCN4

MSN2_4; zinc finger protein MSN2/4

VPS45; vacuolar protein sorting-associated protein 45

SNF1; carbon catabolite-derepressing protein kinase [EC:2.7.11.1]

RIM15; serine/threonine-protein kinase RIM15 [EC:2.7.11.1]

SLC36A; solute carrier family 36 (proton-coupled amino acid transporter)

NAPA; alpha-soluble NSF attachment protein

EIF2AK4; eukaryotic translation initiation factor 2-alpha kinase 4 [EC:2.7.11.1]

ACTR2; actin-related protein 2

IGBP1; immunoglobulin-binding protein 1

TIPRL; type 2A phosphatase activator TIP41

ATG15; lipase ATG15 [EC:3.1.1.3]

ATG2; autophagy-related protein 2

ATG9; autophagy-related protein 9

WIPI1_2; autophagy-related protein 18

MTMR6_7_8; myotubularin-related protein 6/7/8 [EC:3.1.3.64 3.1.3.95]

ACTR3; actin-related protein 3

SCH9; serine/threonine protein kinase SCH9 [EC:2.7.11.1]

VPS3; vacuolar protein sorting-associated protein 3

VPS8; vacuolar protein sorting-associated protein 8

VPS11; vacuolar protein sorting-associated protein 11

VPS16; vacuolar protein sorting-associated protein 16

VPS18; vacuolar protein sorting-associated protein 18

VPS33A; vacuolar protein sorting-associated protein 33A

VPS41; vacuolar protein sorting-associated protein 41

MON1; vacuolar fusion protein MON1

ATG27; autophagy-related protein 27

VAM3; syntaxin VAM3

CCZ1; vacuolar fusion protein CCZ1

SAK1; SNF1-activating kinase 1 [EC:2.7.11.1]

PCL5; PHO85 cyclin-5

18. Endocytosis

PIP5K; 1-phosphatidylinositol-4-phosphate 5-kinase [EC:2.7.1.68]

PLD1_2; phospholipase D1/2 [EC:3.1.4.4]

HSPA1s; heat shock 70kDa protein 1/2/6/8

CDC42; cell division control protein 42

RHOA; Ras homolog gene family, member A

CLTC; clathrin heavy chain

STAM; signal transducing adaptor molecule

ARPC5; actin related protein 2/3 complex, subunit 5

ARPC4; actin related protein 2/3 complex, subunit 4

ARPC3; actin related protein 2/3 complex, subunit 3

ARPC1A_B; actin related protein 2/3 complex, subunit 1A/1B

ARPC2; actin related protein 2/3 complex, subunit 2

RAB5C; Ras-related protein Rab-5C

RAB7A; Ras-related protein Rab-7A

RAB8A; Ras-related protein Rab-8A

RAB11B; Ras-related protein Rab-11B

ARF1_2; ADP-ribosylation factor 1/2

ARF6; ADP-ribosylation factor 6

CAPZA; capping protein (actin filament) muscle Z-line, alpha

KIF5; kinesin family member 5

NEDD4; E3 ubiquitin-protein ligase NEDD4 [EC:2.3.2.26]

AP2A; AP-2 complex subunit alpha

AP2B1; AP-2 complex subunit beta-1

AP2M1; AP-2 complex subunit mu-1

AP2S1; AP-2 complex subunit sigma-1

USP8; ubiquitin carboxyl-terminal hydrolase 8 [EC:3.4.19.12]

HGS; hepatocyte growth factor-regulated tyrosine kinase substrate

TSG101; ESCRT-I complex subunit TSG101

VPS28; ESCRT-I complex subunit VPS28

SNF8; ESCRT-II complex subunit VPS22

VPS25; ESCRT-II complex subunit VPS25

VPS36; ESCRT-II complex subunit VPS36

CHMP2A; charged multivesicular body protein 2A

VPS24; charged multivesicular body protein 3

CHMP4; charged multivesicular body protein 4

CHMP6; charged multivesicular body protein 6

VPS4; vacuolar protein-sorting-associated protein 4

CHMP1; charged multivesicular body protein 1

CHMP5; charged multivesicular body protein 5

VTA1; vacuolar protein sorting-associated protein VTA1

PDCD6IP; programmed cell death 6-interacting protein

EPN; epsin

EPS15; epidermal growth factor receptor substrate 15

VPS45; vacuolar protein sorting-associated protein 45

RBSN; rabenosyn-5

SMAP; stromal membrane-associated protein

ASAP; Arf-GAP with SH3 domain, ANK repeat and PH domain-containing protein

ARFGAP1; ADP-ribosylation factor GTPase-activating protein 1

ARFGAP2_3; ADP-ribosylation factor GTPase-activating protein 2/3

AMPH; amphiphysin

CHMP7; charged multivesicular body protein 7

SNX4; sorting nexin-4

ARFGEF; brefeldin A-inhibited guanine nucleotide-exchange protein

GBF1; golgi-specific brefeldin A-resistance guanine nucleotide exchange factor 1

VPS26; vacuolar protein sorting-associated protein 26

VPS29; vacuolar protein sorting-associated protein 29

VPS35; vacuolar protein sorting-associated protein 35

IST1; vacuolar protein sorting-associated protein IST1

WASL; neural Wiskott-Aldrich syndrome protein

19. Oxidative phosphorylation

SDHA; succinate dehydrogenase (ubiquinone) flavoprotein subunit [EC:1.3.5.1]

SDHB; succinate dehydrogenase (ubiquinone) iron-sulfur subunit [EC:1.3.5.1]

SDHD; succinate dehydrogenase (ubiquinone) membrane anchor subunit

UQCRFS1; ubiquinol-cytochrome c reductase iron-sulfur subunit [EC:7.1.1.8]

CYC1; ubiquinol-cytochrome c reductase cytochrome c1 subunit

QCR1; ubiquinol-cytochrome c reductase core subunit 1

QCR2; ubiquinol-cytochrome c reductase core subunit 2

QCR7; ubiquinol-cytochrome c reductase subunit 7

QCR8; ubiquinol-cytochrome c reductase subunit 8

ppa; inorganic pyrophosphatase [EC:3.6.1.1]

PMA1; H+-transporting ATPase [EC:7.1.2.1]

TIM11; F-type H+-transporting ATP synthase subunit e

ATPeF0B; F-type H+-transporting ATPase subunit b

ATPeF1A; F-type H+-transporting ATPase subunit alpha

ATPeF1B; F-type H+-transporting ATPase subunit beta [EC:7.1.2.2]

ATPeF1D; F-type H+-transporting ATPase subunit delta

ATPeF1G; F-type H+-transporting ATPase subunit gamma

ATPeF0O; F-type H+-transporting ATPase subunit O

ATPeF0D; F-type H+-transporting ATPase subunit d

ATPeFG; F-type H+-transporting ATPase subunit g

ATPeFK; F-type H+-transporting ATPase subunit k

ATPeV1A; V-type H+-transporting ATPase subunit A [EC:7.1.2.2]

ATPeV0D; V-type H+-transporting ATPase subunit d

ATPeV1B; V-type H+-transporting ATPase subunit B

ATPeV1C; V-type H+-transporting ATPase subunit C

ATPeV1D; V-type H+-transporting ATPase subunit D

ATPeV1G; V-type H+-transporting ATPase subunit G

ATPeV0A; V-type H+-transporting ATPase subunit a

ATPeV0C; V-type H+-transporting ATPase 16kDa proteolipid subunit

COX10; heme o synthase [EC:2.5.1.141]

COX11; cytochrome c oxidase assembly protein subunit 11

COX15; cytochrome c oxidase assembly protein subunit 15

COX5B; cytochrome c oxidase subunit 5b

COX6A; cytochrome c oxidase subunit 6a

COX6B; cytochrome c oxidase subunit 6b

COX7C; cytochrome c oxidase subunit 7c

ATPeV0B; V-type H+-transporting ATPase 21kDa proteolipid subunit

NDUFS1; NADH dehydrogenase (ubiquinone) Fe-S protein 1 [EC:7.1.1.2 1.6.99.3]

NDUFS2; NADH dehydrogenase (ubiquinone) Fe-S protein 2 [EC:7.1.1.2 1.6.99.3]

NDUFS3; NADH dehydrogenase (ubiquinone) Fe-S protein 3 [EC:7.1.1.2 1.6.99.3]

NDUFS4; NADH dehydrogenase (ubiquinone) Fe-S protein 4

NDUFS6; NADH dehydrogenase (ubiquinone) Fe-S protein 6

NDUFS7; NADH dehydrogenase (ubiquinone) Fe-S protein 7 [EC:7.1.1.2 1.6.99.3]

NDUFS8; NADH dehydrogenase (ubiquinone) Fe-S protein 8 [EC:7.1.1.2 1.6.99.3]

NDUFV1; NADH dehydrogenase (ubiquinone) flavoprotein 1 [EC:7.1.1.2 1.6.99.3]

NDUFV2; NADH dehydrogenase (ubiquinone) flavoprotein 2 [EC:7.1.1.2 1.6.99.3]

NDUFA2; NADH dehydrogenase (ubiquinone) 1 alpha subcomplex subunit 2

NDUFA5; NADH dehydrogenase (ubiquinone) 1 alpha subcomplex subunit 5

NDUFA6; NADH dehydrogenase (ubiquinone) 1 alpha subcomplex subunit 6

NDUFA8; NADH dehydrogenase (ubiquinone) 1 alpha subcomplex subunit 8

NDUFA9; NADH dehydrogenase (ubiquinone) 1 alpha subcomplex subunit 9

NDUFAB1; NADH dehydrogenase (ubiquinone) 1 alpha/beta subcomplex 1, acyl-carrier protein

NDUFB8; NADH dehydrogenase (ubiquinone) 1 beta subcomplex subunit 8

NDUFB9; NADH dehydrogenase (ubiquinone) 1 beta subcomplex subunit 9

NDUFA12; NADH dehydrogenase (ubiquinone) 1 alpha subcomplex subunit 12

20. Spliceosome

HSPA1s; heat shock 70kDa protein 1/2/6/8

SNW1; SNW domain-containing protein 1

SNRPB; small nuclear ribonucleoprotein B and B'

SNRPD1; small nuclear ribonucleoprotein D1

SNRPD3; small nuclear ribonucleoprotein D3

SNRPA1; U2 small nuclear ribonucleoprotein A'

SNRP70; U1 small nuclear ribonucleoprotein 70kDa

SNRPB2; U2 small nuclear ribonucleoprotein B"

SNRPE; small nuclear ribonucleoprotein E

SART1; U4/U6.U5 tri-snRNP-associated protein 1

LSM2; U6 snRNA-associated Sm-like protein LSm2

LSM4; U6 snRNA-associated Sm-like protein LSm4

PRPF4; U4/U6 small nuclear ribonucleoprotein PRP4

DDX39B; ATP-dependent RNA helicase UAP56/SUB2 [EC:3.6.4.13]

PRP2; pre-mRNA-splicing factor ATP-dependent RNA helicase-like protein PRP2 [EC:3.6.4.13]

DHX38; pre-mRNA-splicing factor ATP-dependent RNA helicase DHX38/PRP16 [EC:3.6.4.13]

CDC40; pre-mRNA-processing factor 17

PRPF18; pre-mRNA-splicing factor 18

DHX8; ATP-dependent RNA helicase DHX8/PRP22 [EC:3.6.4.13]

SLU7; pre-mRNA-processing factor SLU7

DHX15; pre-mRNA-splicing factor ATP-dependent RNA helicase DHX15/PRP43 [EC:3.6.4.13]

PRPF40; pre-mRNA-processing factor 40

DDX5; ATP-dependent RNA helicase DDX5/DBP2 [EC:3.6.4.13]

SF3A2; splicing factor 3A subunit 2

SF3A3; splicing factor 3A subunit 3

SF3B1; splicing factor 3B subunit 1

SF3B2; splicing factor 3B subunit 2

SF3B3; splicing factor 3B subunit 3

SF3B4; splicing factor 3B subunit 4

SF3B14; pre-mRNA branch site protein p14

U2AF1; splicing factor U2AF 35 kDa subunit

PRPF3; U4/U6 small nuclear ribonucleoprotein PRP3

PRPF31; U4/U6 small nuclear ribonucleoprotein PRP31

SNU13; U4/U6 small nuclear ribonucleoprotein SNU13

USP39; U4/U6.U5 tri-snRNP-associated protein 2

SNU23; U4/U6.U5 tri-snRNP component SNU23

EFTUD2; 116 kDa U5 small nuclear ribonucleoprotein component

PRPF6; pre-mRNA-processing factor 6

SNRNP40; Prp8 binding protein

DDX23; ATP-dependent RNA helicase DDX23/PRP28 [EC:3.6.4.13]

CDC5L; pre-mRNA-splicing factor CDC5/CEF1

PLRG1; pleiotropic regulator 1

CWC15; protein CWC15

SYF1; pre-mRNA-splicing factor SYF1

SYF2; pre-mRNA-splicing factor SYF2

CRN; crooked neck

ISY1; pre-mRNA-splicing factor ISY1

THOC2; THO complex subunit 2

THOC3; THO complex subunit 3

NCBP1; nuclear cap-binding protein subunit 1

NCBP2; nuclear cap-binding protein subunit 2

EIF4A3; ATP-dependent RNA helicase [EC:3.6.4.13]

21. Spinocerebellar ataxia

PIK3C3; phosphatidylinositol 3-kinase [EC:2.7.1.137]

PRKCA; classical protein kinase C alpha type [EC:2.7.11.13]

PSMA1; 20S proteasome subunit alpha 6 [EC:3.4.25.1]

PSMA3; 20S proteasome subunit alpha 7 [EC:3.4.25.1]

PSMA4; 20S proteasome subunit alpha 3 [EC:3.4.25.1]

PSMA5; 20S proteasome subunit alpha 5 [EC:3.4.25.1]

PSMA6; 20S proteasome subunit alpha 1 [EC:3.4.25.1]

PSMA7; 20S proteasome subunit alpha 4 [EC:3.4.25.1]

PSMB1; 20S proteasome subunit beta 6 [EC:3.4.25.1]

PSMB2; 20S proteasome subunit beta 4 [EC:3.4.25.1]

PSMB3; 20S proteasome subunit beta 3 [EC:3.4.25.1]

PSMB4; 20S proteasome subunit beta 7 [EC:3.4.25.1]

PSMB5; 20S proteasome subunit beta 5 [EC:3.4.25.1]

PSMB6; 20S proteasome subunit beta 1 [EC:3.4.25.1]

PSMB7; 20S proteasome subunit beta 2 [EC:3.4.25.1]

PSMD2; 26S proteasome regulatory subunit N1

PSMD4; 26S proteasome regulatory subunit N10

PSMD14; 26S proteasome regulatory subunit N11

PSMD8; 26S proteasome regulatory subunit N12

PSMD1; 26S proteasome regulatory subunit N2

PSMD3; 26S proteasome regulatory subunit N3

PSMD12; 26S proteasome regulatory subunit N5

PSMD6; 26S proteasome regulatory subunit N7

PSMD7; 26S proteasome regulatory subunit N8

PSMD13; 26S proteasome regulatory subunit N9

PSMC2; 26S proteasome regulatory subunit T1

PSMC1; 26S proteasome regulatory subunit T2

PSMC4; 26S proteasome regulatory subunit T3

PSMC6; 26S proteasome regulatory subunit T4

PSMC3; 26S proteasome regulatory subunit T5

PSMC5; 26S proteasome regulatory subunit T6

TBP; transcription initiation factor TFIID TATA-box-binding protein

TFIIB; transcription initiation factor TFIIB

RPN13; 26S proteasome regulatory subunit N13

PSMD9; 26S proteasome regulatory subunit N4

MTOR; serine/threonine-protein kinase mTOR [EC:2.7.11.1]

ULK2; serine/threonine-protein kinase ULK2 [EC:2.7.11.1]

ATG13; autophagy-related protein 13

PIK3R4; phosphoinositide-3-kinase, regulatory subunit 4 [EC:2.7.11.1]

BECN; beclin

ERN1; serine/threonine-protein kinase/endoribonuclease IRE1 [EC:2.7.11.1 3.1.26.-]

AFG3; AFG3 family protein [EC:3.4.24.-]

SHFM1; 26 proteasome complex subunit DSS1

TIP60; histone acetyltransferase HTATIP [EC:2.3.1.48]

NOP56; nucleolar protein 56

ATG2; autophagy-related protein 2

WIPI1_2; autophagy-related protein 18

ATXN10; ataxin-10

OMA1; metalloendopeptidase OMA1, mitochondrial [EC:3.4.24.-]

22. Purine metabolism

IMPDH; IMP dehydrogenase [EC:1.1.1.205]

uaZ; urate oxidase [EC:1.7.3.3]

E2.1.2.2; phosphoribosylglycinamide formyltransferase [EC:2.1.2.2]

purH; phosphoribosylaminoimidazolecarboxamide formyltransferase / IMP cyclohydrolase [EC:2.1.2.3 3.5.4.10]

APRT; adenine phosphoribosyltransferase [EC:2.4.2.7]

purF; amidophosphoribosyltransferase [EC:2.4.2.14]

E2.7.1.20; adenosine kinase [EC:2.7.1.20]

cysC; adenylylsulfate kinase [EC:2.7.1.25]

PK; pyruvate kinase [EC:2.7.1.40]

adk; adenylate kinase [EC:2.7.4.3]

ndk; nucleoside-diphosphate kinase [EC:2.7.4.6]

E2.7.4.8; guanylate kinase [EC:2.7.4.8]

PRPS; ribose-phosphate pyrophosphokinase [EC:2.7.6.1]

sat; sulfate adenylyltransferase [EC:2.7.7.4]

APA1_2; sulfate adenylyltransferase (ADP) / ATP adenylyltransferase [EC:2.7.7.5 2.7.7.53]

cpdP; 3',5'-cyclic-nucleotide phosphodiesterase [EC:3.1.4.17]

allB; allantoinase [EC:3.5.2.5]

alc; allantoicase [EC:3.5.3.4]

allA; ureidoglycolate lyase [EC:4.3.2.3]

E3.5.4.3; guanine deaminase [EC:3.5.4.3]

add; adenosine deaminase [EC:3.5.4.4]

AMPD; AMP deaminase [EC:3.5.4.6]

PRUNE; exopolyphosphatase [EC:3.6.1.11]

nudF; ADP-ribose pyrophosphatase [EC:3.6.1.13]

ITPA; inosine triphosphate pyrophosphatase [EC:3.6.1.-]

FHIT; bis(5'-adenosyl)-triphosphatase [EC:3.6.1.29]

purB; adenylosuccinate lyase [EC:4.3.2.2]

E4.6.1.1; adenylate cyclase [EC:4.6.1.1]

pgm; phosphoglucomutase [EC:5.4.2.2]

purC; phosphoribosylaminoimidazole-succinocarboxamide synthase [EC:6.3.2.6]

purA; adenylosuccinate synthase [EC:6.3.4.4]

guaA; GMP synthase (glutamine-hydrolysing) [EC:6.3.5.2]

PFAS; phosphoribosylformylglycinamidine synthase [EC:6.3.5.3]

punA; purine-nucleoside phosphorylase [EC:2.4.2.1]

surE; 5'-nucleotidase [EC:3.1.3.5]

uraH; 5-hydroxyisourate hydrolase [EC:3.5.2.17]

RRM1; ribonucleoside-diphosphate reductase subunit M1 [EC:1.17.4.1]

RRM2; ribonucleoside-diphosphate reductase subunit M2 [EC:1.17.4.1]

ADE5; phosphoribosylamine--glycine ligase / phosphoribosylformylglycinamidine cyclo-ligase [EC:6.3.4.13 6.3.3.1]

ADE2; phosphoribosylaminoimidazole carboxylase [EC:4.1.1.21]

YND1; golgi apyrase [EC:3.6.1.5]

AK6; adenylate kinase [EC:2.7.4.3]

ISN1; IMP and pyridine-specific 5'-nucleotidase [EC:3.1.3.99 3.1.3.-]

HPT1; hypoxanthine phosphoribosyltransferase [EC:2.4.2.8]

23. Cell cycle

BUB1; checkpoint serine/threonine-protein kinase [EC:2.7.11.1]

BUB3; cell cycle arrest protein BUB3

CDK7; cyclin-dependent kinase 7 [EC:2.7.11.22 2.7.11.23]

MCM5; DNA replication licensing factor MCM5 [EC:3.6.4.12]

MCM7; DNA replication licensing factor MCM7 [EC:3.6.4.12]

MCM4; DNA replication licensing factor MCM4 [EC:3.6.4.12]

CDC6; cell division control protein 6

CDC7; cell division control protein 7 [EC:2.7.11.1]

ESP1; separase [EC:3.4.22.49]

MCM2; DNA replication licensing factor MCM2 [EC:3.6.4.12]

MCM3; DNA replication licensing factor MCM3 [EC:3.6.4.12]

MCM6; DNA replication licensing factor MCM6 [EC:3.6.4.12]

ORC1; origin recognition complex subunit 1

ORC2; origin recognition complex subunit 2

ORC4; origin recognition complex subunit 4

ORC5; origin recognition complex subunit 5

ORC6; origin recognition complex subunit 6

GSK3B; glycogen synthase kinase 3 beta [EC:2.7.11.26]

SKP1; S-phase kinase-associated protein 1

CUL1; cullin 1

APC1; anaphase-promoting complex subunit 1

APC2; anaphase-promoting complex subunit 2

APC3; anaphase-promoting complex subunit 3

APC4; anaphase-promoting complex subunit 4

APC5; anaphase-promoting complex subunit 5

APC6; anaphase-promoting complex subunit 6

APC8; anaphase-promoting complex subunit 8

APC10; anaphase-promoting complex subunit 10

APC11; anaphase-promoting complex subunit 11

CDC20; cell division cycle 20, cofactor of APC complex

CDH1; cell division cycle 20-like protein 1, cofactor of APC complex

ATM; serine-protein kinase ATM [EC:2.7.11.1]

PCNA; proliferating cell nuclear antigen

HDAC1_2; histone deacetylase 1/2 [EC:3.5.1.98]

CDC45; cell division control protein 45

YWHAE; 14-3-3 protein epsilon

CCNH; cyclin H

SMC1; structural maintenance of chromosome 1

CDC14; cell division cycle 14 [EC:3.1.3.16 3.1.3.48]

SMC3; structural maintenance of chromosome 3 (chondroitin sulfate proteoglycan 6)

SCC1; cohesin complex subunit SCC1

STAG1_2; cohesin complex subunit SA-1/2

MAD1; mitotic spindle assembly checkpoint protein MAD1

TTK; serine/threonine-protein kinase TTK/MPS1 [EC:2.7.12.1]

24. Ubiquitin mediated proteolysis

UBE2R; ubiquitin-conjugating enzyme E2 R [EC:2.3.2.23]

RP-S27Ae; ubiquitin-small subunit ribosomal protein S27Ae

SKP1; S-phase kinase-associated protein 1

UBE1; ubiquitin-activating enzyme E1 [EC:6.2.1.45]

CUL1; cullin 1

APC1; anaphase-promoting complex subunit 1

APC2; anaphase-promoting complex subunit 2

APC3; anaphase-promoting complex subunit 3

APC4; anaphase-promoting complex subunit 4

APC5; anaphase-promoting complex subunit 5

APC6; anaphase-promoting complex subunit 6

APC8; anaphase-promoting complex subunit 8

APC10; anaphase-promoting complex subunit 10

APC11; anaphase-promoting complex subunit 11

GRR1; F-box and leucine-rich repeat protein GRR1

CDC4; F-box and WD-40 domain protein CDC4

CDC20; cell division cycle 20, cofactor of APC complex

CDH1; cell division cycle 20-like protein 1, cofactor of APC complex

CUL3; cullin 3

ELOC; elongin-C

UBE2J2; ubiquitin-conjugating enzyme E2 J2 [EC:2.3.2.23]

UBE2G2; ubiquitin-conjugating enzyme E2 G2 [EC:2.3.2.23]

HIP2; ubiquitin-conjugating enzyme (huntingtin interacting protein 2) [EC:2.3.2.23]

PIAS1; E3 SUMO-protein ligase PIAS1 [EC:2.3.2.-]

UBE2D; ubiquitin-conjugating enzyme E2 D [EC:2.3.2.23]

UBC; ubiquitin C

STUB1; STIP1 homology and U-box containing protein 1 [EC:2.3.2.27]

MET30; F-box and WD-40 domain protein MET30

ERCC8; DNA excision repair protein ERCC-8

UBE2A; ubiquitin-conjugating enzyme E2 A [EC:2.3.2.23]

UBE2H; ubiquitin-conjugating enzyme E2 H [EC:2.3.2.23]

UBE2I; ubiquitin-conjugating enzyme E2 I

UBE2N; ubiquitin-conjugating enzyme E2 N [EC:2.3.2.23]

UBE3C; ubiquitin-protein ligase E3 C [EC:2.3.2.26]

TRIP12; E3 ubiquitin-protein ligase TRIP12 [EC:2.3.2.26]

NEDD4; E3 ubiquitin-protein ligase NEDD4 [EC:2.3.2.26]

HUWE1; E3 ubiquitin-protein ligase HUWE1 [EC:2.3.2.26]

UBE4B; ubiquitin conjugation factor E4 B [EC:2.3.2.27]

SYVN1; E3 ubiquitin-protein ligase synoviolin [EC:2.3.2.27]

CUL4; cullin 4

UBLE1A; ubiquitin-like 1-activating enzyme E1 A [EC:6.2.1.45]

UBE1C; ubiquitin-activating enzyme E1 C [EC:6.2.1.45]

UBE2W; ubiquitin-conjugating enzyme E2 W [EC:2.3.2.25]

25. Non-alcoholic fatty liver disease (NAFLD)

SDHA; succinate dehydrogenase (ubiquinone) flavoprotein subunit [EC:1.3.5.1]

SDHB; succinate dehydrogenase (ubiquinone) iron-sulfur subunit [EC:1.3.5.1]

SDHD; succinate dehydrogenase (ubiquinone) membrane anchor subunit

UQCRFS1; ubiquinol-cytochrome c reductase iron-sulfur subunit [EC:7.1.1.8]

CYC1; ubiquinol-cytochrome c reductase cytochrome c1 subunit

QCR1; ubiquinol-cytochrome c reductase core subunit 1

QCR2; ubiquinol-cytochrome c reductase core subunit 2

QCR7; ubiquinol-cytochrome c reductase subunit 7

QCR8; ubiquinol-cytochrome c reductase subunit 8

COX5B; cytochrome c oxidase subunit 5b

COX6A; cytochrome c oxidase subunit 6a

COX6B; cytochrome c oxidase subunit 6b

COX7C; cytochrome c oxidase subunit 7c

GSK3B; glycogen synthase kinase 3 beta [EC:2.7.11.26]

EIF2S1; translation initiation factor 2 subunit 1

NDUFS1; NADH dehydrogenase (ubiquinone) Fe-S protein 1 [EC:7.1.1.2 1.6.99.3]

NDUFS2; NADH dehydrogenase (ubiquinone) Fe-S protein 2 [EC:7.1.1.2 1.6.99.3]

NDUFS3; NADH dehydrogenase (ubiquinone) Fe-S protein 3 [EC:7.1.1.2 1.6.99.3]

NDUFS4; NADH dehydrogenase (ubiquinone) Fe-S protein 4

NDUFS6; NADH dehydrogenase (ubiquinone) Fe-S protein 6

NDUFS7; NADH dehydrogenase (ubiquinone) Fe-S protein 7 [EC:7.1.1.2 1.6.99.3]

NDUFS8; NADH dehydrogenase (ubiquinone) Fe-S protein 8 [EC:7.1.1.2 1.6.99.3]

NDUFV1; NADH dehydrogenase (ubiquinone) flavoprotein 1 [EC:7.1.1.2 1.6.99.3]

NDUFV2; NADH dehydrogenase (ubiquinone) flavoprotein 2 [EC:7.1.1.2 1.6.99.3]

NDUFA2; NADH dehydrogenase (ubiquinone) 1 alpha subcomplex subunit 2

NDUFA5; NADH dehydrogenase (ubiquinone) 1 alpha subcomplex subunit 5

NDUFA6; NADH dehydrogenase (ubiquinone) 1 alpha subcomplex subunit 6

NDUFA8; NADH dehydrogenase (ubiquinone) 1 alpha subcomplex subunit 8

NDUFA9; NADH dehydrogenase (ubiquinone) 1 alpha subcomplex subunit 9

NDUFAB1; NADH dehydrogenase (ubiquinone) 1 alpha/beta subcomplex 1, acyl-carrier protein

NDUFB8; NADH dehydrogenase (ubiquinone) 1 beta subcomplex subunit 8

NDUFB9; NADH dehydrogenase (ubiquinone) 1 beta subcomplex subunit 9

RAC1; Ras-related C3 botulinum toxin substrate 1

CDC42; cell division control protein 42

PRKAB; 5'-AMP-activated protein kinase, regulatory beta subunit

PRKAG; 5'-AMP-activated protein kinase, regulatory gamma subunit

ADIPOR; adiponectin receptor

CYC; cytochrome c

ERN1; serine/threonine-protein kinase/endoribonuclease IRE1 [EC:2.7.11.1 3.1.26.-]

NDUFA12; NADH dehydrogenase (ubiquinone) 1 alpha subcomplex subunit 12

26. Shigellosis

HK; hexokinase [EC:2.7.1.1]

PIK3C3; phosphatidylinositol 3-kinase [EC:2.7.1.137]

RP-S27Ae; ubiquitin-small subunit ribosomal protein S27Ae

GSK3B; glycogen synthase kinase 3 beta [EC:2.7.11.26]

SKP1; S-phase kinase-associated protein 1

CUL1; cullin 1

ERK; mitogen-activated protein kinase 1/3 [EC:2.7.11.24]

RAC1; Ras-related C3 botulinum toxin substrate 1

CDC42; cell division control protein 42

P38; p38 MAP kinase [EC:2.7.11.24]

RHOA; Ras homolog gene family, member A

ATM; serine-protein kinase ATM [EC:2.7.11.1]

ARPC5; actin related protein 2/3 complex, subunit 5

ARPC4; actin related protein 2/3 complex, subunit 4

ARPC3; actin related protein 2/3 complex, subunit 3

ARPC1A_B; actin related protein 2/3 complex, subunit 1A/1B

ARPC2; actin related protein 2/3 complex, subunit 2

PLCD; phosphatidylinositol phospholipase C, delta [EC:3.1.4.11]

PPID; peptidyl-prolyl isomerase D [EC:5.2.1.8]

UBE2D; ubiquitin-conjugating enzyme E2 D [EC:2.3.2.23]

MTOR; serine/threonine-protein kinase mTOR [EC:2.7.11.1]

RAPTOR; regulatory associated protein of mTOR

ARF1_2; ADP-ribosylation factor 1/2

PIK3R4; phosphoinositide-3-kinase, regulatory subunit 4 [EC:2.7.11.1]

BECN; beclin

ATG5; autophagy-related protein 5

GABARAP; GABA(A) receptor-associated protein

CYC; cytochrome c

UBC; ubiquitin C

UBE2N; ubiquitin-conjugating enzyme E2 N [EC:2.3.2.23]

UBE2V; ubiquitin-conjugating enzyme E2 variant

H3; histone H3

U2AF1; splicing factor U2AF 35 kDa subunit

RRAGA_B; Ras-related GTP-binding protein A/B

RRAGC_D; Ras-related GTP-binding protein C/D

SEPT3_9_12; septin 3/9/12

SEPT7; septin 7

WIPI1_2; autophagy-related protein 18

FNBP1; formin-binding protein 1

WASL; neural Wiskott-Aldrich syndrome protein

27. RNA degradation

pfkA; 6-phosphofructokinase 1 [EC:2.7.1.11]

ENO; enolase [EC:4.2.1.11]

PAPD5_7; non-canonical poly(A) RNA polymerase PAPD5/7 [EC:2.7.7.19]

RRP45; exosome complex component RRP45

RRP40; exosome complex component RRP40

dnaK; molecular chaperone DnaK

groEL; chaperonin GroEL

CSL4; exosome complex component CSL4

CNOT4; CCR4-NOT transcription complex subunit 4 [EC:2.3.2.27]

RRP41; exosome complex component RRP41

PAN2; PAB-dependent poly(A)-specific ribonuclease subunit 2 [EC:3.1.13.4]

PAN3; PAB-dependent poly(A)-specific ribonuclease subunit 3

CNOT3; CCR4-NOT transcription complex subunit 3

CNOT7_8; CCR4-NOT transcription complex subunit 7/8

DCPS; m7GpppX diphosphatase [EC:3.6.1.59]

RRP43; exosome complex component RRP43

MTR3; exosome complex component MTR3

RRP46; exosome complex component RRP46

RRP6; exosome complex exonuclease RRP6 [EC:3.1.13.-]

C1D; exosome complex protein LRP1

MPP6; M-phase phosphoprotein 6, fungi type

AIR1_2; protein AIR1/2

MTR4; ATP-dependent RNA helicase DOB1 [EC:3.6.4.13]

SKI2; antiviral helicase SKI2 [EC:3.6.4.-]

SKI3; superkiller protein 3

CNOT6; CCR4-NOT transcription complex subunit 6 [EC:3.1.13.4]

CNOT1; CCR4-NOT transcription complex subunit 1

RCD1; CCR4-NOT transcription complex subunit 9

CAF16; CCR4-NOT complex subunit CAF16

CAF120; CCR4-NOT transcriptional complex subunit CAF120

DCP2; mRNA-decapping enzyme subunit 2 [EC:3.6.1.62]

DDX6; ATP-dependent RNA helicase DDX6/DHH1 [EC:3.6.4.13]

EDC3; enhancer of mRNA-decapping protein 3

PATL1; DNA topoisomerase 2-associated protein PAT1

XRN1; 5'-3' exoribonuclease 1 [EC:3.1.13.-]

XRN2; 5'-3' exoribonuclease 2 [EC:3.1.13.-]

LSM1; U6 snRNA-associated Sm-like protein LSm1

LSM2; U6 snRNA-associated Sm-like protein LSm2

LSM4; U6 snRNA-associated Sm-like protein LSm4

PABPC; polyadenylate-binding protein

28. Salmonella infection

GAPDH; glyceraldehyde 3-phosphate dehydrogenase [EC:1.2.1.12]

PIK3C3; phosphatidylinositol 3-kinase [EC:2.7.1.137]

RP-S3e; small subunit ribosomal protein S3e

SKP1; S-phase kinase-associated protein 1

trxA; thioredoxin 1

HSP90A; molecular chaperone HtpG

ERK; mitogen-activated protein kinase 1/3 [EC:2.7.11.24]

RAC1; Ras-related C3 botulinum toxin substrate 1

CDC42; cell division control protein 42

PAK1; p21-activated kinase 1 [EC:2.7.11.1]

P38; p38 MAP kinase [EC:2.7.11.24]

RHOA; Ras homolog gene family, member A

ARPC5; actin related protein 2/3 complex, subunit 5

ARPC4; actin related protein 2/3 complex, subunit 4

ARPC3; actin related protein 2/3 complex, subunit 3

ARPC1A_B; actin related protein 2/3 complex, subunit 1A/1B

ARPC2; actin related protein 2/3 complex, subunit 2

EXOC4; exocyst complex component 4

EXOC7; exocyst complex component 7

RAB5C; Ras-related protein Rab-5C

RAB7A; Ras-related protein Rab-7A

ARF1_2; ADP-ribosylation factor 1/2

ARF6; ADP-ribosylation factor 6

CYC; cytochrome c

M6PR; cation-dependent mannose-6-phosphate receptor

MYH; myosin heavy chain

DYNC1H; dynein heavy chain 1, cytosolic

DYNC1I; dynein intermediate chain, cytosolic

EXOC2; exocyst complex component 2

CSE1; exportin-2 (importin alpha re-exporter)

EXOC5; exocyst complex component 5

VPS11; vacuolar protein sorting-associated protein 11

VPS16; vacuolar protein sorting-associated protein 16

VPS18; vacuolar protein sorting-associated protein 18

VPS33A; vacuolar protein sorting-associated protein 33A

VPS41; vacuolar protein sorting-associated protein 41

WASL; neural Wiskott-Aldrich syndrome protein

29. mRNA surveillance pathway

RNMT; mRNA (guanine-N7-)-methyltransferase [EC:2.1.1.56]

ETF1; peptide chain release factor subunit 1

ERF3; peptide chain release factor subunit 3

PPP2R1; serine/threonine-protein phosphatase 2A regulatory subunit A

PPP2R2; serine/threonine-protein phosphatase 2A regulatory subunit B

SYMPK; symplekin

PPP1C; serine/threonine-protein phosphatase PP1 catalytic subunit [EC:3.1.3.16]

PELO; protein pelota

PPP2R5; serine/threonine-protein phosphatase 2A regulatory subunit B'

DDX39B; ATP-dependent RNA helicase UAP56/SUB2 [EC:3.6.4.13]

NCBP1; nuclear cap-binding protein subunit 1

NCBP2; nuclear cap-binding protein subunit 2

EIF4A3; ATP-dependent RNA helicase [EC:3.6.4.13]

PABPC; polyadenylate-binding protein

SRRM1; serine/arginine repetitive matrix protein 1

NXF; nuclear RNA export factor

UPF1; regulator of nonsense transcripts 1 [EC:3.6.4.-]

UPF2; regulator of nonsense transcripts 2

UPF3; regulator of nonsense transcripts 3

PAP; poly(A) polymerase [EC:2.7.7.19]

PABPN1; polyadenylate-binding protein 2

CLP1; polyribonucleotide 5'-hydroxyl-kinase [EC:2.7.1.78]

PCF11; pre-mRNA cleavage complex 2 protein Pcf11

CPSF1; cleavage and polyadenylation specificity factor subunit 1

CPSF2; cleavage and polyadenylation specificity factor subunit 2

CPSF3; cleavage and polyadenylation specificity factor subunit 3 [EC:3.1.27.-]

CPSF4; cleavage and polyadenylation specificity factor subunit 4

FIP1L1; pre-mRNA 3'-end-processing factor FIP1

CSTF2; cleavage stimulation factor subunit 2

CSTF3; cleavage stimulation factor subunit 3

MSI; RNA-binding protein Musashi

HBS1; elongation factor 1 alpha-like protein

WDR82; COMPASS component SWD2

MPE1; protein MPE1

PFS2; polyadenylation factor subunit 2

SSU72; RNA polymerase II subunit A C-terminal domain phosphatase SSU72 [EC:3.1.3.16]

30. Nucleotide excision repair

CDK7; cyclin-dependent kinase 7 [EC:2.7.11.22 2.7.11.23]

POLE; DNA polymerase epsilon subunit 1 [EC:2.7.7.7]

POLE2; DNA polymerase epsilon subunit 2 [EC:2.7.7.7]

POLE3; DNA polymerase epsilon subunit 3 [EC:2.7.7.7]

POLD1; DNA polymerase delta subunit 1 [EC:2.7.7.7]

POLD2; DNA polymerase delta subunit 2

TFIIH1; transcription initiation factor TFIIH subunit 1

TFIIH2; transcription initiation factor TFIIH subunit 2

TFIIH3; transcription initiation factor TFIIH subunit 3

TFIIH4; transcription initiation factor TFIIH subunit 4

POLD3; DNA polymerase delta subunit 3

POLE4; DNA polymerase epsilon subunit 4 [EC:2.7.7.7]

uvrD; DNA helicase II / ATP-dependent DNA helicase PcrA [EC:3.6.4.12]

PCNA; proliferating cell nuclear antigen

CCNH; cyclin H

RFA1; replication factor A1

ERCC8; DNA excision repair protein ERCC-8

CUL4; cullin 4

RFA2; replication factor A2

RPA3; replication factor A3

LIG1; DNA ligase 1 [EC:6.5.1.1 6.5.1.6 6.5.1.7]

RFC1; replication factor C subunit 1

RFC2_4; replication factor C subunit 2/4

RFC3_5; replication factor C subunit 3/5

XPC; xeroderma pigmentosum group C-complementing protein

RAD23; UV excision repair protein RAD23

ERCC6; DNA excision repair protein ERCC-6

MNAT1; CDK-activating kinase assembly factor MAT1

ERCC3; DNA excision repair protein ERCC-3 [EC:3.6.4.12]

ERCC2; DNA excision repair protein ERCC-2 [EC:3.6.4.12]

TTDA; TFIIH basal transcription factor complex TTD-A subunit

ERCC5; DNA excision repair protein ERCC-5

XPA; DNA-repair protein complementing XP-A cells

ERCC4; DNA excision repair protein ERCC-4 [EC:3.1.-.-]

ERCC1; DNA excision repair protein ERCC-1

31. Human T-cell leukemia virus 1 infection

FDPS; farnesyl diphosphate synthase [EC:2.5.1.1 2.5.1.10]

PTEN; phosphatidylinositol-3,4,5-trisphosphate 3-phosphatase and dual-specificity protein phosphatase PTEN [EC:3.1.3.16 3.1.3.48 3.1.3.67]

BUB3; cell cycle arrest protein BUB3

ESP1; separase [EC:3.4.22.49]

TBP; transcription initiation factor TFIID TATA-box-binding protein

APC1; anaphase-promoting complex subunit 1

APC2; anaphase-promoting complex subunit 2

APC3; anaphase-promoting complex subunit 3

APC4; anaphase-promoting complex subunit 4

APC5; anaphase-promoting complex subunit 5

APC6; anaphase-promoting complex subunit 6

APC8; anaphase-promoting complex subunit 8

APC10; anaphase-promoting complex subunit 10

APC11; anaphase-promoting complex subunit 11

CDC20; cell division cycle 20, cofactor of APC complex

PKA; protein kinase A [EC:2.7.11.11]

PPP3C; serine/threonine-protein phosphatase 2B catalytic subunit [EC:3.1.3.16]

ERK; mitogen-activated protein kinase 1/3 [EC:2.7.11.24]

ATM; serine-protein kinase ATM [EC:2.7.11.1]

SLC25A4S; solute carrier family 25 (mitochondrial adenine nucleotide translocator), member 4/5/6/31

PCAF; histone acetyltransferase [EC:2.3.1.48]

PPP3R; serine/threonine-protein phosphatase 2B regulatory subunit

MAD1; mitotic spindle assembly checkpoint protein MAD1

KRAS; GTPase KRas

RAN; GTP-binding nuclear protein Ran

CANX; calnexin

NFYB; nuclear transcription Y subunit beta

TRRAP; transformation/transcription domain-associated protein

TERT; telomerase reverse transcriptase [EC:2.7.7.49]

TIP60; histone acetyltransferase HTATIP [EC:2.3.1.48]

XPO1; exportin-1

VDAC2; voltage-dependent anion channel protein 2

RANBP3; Ran-binding protein 3

VAC14; vacuole morphology and inheritance protein 14

RANBP1; Ran-binding protein 1

32. mTOR signaling pathway

PTEN; phosphatidylinositol-3,4,5-trisphosphate 3-phosphatase and dual-specificity protein phosphatase PTEN [EC:3.1.3.16 3.1.3.48 3.1.3.67]

ATPeV1A; V-type H+-transporting ATPase subunit A [EC:7.1.2.2]

ATPeV1B; V-type H+-transporting ATPase subunit B

ATPeV1C; V-type H+-transporting ATPase subunit C

ATPeV1D; V-type H+-transporting ATPase subunit D

ATPeV1G; V-type H+-transporting ATPase subunit G

PRKCA; classical protein kinase C alpha type [EC:2.7.11.13]

GSK3B; glycogen synthase kinase 3 beta [EC:2.7.11.26]

SOS; son of sevenless

EIF4B; translation initiation factor 4B

EIF4E; translation initiation factor 4E

ERK; mitogen-activated protein kinase 1/3 [EC:2.7.11.24]

RHOA; Ras homolog gene family, member A

PDPK1; 3-phosphoinositide dependent protein kinase-1 [EC:2.7.11.1]

MTOR; serine/threonine-protein kinase mTOR [EC:2.7.11.1]

RAPTOR; regulatory associated protein of mTOR

RHEB; Ras homolog enriched in brain

KRAS; GTPase KRas

MLST8; target of rapamycin complex subunit LST8

RICTOR; rapamycin-insensitive companion of mTOR

ULK2; serine/threonine-protein kinase ULK2 [EC:2.7.11.1]

CAB39; calcium binding protein 39

TELO2; telomere length regulation protein

SEC13; protein transport protein SEC13

SEH1; nucleoporin SEH1

LPIN; phosphatidate phosphatase LPIN [EC:3.1.3.4]

RRAGA_B; Ras-related GTP-binding protein A/B

RRAGC_D; Ras-related GTP-binding protein C/D

TTI1; TELO2-interacting protein 1

DEPDC5; DEP domain-containing protein 5

NPRL2; nitrogen permease regulator 2-like protein

NPRL3; nitrogen permease regulator 3-like protein

MIOS; WD repeat-containing protein mio

WDR24; WD repeat-containing protein 24

WDR59; WD repeat-containing protein 59

33. Proteasome

PSMA1; 20S proteasome subunit alpha 6 [EC:3.4.25.1]

PSMA3; 20S proteasome subunit alpha 7 [EC:3.4.25.1]

PSMA4; 20S proteasome subunit alpha 3 [EC:3.4.25.1]

PSMA5; 20S proteasome subunit alpha 5 [EC:3.4.25.1]

PSMA6; 20S proteasome subunit alpha 1 [EC:3.4.25.1]

PSMA7; 20S proteasome subunit alpha 4 [EC:3.4.25.1]

PSMB1; 20S proteasome subunit beta 6 [EC:3.4.25.1]

PSMB2; 20S proteasome subunit beta 4 [EC:3.4.25.1]

PSMB3; 20S proteasome subunit beta 3 [EC:3.4.25.1]

PSMB4; 20S proteasome subunit beta 7 [EC:3.4.25.1]

PSMB5; 20S proteasome subunit beta 5 [EC:3.4.25.1]

PSMB6; 20S proteasome subunit beta 1 [EC:3.4.25.1]

PSMB7; 20S proteasome subunit beta 2 [EC:3.4.25.1]

PSMD2; 26S proteasome regulatory subunit N1

PSMD4; 26S proteasome regulatory subunit N10

PSMD14; 26S proteasome regulatory subunit N11

PSMD8; 26S proteasome regulatory subunit N12

PSMD1; 26S proteasome regulatory subunit N2

PSMD3; 26S proteasome regulatory subunit N3

PSMD12; 26S proteasome regulatory subunit N5

PSMD6; 26S proteasome regulatory subunit N7

PSMD7; 26S proteasome regulatory subunit N8

PSMD13; 26S proteasome regulatory subunit N9

PSMC2; 26S proteasome regulatory subunit T1

PSMC1; 26S proteasome regulatory subunit T2

PSMC4; 26S proteasome regulatory subunit T3

PSMC6; 26S proteasome regulatory subunit T4

PSMC3; 26S proteasome regulatory subunit T5

PSMC5; 26S proteasome regulatory subunit T6

RPN13; 26S proteasome regulatory subunit N13

PSMD9; 26S proteasome regulatory subunit N4

PSME4; proteasome activator subunit 4

SHFM1; 26 proteasome complex subunit DSS1

34. Peroxisome

IDH1; isocitrate dehydrogenase [EC:1.1.1.42]

E1.3.3.6; acyl-CoA oxidase [EC:1.3.3.6]

DAO; D-amino-acid oxidase [EC:1.4.3.3]

PIPOX; sarcosine oxidase / L-pipecolate oxidase [EC:1.5.3.1 1.5.3.7]

PHYH; phytanoyl-CoA hydroxylase [EC:1.14.11.18]

E2.3.1.7; carnitine O-acetyltransferase [EC:2.3.1.7]

AGXT; alanine-glyoxylate transaminase / serine-glyoxylate transaminase / serine-pyruvate transaminase [EC:2.6.1.44 2.6.1.45 2.6.1.51]

E2.7.1.36; mevalonate kinase [EC:2.7.1.36]

ACSL; long-chain acyl-CoA synthetase [EC:6.2.1.3]

E3.6.1.22; NAD+ diphosphatase [EC:3.6.1.22]

katE; catalase [EC:1.11.1.6]

SOD2; superoxide dismutase, Fe-Mn family [EC:1.15.1.1]

SOD1; superoxide dismutase, Cu-Zn family [EC:1.15.1.1]

PEX2; peroxin-2

ACAA1; acetyl-CoA acyltransferase 1 [EC:2.3.1.16]

HAO; (S)-2-hydroxy-acid oxidase [EC:1.1.3.15]

ECH1; Delta3,5-Delta2,4-dienoyl-CoA isomerase [EC:5.3.3.21]

DECR2; peroxisomal 2,4-dienoyl-CoA reductase [EC:1.3.1.34]

ECI2; Delta3-Delta2-enoyl-CoA isomerase [EC:5.3.3.8]

PEX3; peroxin-3

PEX19; peroxin-19

PEX1; peroxin-1

PEX6; peroxin-6

PEX7; peroxin-7

PEX5; peroxin-5

PEX14; peroxin-14

PEX13; peroxin-13

PEX12; peroxin-12

MPV17; protein Mpv17

PXMP4; peroxisomal membrane protein 4

PEX11B; peroxin-11B

SLC25A17; solute carrier family 25 (peroxisomal adenine nucleotide transporter), member 17

PXA; ATP-binding cassette, subfamily D (ALD), peroxisomal long-chain fatty acid import protein

35. Cysteine and methionine metabolism

hom; homoserine dehydrogenase [EC:1.1.1.3]

MDH2; malate dehydrogenase [EC:1.1.1.37]

serA; D-3-phosphoglycerate dehydrogenase / 2-oxoglutarate reductase [EC:1.1.1.95 1.1.1.399]

asd; aspartate-semialdehyde dehydrogenase [EC:1.2.1.11]

CDO1; cysteine dioxygenase [EC:1.13.11.20]

mmuM; homocysteine S-methyltransferase [EC:2.1.1.10]

metE; 5-methyltetrahydropteroyltriglutamate--homocysteine methyltransferase [EC:2.1.1.14]

metX; homoserine O-acetyltransferase/O-succinyltransferase [EC:2.3.1.31 2.3.1.46]

metK; S-adenosylmethionine synthetase [EC:2.5.1.6]

speE; spermidine synthase [EC:2.5.1.16]

SMS; spermine synthase [EC:2.5.1.22]

E2.6.1.42; branched-chain amino acid aminotransferase [EC:2.6.1.42]

serC; phosphoserine aminotransferase [EC:2.6.1.52]

ARO8; aromatic amino acid aminotransferase I / 2-aminoadipate transaminase [EC:2.6.1.57 2.6.1.39 2.6.1.27 2.6.1.5]

lysC; aspartate kinase [EC:2.7.2.4]

E3.3.1.1; adenosylhomocysteinase [EC:3.3.1.1]

speD; S-adenosylmethionine decarboxylase [EC:4.1.1.50]

CBS; cystathionine beta-synthase [EC:4.2.1.22]

cysK; cysteine synthase [EC:2.5.1.47]

metB; cystathionine gamma-synthase [EC:2.5.1.48]

CTH; cystathionine gamma-lyase [EC:4.4.1.1]

metC; cysteine-S-conjugate beta-lyase [EC:4.4.1.13]

mtnA; methylthioribose-1-phosphate isomerase [EC:5.3.1.23]

mtnB; methylthioribulose-1-phosphate dehydratase [EC:4.2.1.109]

mtnD; 1,2-dihydroxy-3-keto-5-methylthiopentene dioxygenase [EC:1.13.11.53 1.13.11.54]

msrC; L-methionine (R)-S-oxide reductase [EC:1.8.4.14]

mtnC; enolase-phosphatase E1 [EC:3.1.3.77]

GCLC; glutamate--cysteine ligase catalytic subunit [EC:6.3.2.2]

GOT1; aspartate aminotransferase, cytoplasmic [EC:2.6.1.1]

GOT2; aspartate aminotransferase, mitochondrial [EC:2.6.1.1]

MET17; O-acetylhomoserine/O-acetylserine sulfhydrylase [EC:2.5.1.49 2.5.1.47]

SDS; L-serine/L-threonine ammonia-lyase [EC:4.3.1.17 4.3.1.19]

GSS; glutathione synthase [EC:6.3.2.3]

36. Viral carcinogenesis

PK; pyruvate kinase [EC:2.7.1.40]

ATPeV0D; V-type H+-transporting ATPase subunit d

PSMC1; 26S proteasome regulatory subunit T2

TBP; transcription initiation factor TFIID TATA-box-binding protein

TFIIA2; transcription initiation factor TFIIA small subunit

TFIIB; transcription initiation factor TFIIB

TFIIE1; transcription initiation factor TFIIE subunit alpha

TFIIE2; transcription initiation factor TFIIE subunit beta

TFIIH1; transcription initiation factor TFIIH subunit 1

TFIIH2; transcription initiation factor TFIIH subunit 2

TFIIH3; transcription initiation factor TFIIH subunit 3

TFIIH4; transcription initiation factor TFIIH subunit 4

CDC20; cell division cycle 20, cofactor of APC complex

PKA; protein kinase A [EC:2.7.11.11]

ERK; mitogen-activated protein kinase 1/3 [EC:2.7.11.24]

RAC1; Ras-related C3 botulinum toxin substrate 1

CDC42; cell division control protein 42

RHOA; Ras homolog gene family, member A

PCAF; histone acetyltransferase [EC:2.3.1.48]

SNW1; SNW domain-containing protein 1

HDAC1_2; histone deacetylase 1/2 [EC:3.5.1.98]

YWHAE; 14-3-3 protein epsilon

MAD1; mitotic spindle assembly checkpoint protein MAD1

KRAS; GTPase KRas

H2B; histone H2B

H4; histone H4

HDAC6; histone deacetylase 6 [EC:3.5.1.98]

DDX3X; ATP-dependent RNA helicase DDX3X [EC:3.6.4.13]

USP7; ubiquitin carboxyl-terminal hydrolase 7 [EC:3.4.19.12]

VAC14; vacuole morphology and inheritance protein 14

RANBP1; Ran-binding protein 1

SND1; staphylococcal nuclease domain-containing protein 1

37. Mitophagy – yeast

PIP5K; 1-phosphatidylinositol-4-phosphate 5-kinase [EC:2.7.1.68]

POLG; DNA polymerase gamma 1 [EC:2.7.7.7]

PRKCA; classical protein kinase C alpha type [EC:2.7.11.13]

CSNK2A; casein kinase II subunit alpha [EC:2.7.11.1]

CSNK2B; casein kinase II subunit beta

P38; p38 MAP kinase [EC:2.7.11.24]

MAPK7; mitogen-activated protein kinase 7 [EC:2.7.11.24]

MTOR; serine/threonine-protein kinase mTOR [EC:2.7.11.1]

ULK2; serine/threonine-protein kinase ULK2 [EC:2.7.11.1]

MKK1_2; mitogen-activated protein kinase kinase [EC:2.7.12.2]

ATG11; autophagy-related protein 11

GABARAP; GABA(A) receptor-associated protein

YME1; ATP-dependent metalloprotease [EC:3.4.24.-]

SNRPD3; small nuclear ribonucleoprotein D3

PBS2; mitogen-activated protein kinase kinase [EC:2.7.12.2]

BCK1; mitogen-activated protein kinase kinase kinase [EC:2.7.11.25]

SLN1; osomolarity two-component system, sensor histidine kinase SLN1 [EC:2.7.13.3]

SSK1; osomolarity two-component system, response regulator SSK1

WSC; cell wall integrity and stress response component

SIN3A; paired amphipathic helix protein Sin3a

USP10; ubiquitin carboxyl-terminal hydrolase 10 [EC:3.4.19.12]

DNM1L; dynamin 1-like protein [EC:3.6.5.5]

MMM1; maintenance of mitochondrial morphology protein 1

MDM12; mitochondrial distribution and morphology protein 12

MDM10; mitochondrial distribution and morphology protein 10

MDM34; mitochondrial distribution and morphology protein 34

LETM1; LETM1 and EF-hand domain-containing protein 1, mitochondrial

FIS1; mitochondrial fission 1 protein

FMC1; ATP synthase assembly factor FMC1, mitochondrial

PTC6; protein phosphatase PTC6 [EC:3.1.3.16 3.1.3.43]

ATG33; autophagy-related protein 33

BRE5; UBP3-associated protein BRE5

38. Autophagy – animal

PIK3C3; phosphatidylinositol 3-kinase [EC:2.7.1.137]

PTEN; phosphatidylinositol-3,4,5-trisphosphate 3-phosphatase and dual-specificity protein phosphatase PTEN [EC:3.1.3.16 3.1.3.48 3.1.3.67]

EIF2S1; translation initiation factor 2 subunit 1

PKA; protein kinase A [EC:2.7.11.11]

ERK; mitogen-activated protein kinase 1/3 [EC:2.7.11.24]

PDPK1; 3-phosphoinositide dependent protein kinase-1 [EC:2.7.11.1]

MTOR; serine/threonine-protein kinase mTOR [EC:2.7.11.1]

RAPTOR; regulatory associated protein of mTOR

RHEB; Ras homolog enriched in brain

KRAS; GTPase KRas

RAB1A; Ras-related protein Rab-1A

RAB7A; Ras-related protein Rab-7A

RAB8A; Ras-related protein Rab-8A

MLST8; target of rapamycin complex subunit LST8

ULK2; serine/threonine-protein kinase ULK2 [EC:2.7.11.1]

ATG13; autophagy-related protein 13

PIK3R4; phosphoinositide-3-kinase, regulatory subunit 4 [EC:2.7.11.1]

BECN; beclin

ATG7; ubiquitin-like modifier-activating enzyme ATG7

ATG5; autophagy-related protein 5

GABARAP; GABA(A) receptor-associated protein

ATG4; cysteine protease ATG4 [EC:3.4.22.-]

ATG3; ubiquitin-like-conjugating enzyme ATG3

ERN1; serine/threonine-protein kinase/endoribonuclease IRE1 [EC:2.7.11.1 3.1.26.-]

RRAGA_B; Ras-related GTP-binding protein A/B

RRAGC_D; Ras-related GTP-binding protein C/D

EIF2AK4; eukaryotic translation initiation factor 2-alpha kinase 4 [EC:2.7.11.1]

IGBP1; immunoglobulin-binding protein 1

ATG2; autophagy-related protein 2

ATG9; autophagy-related protein 9

WIPI1_2; autophagy-related protein 18

39. Glycerophospholipid metabolism

GPD1; glycerol-3-phosphate dehydrogenase (NAD+) [EC:1.1.1.8]

glpA; glycerol-3-phosphate dehydrogenase [EC:1.1.5.3]

PEMT; phosphatidylethanolamine/phosphatidyl-N-methylethanolamine N-methyltransferase [EC:2.1.1.17 2.1.1.71]

CKI1; choline kinase [EC:2.7.1.32]

ETNK; ethanolamine kinase [EC:2.7.1.82]

PCYT2; ethanolamine-phosphate cytidylyltransferase [EC:2.7.7.14]

PCYT1; choline-phosphate cytidylyltransferase [EC:2.7.7.15]

EPT1; ethanolaminephosphotransferase [EC:2.7.8.1]

pgsA; CDP-diacylglycerol---glycerol-3-phosphate 3-phosphatidyltransferase [EC:2.7.8.5]

CDIPT; CDP-diacylglycerol--inositol 3-phosphatidyltransferase [EC:2.7.8.11]

GEP4; phosphatidylglycerophosphatase GEP4 [EC:3.1.3.27]

PLD1_2; phospholipase D1/2 [EC:3.1.4.4]

psd; phosphatidylserine decarboxylase [EC:4.1.1.65]

AYR1; 1-acylglycerone phosphate reductase [EC:1.1.1.101]

CRLS; cardiolipin synthase (CMP-forming) [EC:2.7.8.41]

PLB; lysophospholipase [EC:3.1.1.5]

GAT; glycerol-3-phosphate O-acyltransferase / dihydroxyacetone phosphate acyltransferase [EC:2.3.1.15 2.3.1.42]

AGPAT1_2; lysophosphatidate acyltransferase [EC:2.3.1.51]

TAZ; monolysocardiolipin acyltransferase [EC:2.3.1.-]

LPT1; lysophospholipid acyltransferase [EC:2.3.1.51 2.3.1.23 2.3.1.-]

CLD1; cardiolipin-specific phospholipase [EC:3.1.1.-]

BTA1; betaine lipid synthase

TGL4; TAG lipase / steryl ester hydrolase / phospholipase A2 / LPA acyltransferase [EC:3.1.1.3 3.1.1.13 3.1.1.4 2.3.1.51]

NTE; lysophospholipid hydrolase [EC:3.1.1.5]

LPIN; phosphatidate phosphatase LPIN [EC:3.1.3.4]

CHO2; phosphatidylethanolamine N-methyltransferase [EC:2.1.1.17]

CHO1; CDP-diacylglycerol---serine O-phosphatidyltransferase [EC:2.7.8.8]

DPP1; diacylglycerol diphosphate phosphatase / phosphatidate phosphatase [EC:3.1.3.81 3.1.3.4]

PGC1; phosphatidylglycerol phospholipase C [EC:3.1.4.-]

GDE1; glycerophosphodiester phosphodiesterase [EC:3.1.4.46]

LOA1; 1-acylglycerol-3-phosphate O-acyltransferase [EC:2.3.1.51]

40. Basal transcription factors

CDK7; cyclin-dependent kinase 7 [EC:2.7.11.22 2.7.11.23]

TBP; transcription initiation factor TFIID TATA-box-binding protein

TFIIA2; transcription initiation factor TFIIA small subunit

TFIIB; transcription initiation factor TFIIB

TAF12; transcription initiation factor TFIID subunit 12

TAF13; transcription initiation factor TFIID subunit 13

TAF2; transcription initiation factor TFIID subunit 2

TAF5; transcription initiation factor TFIID subunit 5

TAF6; transcription initiation factor TFIID subunit 6

TAF7; transcription initiation factor TFIID subunit 7

TAF9B; transcription initiation factor TFIID subunit 9B

TAF10; transcription initiation factor TFIID subunit 10

TAF11; transcription initiation factor TFIID subunit 11

TFIIE1; transcription initiation factor TFIIE subunit alpha

TFIIE2; transcription initiation factor TFIIE subunit beta

TFIIF1; transcription initiation factor TFIIF subunit alpha

TFIIF2; transcription initiation factor TFIIF subunit beta [EC:3.6.4.12]

TAF14; transcription initiation factor TFIID/TFIIF subunit

TFIIH1; transcription initiation factor TFIIH subunit 1

TFIIH2; transcription initiation factor TFIIH subunit 2

TFIIH3; transcription initiation factor TFIIH subunit 3

TFIIH4; transcription initiation factor TFIIH subunit 4

CCNH; cyclin H

MNAT1; CDK-activating kinase assembly factor MAT1

ERCC3; DNA excision repair protein ERCC-3 [EC:3.6.4.12]

ERCC2; DNA excision repair protein ERCC-2 [EC:3.6.4.12]

TTDA; TFIIH basal transcription factor complex TTD-A subunit

TAF8; transcription initiation factor TFIID subunit 8

TAF3; transcription initiation factor TFIID subunit 3

TAF15; transcription initiation factor TFIID subunit 15

TAF1; transcription initiation factor TFIID subunit 1, fungi type [EC:2.3.1.48]

41. Pathways in cancer

GST; glutathione S-transferase [EC:2.5.1.18]

PTEN; phosphatidylinositol-3,4,5-trisphosphate 3-phosphatase and dual-specificity protein phosphatase PTEN [EC:3.1.3.16 3.1.3.48 3.1.3.67]

PLD1_2; phospholipase D1/2 [EC:3.1.4.4]

E4.2.1.2B; fumarate hydratase, class II [EC:4.2.1.2]

CALM; calmodulin

CKS1; cyclin-dependent kinase regulatory subunit CKS1

PRKCA; classical protein kinase C alpha type [EC:2.7.11.13]

GSK3B; glycogen synthase kinase 3 beta [EC:2.7.11.26]

SKP1; S-phase kinase-associated protein 1

SOS; son of sevenless

CUL1; cullin 1

ELOC; elongin-C

HSP90A; molecular chaperone HtpG

PKA; protein kinase A [EC:2.7.11.11]

ERK; mitogen-activated protein kinase 1/3 [EC:2.7.11.24]

RAC1; Ras-related C3 botulinum toxin substrate 1

CDC42; cell division control protein 42

RAD51; DNA repair protein RAD51

RHOA; Ras homolog gene family, member A

GNB1; guanine nucleotide-binding protein G(I)/G(S)/G(T) subunit beta-1

GNAI; guanine nucleotide-binding protein G(i) subunit alpha

HDAC1_2; histone deacetylase 1/2 [EC:3.5.1.98]

MTOR; serine/threonine-protein kinase mTOR [EC:2.7.11.1]

KRAS; GTPase KRas

MLH1; DNA mismatch repair protein MLH1

MSH2; DNA mismatch repair protein MSH2

MSH3; DNA mismatch repair protein MSH3

MSH6; DNA mismatch repair protein MSH6

CYC; cytochrome c

TPR; nucleoprotein TPR

TERT; telomerase reverse transcriptase [EC:2.7.7.49]

42. Human papillomavirus infection

PK; pyruvate kinase [EC:2.7.1.40]

PTEN; phosphatidylinositol-3,4,5-trisphosphate 3-phosphatase and dual-specificity protein phosphatase PTEN [EC:3.1.3.16 3.1.3.48 3.1.3.67]

ATPeV1A; V-type H+-transporting ATPase subunit A [EC:7.1.2.2]

ATPeV0D; V-type H+-transporting ATPase subunit d

ATPeV1B; V-type H+-transporting ATPase subunit B

ATPeV1C; V-type H+-transporting ATPase subunit C

ATPeV1D; V-type H+-transporting ATPase subunit D

ATPeV1G; V-type H+-transporting ATPase subunit G

ATPeV0A; V-type H+-transporting ATPase subunit a

ATPeV0C; V-type H+-transporting ATPase 16kDa proteolipid subunit

PSMC1; 26S proteasome regulatory subunit T2

GSK3B; glycogen synthase kinase 3 beta [EC:2.7.11.26]

SOS; son of sevenless

TBP; transcription initiation factor TFIID TATA-box-binding protein

PPP2R1; serine/threonine-protein phosphatase 2A regulatory subunit A

ATPeV0B; V-type H+-transporting ATPase 21kDa proteolipid subunit

PKA; protein kinase A [EC:2.7.11.11]

PPP2R2; serine/threonine-protein phosphatase 2A regulatory subunit B

ERK; mitogen-activated protein kinase 1/3 [EC:2.7.11.24]

CDC42; cell division control protein 42

ATM; serine-protein kinase ATM [EC:2.7.11.1]

HDAC1_2; histone deacetylase 1/2 [EC:3.5.1.98]

MTOR; serine/threonine-protein kinase mTOR [EC:2.7.11.1]

RHEB; Ras homolog enriched in brain

KRAS; GTPase KRas

TUBG; tubulin gamma

TERT; telomerase reverse transcriptase [EC:2.7.7.49]

PPP2R5; serine/threonine-protein phosphatase 2A regulatory subunit B'

NFX1; transcriptional repressor NF-X1

BCAP31; B-cell receptor-associated protein 31

43. DNA replication

MCM5; DNA replication licensing factor MCM5 [EC:3.6.4.12]

MCM7; DNA replication licensing factor MCM7 [EC:3.6.4.12]

MCM4; DNA replication licensing factor MCM4 [EC:3.6.4.12]

POLA1; DNA polymerase alpha subunit A [EC:2.7.7.7]

POLE; DNA polymerase epsilon subunit 1 [EC:2.7.7.7]

POLE2; DNA polymerase epsilon subunit 2 [EC:2.7.7.7]

POLE3; DNA polymerase epsilon subunit 3 [EC:2.7.7.7]

POLD1; DNA polymerase delta subunit 1 [EC:2.7.7.7]

POLD2; DNA polymerase delta subunit 2

MCM2; DNA replication licensing factor MCM2 [EC:3.6.4.12]

MCM3; DNA replication licensing factor MCM3 [EC:3.6.4.12]

MCM6; DNA replication licensing factor MCM6 [EC:3.6.4.12]

PRI1; DNA primase small subunit [EC:2.7.7.102]

PRI2; DNA primase large subunit

rnhA; ribonuclease HI [EC:3.1.26.4]

POLD3; DNA polymerase delta subunit 3

POLE4; DNA polymerase epsilon subunit 4 [EC:2.7.7.7]

FEN1; flap endonuclease-1 [EC:3.-.-.-]

PCNA; proliferating cell nuclear antigen

RFA1; replication factor A1

RFA2; replication factor A2

RPA3; replication factor A3

DNA2; DNA replication ATP-dependent helicase Dna2 [EC:3.6.4.12]

RNASEH2A; ribonuclease H2 subunit A [EC:3.1.26.4]

RNASEH2B; ribonuclease H2 subunit B

LIG1; DNA ligase 1 [EC:6.5.1.1 6.5.1.6 6.5.1.7]

RFC1; replication factor C subunit 1

RFC2_4; replication factor C subunit 2/4

RFC3_5; replication factor C subunit 3/5

44. Glycine, serine and threonine metabolism

hom; homoserine dehydrogenase [EC:1.1.1.3]

serA; D-3-phosphoglycerate dehydrogenase / 2-oxoglutarate reductase [EC:1.1.1.95 1.1.1.399]

asd; aspartate-semialdehyde dehydrogenase [EC:1.2.1.11]

DAO; D-amino-acid oxidase [EC:1.4.3.3]

AOC3; primary-amine oxidase [EC:1.4.3.21]

GLDC; glycine dehydrogenase [EC:1.4.4.2]

PIPOX; sarcosine oxidase / L-pipecolate oxidase [EC:1.5.3.1 1.5.3.7]

DLD; dihydrolipoamide dehydrogenase [EC:1.8.1.4]

glyA; glycine hydroxymethyltransferase [EC:2.1.2.1]

gcvT; aminomethyltransferase [EC:2.1.2.10]

E2.3.1.37; 5-aminolevulinate synthase [EC:2.3.1.37]

AGXT; alanine-glyoxylate transaminase / serine-glyoxylate transaminase / serine-pyruvate transaminase [EC:2.6.1.44 2.6.1.45 2.6.1.51]

serC; phosphoserine aminotransferase [EC:2.6.1.52]

thrB; homoserine kinase [EC:2.7.1.39]

lysC; aspartate kinase [EC:2.7.2.4]

serB; phosphoserine phosphatase [EC:3.1.3.3]

ltaE; threonine aldolase [EC:4.1.2.48]

TRP; tryptophan synthase [EC:4.2.1.20]

CBS; cystathionine beta-synthase [EC:4.2.1.22]

thrC; threonine synthase [EC:4.2.3.1]

E4.3.1.19; threonine dehydratase [EC:4.3.1.19]

CTH; cystathionine gamma-lyase [EC:4.4.1.1]

PGAM; 2,3-bisphosphoglycerate-dependent phosphoglycerate mutase [EC:5.4.2.11]

gcvH; glycine cleavage system H protein

GLYK; D-glycerate 3-kinase [EC:2.7.1.31]

ydfG; 3-hydroxy acid dehydrogenase / malonic semialdehyde reductase [EC:1.1.1.381 1.1.1.-]

CHO1; CDP-diacylglycerol---serine O-phosphatidyltransferase [EC:2.7.8.8]

SDS; L-serine/L-threonine ammonia-lyase [EC:4.3.1.17 4.3.1.19]

DSD1; D-serine ammonia-lyase [EC:4.3.1.18]

45. 2-Oxocarboxylic acid metabolism

IDH3; isocitrate dehydrogenase (NAD+) [EC:1.1.1.41]

IDH1; isocitrate dehydrogenase [EC:1.1.1.42]

leuB; 3-isopropylmalate dehydrogenase [EC:1.1.1.85]

ilvC; ketol-acid reductoisomerase [EC:1.1.1.86]

asd; aspartate-semialdehyde dehydrogenase [EC:1.2.1.11]

ARG2; amino-acid N-acetyltransferase [EC:2.3.1.1]

argJ; glutamate N-acetyltransferase / amino-acid N-acetyltransferase [EC:2.3.1.35 2.3.1.1]

GPT; alanine transaminase [EC:2.6.1.2]

E2.6.1.11; acetylornithine aminotransferase [EC:2.6.1.11]

E2.6.1.42; branched-chain amino acid aminotransferase [EC:2.6.1.42]

ARO8; aromatic amino acid aminotransferase I / 2-aminoadipate transaminase [EC:2.6.1.57 2.6.1.39 2.6.1.27 2.6.1.5]

lysC; aspartate kinase [EC:2.7.2.4]

argE; acetylornithine deacetylase [EC:3.5.1.16]

CS; citrate synthase [EC:2.3.3.1]

leuA; 2-isopropylmalate synthase [EC:2.3.3.13]

E2.2.1.6L; acetolactate synthase I/II/III large subunit [EC:2.2.1.6]

E2.2.1.6S; acetolactate synthase I/III small subunit [EC:2.2.1.6]

LYS21; homocitrate synthase [EC:2.3.3.14]

ACO; aconitate hydratase [EC:4.2.1.3]

ilvD; dihydroxy-acid dehydratase [EC:4.2.1.9]

LEU1; 3-isopropylmalate dehydratase [EC:4.2.1.33]

LYS4; homoaconitate hydratase [EC:4.2.1.36]

LYS12; homoisocitrate dehydrogenase [EC:1.1.1.87]

ARG56; N-acetyl-gamma-glutamyl-phosphate reductase / acetylglutamate kinase [EC:1.2.1.38 2.7.2.8]

GOT1; aspartate aminotransferase, cytoplasmic [EC:2.6.1.1]

GOT2; aspartate aminotransferase, mitochondrial [EC:2.6.1.1]

ACO2; homoaconitase [EC:4.2.1.-]

46. Oocyte meiosis

BUB1; checkpoint serine/threonine-protein kinase [EC:2.7.11.1]

CALM; calmodulin

ESP1; separase [EC:3.4.22.49]

SKP1; S-phase kinase-associated protein 1

CUL1; cullin 1

APC1; anaphase-promoting complex subunit 1

APC2; anaphase-promoting complex subunit 2

APC3; anaphase-promoting complex subunit 3

APC4; anaphase-promoting complex subunit 4

APC5; anaphase-promoting complex subunit 5

APC6; anaphase-promoting complex subunit 6

APC8; anaphase-promoting complex subunit 8

APC10; anaphase-promoting complex subunit 10

APC11; anaphase-promoting complex subunit 11

CDC20; cell division cycle 20, cofactor of APC complex

PPP2R1; serine/threonine-protein phosphatase 2A regulatory subunit A

PKA; protein kinase A [EC:2.7.11.11]

PPP3C; serine/threonine-protein phosphatase 2B catalytic subunit [EC:3.1.3.16]

ERK; mitogen-activated protein kinase 1/3 [EC:2.7.11.24]

P38; p38 MAP kinase [EC:2.7.11.24]

PPP3R; serine/threonine-protein phosphatase 2B regulatory subunit

PPP1C; serine/threonine-protein phosphatase PP1 catalytic subunit [EC:3.1.3.16]

YWHAE; 14-3-3 protein epsilon

SMC1; structural maintenance of chromosome 1

SMC3; structural maintenance of chromosome 3 (chondroitin sulfate proteoglycan 6)

MAD1; mitotic spindle assembly checkpoint protein MAD1

PPP2R5; serine/threonine-protein phosphatase 2A regulatory subunit B'

47. Epstein-Barr virus infection

PSMD2; 26S proteasome regulatory subunit N1

PSMD4; 26S proteasome regulatory subunit N10

PSMD14; 26S proteasome regulatory subunit N11

PSMD8; 26S proteasome regulatory subunit N12

PSMD1; 26S proteasome regulatory subunit N2

PSMD3; 26S proteasome regulatory subunit N3

PSMD12; 26S proteasome regulatory subunit N5

PSMD6; 26S proteasome regulatory subunit N7

PSMD7; 26S proteasome regulatory subunit N8

PSMD13; 26S proteasome regulatory subunit N9

PSMC2; 26S proteasome regulatory subunit T1

PSMC1; 26S proteasome regulatory subunit T2

PSMC4; 26S proteasome regulatory subunit T3

PSMC6; 26S proteasome regulatory subunit T4

PSMC3; 26S proteasome regulatory subunit T5

PSMC5; 26S proteasome regulatory subunit T6

RAC1; Ras-related C3 botulinum toxin substrate 1

P38; p38 MAP kinase [EC:2.7.11.24]

SNW1; SNW domain-containing protein 1

HDAC1_2; histone deacetylase 1/2 [EC:3.5.1.98]

RPN13; 26S proteasome regulatory subunit N13

CYC; cytochrome c

NEDD4; E3 ubiquitin-protein ligase NEDD4 [EC:2.3.2.26]

SHFM1; 26 proteasome complex subunit DSS1

SIN3A; paired amphipathic helix protein Sin3a

USP7; ubiquitin carboxyl-terminal hydrolase 7 [EC:3.4.19.12]

SAP30; histone deacetylase complex subunit SAP30

48. Retrograde endocannabinoid signalling

MGLL; acylglycerol lipase [EC:3.1.1.23]

PRKCA; classical protein kinase C alpha type [EC:2.7.11.13]

NDUFS1; NADH dehydrogenase (ubiquinone) Fe-S protein 1 [EC:7.1.1.2 1.6.99.3]

NDUFS2; NADH dehydrogenase (ubiquinone) Fe-S protein 2 [EC:7.1.1.2 1.6.99.3]

NDUFS3; NADH dehydrogenase (ubiquinone) Fe-S protein 3 [EC:7.1.1.2 1.6.99.3]

NDUFS4; NADH dehydrogenase (ubiquinone) Fe-S protein 4

NDUFS6; NADH dehydrogenase (ubiquinone) Fe-S protein 6

NDUFS7; NADH dehydrogenase (ubiquinone) Fe-S protein 7 [EC:7.1.1.2 1.6.99.3]

NDUFS8; NADH dehydrogenase (ubiquinone) Fe-S protein 8 [EC:7.1.1.2 1.6.99.3]

NDUFV1; NADH dehydrogenase (ubiquinone) flavoprotein 1 [EC:7.1.1.2 1.6.99.3]

NDUFV2; NADH dehydrogenase (ubiquinone) flavoprotein 2 [EC:7.1.1.2 1.6.99.3]

NDUFA2; NADH dehydrogenase (ubiquinone) 1 alpha subcomplex subunit 2

NDUFA5; NADH dehydrogenase (ubiquinone) 1 alpha subcomplex subunit 5

NDUFA6; NADH dehydrogenase (ubiquinone) 1 alpha subcomplex subunit 6

NDUFA8; NADH dehydrogenase (ubiquinone) 1 alpha subcomplex subunit 8

NDUFA9; NADH dehydrogenase (ubiquinone) 1 alpha subcomplex subunit 9

NDUFAB1; NADH dehydrogenase (ubiquinone) 1 alpha/beta subcomplex 1, acyl-carrier protein

NDUFB8; NADH dehydrogenase (ubiquinone) 1 beta subcomplex subunit 8

NDUFB9; NADH dehydrogenase (ubiquinone) 1 beta subcomplex subunit 9

PKA; protein kinase A [EC:2.7.11.11]

ERK; mitogen-activated protein kinase 1/3 [EC:2.7.11.24]

P38; p38 MAP kinase [EC:2.7.11.24]

GNB1; guanine nucleotide-binding protein G(I)/G(S)/G(T) subunit beta-1

GNAI; guanine nucleotide-binding protein G(i) subunit alpha

NDUFA12; NADH dehydrogenase (ubiquinone) 1 alpha subcomplex subunit 12

NAPEPLD; N-acyl-phosphatidylethanolamine-hydrolysing phospholipase D [EC:3.1.4.54]

SLC32A; solute carrier family 32 (vesicular inhibitory amino acid transporter)

49. Aminoacyl-tRNA biosynthesis

MTFMT; methionyl-tRNA formyltransferase [EC:2.1.2.9]

YARS; tyrosyl-tRNA synthetase [EC:6.1.1.1]

WARS; tryptophanyl-tRNA synthetase [EC:6.1.1.2]

TARS; threonyl-tRNA synthetase [EC:6.1.1.3]

LARS; leucyl-tRNA synthetase [EC:6.1.1.4]

IARS; isoleucyl-tRNA synthetase [EC:6.1.1.5]

AARS; alanyl-tRNA synthetase [EC:6.1.1.7]

VARS; valyl-tRNA synthetase [EC:6.1.1.9]

MARS; methionyl-tRNA synthetase [EC:6.1.1.10]

SARS; seryl-tRNA synthetase [EC:6.1.1.11]

aspS; aspartyl-tRNA synthetase [EC:6.1.1.12]

GARS; glycyl-tRNA synthetase [EC:6.1.1.14]

PARS; prolyl-tRNA synthetase [EC:6.1.1.15]

CARS; cysteinyl-tRNA synthetase [EC:6.1.1.16]

EARS; glutamyl-tRNA synthetase [EC:6.1.1.17]

QARS; glutaminyl-tRNA synthetase [EC:6.1.1.18]

RARS; arginyl-tRNA synthetase [EC:6.1.1.19]

FARSA; phenylalanyl-tRNA synthetase alpha chain [EC:6.1.1.20]

FARSB; phenylalanyl-tRNA synthetase beta chain [EC:6.1.1.20]

HARS; histidyl-tRNA synthetase [EC:6.1.1.21]

NARS; asparaginyl-tRNA synthetase [EC:6.1.1.22]

gatA; aspartyl-tRNA(Asn)/glutamyl-tRNA(Gln) amidotransferase subunit A [EC:6.3.5.6 6.3.5.7]

gatB; aspartyl-tRNA(Asn)/glutamyl-tRNA(Gln) amidotransferase subunit B [EC:6.3.5.6 6.3.5.7]

GTF1; glutamyl-tRNA(Gln) amidotransferase subunit F [EC:6.3.5.7]

KARS; lysyl-tRNA synthetase, class II [EC:6.1.1.6]

DARS; aspartyl-tRNA synthetase [EC:6.1.1.12]

50. Glycolysis / Gluconeogenesis

AKR1A1; alcohol dehydrogenase (NADP+) [EC:1.1.1.2]

frmA; S-(hydroxymethyl)glutathione dehydrogenase / alcohol dehydrogenase [EC:1.1.1.284 1.1.1.1]

ALDH; aldehyde dehydrogenase (NAD+) [EC:1.2.1.3]

E1.2.1.5; aldehyde dehydrogenase (NAD(P)+) [EC:1.2.1.5]

GAPDH; glyceraldehyde 3-phosphate dehydrogenase [EC:1.2.1.12]

PDHA; pyruvate dehydrogenase E1 component alpha subunit [EC:1.2.4.1]

PDHB; pyruvate dehydrogenase E1 component beta subunit [EC:1.2.4.1]

DLD; dihydrolipoamide dehydrogenase [EC:1.8.1.4]

DLAT; pyruvate dehydrogenase E2 component (dihydrolipoamide acetyltransferase) [EC:2.3.1.12]

HK; hexokinase [EC:2.7.1.1]

pfkA; 6-phosphofructokinase 1 [EC:2.7.1.11]

PK; pyruvate kinase [EC:2.7.1.40]

PGK; phosphoglycerate kinase [EC:2.7.2.3]

PDC; pyruvate decarboxylase [EC:4.1.1.1]

E4.1.1.49; phosphoenolpyruvate carboxykinase (ATP) [EC:4.1.1.49]

FBA; fructose-bisphosphate aldolase, class II [EC:4.1.2.13]

ENO; enolase [EC:4.2.1.11]

galM; aldose 1-epimerase [EC:5.1.3.3]

E5.1.3.15; glucose-6-phosphate 1-epimerase [EC:5.1.3.15]

TPI; triosephosphate isomerase (TIM) [EC:5.3.1.1]

GPI; glucose-6-phosphate isomerase [EC:5.3.1.9]

PGAM; 2,3-bisphosphoglycerate-dependent phosphoglycerate mutase [EC:5.4.2.11]

pgm; phosphoglucomutase [EC:5.4.2.2]

ACSS1_2; acetyl-CoA synthetase [EC:6.2.1.1]

FBP; fructose-1,6-bisphosphatase I [EC:3.1.3.11]

adhP; alcohol dehydrogenase, propanol-preferring [EC:1.1.1.1]

51. Pyruvate metabolism

MDH2; malate dehydrogenase [EC:1.1.1.37]

ME2; malate dehydrogenase (oxaloacetate-decarboxylating) [EC:1.1.1.38]

lldD; L-lactate dehydrogenase (cytochrome) [EC:1.1.2.3]

LDHD; D-lactate dehydrogenase (cytochrome) [EC:1.1.2.4]

ALDH; aldehyde dehydrogenase (NAD+) [EC:1.2.1.3]

PDHA; pyruvate dehydrogenase E1 component alpha subunit [EC:1.2.4.1]

PDHB; pyruvate dehydrogenase E1 component beta subunit [EC:1.2.4.1]

DLD; dihydrolipoamide dehydrogenase [EC:1.8.1.4]

ACAT; acetyl-CoA C-acetyltransferase [EC:2.3.1.9]

DLAT; pyruvate dehydrogenase E2 component (dihydrolipoamide acetyltransferase) [EC:2.3.1.12]

PK; pyruvate kinase [EC:2.7.1.40]

E3.1.2.1; acetyl-CoA hydrolase [EC:3.1.2.1]

gloB; hydroxyacylglutathione hydrolase [EC:3.1.2.6]

E4.1.1.49; phosphoenolpyruvate carboxykinase (ATP) [EC:4.1.1.49]

aceB; malate synthase [EC:2.3.3.9]

leuA; 2-isopropylmalate synthase [EC:2.3.3.13]

LYS21; homocitrate synthase [EC:2.3.3.14]

E4.2.1.2B; fumarate hydratase, class II [EC:4.2.1.2]

GLO1; lactoylglutathione lyase [EC:4.4.1.5]

ACSS1_2; acetyl-CoA synthetase [EC:6.2.1.1]

PC; pyruvate carboxylase [EC:6.4.1.1]

ACACA; acetyl-CoA carboxylase / biotin carboxylase 1 [EC:6.4.1.2 6.3.4.14 2.1.3.15]

GRE2; NADPH-dependent methylglyoxal reductase [EC:1.1.1.283]

DLD3; (R)-2-hydroxyglutarate---pyruvate transhydrogenase [EC:1.1.99.40]

GLX3; D-lactate dehydratase [EC:4.2.1.130]

52. Pathogenic Escherichia coli infection

GAPDH; glyceraldehyde 3-phosphate dehydrogenase [EC:1.2.1.12]

RP-S3e; small subunit ribosomal protein S3e

ERK; mitogen-activated protein kinase 1/3 [EC:2.7.11.24]

RAC1; Ras-related C3 botulinum toxin substrate 1

CDC42; cell division control protein 42

PAK1; p21-activated kinase 1 [EC:2.7.11.1]

P38; p38 MAP kinase [EC:2.7.11.24]

RHOA; Ras homolog gene family, member A

ARPC5; actin related protein 2/3 complex, subunit 5

ARPC4; actin related protein 2/3 complex, subunit 4

ARPC3; actin related protein 2/3 complex, subunit 3

ARPC1A_B; actin related protein 2/3 complex, subunit 1A/1B

ARPC2; actin related protein 2/3 complex, subunit 2

ABCF2; ATP-binding cassette, subfamily F, member 2

RAB1A; Ras-related protein Rab-1A

ARF1_2; ADP-ribosylation factor 1/2

ARF6; ADP-ribosylation factor 6

CYC; cytochrome c

MYH; myosin heavy chain

MYO1; myosin I

MYO5; myosin V

NCL; nucleolin

SEC24; protein transport protein SEC24

TMED10; p24 family protein delta-1

WASL; neural Wiskott-Aldrich syndrome protein

53. RNA polymerase

RPA1; DNA-directed RNA polymerase I subunit RPA1 [EC:2.7.7.6]

RPA12; DNA-directed RNA polymerase I subunit RPA12

RPA2; DNA-directed RNA polymerase I subunit RPA2 [EC:2.7.7.6]

RPA34; DNA-directed RNA polymerase I subunit RPA34

RPA43; DNA-directed RNA polymerase I subunit RPA43

RPA49; DNA-directed RNA polymerase I subunit RPA49

RPB1; DNA-directed RNA polymerase II subunit RPB1 [EC:2.7.7.6]

RPB10; DNA-directed RNA polymerases I, II, and III subunit RPABC5

RPB2; DNA-directed RNA polymerase II subunit RPB2 [EC:2.7.7.6]

RPB3; DNA-directed RNA polymerase II subunit RPB3

RPB5; DNA-directed RNA polymerases I, II, and III subunit RPABC1

RPB6; DNA-directed RNA polymerases I, II, and III subunit RPABC2

RPB7; DNA-directed RNA polymerase II subunit RPB7

RPB8; DNA-directed RNA polymerases I, II, and III subunit RPABC3

RPC1; DNA-directed RNA polymerase III subunit RPC1 [EC:2.7.7.6]

RPC19; DNA-directed RNA polymerases I and III subunit RPAC2

RPC2; DNA-directed RNA polymerase III subunit RPC2 [EC:2.7.7.6]

RPC8; DNA-directed RNA polymerase III subunit RPC8

RPC3; DNA-directed RNA polymerase III subunit RPC3

RPC7; DNA-directed RNA polymerase III subunit RPC7

RPC6; DNA-directed RNA polymerase III subunit RPC6

RPC4; DNA-directed RNA polymerase III subunit RPC4

RPC40; DNA-directed RNA polymerases I and III subunit RPAC1

RPC5; DNA-directed RNA polymerase III subunit RPC5

54. Pyrimidine metabolism

DHODH; dihydroorotate dehydrogenase [EC:1.3.5.2]

thyA; thymidylate synthase [EC:2.1.1.45]

upp; uracil phosphoribosyltransferase [EC:2.4.2.9]

pyrE; orotate phosphoribosyltransferase [EC:2.4.2.10]

udk; uridine kinase [EC:2.7.1.48]

ndk; nucleoside-diphosphate kinase [EC:2.7.4.6]

tmk; dTMP kinase [EC:2.7.4.9]

URH1; uridine nucleosidase [EC:3.2.2.3]

URA4; dihydroorotase [EC:3.5.2.3]

comEB; dCMP deaminase [EC:3.5.4.12]

dut; dUTP pyrophosphatase [EC:3.6.1.23]

pyrF; orotidine-5'-phosphate decarboxylase [EC:4.1.1.23]

pyrG; CTP synthase [EC:6.3.4.2]

carA; carbamoyl-phosphate synthase small subunit [EC:6.3.5.5]

punA; purine-nucleoside phosphorylase [EC:2.4.2.1]

surE; 5'-nucleotidase [EC:3.1.3.5]

RRM1; ribonucleoside-diphosphate reductase subunit M1 [EC:1.17.4.1]

RRM2; ribonucleoside-diphosphate reductase subunit M2 [EC:1.17.4.1]

URA2; carbamoyl-phosphate synthase / aspartate carbamoyltransferase [EC:6.3.5.5 2.1.3.2]

CMPK1; UMP-CMP kinase [EC:2.7.4.14]

YND1; golgi apyrase [EC:3.6.1.5]

ydfG; 3-hydroxy acid dehydrogenase / malonic semialdehyde reductase [EC:1.1.1.381 1.1.1.-]

psuG; pseudouridylate synthase [EC:4.2.1.70]

55. PI3K-Akt signaling pathway

GYS; glycogen synthase [EC:2.4.1.11]

PTEN; phosphatidylinositol-3,4,5-trisphosphate 3-phosphatase and dual-specificity protein phosphatase PTEN [EC:3.1.3.16 3.1.3.48 3.1.3.67]

PRKCA; classical protein kinase C alpha type [EC:2.7.11.13]

GSK3B; glycogen synthase kinase 3 beta [EC:2.7.11.26]

SOS; son of sevenless

EIF4B; translation initiation factor 4B

EIF4E; translation initiation factor 4E

PPP2R1; serine/threonine-protein phosphatase 2A regulatory subunit A

HSP90A; molecular chaperone HtpG

PPP2R2; serine/threonine-protein phosphatase 2A regulatory subunit B

ERK; mitogen-activated protein kinase 1/3 [EC:2.7.11.24]

RAC1; Ras-related C3 botulinum toxin substrate 1

GNB1; guanine nucleotide-binding protein G(I)/G(S)/G(T) subunit beta-1

PDPK1; 3-phosphoinositide dependent protein kinase-1 [EC:2.7.11.1]

YWHAE; 14-3-3 protein epsilon

MTOR; serine/threonine-protein kinase mTOR [EC:2.7.11.1]

RAPTOR; regulatory associated protein of mTOR

RHEB; Ras homolog enriched in brain

KRAS; GTPase KRas

MLST8; target of rapamycin complex subunit LST8

CDC37; cell division cycle protein 37

PPP2R5; serine/threonine-protein phosphatase 2A regulatory subunit B'

SGK2; serum/glucocorticoid-regulated kinase 2 [EC:2.7.11.1]

56. Various types of N-glycan biosynthesis

ALG1; beta-1,4-mannosyltransferase [EC:2.4.1.142]

ALG2; alpha-1,3/alpha-1,6-mannosyltransferase [EC:2.4.1.132 2.4.1.257]

ALG11; alpha-1,2-mannosyltransferase [EC:2.4.1.131]

ALG3; alpha-1,3-mannosyltransferase [EC:2.4.1.258]

ALG9; alpha-1,2-mannosyltransferase [EC:2.4.1.259 2.4.1.261]

ALG12; alpha-1,6-mannosyltransferase [EC:2.4.1.260]

OCH1; alpha 1,6-mannosyltransferase [EC:2.4.1.232]

MNN9; mannan polymerase complexes MNN9 subunit [EC:2.4.1.232]

MNN10; mannan polymerase II complex MNN10 subunit [EC:2.4.1.-]

MNN11; mannan polymerase II complex MNN11 subunit [EC:2.4.1.-]

ANP1; mannan polymerase II complex ANP1 subunit [EC:2.4.1.-]

HOC1; mannan polymerase II complex HOC1 subunit [EC:2.4.1.-]

MNN2; alpha 1,2-mannosyltransferase [EC:2.4.1.-]

MNN1; alpha 1,3-mannosyltransferase [EC:2.4.1.-]

STT3; dolichyl-diphosphooligosaccharide---protein glycosyltransferase [EC:2.4.99.18]

ALG13; beta-1,4-N-acetylglucosaminyltransferase [EC:2.4.1.141]

HEXA_B; hexosaminidase [EC:3.2.1.52]

OST1; oligosaccharyltransferase complex subunit alpha (ribophorin I)

SWP1; oligosaccharyltransferase complex subunit delta (ribophorin II)

OST2; oligosaccharyltransferase complex subunit epsilon

OST3; oligosaccharyltransferase complex subunit gamma

WBP1; oligosaccharyltransferase complex subunit beta

MAN1B; endoplasmic reticulum Man9GlcNAc2 1,2-alpha-mannosidase [EC:3.2.1.209]

57. N-Glycan biosynthesis

ALG5; dolichyl-phosphate beta-glucosyltransferase [EC:2.4.1.117]

DOLK; dolichol kinase [EC:2.7.1.108]

ALG7; UDP-N-acetylglucosamine--dolichyl-phosphate N-acetyl glucosamine phosphotransferase [EC:2.7.8.15]

MOGS; mannosyl-oligosaccharide glucosidase [EC:3.2.1.106]

ALG1; beta-1,4-mannosyltransferase [EC:2.4.1.142]

ALG2; alpha-1,3/alpha-1,6-mannosyltransferase [EC:2.4.1.132 2.4.1.257]

ALG11; alpha-1,2-mannosyltransferase [EC:2.4.1.131]

ALG3; alpha-1,3-mannosyltransferase [EC:2.4.1.258]

ALG9; alpha-1,2-mannosyltransferase [EC:2.4.1.259 2.4.1.261]

ALG12; alpha-1,6-mannosyltransferase [EC:2.4.1.260]

ALG6; alpha-1,3-glucosyltransferase [EC:2.4.1.267]

ALG10; alpha-1,2-glucosyltransferase [EC:2.4.1.256]

STT3; dolichyl-diphosphooligosaccharide---protein glycosyltransferase [EC:2.4.99.18]

DOLPP1; dolichyldiphosphatase [EC:3.6.1.43]

ALG13; beta-1,4-N-acetylglucosaminyltransferase [EC:2.4.1.141]

DPM2; dolichol phosphate-mannose biosynthesis regulatory protein

SRD5A3; 3-oxo-5-alpha-steroid 4-dehydrogenase 3 / polyprenol reductase [EC:1.3.1.22 1.3.1.94]

OST1; oligosaccharyltransferase complex subunit alpha (ribophorin I)

SWP1; oligosaccharyltransferase complex subunit delta (ribophorin II)

OST2; oligosaccharyltransferase complex subunit epsilon

OST3; oligosaccharyltransferase complex subunit gamma

WBP1; oligosaccharyltransferase complex subunit beta

MAN1B; endoplasmic reticulum Man9GlcNAc2 1,2-alpha-mannosidase [EC:3.2.1.209]

58. Lysosome

LIPA; lysosomal acid lipase/cholesteryl ester hydrolase [EC:3.1.1.13]

PPT; palmitoyl-protein thioesterase [EC:3.1.2.22]

E3.2.1.25; beta-mannosidase [EC:3.2.1.25]

ATPeV0D; V-type H+-transporting ATPase subunit d

ATPeV0A; V-type H+-transporting ATPase subunit a

ATPeV0C; V-type H+-transporting ATPase 16kDa proteolipid subunit

ATPeV0B; V-type H+-transporting ATPase 21kDa proteolipid subunit

CLTC; clathrin heavy chain

M6PR; cation-dependent mannose-6-phosphate receptor

HEXA_B; hexosaminidase [EC:3.2.1.52]

NPC1; Niemann-Pick C1 protein

BTS; battenin

AP1G1; AP-1 complex subunit gamma-1

AP1B1; AP-1 complex subunit beta-1

AP1M; AP-1 complex subunit mu

AP1S1_2; AP-1 complex subunit sigma 1/2

AP3D; AP-3 complex subunit delta

AP3B; AP-3 complex subunit beta

AP3M; AP-3 complex subunit mu

AP3S; AP-3 complex subunit sigma

GGA; ADP-ribosylation factor-binding protein GGA

CTSA; cathepsin A (carboxypeptidase C) [EC:3.4.16.5]

59. AMPK signaling pathway

HMGCR; hydroxymethylglutaryl-CoA reductase (NADPH) [EC:1.1.1.34]

SCD; stearoyl-CoA desaturase (Delta-9 desaturase) [EC:1.14.19.1]

GYS; glycogen synthase [EC:2.4.1.11]

pfkA; 6-phosphofructokinase 1 [EC:2.7.1.11]

EEF2; elongation factor 2

PPP2R1; serine/threonine-protein phosphatase 2A regulatory subunit A

FBP; fructose-1,6-bisphosphatase I [EC:3.1.3.11]

PPP2R2; serine/threonine-protein phosphatase 2A regulatory subunit B

PDPK1; 3-phosphoinositide dependent protein kinase-1 [EC:2.7.11.1]

PRKAB; 5'-AMP-activated protein kinase, regulatory beta subunit

PRKAG; 5'-AMP-activated protein kinase, regulatory gamma subunit

MTOR; serine/threonine-protein kinase mTOR [EC:2.7.11.1]

RAPTOR; regulatory associated protein of mTOR

RHEB; Ras homolog enriched in brain

ADIPOR; adiponectin receptor

RAB8A; Ras-related protein Rab-8A

RAB11B; Ras-related protein Rab-11B

CAB39; calcium binding protein 39

ACACA; acetyl-CoA carboxylase / biotin carboxylase 1 [EC:6.4.1.2 6.3.4.14 2.1.3.15]

PPP2R5; serine/threonine-protein phosphatase 2A regulatory subunit B'

PFKFB2; 6-phosphofructo-2-kinase / fructose-2,6-biphosphatase 2 [EC:2.7.1.105 3.1.3.46]

PFKFB4; 6-phosphofructo-2-kinase / fructose-2,6-biphosphatase 4 [EC:2.7.1.105 3.1.3.46]

60. Human immunodeficiency virus 1 infection

CALM; calmodulin

PRKCA; classical protein kinase C alpha type [EC:2.7.11.13]

SKP1; S-phase kinase-associated protein 1

CUL1; cullin 1

ELOC; elongin-C

PPP3C; serine/threonine-protein phosphatase 2B catalytic subunit [EC:3.1.3.16]

ERK; mitogen-activated protein kinase 1/3 [EC:2.7.11.24]

RAC1; Ras-related C3 botulinum toxin substrate 1

PAK1; p21-activated kinase 1 [EC:2.7.11.1]

P38; p38 MAP kinase [EC:2.7.11.24]

GNB1; guanine nucleotide-binding protein G(I)/G(S)/G(T) subunit beta-1

GNAI; guanine nucleotide-binding protein G(i) subunit alpha

ATM; serine-protein kinase ATM [EC:2.7.11.1]

PPP3R; serine/threonine-protein phosphatase 2B regulatory subunit

MTOR; serine/threonine-protein kinase mTOR [EC:2.7.11.1]

KRAS; GTPase KRas

CYC; cytochrome c

CUL4; cullin 4

AP1G1; AP-1 complex subunit gamma-1

AP1B1; AP-1 complex subunit beta-1

AP1M; AP-1 complex subunit mu

AP1S1_2; AP-1 complex subunit sigma ½

61. Fatty acid metabolism

fabG; 3-oxoacyl-[acyl-carrier protein] reductase [EC:1.1.1.100]

E1.3.3.6; acyl-CoA oxidase [EC:1.3.3.6]

ACADM; acyl-CoA dehydrogenase [EC:1.3.8.7]

SCD; stearoyl-CoA desaturase (Delta-9 desaturase) [EC:1.14.19.1]

ACAT; acetyl-CoA C-acetyltransferase [EC:2.3.1.9]

fabD; [acyl-carrier-protein] S-malonyltransferase [EC:2.3.1.39]

FAS2; fatty acid synthase subunit alpha, fungi type [EC:2.3.1.86]

FAS1; fatty acid synthase subunit beta, fungi type [EC:2.3.1.86]

PPT; palmitoyl-protein thioesterase [EC:3.1.2.22]

ACSL; long-chain acyl-CoA synthetase [EC:6.2.1.3]

MECR; mitochondrial enoyl-[acyl-carrier protein] reductase / trans-2-enoyl-CoA reductase [EC:1.3.1.- 1.3.1.38]

ACAA1; acetyl-CoA acyltransferase 1 [EC:2.3.1.16]

fabF; 3-oxoacyl-[acyl-carrier-protein] synthase II [EC:2.3.1.179]

ELO2; fatty acid elongase 2 [EC:2.3.1.199]

ELO3; fatty acid elongase 3 [EC:2.3.1.199]

HSD17B12; 17beta-estradiol 17-dehydrogenase / very-long-chain 3-oxoacyl-CoA reductase [EC:1.1.1.62 1.1.1.330]

FAD2; omega-6 fatty acid desaturase / acyl-lipid omega-6 desaturase (Delta-12 desaturase) [EC:1.14.19.6 1.14.19.22]

TER; very-long-chain enoyl-CoA reductase [EC:1.3.1.93]

HACD; very-long-chain (3R)-3-hydroxyacyl-CoA dehydratase [EC:4.2.1.134]

ACACA; acetyl-CoA carboxylase / biotin carboxylase 1 [EC:6.4.1.2 6.3.4.14 2.1.3.15]

HTD2; 3-hydroxyacyl-thioester dehydratase, fungi type [EC:4.2.1.-]

FAD3; omega-3 fatty acid desaturase (Delta-15 desaturase) [EC:1.14.19.-]

62. Phagosome

PIK3C3; phosphatidylinositol 3-kinase [EC:2.7.1.137]

PIKFYVE; 1-phosphatidylinositol-3-phosphate 5-kinase [EC:2.7.1.150]

ATPeV1A; V-type H+-transporting ATPase subunit A [EC:7.1.2.2]

ATPeV0D; V-type H+-transporting ATPase subunit d

ATPeV1B; V-type H+-transporting ATPase subunit B

ATPeV1C; V-type H+-transporting ATPase subunit C

ATPeV1D; V-type H+-transporting ATPase subunit D

ATPeV1G; V-type H+-transporting ATPase subunit G

ATPeV0A; V-type H+-transporting ATPase subunit a

ATPeV0C; V-type H+-transporting ATPase 16kDa proteolipid subunit

ATPeV0B; V-type H+-transporting ATPase 21kDa proteolipid subunit

RAC1; Ras-related C3 botulinum toxin substrate 1

RAB5C; Ras-related protein Rab-5C

RAB7A; Ras-related protein Rab-7A

CANX; calnexin

STX18; syntaxin 18

SEC22; vesicle transport protein SEC22

M6PR; cation-dependent mannose-6-phosphate receptor

DYNC1H; dynein heavy chain 1, cytosolic

DYNC1I; dynein intermediate chain, cytosolic

SEC61A; protein transport protein SEC61 subunit alpha

HGS; hepatocyte growth factor-regulated tyrosine kinase substrate

63. Glycosylphosphatidylinositol (GPI)-anchor biosynthesis

PIGL; N-acetylglucosaminylphosphatidylinositol deacetylase [EC:3.5.1.89]

PIGA; phosphatidylinositol N-acetylglucosaminyltransferase subunit A [EC:2.4.1.198]

PIGH; phosphatidylinositol N-acetylglucosaminyltransferase subunit H

PIGC; phosphatidylinositol N-acetylglucosaminyltransferase subunit C

PIGQ; phosphatidylinositol N-acetylglucosaminyltransferase subunit Q

PIGW; glucosaminylphosphatidylinositol acyltransferase [EC:2.3.-.-]

PIGM; GPI mannosyltransferase 1 subunit M [EC:2.4.1.-]

PIGN; GPI ethanolamine phosphate transferase 1 [EC:2.7.-.-]

PIGB; GPI mannosyltransferase 3 [EC:2.4.1.-]

PIGF; GPI ethanolamine phosphate transferase 2/3 subunit F

PIGO; GPI ethanolamine phosphate transferase 3 subunit O [EC:2.7.-.-]

GAA1; GPI-anchor transamidase subunit GAA1

PIGK; GPI-anchor transamidase subunit K

PIGS; GPI-anchor transamidase subunit S

PIGT; GPI-anchor transamidase subunit T

PIGU; GPI-anchor transamidase subunit U

PGAP1; GPI inositol-deacylase [EC:3.-.-.-]

PIGG; ethanolamine phosphate transferase 2 subunit G [EC:2.7.-.-]

PIGV; GPI mannosyltransferase 2 [EC:2.4.1.-]

PIGZ; GPI mannosyltransferase 4 [EC:2.4.1.-]

DPM2; dolichol phosphate-mannose biosynthesis regulatory protein

64. Alanine, aspartate and glutamate metabolism

gabD; succinate-semialdehyde dehydrogenase / glutarate-semialdehyde dehydrogenase [EC:1.2.1.16 1.2.1.79 1.2.1.20]

E1.4.1.4; glutamate dehydrogenase (NADP+) [EC:1.4.1.4]

E1.2.1.88; 1-pyrroline-5-carboxylate dehydrogenase [EC:1.2.1.88]

purF; amidophosphoribosyltransferase [EC:2.4.2.14]

GPT; alanine transaminase [EC:2.6.1.2]

glmS; glutamine---fructose-6-phosphate transaminase (isomerizing) [EC:2.6.1.16]

AGXT; alanine-glyoxylate transaminase / serine-glyoxylate transaminase / serine-pyruvate transaminase [EC:2.6.1.44 2.6.1.45 2.6.1.51]

E3.5.1.1; L-asparaginase [EC:3.5.1.1]

E4.1.1.15; glutamate decarboxylase [EC:4.1.1.15]

argH; argininosuccinate lyase [EC:4.3.2.1]

purB; adenylosuccinate lyase [EC:4.3.2.2]

purA; adenylosuccinate synthase [EC:6.3.4.4]

argG; argininosuccinate synthase [EC:6.3.4.5]

asnB; asparagine synthase (glutamine-hydrolysing) [EC:6.3.5.4]

carA; carbamoyl-phosphate synthase small subunit [EC:6.3.5.5]

URA2; carbamoyl-phosphate synthase / aspartate carbamoyltransferase [EC:6.3.5.5 2.1.3.2]

ABAT; 4-aminobutyrate aminotransferase / (S)-3-amino-2-methylpropionate transaminase [EC:2.6.1.19 2.6.1.22]

NIT2; omega-amidase [EC:3.5.1.3]

GOT1; aspartate aminotransferase, cytoplasmic [EC:2.6.1.1]

GOT2; aspartate aminotransferase, mitochondrial [EC:2.6.1.1]

GDH2; glutamate dehydrogenase [EC:1.4.1.2]

65. Insulin signaling pathway

PYG; glycogen phosphorylase [EC:2.4.1.1]

GYS; glycogen synthase [EC:2.4.1.11]

HK; hexokinase [EC:2.7.1.1]

CALM; calmodulin

GSK3B; glycogen synthase kinase 3 beta [EC:2.7.11.26]

SOS; son of sevenless

EIF4E; translation initiation factor 4E

FBP; fructose-1,6-bisphosphatase I [EC:3.1.3.11]

PKA; protein kinase A [EC:2.7.11.11]

ERK; mitogen-activated protein kinase 1/3 [EC:2.7.11.24]

PRKAR; cAMP-dependent protein kinase regulator

PPP1C; serine/threonine-protein phosphatase PP1 catalytic subunit [EC:3.1.3.16]

PDPK1; 3-phosphoinositide dependent protein kinase-1 [EC:2.7.11.1]

EXOC7; exocyst complex component 7

PRKAB; 5'-AMP-activated protein kinase, regulatory beta subunit

PRKAG; 5'-AMP-activated protein kinase, regulatory gamma subunit

MTOR; serine/threonine-protein kinase mTOR [EC:2.7.11.1]

RAPTOR; regulatory associated protein of mTOR

RHEB; Ras homolog enriched in brain

KRAS; GTPase KRas

ACACA; acetyl-CoA carboxylase / biotin carboxylase 1 [EC:6.4.1.2 6.3.4.14 2.1.3.15]

66. Glyoxylate and dicarboxylate metabolism

gyaR; glyoxylate reductase [EC:1.1.1.26]

MDH2; malate dehydrogenase [EC:1.1.1.37]

FDH; formate dehydrogenase [EC:1.17.1.9]

GLDC; glycine dehydrogenase [EC:1.4.4.2]

DLD; dihydrolipoamide dehydrogenase [EC:1.8.1.4]

glyA; glycine hydroxymethyltransferase [EC:2.1.2.1]

gcvT; aminomethyltransferase [EC:2.1.2.10]

ACAT; acetyl-CoA C-acetyltransferase [EC:2.3.1.9]

AGXT; alanine-glyoxylate transaminase / serine-glyoxylate transaminase / serine-pyruvate transaminase [EC:2.6.1.44 2.6.1.45 2.6.1.51]

E3.5.1.49; formamidase [EC:3.5.1.49]

E4.1.3.1; isocitrate lyase [EC:4.1.3.1]

aceB; malate synthase [EC:2.3.3.9]

CS; citrate synthase [EC:2.3.3.1]

ACO; aconitate hydratase [EC:4.2.1.3]

ACSS1_2; acetyl-CoA synthetase [EC:6.2.1.1]

gcvH; glycine cleavage system H protein

katE; catalase [EC:1.11.1.6]

HAO; (S)-2-hydroxy-acid oxidase [EC:1.1.3.15]

BNA7; kynurenine formamidase [EC:3.5.1.9]

GLYK; D-glycerate 3-kinase [EC:2.7.1.31]

67. Amino sugar and nucleotide sugar metabolism

E1.6.2.2; cytochrome-b5 reductase [EC:1.6.2.2]

GNPNAT1; glucosamine-phosphate N-acetyltransferase [EC:2.3.1.4]

CHS1; chitin synthase [EC:2.4.1.16]

glmS; glutamine---fructose-6-phosphate transaminase (isomerizing) [EC:2.6.1.16]

HK; hexokinase [EC:2.7.1.1]

galK; galactokinase [EC:2.7.1.6]

UGP2; UTP--glucose-1-phosphate uridylyltransferase [EC:2.7.7.9]

galT; UDPglucose--hexose-1-phosphate uridylyltransferase [EC:2.7.7.12]

GMPP; mannose-1-phosphate guanylyltransferase [EC:2.7.7.13]

E3.2.1.14; chitinase [EC:3.2.1.14]

nagZ; beta-N-acetylhexosaminidase [EC:3.2.1.52]

nagA; N-acetylglucosamine-6-phosphate deacetylase [EC:3.5.1.25]

E3.5.1.41; chitin deacetylase [EC:3.5.1.41]

manA; mannose-6-phosphate isomerase [EC:5.3.1.8]

GPI; glucose-6-phosphate isomerase [EC:5.3.1.9]

pgm; phosphoglucomutase [EC:5.4.2.2]

PGM3; phosphoacetylglucosamine mutase [EC:5.4.2.3]

nagB; glucosamine-6-phosphate deaminase [EC:3.5.99.6]

HEXA_B; hexosaminidase [EC:3.2.1.52]

PMM; phosphomannomutase [EC:5.4.2.8]

68. Longevity regulating pathway - multiple species

PNC1; nicotinamidase [EC:3.5.1.19]

E4.6.1.1; adenylate cyclase [EC:4.6.1.1]

HSPA1s; heat shock 70kDa protein 1/2/6/8

clpB; ATP-dependent Clp protease ATP-binding subunit ClpB

katE; catalase [EC:1.11.1.6]

PKA; protein kinase A [EC:2.7.11.11]

SOD2; superoxide dismutase, Fe-Mn family [EC:1.15.1.1]

SOD1; superoxide dismutase, Cu-Zn family [EC:1.15.1.1]

HDAC1_2; histone deacetylase 1/2 [EC:3.5.1.98]

PRKAB; 5'-AMP-activated protein kinase, regulatory beta subunit

PRKAG; 5'-AMP-activated protein kinase, regulatory gamma subunit

MTOR; serine/threonine-protein kinase mTOR [EC:2.7.11.1]

RAPTOR; regulatory associated protein of mTOR

KRAS; GTPase KRas

ATG5; autophagy-related protein 5

MSN2_4; zinc finger protein MSN2/4

SIR2; NAD+-dependent protein deacetylase SIR2 [EC:2.3.1.286]

GPR1; G protein-coupled receptor GPR1

RIM15; serine/threonine-protein kinase RIM15 [EC:2.7.11.1]

SCH9; serine/threonine protein kinase SCH9 [EC:2.7.11.1]

69. Homologous recombination

POLD1; DNA polymerase delta subunit 1 [EC:2.7.7.7]

POLD2; DNA polymerase delta subunit 2

TOP3; DNA topoisomerase III [EC:5.6.2.1]

POLD3; DNA polymerase delta subunit 3

RAD51; DNA repair protein RAD51

ATM; serine-protein kinase ATM [EC:2.7.11.1]

RFA1; replication factor A1

MUS81; crossover junction endonuclease MUS81 [EC:3.1.22.-]

RFA2; replication factor A2

RPA3; replication factor A3

MRE11; double-strand break repair protein MRE11

RAD50; DNA repair protein RAD50 [EC:3.6.-.-]

RAD52; DNA repair and recombination protein RAD52

RAD54L; DNA repair and recombination protein RAD54 and RAD54-like protein [EC:3.6.4.-]

RAD54B; DNA repair and recombination protein RAD54B [EC:3.6.4.-]

SHFM1; 26 proteasome complex subunit DSS1

EME1; crossover junction endonuclease EME1 [EC:3.1.22.-]

BLM; bloom syndrome protein [EC:3.6.4.12]

RAD57; DNA repair protein RAD57

RAD59; DNA repair protein RAD59

70. Fanconi anemia pathway

REV3L; DNA polymerase zeta [EC:2.7.7.7]

TOP3; DNA topoisomerase III [EC:5.6.2.1]

POLZ2; DNA polymerase zeta [EC:2.7.7.7]

POLH; DNA polymerase eta [EC:2.7.7.7]

REV1; DNA repair protein REV1 [EC:2.7.7.-]

RAD51; DNA repair protein RAD51

RFA1; replication factor A1

MLH1; DNA mismatch repair protein MLH1

MUS81; crossover junction endonuclease MUS81 [EC:3.1.22.-]

RFA2; replication factor A2

RPA3; replication factor A3

ERCC4; DNA excision repair protein ERCC-4 [EC:3.1.-.-]

ERCC1; DNA excision repair protein ERCC-1

PMS2; DNA mismatch repair protein PMS2

EME1; crossover junction endonuclease EME1 [EC:3.1.22.-]

BLM; bloom syndrome protein [EC:3.6.4.12]

TELO2; telomere length regulation protein

APITD1; centromere protein S

WDR48; WD repeat-containing protein 48

NCE4; RecQ-mediated genome instability protein 1

71. Starch and sucrose metabolism

PYG; glycogen phosphorylase [EC:2.4.1.1]

GYS; glycogen synthase [EC:2.4.1.11]

otsA; trehalose 6-phosphate synthase [EC:2.4.1.15 2.4.1.347]

GBE1; 1,4-alpha-glucan branching enzyme [EC:2.4.1.18]

E2.4.1.34; 1,3-beta-glucan synthase [EC:2.4.1.34]

GYG1; glycogenin [EC:2.4.1.186]

HK; hexokinase [EC:2.7.1.1]

UGP2; UTP--glucose-1-phosphate uridylyltransferase [EC:2.7.7.9]

SGA1; glucoamylase [EC:3.2.1.3]

IMA; oligo-1,6-glucosidase [EC:3.2.1.10]

malZ; alpha-glucosidase [EC:3.2.1.20]

INV; beta-fructofuranosidase [EC:3.2.1.26]

TREH; alpha,alpha-trehalase [EC:3.2.1.28]

AGL; glycogen debranching enzyme [EC:2.4.1.25 3.2.1.33]

E3.2.1.58; glucan 1,3-beta-glucosidase [EC:3.2.1.58]

GPI; glucose-6-phosphate isomerase [EC:5.3.1.9]

pgm; phosphoglucomutase [EC:5.4.2.2]

bglX; beta-glucosidase [EC:3.2.1.21]

TPS; trehalose 6-phosphate synthase/phosphatase [EC:2.4.1.15 3.1.3.12]

TSL1; trehalose 6-phosphate synthase complex regulatory subunit

72. Mismatch repair

POLD1; DNA polymerase delta subunit 1 [EC:2.7.7.7]

POLD2; DNA polymerase delta subunit 2

POLD3; DNA polymerase delta subunit 3

uvrD; DNA helicase II / ATP-dependent DNA helicase PcrA [EC:3.6.4.12]

PCNA; proliferating cell nuclear antigen

RFA1; replication factor A1

MLH1; DNA mismatch repair protein MLH1

MSH2; DNA mismatch repair protein MSH2

MSH3; DNA mismatch repair protein MSH3

MSH6; DNA mismatch repair protein MSH6

MLH3; DNA mismatch repair protein MLH3

RFA2; replication factor A2

RPA3; replication factor A3

EXO1; exonuclease 1 [EC:3.1.-.-]

LIG1; DNA ligase 1 [EC:6.5.1.1 6.5.1.6 6.5.1.7]

RFC1; replication factor C subunit 1

RFC2_4; replication factor C subunit 2/4

RFC3_5; replication factor C subunit 3/5

PMS2; DNA mismatch repair protein PMS2

73. Synaptic vesicle cycle

ATPeV1A; V-type H+-transporting ATPase subunit A [EC:7.1.2.2]

ATPeV0D; V-type H+-transporting ATPase subunit d

ATPeV1B; V-type H+-transporting ATPase subunit B

ATPeV1C; V-type H+-transporting ATPase subunit C

ATPeV1D; V-type H+-transporting ATPase subunit D

ATPeV1G; V-type H+-transporting ATPase subunit G

ATPeV0A; V-type H+-transporting ATPase subunit a

ATPeV0C; V-type H+-transporting ATPase 16kDa proteolipid subunit

ATPeV0B; V-type H+-transporting ATPase 21kDa proteolipid subunit

CLTC; clathrin heavy chain

NSF; vesicle-fusing ATPase [EC:3.6.4.6]

STX1B_2_3; syntaxin 1B/2/3

AP2A; AP-2 complex subunit alpha

AP2B1; AP-2 complex subunit beta-1

AP2M1; AP-2 complex subunit mu-1

AP2S1; AP-2 complex subunit sigma-1

SLC32A; solute carrier family 32 (vesicular inhibitory amino acid transporter)

STXBP1; syntaxin-binding protein 1

NAPA; alpha-soluble NSF attachment protein

74. Tuberculosis

PIK3C3; phosphatidylinositol 3-kinase [EC:2.7.1.137]

ATPeV0D; V-type H+-transporting ATPase subunit d

ATPeV0A; V-type H+-transporting ATPase subunit a

ATPeV0C; V-type H+-transporting ATPase 16kDa proteolipid subunit

CALM; calmodulin

ATPeV0B; V-type H+-transporting ATPase 21kDa proteolipid subunit

dnaK; molecular chaperone DnaK

groEL; chaperonin GroEL

PPP3C; serine/threonine-protein phosphatase 2B catalytic subunit [EC:3.1.3.16]

ERK; mitogen-activated protein kinase 1/3 [EC:2.7.11.24]

P38; p38 MAP kinase [EC:2.7.11.24]

RHOA; Ras homolog gene family, member A

SPHK; sphingosine kinase [EC:2.7.1.91]

PPP3R; serine/threonine-protein phosphatase 2B regulatory subunit

RAB5C; Ras-related protein Rab-5C

RAB7A; Ras-related protein Rab-7A

NFYB; nuclear transcription Y subunit beta

NFYC; nuclear transcription factor Y, gamma

CYC; cytochrome c

75. Citrate cycle (TCA cycle)

MDH2; malate dehydrogenase [EC:1.1.1.37]

IDH3; isocitrate dehydrogenase (NAD+) [EC:1.1.1.41]

IDH1; isocitrate dehydrogenase [EC:1.1.1.42]

PDHA; pyruvate dehydrogenase E1 component alpha subunit [EC:1.2.4.1]

PDHB; pyruvate dehydrogenase E1 component beta subunit [EC:1.2.4.1]

OGDH; 2-oxoglutarate dehydrogenase E1 component [EC:1.2.4.2]

SDHA; succinate dehydrogenase (ubiquinone) flavoprotein subunit [EC:1.3.5.1]

SDHB; succinate dehydrogenase (ubiquinone) iron-sulfur subunit [EC:1.3.5.1]

SDHD; succinate dehydrogenase (ubiquinone) membrane anchor subunit

DLD; dihydrolipoamide dehydrogenase [EC:1.8.1.4]

DLAT; pyruvate dehydrogenase E2 component (dihydrolipoamide acetyltransferase) [EC:2.3.1.12]

DLST; 2-oxoglutarate dehydrogenase E2 component (dihydrolipoamide succinyltransferase) [EC:2.3.1.61]

E4.1.1.49; phosphoenolpyruvate carboxykinase (ATP) [EC:4.1.1.49]

CS; citrate synthase [EC:2.3.3.1]

E4.2.1.2B; fumarate hydratase, class II [EC:4.2.1.2]

ACO; aconitate hydratase [EC:4.2.1.3]

LSC1; succinyl-CoA synthetase alpha subunit [EC:6.2.1.4 6.2.1.5]

LSC2; succinyl-CoA synthetase beta subunit [EC:6.2.1.4 6.2.1.5]

PC; pyruvate carboxylase [EC:6.4.1.1]

76. Sphingolipid signaling pathway

SPT; serine palmitoyltransferase [EC:2.3.1.50]

PTEN; phosphatidylinositol-3,4,5-trisphosphate 3-phosphatase and dual-specificity protein phosphatase PTEN [EC:3.1.3.16 3.1.3.48 3.1.3.67]

PLD1_2; phospholipase D1/2 [EC:3.1.4.4]

SGPL1; sphinganine-1-phosphate aldolase [EC:4.1.2.27]

PRKCA; classical protein kinase C alpha type [EC:2.7.11.13]

PPP2R1; serine/threonine-protein phosphatase 2A regulatory subunit A

PPP2R2; serine/threonine-protein phosphatase 2A regulatory subunit B

ERK; mitogen-activated protein kinase 1/3 [EC:2.7.11.24]

RAC1; Ras-related C3 botulinum toxin substrate 1

P38; p38 MAP kinase [EC:2.7.11.24]

RHOA; Ras homolog gene family, member A

GNAI; guanine nucleotide-binding protein G(i) subunit alpha

DEGS; sphingolipid 4-desaturase/C4-monooxygenase [EC:1.14.19.17 1.14.18.5]

SPHK; sphingosine kinase [EC:2.7.1.91]

ABCC1; ATP-binding cassette, subfamily C (CFTR/MRP), member 1 [EC:7.6.2.3]

PDPK1; 3-phosphoinositide dependent protein kinase-1 [EC:2.7.11.1]

KRAS; GTPase KRas

PPP2R5; serine/threonine-protein phosphatase 2A regulatory subunit B'

SMPD2; sphingomyelin phosphodiesterase 2 [EC:3.1.4.12]

77. Glycerolipid metabolism

AKR1A1; alcohol dehydrogenase (NADP+) [EC:1.1.1.2]

ALDH; aldehyde dehydrogenase (NAD+) [EC:1.2.1.3]

E2.3.1.158; phospholipid:diacylglycerol acyltransferase [EC:2.3.1.158]

DAK; triose/dihydroxyacetone kinase / FAD-AMP lyase (cyclizing) [EC:2.7.1.28 2.7.1.29 4.6.1.15]

lip; triacylglycerol lipase [EC:3.1.1.3]

MGLL; acylglycerol lipase [EC:3.1.1.23]

GPP; glycerol-1-phosphatase [EC:3.1.3.21]

E3.2.1.22B; alpha-galactosidase [EC:3.2.1.22]

GAT; glycerol-3-phosphate O-acyltransferase / dihydroxyacetone phosphate acyltransferase [EC:2.3.1.15 2.3.1.42]

AGPAT1_2; lysophosphatidate acyltransferase [EC:2.3.1.51]

LPT1; lysophospholipid acyltransferase [EC:2.3.1.51 2.3.1.23 2.3.1.-]

TGL4; TAG lipase / steryl ester hydrolase / phospholipase A2 / LPA acyltransferase [EC:3.1.1.3 3.1.1.13 3.1.1.4 2.3.1.51]

TGL3; TAG lipase / lysophosphatidylethanolamine acyltransferase [EC:3.1.1.3 2.3.1.-]

LPIN; phosphatidate phosphatase LPIN [EC:3.1.3.4]

GLYK; D-glycerate 3-kinase [EC:2.7.1.31]

ATG15; lipase ATG15 [EC:3.1.1.3]

GCY1; glycerol 2-dehydrogenase (NADP+) [EC:1.1.1.156]

DPP1; diacylglycerol diphosphate phosphatase / phosphatidate phosphatase [EC:3.1.3.81 3.1.3.4]

LOA1; 1-acylglycerol-3-phosphate O-acyltransferase [EC:2.3.1.51]

78. Inositol phosphate metabolism

mmsA; malonate-semialdehyde dehydrogenase (acetylating) / methylmalonate-semialdehyde dehydrogenase [EC:1.2.1.18 1.2.1.27]

PI4KA; phosphatidylinositol 4-kinase A [EC:2.7.1.67]

PIP5K; 1-phosphatidylinositol-4-phosphate 5-kinase [EC:2.7.1.68]

PIK3C3; phosphatidylinositol 3-kinase [EC:2.7.1.137]

IPMK; inositol-polyphosphate multikinase [EC:2.7.1.140 2.7.1.151]

PIKFYVE; 1-phosphatidylinositol-3-phosphate 5-kinase [EC:2.7.1.150]

CDIPT; CDP-diacylglycerol--inositol 3-phosphatidyltransferase [EC:2.7.8.11]

PTEN; phosphatidylinositol-3,4,5-trisphosphate 3-phosphatase and dual-specificity protein phosphatase PTEN [EC:3.1.3.16 3.1.3.48 3.1.3.67]

plc; 1-phosphatidylinositol phosphodiesterase [EC:4.6.1.13]

TPI; triosephosphate isomerase (TIM) [EC:5.3.1.1]

INO1; myo-inositol-1-phosphate synthase [EC:5.5.1.4]

PLCD; phosphatidylinositol phospholipase C, delta [EC:3.1.4.11]

PI4K2; phosphatidylinositol 4-kinase type 2 [EC:2.7.1.67]

MTMR6_7_8; myotubularin-related protein 6/7/8 [EC:3.1.3.64 3.1.3.95]

IPK1; inositol-pentakisphosphate 2-kinase [EC:2.7.1.158]

PI4KB; phosphatidylinositol 4-kinase B [EC:2.7.1.67]

SYNJ; synaptojanin [EC:3.1.3.36]

SAC1; phosphatidylinositol 4-phosphatase [EC:3.1.3.-]

FIG4; phosphatidylinositol 3,5-bisphosphate 5-phosphatase [EC:3.1.3.-]

79. Progesterone-mediated oocyte maturation

BUB1; checkpoint serine/threonine-protein kinase [EC:2.7.11.1]

APC1; anaphase-promoting complex subunit 1

APC2; anaphase-promoting complex subunit 2

APC3; anaphase-promoting complex subunit 3

APC4; anaphase-promoting complex subunit 4

APC5; anaphase-promoting complex subunit 5

APC6; anaphase-promoting complex subunit 6

APC8; anaphase-promoting complex subunit 8

APC10; anaphase-promoting complex subunit 10

APC11; anaphase-promoting complex subunit 11

CDH1; cell division cycle 20-like protein 1, cofactor of APC complex

HSP90A; molecular chaperone HtpG

PKA; protein kinase A [EC:2.7.11.11]

ERK; mitogen-activated protein kinase 1/3 [EC:2.7.11.24]

P38; p38 MAP kinase [EC:2.7.11.24]

GNAI; guanine nucleotide-binding protein G(i) subunit alpha

MAD1; mitotic spindle assembly checkpoint protein MAD1

KRAS; GTPase KRas

80. Phosphatidylinositol signaling system

PI4KA; phosphatidylinositol 4-kinase A [EC:2.7.1.67]

PIP5K; 1-phosphatidylinositol-4-phosphate 5-kinase [EC:2.7.1.68]

PIK3C3; phosphatidylinositol 3-kinase [EC:2.7.1.137]

IPMK; inositol-polyphosphate multikinase [EC:2.7.1.140 2.7.1.151]

PIKFYVE; 1-phosphatidylinositol-3-phosphate 5-kinase [EC:2.7.1.150]

CDIPT; CDP-diacylglycerol--inositol 3-phosphatidyltransferase [EC:2.7.8.11]

PTEN; phosphatidylinositol-3,4,5-trisphosphate 3-phosphatase and dual-specificity protein phosphatase PTEN [EC:3.1.3.16 3.1.3.48 3.1.3.67]

CALM; calmodulin

PRKCA; classical protein kinase C alpha type [EC:2.7.11.13]

PLCD; phosphatidylinositol phospholipase C, delta [EC:3.1.4.11]

IP6K; inositol-hexakisphosphate 5-kinase [EC:2.7.4.21]

PPIP5K; inositol-hexakisphosphate/diphosphoinositol-pentakisphosphate 1-kinase [EC:2.7.4.24]

PI4K2; phosphatidylinositol 4-kinase type 2 [EC:2.7.1.67]

MTMR6_7_8; myotubularin-related protein 6/7/8 [EC:3.1.3.64 3.1.3.95]

IPK1; inositol-pentakisphosphate 2-kinase [EC:2.7.1.158]

PI4KB; phosphatidylinositol 4-kinase B [EC:2.7.1.67]

SYNJ; synaptojanin [EC:3.1.3.36]

SAC1; phosphatidylinositol 4-phosphatase [EC:3.1.3.-]

81. Cellular senescence

PTEN; phosphatidylinositol-3,4,5-trisphosphate 3-phosphatase and dual-specificity protein phosphatase PTEN [EC:3.1.3.16 3.1.3.48 3.1.3.67]

CALM; calmodulin

HRAD1; cell cycle checkpoint protein [EC:3.1.11.2]

PPP3C; serine/threonine-protein phosphatase 2B catalytic subunit [EC:3.1.3.16]

ERK; mitogen-activated protein kinase 1/3 [EC:2.7.11.24]

P38; p38 MAP kinase [EC:2.7.11.24]

ATM; serine-protein kinase ATM [EC:2.7.11.1]

SLC25A4S; solute carrier family 25 (mitochondrial adenine nucleotide translocator), member 4/5/6/31

PPID; peptidyl-prolyl isomerase D [EC:5.2.1.8]

PPP3R; serine/threonine-protein phosphatase 2B regulatory subunit

PPP1C; serine/threonine-protein phosphatase PP1 catalytic subunit [EC:3.1.3.16]

MTOR; serine/threonine-protein kinase mTOR [EC:2.7.11.1]

RHEB; Ras homolog enriched in brain

KRAS; GTPase KRas

RBBP4; histone-binding protein RBBP4

MRE11; double-strand break repair protein MRE11

RAD50; DNA repair protein RAD50 [EC:3.6.-.-]

VDAC2; voltage-dependent anion channel protein 2

82. Autophagy - other

PIK3C3; phosphatidylinositol 3-kinase [EC:2.7.1.137]

MTOR; serine/threonine-protein kinase mTOR [EC:2.7.11.1]

RAPTOR; regulatory associated protein of mTOR

MLST8; target of rapamycin complex subunit LST8

ULK2; serine/threonine-protein kinase ULK2 [EC:2.7.11.1]

ATG11; autophagy-related protein 11

ATG13; autophagy-related protein 13

PIK3R4; phosphoinositide-3-kinase, regulatory subunit 4 [EC:2.7.11.1]

BECN; beclin

ATG7; ubiquitin-like modifier-activating enzyme ATG7

ATG5; autophagy-related protein 5

GABARAP; GABA(A) receptor-associated protein

ATG4; cysteine protease ATG4 [EC:3.4.22.-]

ATG3; ubiquitin-like-conjugating enzyme ATG3

IGBP1; immunoglobulin-binding protein 1

ATG2; autophagy-related protein 2

ATG9; autophagy-related protein 9

WIPI1_2; autophagy-related protein 18

83. Arginine and proline metabolism

ALDH; aldehyde dehydrogenase (NAD+) [EC:1.2.1.3]

proA; glutamate-5-semialdehyde dehydrogenase [EC:1.2.1.41]

DAO; D-amino-acid oxidase [EC:1.4.3.3]

proC; pyrroline-5-carboxylate reductase [EC:1.5.1.2]

E1.2.1.88; 1-pyrroline-5-carboxylate dehydrogenase [EC:1.2.1.88]

PRODH; proline dehydrogenase [EC:1.5.5.2]

speE; spermidine synthase [EC:2.5.1.16]

SMS; spermine synthase [EC:2.5.1.22]

rocD; ornithine--oxo-acid transaminase [EC:2.6.1.13]

proB; glutamate 5-kinase [EC:2.7.2.11]

E3.5.1.4; amidase [EC:3.5.1.4]

E3.5.3.1; arginase [EC:3.5.3.1]

speB; agmatinase [EC:3.5.3.11]

E4.1.1.17; ornithine decarboxylase [EC:4.1.1.17]

speD; S-adenosylmethionine decarboxylase [EC:4.1.1.50]

FMS1; polyamine oxidase [EC:1.5.3.17]

GOT1; aspartate aminotransferase, cytoplasmic [EC:2.6.1.1]

GOT2; aspartate aminotransferase, mitochondrial [EC:2.6.1.1]

84. SNARE interactions in vesicular transport

STX1B_2_3; syntaxin 1B/2/3

STX16; syntaxin 16

STX5; syntaxin 5

STX18; syntaxin 18

VTI1; vesicle transport through interaction with t-SNAREs 1

GOSR1; golgi SNAP receptor complex member 1

GOSR2; golgi SNAP receptor complex member 2

SEC20; protein transport protein SEC20

TLG1; member of the syntaxin family of t-SNAREs

STX8; syntaxin 8

VAM7; regulator of vacuolar morphogenesis

BET1; blocked early in transport 1

USE1; unconventional SNARE in the endoplasmic reticulum protein 1

VAMP7; vesicle-associated membrane protein 7

YKT6; synaptobrevin homolog YKT6

SEC22; vesicle transport protein SEC22

VAM3; syntaxin VAM3

85. Base excision repair

alkA; DNA-3-methyladenine glycosylase II [EC:3.2.2.21]

POLE; DNA polymerase epsilon subunit 1 [EC:2.7.7.7]

POLE2; DNA polymerase epsilon subunit 2 [EC:2.7.7.7]

POLE3; DNA polymerase epsilon subunit 3 [EC:2.7.7.7]

POLD1; DNA polymerase delta subunit 1 [EC:2.7.7.7]

POLD2; DNA polymerase delta subunit 2

POLD3; DNA polymerase delta subunit 3

POLE4; DNA polymerase epsilon subunit 4 [EC:2.7.7.7]

UNG; uracil-DNA glycosylase [EC:3.2.2.27]

FEN1; flap endonuclease-1 [EC:3.-.-.-]

PCNA; proliferating cell nuclear antigen

mutM; formamidopyrimidine-DNA glycosylase [EC:3.2.2.23 4.2.99.18]

LIG1; DNA ligase 1 [EC:6.5.1.1 6.5.1.6 6.5.1.7]

APEX1; AP endonuclease 1 [EC:4.2.99.18]

APEX2; AP endonuclease 2 [EC:4.2.99.18]

NTH; endonuclease III [EC:4.2.99.18]

TDG; thymine-DNA glycosylase [EC:3.2.2.29]

86. Human cytomegalovirus infection

CALM; calmodulin

PRKCA; classical protein kinase C alpha type [EC:2.7.11.13]

GSK3B; glycogen synthase kinase 3 beta [EC:2.7.11.26]

SOS; son of sevenless

PKA; protein kinase A [EC:2.7.11.11]

PPP3C; serine/threonine-protein phosphatase 2B catalytic subunit [EC:3.1.3.16]

ERK; mitogen-activated protein kinase 1/3 [EC:2.7.11.24]

RAC1; Ras-related C3 botulinum toxin substrate 1

P38; p38 MAP kinase [EC:2.7.11.24]

RHOA; Ras homolog gene family, member A

GNB1; guanine nucleotide-binding protein G(I)/G(S)/G(T) subunit beta-1

GNAI; guanine nucleotide-binding protein G(i) subunit alpha

PPP3R; serine/threonine-protein phosphatase 2B regulatory subunit

MTOR; serine/threonine-protein kinase mTOR [EC:2.7.11.1]

RHEB; Ras homolog enriched in brain

KRAS; GTPase KRas

CYC; cytochrome c

87. Nicotinate and nicotinamide metabolism

gabD; succinate-semialdehyde dehydrogenase / glutarate-semialdehyde dehydrogenase [EC:1.2.1.16 1.2.1.79 1.2.1.20]

pncB; nicotinate phosphoribosyltransferase [EC:6.3.4.21]

nadC; nicotinate-nucleotide pyrophosphorylase (carboxylating) [EC:2.4.2.19]

ppnK; NAD+ kinase [EC:2.7.1.23]

URH1; uridine nucleosidase [EC:3.2.2.3]

PNC1; nicotinamidase [EC:3.5.1.19]

E6.3.5.1; NAD+ synthase (glutamine-hydrolysing) [EC:6.3.5.1]

E3.6.1.22; NAD+ diphosphatase [EC:3.6.1.22]

punA; purine-nucleoside phosphorylase [EC:2.4.2.1]

surE; 5'-nucleotidase [EC:3.1.3.5]

NMNAT; nicotinamide mononucleotide adenylyltransferase [EC:2.7.7.1 2.7.7.18]

NRK1_2; nicotinamide/nicotinate riboside kinase [EC:2.7.1.22 2.7.1.173]

SIR2; NAD+-dependent protein deacetylase SIR2 [EC:2.3.1.286]

SIRT5; NAD+-dependent protein deacetylase sirtuin 5 [EC:2.3.1.286]

ISN1; IMP and pyridine-specific 5'-nucleotidase [EC:3.1.3.99 3.1.3.-]

SDT1; pyrimidine and pyridine-specific 5'-nucleotidase [EC:3.1.3.-]

POF1; nicotinamide-nucleotide adenylyltransferase [EC:2.7.7.1]

88. Glucagon signaling pathway

PDHA; pyruvate dehydrogenase E1 component alpha subunit [EC:1.2.4.1]

PDHB; pyruvate dehydrogenase E1 component beta subunit [EC:1.2.4.1]

PYG; glycogen phosphorylase [EC:2.4.1.1]

GYS; glycogen synthase [EC:2.4.1.11]

pfkA; 6-phosphofructokinase 1 [EC:2.7.1.11]

PK; pyruvate kinase [EC:2.7.1.40]

PGAM; 2,3-bisphosphoglycerate-dependent phosphoglycerate mutase [EC:5.4.2.11]

CALM; calmodulin

FBP; fructose-1,6-bisphosphatase I [EC:3.1.3.11]

PKA; protein kinase A [EC:2.7.11.11]

PPP3C; serine/threonine-protein phosphatase 2B catalytic subunit [EC:3.1.3.16]

PPP3R; serine/threonine-protein phosphatase 2B regulatory subunit

PRKAB; 5'-AMP-activated protein kinase, regulatory beta subunit

PRKAG; 5'-AMP-activated protein kinase, regulatory gamma subunit

ACACA; acetyl-CoA carboxylase / biotin carboxylase 1 [EC:6.4.1.2 6.3.4.14 2.1.3.15]

PRMT1; type I protein arginine methyltransferase [EC:2.1.1.319]

SMEK; protein phosphatase 4 regulatory subunit 3

89. Regulation of actin cytoskeleton

PIP5K; 1-phosphatidylinositol-4-phosphate 5-kinase [EC:2.7.1.68]

PIKFYVE; 1-phosphatidylinositol-3-phosphate 5-kinase [EC:2.7.1.150]

SOS; son of sevenless

ERK; mitogen-activated protein kinase 1/3 [EC:2.7.11.24]

RAC1; Ras-related C3 botulinum toxin substrate 1

CDC42; cell division control protein 42

PAK1; p21-activated kinase 1 [EC:2.7.11.1]

RHOA; Ras homolog gene family, member A

ARPC5; actin related protein 2/3 complex, subunit 5

ARPC4; actin related protein 2/3 complex, subunit 4

ARPC3; actin related protein 2/3 complex, subunit 3

ARPC1A_B; actin related protein 2/3 complex, subunit 1A/1B

ARPC2; actin related protein 2/3 complex, subunit 2

IQGAP2_3; Ras GTPase-activating-like protein IQGAP2/3

PPP1C; serine/threonine-protein phosphatase PP1 catalytic subunit [EC:3.1.3.16]

KRAS; GTPase KRas

WASL; neural Wistt-Aldrich syndrome protein

90. Glutathione metabolism

IDH1; isocitrate dehydrogenase [EC:1.1.1.42]

PGD; 6-phosphogluconate dehydrogenase [EC:1.1.1.44 1.1.1.343]

G6PD; glucose-6-phosphate 1-dehydrogenase [EC:1.1.1.49 1.1.1.363]

GSR; glutathione reductase (NADPH) [EC:1.8.1.7]

ggt; gamma-glutamyltranspeptidase / glutathione hydrolase [EC:2.3.2.2 3.4.19.13]

speE; spermidine synthase [EC:2.5.1.16]

GST; glutathione S-transferase [EC:2.5.1.18]

SMS; spermine synthase [EC:2.5.1.22]

OPLAH; 5-oxoprolinase (ATP-hydrolysing) [EC:3.5.2.9]

E4.1.1.17; ornithine decarboxylase [EC:4.1.1.17]

CHAC; glutathione-specific gamma-glutamylcyclotransferase [EC:4.3.2.7]

RRM1; ribonucleoside-diphosphate reductase subunit M1 [EC:1.17.4.1]

RRM2; ribonucleoside-diphosphate reductase subunit M2 [EC:1.17.4.1]

GCLC; glutamate--cysteine ligase catalytic subunit [EC:6.3.2.2]

DUG1; Cys-Gly metallodipeptidase DUG1 [EC:3.4.13.-]

GSS; glutathione synthase [EC:6.3.2.3]

PRX1; glutaredoxin/glutathione-dependent peroxiredoxin [EC:1.11.1.25 1.11.1.27]

91. Kaposi sarcoma-associated herpesvirus infection

PIK3C3; phosphatidylinositol 3-kinase [EC:2.7.1.137]

CALM; calmodulin

RP-S27Ae; ubiquitin-small subunit ribosomal protein S27Ae

GSK3B; glycogen synthase kinase 3 beta [EC:2.7.11.26]

PPP3C; serine/threonine-protein phosphatase 2B catalytic subunit [EC:3.1.3.16]

ERK; mitogen-activated protein kinase 1/3 [EC:2.7.11.24]

RAC1; Ras-related C3 botulinum toxin substrate 1

P38; p38 MAP kinase [EC:2.7.11.24]

GNB1; guanine nucleotide-binding protein G(I)/G(S)/G(T) subunit beta-1

PPP3R; serine/threonine-protein phosphatase 2B regulatory subunit

MTOR; serine/threonine-protein kinase mTOR [EC:2.7.11.1]

KRAS; GTPase KRas

BECN; beclin

GABARAP; GABA(A) receptor-associated protein

ATG3; ubiquitin-like-conjugating enzyme ATG3

CYC; cytochrome c

UBC; ubiquitin C

92. Ras signaling pathway

PLD1_2; phospholipase D1/2 [EC:3.1.4.4]

CALM; calmodulin

PRKCA; classical protein kinase C alpha type [EC:2.7.11.13]

SOS; son of sevenless

PKA; protein kinase A [EC:2.7.11.11]

ERK; mitogen-activated protein kinase 1/3 [EC:2.7.11.24]

RAC1; Ras-related C3 botulinum toxin substrate 1

CDC42; cell division control protein 42

PAK1; p21-activated kinase 1 [EC:2.7.11.1]

RHOA; Ras homolog gene family, member A

GNB1; guanine nucleotide-binding protein G(I)/G(S)/G(T) subunit beta-1

KRAS; GTPase KRas

RAP1B; Ras-related protein Rap-1B

RAB5C; Ras-related protein Rab-5C

ARF6; ADP-ribosylation factor 6

BRAP; BRCA1-associated protein [EC:2.3.2.27]

EXOC2; exocyst complex component 2

93. Platinum drug resistance

GST; glutathione S-transferase [EC:2.5.1.18]

REV3L; DNA polymerase zeta [EC:2.7.7.7]

TOP2; DNA topoisomerase II [EC:5.6.2.2]

POLH; DNA polymerase eta [EC:2.7.7.7]

ERK; mitogen-activated protein kinase 1/3 [EC:2.7.11.24]

ATM; serine-protein kinase ATM [EC:2.7.11.1]

PDPK1; 3-phosphoinositide dependent protein kinase-1 [EC:2.7.11.1]

MLH1; DNA mismatch repair protein MLH1

MSH2; DNA mismatch repair protein MSH2

MSH3; DNA mismatch repair protein MSH3

MSH6; DNA mismatch repair protein MSH6

CYC; cytochrome c

XPA; DNA-repair protein complementing XP-A cells

ERCC1; DNA excision repair protein ERCC-1

SLC31A1; solute carrier family 31 (copper transporter), member 1

copA; P-type Cu+ transporter [EC:7.2.2.8]

94. Alcoholism

CALM; calmodulin

SOS; son of sevenless

PKA; protein kinase A [EC:2.7.11.11]

ERK; mitogen-activated protein kinase 1/3 [EC:2.7.11.24]

GNB1; guanine nucleotide-binding protein G(I)/G(S)/G(T) subunit beta-1

GNAI; guanine nucleotide-binding protein G(i) subunit alpha

HDAC1_2; histone deacetylase 1/2 [EC:3.5.1.98]

PPP1C; serine/threonine-protein phosphatase PP1 catalytic subunit [EC:3.1.3.16]

KRAS; GTPase KRas

H2A; histone H2A

H2B; histone H2B

H3; histone H3

H4; histone H4

HAT1; histone acetyltransferase 1 [EC:2.3.1.48]

HDAC6; histone deacetylase 6 [EC:3.5.1.98]

SLC29A1_2_3; solute carrier family 29 (equilibrative nucleoside transporter), member 1/2/3

95. Methane metabolism

serA; D-3-phosphoglycerate dehydrogenase / 2-oxoglutarate reductase [EC:1.1.1.95 1.1.1.399]

frmA; S-(hydroxymethyl)glutathione dehydrogenase / alcohol dehydrogenase [EC:1.1.1.284 1.1.1.1]

FDH; formate dehydrogenase [EC:1.17.1.9]

glyA; glycine hydroxymethyltransferase [EC:2.1.2.1]

AGXT; alanine-glyoxylate transaminase / serine-glyoxylate transaminase / serine-pyruvate transaminase [EC:2.6.1.44 2.6.1.45 2.6.1.51]

serC; phosphoserine aminotransferase [EC:2.6.1.52]

pfkA; 6-phosphofructokinase 1 [EC:2.7.1.11]

DAK; triose/dihydroxyacetone kinase / FAD-AMP lyase (cyclizing) [EC:2.7.1.28 2.7.1.29 4.6.1.15]

frmB; S-formylglutathione hydrolase [EC:3.1.2.12]

serB; phosphoserine phosphatase [EC:3.1.3.3]

FBA; fructose-bisphosphate aldolase, class II [EC:4.1.2.13]

ENO; enolase [EC:4.2.1.11]

PGAM; 2,3-bisphosphoglycerate-dependent phosphoglycerate mutase [EC:5.4.2.11]

ACSS1_2; acetyl-CoA synthetase [EC:6.2.1.1]

FBP; fructose-1,6-bisphosphatase I [EC:3.1.3.11]

AS; dihydroxyacetone synthase [EC:2.2.1.3]

96. MAPK signaling pathway

PRKCA; classical protein kinase C alpha type [EC:2.7.11.13]

SOS; son of sevenless

HSPA1s; heat shock 70kDa protein 1/2/6/8

PKA; protein kinase A [EC:2.7.11.11]

PPP3C; serine/threonine-protein phosphatase 2B catalytic subunit [EC:3.1.3.16]

ERK; mitogen-activated protein kinase 1/3 [EC:2.7.11.24]

RAC1; Ras-related C3 botulinum toxin substrate 1

CDC42; cell division control protein 42

PAK1; p21-activated kinase 1 [EC:2.7.11.1]

P38; p38 MAP kinase [EC:2.7.11.24]

DUSP; dual specificity MAP kinase phosphatase [EC:3.1.3.16 3.1.3.48]

PPP5C; serine/threonine-protein phosphatase 5 [EC:3.1.3.16]

MAPK7; mitogen-activated protein kinase 7 [EC:2.7.11.24]

PPP3R; serine/threonine-protein phosphatase 2B regulatory subunit

KRAS; GTPase KRas

RAP1B; Ras-related protein Rap-1B

97. Phenylalanine, tyrosine and tryptophan biosynthesis

TYR1; prephenate dehydrogenase (NADP+) [EC:1.3.1.13]

trpD; anthranilate phosphoribosyltransferase [EC:2.4.2.18]

hisC; histidinol-phosphate aminotransferase [EC:2.6.1.9]

ARO8; aromatic amino acid aminotransferase I / 2-aminoadipate transaminase [EC:2.6.1.57 2.6.1.39 2.6.1.27 2.6.1.5]

E2.5.1.54; 3-deoxy-7-phosphoheptulonate synthase [EC:2.5.1.54]

TRP3; anthranilate synthase / indole-3-glycerol phosphate synthase [EC:4.1.3.27 4.1.1.48]

trpE; anthranilate synthase component I [EC:4.1.3.27]

TRP; tryptophan synthase [EC:4.2.1.20]

aroC; chorismate synthase [EC:4.2.3.5]

trpF; phosphoribosylanthranilate isomerase [EC:5.3.1.24]

E5.4.99.5; chorismate mutase [EC:5.4.99.5]

aroQ; 3-dehydroquinate dehydratase II [EC:4.2.1.10]

ARO9; aromatic amino acid aminotransferase II [EC:2.6.1.58 2.6.1.28]

ARO1; pentafunctional AROM polypeptide [EC:4.2.3.4 4.2.1.10 1.1.1.25 2.7.1.71 2.5.1.19]

GOT1; aspartate aminotransferase, cytoplasmic [EC:2.6.1.1]

GOT2; aspartate aminotransferase, mitochondrial [EC:2.6.1.1]

98. Fc gamma R-mediated phagocytosis

PIP5K; 1-phosphatidylinositol-4-phosphate 5-kinase [EC:2.7.1.68]

PLD1_2; phospholipase D1/2 [EC:3.1.4.4]

PRKCA; classical protein kinase C alpha type [EC:2.7.11.13]

ERK; mitogen-activated protein kinase 1/3 [EC:2.7.11.24]

RAC1; Ras-related C3 botulinum toxin substrate 1

CDC42; cell division control protein 42

PAK1; p21-activated kinase 1 [EC:2.7.11.1]

SPHK; sphingosine kinase [EC:2.7.1.91]

ARPC5; actin related protein 2/3 complex, subunit 5

ARPC4; actin related protein 2/3 complex, subunit 4

ARPC3; actin related protein 2/3 complex, subunit 3

ARPC1A_B; actin related protein 2/3 complex, subunit 1A/1B

ARPC2; actin related protein 2/3 complex, subunit 2

ARF6; ADP-ribosylation factor 6

ASAP; Arf-GAP with SH3 domain, ANK repeat and PH domain-containing protein

AMPH; amphiphysin

99. Thyroid hormone signaling pathway

pfkA; 6-phosphofructokinase 1 [EC:2.7.1.11]

PRKCA; classical protein kinase C alpha type [EC:2.7.11.13]

GSK3B; glycogen synthase kinase 3 beta [EC:2.7.11.26]

PKA; protein kinase A [EC:2.7.11.11]

ERK; mitogen-activated protein kinase 1/3 [EC:2.7.11.24]

PLCD; phosphatidylinositol phospholipase C, delta [EC:3.1.4.11]

PCAF; histone acetyltransferase [EC:2.3.1.48]

HDAC1_2; histone deacetylase 1/2 [EC:3.5.1.98]

PDPK1; 3-phosphoinositide dependent protein kinase-1 [EC:2.7.11.1]

MTOR; serine/threonine-protein kinase mTOR [EC:2.7.11.1]

RHEB; Ras homolog enriched in brain

KRAS; GTPase KRas

SIN3A; paired amphipathic helix protein Sin3a

MED4; mediator of RNA polymerase II transcription subunit 4

MED14; mediator of RNA polymerase II transcription subunit 14

PFKFB2; 6-phosphofructo-2-kinase / fructose-2,6-biphosphatase 2 [EC:2.7.1.105 3.1.3.46]

100. Proteoglycans in cancer

PRKCA; classical protein kinase C alpha type [EC:2.7.11.13]

SOS; son of sevenless

EIF4B; translation initiation factor 4B

rnc; ribonuclease III [EC:3.1.26.3]

PKA; protein kinase A [EC:2.7.11.11]

ERK; mitogen-activated protein kinase 1/3 [EC:2.7.11.24]

RAC1; Ras-related C3 botulinum toxin substrate 1

CDC42; cell division control protein 42

PAK1; p21-activated kinase 1 [EC:2.7.11.1]

P38; p38 MAP kinase [EC:2.7.11.24]

RHOA; Ras homolog gene family, member A

PPP1C; serine/threonine-protein phosphatase PP1 catalytic subunit [EC:3.1.3.16]

PDPK1; 3-phosphoinositide dependent protein kinase-1 [EC:2.7.11.1]

MTOR; serine/threonine-protein kinase mTOR [EC:2.7.11.1]

KRAS; GTPase KRas

DDX5; ATP-dependent RNA helicase DDX5/DBP2 [EC:3.6.4.13]

101. Arginine biosynthesis

E1.4.1.4; glutamate dehydrogenase (NADP+) [EC:1.4.1.4]

OTC; ornithine carbamoyltransferase [EC:2.1.3.3]

ARG2; amino-acid N-acetyltransferase [EC:2.3.1.1]

argJ; glutamate N-acetyltransferase / amino-acid N-acetyltransferase [EC:2.3.1.35 2.3.1.1]

GPT; alanine transaminase [EC:2.6.1.2]

E2.6.1.11; acetylornithine aminotransferase [EC:2.6.1.11]

argE; acetylornithine deacetylase [EC:3.5.1.16]

E3.5.3.1; arginase [EC:3.5.3.1]

argH; argininosuccinate lyase [EC:4.3.2.1]

argG; argininosuccinate synthase [EC:6.3.4.5]

E6.3.4.6; urea carboxylase [EC:6.3.4.6]

ARG56; N-acetyl-gamma-glutamyl-phosphate reductase / acetylglutamate kinase [EC:1.2.1.38 2.7.2.8]

GOT1; aspartate aminotransferase, cytoplasmic [EC:2.6.1.1]

GOT2; aspartate aminotransferase, mitochondrial [EC:2.6.1.1]

GDH2; glutamate dehydrogenase [EC:1.4.1.2]

102. Longevity regulating pathway - worm

SCD; stearoyl-CoA desaturase (Delta-9 desaturase) [EC:1.14.19.1]

GST; glutathione S-transferase [EC:2.5.1.18]

PTEN; phosphatidylinositol-3,4,5-trisphosphate 3-phosphatase and dual-specificity protein phosphatase PTEN [EC:3.1.3.16 3.1.3.48 3.1.3.67]

katE; catalase [EC:1.11.1.6]

dnaK; molecular chaperone DnaK

groEL; chaperonin GroEL

P38; p38 MAP kinase [EC:2.7.11.24]

SOD2; superoxide dismutase, Fe-Mn family [EC:1.15.1.1]

MTOR; serine/threonine-protein kinase mTOR [EC:2.7.11.1]

ULK2; serine/threonine-protein kinase ULK2 [EC:2.7.11.1]

GABARAP; GABA(A) receptor-associated protein

GCLC; glutamate--cysteine ligase catalytic subunit [EC:6.3.2.2]

UBL5; ubiquitin-like protein 5

SMEK; protein phosphatase 4 regulatory subunit 3

TIM23; mitochondrial import inner membrane translocase subunit TIM23

103. Tight junction

PPP2R1; serine/threonine-protein phosphatase 2A regulatory subunit A

PKA; protein kinase A [EC:2.7.11.11]

PPP2R2; serine/threonine-protein phosphatase 2A regulatory subunit B

RAC1; Ras-related C3 botulinum toxin substrate 1

CDC42; cell division control protein 42

RHOA; Ras homolog gene family, member A

PCNA; proliferating cell nuclear antigen

SYMPK; symplekin

PRKAB; 5'-AMP-activated protein kinase, regulatory beta subunit

PRKAG; 5'-AMP-activated protein kinase, regulatory gamma subunit

RAB8A; Ras-related protein Rab-8A

MYH; myosin heavy chain

NEDD4; E3 ubiquitin-protein ligase NEDD4 [EC:2.3.2.26]

ACTR2; actin-related protein 2

ACTR3; actin-related protein 3

104. Steroid biosynthesis

TM7SF2; Delta14-sterol reductase [EC:1.3.1.70]

ERG4; Delta24(24(1))-sterol reductase [EC:1.3.1.71]

SQLE; squalene monooxygenase [EC:1.14.14.17]

SMT1; sterol 24-C-methyltransferase [EC:2.1.1.41]

SOAT; sterol O-acyltransferase [EC:2.3.1.26]

FDFT1; farnesyl-diphosphate farnesyltransferase [EC:2.5.1.21]

LIPA; lysosomal acid lipase/cholesteryl ester hydrolase [EC:3.1.1.13]

LSS; lanosterol synthase [EC:5.4.99.7]

CYP51; sterol 14alpha-demethylase [EC:1.14.14.154 1.14.15.36]

NSDHL; sterol-4alpha-carboxylate 3-dehydrogenase (decarboxylating) [EC:1.1.1.170]

MESO1; methylsterol monooxygenase [EC:1.14.18.9]

ERG27; 3-keto steroid reductase [EC:1.1.1.270]

ERG2; C-8 sterol isomerase [EC:5.-.-.-]

ERG5; sterol 22-desaturase [EC:1.14.19.41]

TGL4; TAG lipase / steryl ester hydrolase / phospholipase A2 / LPA acyltransferase [EC:3.1.1.3 3.1.1.13 3.1.1.4 2.3.1.51]

105. Mitophagy - animal

RP-S27Ae; ubiquitin-small subunit ribosomal protein S27Ae

CSNK2A; casein kinase II subunit alpha [EC:2.7.11.1]

CSNK2B; casein kinase II subunit beta

MFN2; mitofusin 2 [EC:3.6.5.-]

KRAS; GTPase KRas

RHOT1; mitochondrial Rho GTPase 1 [EC:3.6.5.-]

RAB7A; Ras-related protein Rab-7A

BECN; beclin

ATG5; autophagy-related protein 5

GABARAP; GABA(A) receptor-associated protein

UBC; ubiquitin C

USP8; ubiquitin carboxyl-terminal hydrolase 8 [EC:3.4.19.12]

ATG9; autophagy-related protein 9

FIS1; mitochondrial fission 1 protein

TBC1D15; TBC1 domain family member 15

106. Influenza A

FDPS; farnesyl diphosphate synthase [EC:2.5.1.1 2.5.1.10]

PRKCA; classical protein kinase C alpha type [EC:2.7.11.13]

EIF2S1; translation initiation factor 2 subunit 1

ERK; mitogen-activated protein kinase 1/3 [EC:2.7.11.24]

SLC25A4S; solute carrier family 25 (mitochondrial adenine nucleotide translocator), member 4/5/6/31

RAB11B; Ras-related protein Rab-11B

CYC; cytochrome c

DNAJC3; DnaJ homolog subfamily C member 3

NXF; nuclear RNA export factor

XPO1; exportin-1

NUP98; nuclear pore complex protein Nup98-Nup96

RAE1; mRNA export factor

PABPN1; polyadenylate-binding protein 2

CPSF4; cleavage and polyadenylation specificity factor subunit 4

KPNA5_6; importin subunit alpha-6/7

107. Pentose phosphate pathway

PGD; 6-phosphogluconate dehydrogenase [EC:1.1.1.44 1.1.1.343]

G6PD; glucose-6-phosphate 1-dehydrogenase [EC:1.1.1.49 1.1.1.363]

E2.2.1.1; transketolase [EC:2.2.1.1]

E2.2.1.2; transaldolase [EC:2.2.1.2]

pfkA; 6-phosphofructokinase 1 [EC:2.7.1.11]

E2.7.1.12; gluconokinase [EC:2.7.1.12]

rbsK; ribokinase [EC:2.7.1.15]

PRPS; ribose-phosphate pyrophosphokinase [EC:2.7.6.1]

PGLS; 6-phosphogluconolactonase [EC:3.1.1.31]

FBA; fructose-bisphosphate aldolase, class II [EC:4.1.2.13]

rpe; ribulose-phosphate 3-epimerase [EC:5.1.3.1]

rpiA; ribose 5-phosphate isomerase A [EC:5.3.1.6]

GPI; glucose-6-phosphate isomerase [EC:5.3.1.9]

pgm; phosphoglucomutase [EC:5.4.2.2]

FBP; fructose-1,6-bisphosphatase I [EC:3.1.3.11]

108. FoxO signaling pathway

PTEN; phosphatidylinositol-3,4,5-trisphosphate 3-phosphatase and dual-specificity protein phosphatase PTEN [EC:3.1.3.16 3.1.3.48 3.1.3.67]

SOS; son of sevenless

katE; catalase [EC:1.11.1.6]

ERK; mitogen-activated protein kinase 1/3 [EC:2.7.11.24]

P38; p38 MAP kinase [EC:2.7.11.24]

SOD2; superoxide dismutase, Fe-Mn family [EC:1.15.1.1]

ATM; serine-protein kinase ATM [EC:2.7.11.1]

PDPK1; 3-phosphoinositide dependent protein kinase-1 [EC:2.7.11.1]

PRKAB; 5'-AMP-activated protein kinase, regulatory beta subunit

PRKAG; 5'-AMP-activated protein kinase, regulatory gamma subunit

KRAS; GTPase KRas

GABARAP; GABA(A) receptor-associated protein

PRMT1; type I protein arginine methyltransferase [EC:2.1.1.319]

USP7; ubiquitin carboxyl-terminal hydrolase 7 [EC:3.4.19.12]

SGK2; serum/glucocorticoid-regulated kinase 2 [EC:2.7.11.1]

109. Fructose and mannose metabolism

SORD; L-iditol 2-dehydrogenase [EC:1.1.1.14]

HK; hexokinase [EC:2.7.1.1]

pfkA; 6-phosphofructokinase 1 [EC:2.7.1.11]

DAK; triose/dihydroxyacetone kinase / FAD-AMP lyase (cyclizing) [EC:2.7.1.28 2.7.1.29 4.6.1.15]

E2.7.1.105; 6-phosphofructo-2-kinase [EC:2.7.1.105]

GMPP; mannose-1-phosphate guanylyltransferase [EC:2.7.7.13]

FBA; fructose-bisphosphate aldolase, class II [EC:4.1.2.13]

TPI; triosephosphate isomerase (TIM) [EC:5.3.1.1]

manA; mannose-6-phosphate isomerase [EC:5.3.1.8]

FBP; fructose-1,6-bisphosphatase I [EC:3.1.3.11]

PMM; phosphomannomutase [EC:5.4.2.8]

SOU1; sorbose reductase [EC:1.1.1.289]

LRA4; 2-keto-3-deoxy-L-rhamnonate aldolase [EC:4.1.2.53]

PFKFB2; 6-phosphofructo-2-kinase / fructose-2,6-biphosphatase 2 [EC:2.7.1.105 3.1.3.46]

PFKFB4; 6-phosphofructo-2-kinase / fructose-2,6-biphosphatase 4 [EC:2.7.1.105 3.1.3.46]

110. Necroptosis

PYG; glycogen phosphorylase [EC:2.4.1.1]

HSP90A; molecular chaperone HtpG

SLC25A4S; solute carrier family 25 (mitochondrial adenine nucleotide translocator), member 4/5/6/31

PPID; peptidyl-prolyl isomerase D [EC:5.2.1.8]

H2A; histone H2A

CHMP2A; charged multivesicular body protein 2A

VPS24; charged multivesicular body protein 3

CHMP4; charged multivesicular body protein 4

CHMP6; charged multivesicular body protein 6

VPS4; vacuolar protein-sorting-associated protein 4

CHMP1; charged multivesicular body protein 1

CHMP5; charged multivesicular body protein 5

VDAC2; voltage-dependent anion channel protein 2

CHMP7; charged multivesicular body protein 7

DNM1L; dynamin 1-like protein [EC:3.6.5.5]

111. Two-component system

ME2; malate dehydrogenase (oxaloacetate-decarboxylating) [EC:1.1.1.38]

UQCRFS1; ubiquinol-cytochrome c reductase iron-sulfur subunit [EC:7.1.1.8]

CYC1; ubiquinol-cytochrome c reductase cytochrome c1 subunit

ACAT; acetyl-CoA C-acetyltransferase [EC:2.3.1.9]

E3.1.3.1; alkaline phosphatase [EC:3.1.3.1]

E3.1.3.48; protein-tyrosine phosphatase [EC:3.1.3.48]

COX15; cytochrome c oxidase assembly protein subunit 15

CYC; cytochrome c

SSK2; mitogen-activated protein kinase kinase kinase [EC:2.7.11.25]

SLN1; osomolarity two-component system, sensor histidine kinase SLN1 [EC:2.7.13.3]

YPD1; osomolarity two-component system, phosphorelay intermediate protein YPD1

SSK1; osomolarity two-component system, response regulator SSK1

SKN7; osomolarity two-component system, response regulator SKN7

CHK1; osomolarity two-component system, sensor histidine kinase CHK1 [EC:2.7.13.3]

NIK1; osomolarity two-component system, sensor histidine kinase NIK1 [EC:2.7.13.3]

112. Terpenoid backbone biosynthesis

HMGCR; hydroxymethylglutaryl-CoA reductase (NADPH) [EC:1.1.1.34]

ICMT; protein-S-isoprenylcysteine O-methyltransferase [EC:2.1.1.100]

ACAT; acetyl-CoA C-acetyltransferase [EC:2.3.1.9]

FDPS; farnesyl diphosphate synthase [EC:2.5.1.1 2.5.1.10]

GGPS1; geranylgeranyl diphosphate synthase, type III [EC:2.5.1.1 2.5.1.10 2.5.1.29]

E2.7.1.36; mevalonate kinase [EC:2.7.1.36]

E2.7.4.2; phosphomevalonate kinase [EC:2.7.4.2]

E2.3.3.10; hydroxymethylglutaryl-CoA synthase [EC:2.3.3.10]

idi; isopentenyl-diphosphate Delta-isomerase [EC:5.3.3.2]

hexPS; hexaprenyl-diphosphate synthase [EC:2.5.1.82 2.5.1.83]

FNTB; protein farnesyltransferase subunit beta [EC:2.5.1.58]

FNTA; protein farnesyltransferase/geranylgeranyltransferase type-1 subunit alpha [EC:2.5.1.58 2.5.1.59]

STE24; STE24 endopeptidase [EC:3.4.24.84]

DHDDS; ditrans,polycis-polyprenyl diphosphate synthase [EC:2.5.1.87]

NUS1; dehydrodolichyl diphosphate syntase complex subunit NUS1 [EC:2.5.1.87]

113. Oxytocin signaling pathway

CALM; calmodulin

PRKCA; classical protein kinase C alpha type [EC:2.7.11.13]

EEF2; elongation factor 2

PKA; protein kinase A [EC:2.7.11.11]

PPP3C; serine/threonine-protein phosphatase 2B catalytic subunit [EC:3.1.3.16]

ERK; mitogen-activated protein kinase 1/3 [EC:2.7.11.24]

MAPK7; mitogen-activated protein kinase 7 [EC:2.7.11.24]

RHOA; Ras homolog gene family, member A

GNAI; guanine nucleotide-binding protein G(i) subunit alpha

PPP3R; serine/threonine-protein phosphatase 2B regulatory subunit

PPP1C; serine/threonine-protein phosphatase PP1 catalytic subunit [EC:3.1.3.16]

PRKAB; 5'-AMP-activated protein kinase, regulatory beta subunit

PRKAG; 5'-AMP-activated protein kinase, regulatory gamma subunit

KRAS; GTPase KRas

CAMK1; calcium/calmodulin-dependent protein kinase I [EC:2.7.11.17]

114. Pantothenate and CoA biosynthesis

ilvC; ketol-acid reductoisomerase [EC:1.1.1.86]

panE; 2-dehydropantoate 2-reductase [EC:1.1.1.169]

panB; 3-methyl-2-oxobutanoate hydroxymethyltransferase [EC:2.1.2.11]

E2.6.1.42; branched-chain amino acid aminotransferase [EC:2.6.1.42]

coaE; dephospho-CoA kinase [EC:2.7.1.24]

PPCDC; phosphopantothenoylcysteine decarboxylase [EC:4.1.1.36]

E2.2.1.6L; acetolactate synthase I/II/III large subunit [EC:2.2.1.6]

E2.2.1.6S; acetolactate synthase I/III small subunit [EC:2.2.1.6]

ilvD; dihydroxy-acid dehydratase [EC:4.2.1.9]

panC; pantoate--beta-alanine ligase [EC:6.3.2.1]

PPCS; phosphopantothenate---cysteine ligase (ATP) [EC:6.3.2.51]

E2.7.7.3B; pantetheine-phosphate adenylyltransferase [EC:2.7.7.3]

LYS5; 4'-phosphopantetheinyl transferase [EC:2.7.8.-]

coaW; type II pantothenate kinase [EC:2.7.1.33]

115. Apelin signaling pathway

PIK3C3; phosphatidylinositol 3-kinase [EC:2.7.1.137]

CALM; calmodulin

PKA; protein kinase A [EC:2.7.11.11]

ERK; mitogen-activated protein kinase 1/3 [EC:2.7.11.24]

GNB1; guanine nucleotide-binding protein G(I)/G(S)/G(T) subunit beta-1

GNAI; guanine nucleotide-binding protein G(i) subunit alpha

SPHK; sphingosine kinase [EC:2.7.1.91]

PRKAB; 5'-AMP-activated protein kinase, regulatory beta subunit

PRKAG; 5'-AMP-activated protein kinase, regulatory gamma subunit

MTOR; serine/threonine-protein kinase mTOR [EC:2.7.11.1]

KRAS; GTPase KRas

PIK3R4; phosphoinositide-3-kinase, regulatory subunit 4 [EC:2.7.11.1]

BECN; beclin

GABARAP; GABA(A) receptor-associated protein

116. MicroRNAs in cancer

PTEN; phosphatidylinositol-3,4,5-trisphosphate 3-phosphatase and dual-specificity protein phosphatase PTEN [EC:3.1.3.16 3.1.3.48 3.1.3.67]

PRKCA; classical protein kinase C alpha type [EC:2.7.11.13]

SOS; son of sevenless

ERK; mitogen-activated protein kinase 1/3 [EC:2.7.11.24]

MAPK7; mitogen-activated protein kinase 7 [EC:2.7.11.24]

RHOA; Ras homolog gene family, member A

ATM; serine-protein kinase ATM [EC:2.7.11.1]

ABCB1; ATP-binding cassette, subfamily B (MDR/TAP), member 1 [EC:7.6.2.2]

ABCC1; ATP-binding cassette, subfamily C (CFTR/MRP), member 1 [EC:7.6.2.3]

HDAC1_2; histone deacetylase 1/2 [EC:3.5.1.98]

MTOR; serine/threonine-protein kinase mTOR [EC:2.7.11.1]

RAPTOR; regulatory associated protein of mTOR

KRAS; GTPase KRas

UBE2I; ubiquitin-conjugating enzyme E2 I

117. Vibrio cholerae infection

ATPeV1A; V-type H+-transporting ATPase subunit A [EC:7.1.2.2]

ATPeV0D; V-type H+-transporting ATPase subunit d

ATPeV1B; V-type H+-transporting ATPase subunit B

ATPeV1C; V-type H+-transporting ATPase subunit C

ATPeV1D; V-type H+-transporting ATPase subunit D

ATPeV1G; V-type H+-transporting ATPase subunit G

ATPeV0A; V-type H+-transporting ATPase subunit a

ATPeV0C; V-type H+-transporting ATPase 16kDa proteolipid subunit

PRKCA; classical protein kinase C alpha type [EC:2.7.11.13]

ATPeV0B; V-type H+-transporting ATPase 21kDa proteolipid subunit

PKA; protein kinase A [EC:2.7.11.11]

ARF1_2; ADP-ribosylation factor 1/2

KDELR; ER lumen protein retaining receptor

SEC61A; protein transport protein SEC61 subunit alpha

118. Neurotrophin signaling pathway

CALM; calmodulin

GSK3B; glycogen synthase kinase 3 beta [EC:2.7.11.26]

SOS; son of sevenless

ERK; mitogen-activated protein kinase 1/3 [EC:2.7.11.24]

RAC1; Ras-related C3 botulinum toxin substrate 1

CDC42; cell division control protein 42

P38; p38 MAP kinase [EC:2.7.11.24]

MAPK7; mitogen-activated protein kinase 7 [EC:2.7.11.24]

RHOA; Ras homolog gene family, member A

PDPK1; 3-phosphoinositide dependent protein kinase-1 [EC:2.7.11.1]

YWHAE; 14-3-3 protein epsilon

KRAS; GTPase KRas

RAP1B; Ras-related protein Rap-1B

ARHGDI; Rho GDP-dissociation inhibitor

119. Protein export

SRP19; signal recognition particle subunit SRP19

SRP54; signal recognition particle subunit SRP54 [EC:3.6.5.4]

SRP68; signal recognition particle subunit SRP68

SRP72; signal recognition particle subunit SRP72

yidC; YidC/Oxa1 family membrane protein insertase

SEC63; translocation protein SEC63

IMP1; mitochondrial inner membrane protease subunit 1 [EC:3.4.21.-]

IMP2; mitochondrial inner membrane protease subunit 2 [EC:3.4.21.-]

SEC61A; protein transport protein SEC61 subunit alpha

SRPRB; signal recognition particle receptor subunit beta

SEC62; translocation protein SEC62

SPCS3; signal peptidase complex subunit 3 [EC:3.4.-.-]

SEC11; signal peptidase I [EC:3.4.21.89]

SRPR; signal recognition particle receptor subunit alpha

120. Cytosolic DNA-sensing pathway

RPB10; DNA-directed RNA polymerases I, II, and III subunit RPABC5

RPB5; DNA-directed RNA polymerases I, II, and III subunit RPABC1

RPB6; DNA-directed RNA polymerases I, II, and III subunit RPABC2

RPB8; DNA-directed RNA polymerases I, II, and III subunit RPABC3

RPC1; DNA-directed RNA polymerase III subunit RPC1 [EC:2.7.7.6]

RPC19; DNA-directed RNA polymerases I and III subunit RPAC2

RPC2; DNA-directed RNA polymerase III subunit RPC2 [EC:2.7.7.6]

RPC8; DNA-directed RNA polymerase III subunit RPC8

RPC3; DNA-directed RNA polymerase III subunit RPC3

RPC7; DNA-directed RNA polymerase III subunit RPC7

RPC6; DNA-directed RNA polymerase III subunit RPC6

RPC4; DNA-directed RNA polymerase III subunit RPC4

RPC40; DNA-directed RNA polymerases I and III subunit RPAC1

RPC5; DNA-directed RNA polymerase III subunit RPC5

121. Sphingolipid metabolism

SPT; serine palmitoyltransferase [EC:2.3.1.50]

UGCG; ceramide glucosyltransferase [EC:2.4.1.80]

E3.1.6.1; arylsulfatase [EC:3.1.6.1]

lacZ; beta-galactosidase [EC:3.2.1.23]

SGPL1; sphinganine-1-phosphate aldolase [EC:4.1.2.27]

KDSR; 3-dehydrosphinganine reductase [EC:1.1.1.102]

LAG1; Acyl-CoA-dependent ceramide synthase [EC:2.3.1.24]

ACER3; dihydroceramidase [EC:3.5.1.-]

DEGS; sphingolipid 4-desaturase/C4-monooxygenase [EC:1.14.19.17 1.14.18.5]

SUR2; sphinganine C4-monooxygenase [EC:1.14.18.5]

SPHK; sphingosine kinase [EC:2.7.1.91]

E3.2.1.22B; alpha-galactosidase [EC:3.2.1.22]

SMPD2; sphingomyelin phosphodiesterase 2 [EC:3.1.4.12]

LCB3; dihydrosphingosine 1-phosphate phosphatase [EC:3.1.3.-]

122. Dopaminergic synapse

COMT; catechol O-methyltransferase [EC:2.1.1.6]

CALM; calmodulin

PRKCA; classical protein kinase C alpha type [EC:2.7.11.13]

GSK3B; glycogen synthase kinase 3 beta [EC:2.7.11.26]

PPP2R1; serine/threonine-protein phosphatase 2A regulatory subunit A

PKA; protein kinase A [EC:2.7.11.11]

PPP3C; serine/threonine-protein phosphatase 2B catalytic subunit [EC:3.1.3.16]

PPP2R2; serine/threonine-protein phosphatase 2A regulatory subunit B

P38; p38 MAP kinase [EC:2.7.11.24]

GNB1; guanine nucleotide-binding protein G(I)/G(S)/G(T) subunit beta-1

GNAI; guanine nucleotide-binding protein G(i) subunit alpha

PPP1C; serine/threonine-protein phosphatase PP1 catalytic subunit [EC:3.1.3.16]

KIF5; kinesin family member 5

PPP2R5; serine/threonine-protein phosphatase 2A regulatory subunit B'

123. Lysine degradation

ALDH; aldehyde dehydrogenase (NAD+) [EC:1.2.1.3]

gabD; succinate-semialdehyde dehydrogenase / glutarate-semialdehyde dehydrogenase [EC:1.2.1.16 1.2.1.79 1.2.1.20]

LYS1; saccharopine dehydrogenase (NAD+, L-lysine forming) [EC:1.5.1.7]

LYS9; saccharopine dehydrogenase (NADP+, L-glutamate forming) [EC:1.5.1.10]

PIPOX; sarcosine oxidase / L-pipecolate oxidase [EC:1.5.3.1 1.5.3.7]

DLD; dihydrolipoamide dehydrogenase [EC:1.8.1.4]

TMLHE; trimethyllysine dioxygenase [EC:1.14.11.8]

ACAT; acetyl-CoA C-acetyltransferase [EC:2.3.1.9]

DLST; 2-oxoglutarate dehydrogenase E2 component (dihydrolipoamide succinyltransferase) [EC:2.3.1.61]

SETD1; [histone H3]-lysine4 N-trimethyltransferase SETD1 [EC:2.1.1.354]

SMYD; [histone H3]-lysine4/36 N-trimethyltransferase SMYD [EC:2.1.1.354 2.1.1.357]

DOT1L; [histone H3]-lysine79 N-trimethyltransferase [EC:2.1.1.360]

CTM1; [cytochrome c]-lysine N-methyltransferase [EC:2.1.1.59]

SET2; [histone H3]-lysine36 N-trimethyltransferase [EC:2.1.1.359]

124. Tryptophan metabolism

ALDH; aldehyde dehydrogenase (NAD+) [EC:1.2.1.3]

DLD; dihydrolipoamide dehydrogenase [EC:1.8.1.4]

HAAO; 3-hydroxyanthranilate 3,4-dioxygenase [EC:1.13.11.6]

IDO; indoleamine 2,3-dioxygenase [EC:1.13.11.52]

KMO; kynurenine 3-monooxygenase [EC:1.14.13.9]

ACAT; acetyl-CoA C-acetyltransferase [EC:2.3.1.9]

DLST; 2-oxoglutarate dehydrogenase E2 component (dihydrolipoamide succinyltransferase) [EC:2.3.1.61]

ARO8; aromatic amino acid aminotransferase I / 2-aminoadipate transaminase [EC:2.6.1.57 2.6.1.39 2.6.1.27 2.6.1.5]

E3.5.1.4; amidase [EC:3.5.1.4]

E3.5.5.1; nitrilase [EC:3.5.5.1]

KYNU; kynureninase [EC:3.7.1.3]

katE; catalase [EC:1.11.1.6]

BNA7; kynurenine formamidase [EC:3.5.1.9]

BNA3; kynurenine aminotransferase [EC:2.6.1.7]

125. Phospholipase D signaling pathway

PIP5K; 1-phosphatidylinositol-4-phosphate 5-kinase [EC:2.7.1.68]

PLD1_2; phospholipase D1/2 [EC:3.1.4.4]

PRKCA; classical protein kinase C alpha type [EC:2.7.11.13]

SOS; son of sevenless

ERK; mitogen-activated protein kinase 1/3 [EC:2.7.11.24]

RHOA; Ras homolog gene family, member A

SPHK; sphingosine kinase [EC:2.7.1.91]

MTOR; serine/threonine-protein kinase mTOR [EC:2.7.11.1]

RHEB; Ras homolog enriched in brain

KRAS; GTPase KRas

ARF1_2; ADP-ribosylation factor 1/2

ARF6; ADP-ribosylation factor 6

AGPAT1_2; lysophosphatidate acyltransferase [EC:2.3.1.51]

126. Carbon fixation in photosynthetic organisms

MDH2; malate dehydrogenase [EC:1.1.1.37]

GAPDH; glyceraldehyde 3-phosphate dehydrogenase [EC:1.2.1.12]

E2.2.1.1; transketolase [EC:2.2.1.1]

GPT; alanine transaminase [EC:2.6.1.2]

PGK; phosphoglycerate kinase [EC:2.7.2.3]

E4.1.1.49; phosphoenolpyruvate carboxykinase (ATP) [EC:4.1.1.49]

FBA; fructose-bisphosphate aldolase, class II [EC:4.1.2.13]

rpe; ribulose-phosphate 3-epimerase [EC:5.1.3.1]

TPI; triosephosphate isomerase (TIM) [EC:5.3.1.1]

rpiA; ribose 5-phosphate isomerase A [EC:5.3.1.6]

FBP; fructose-1,6-bisphosphatase I [EC:3.1.3.11]

GOT1; aspartate aminotransferase, cytoplasmic [EC:2.6.1.1]

GOT2; aspartate aminotransferase, mitochondrial [EC:2.6.1.1]

127. Porphyrin and chlorophyll metabolism

PPOX; protoporphyrinogen/coproporphyrinogen III oxidase [EC:1.3.3.4 1.3.3.15]

MET1; uroporphyrin-III C-methyltransferase [EC:2.1.1.107]

E2.3.1.37; 5-aminolevulinate synthase [EC:2.3.1.37]

hemE; uroporphyrinogen decarboxylase [EC:4.1.1.37]

hemB; porphobilinogen synthase [EC:4.2.1.24]

hemD; uroporphyrinogen-III synthase [EC:4.2.1.75]

hemC; hydroxymethylbilane synthase [EC:2.5.1.61]

HCCS; cytochrome c heme-lyase [EC:4.4.1.17]

hemH; protoporphyrin/coproporphyrin ferrochelatase [EC:4.99.1.1 4.99.1.9]

EARS; glutamyl-tRNA synthetase [EC:6.1.1.17]

COX10; heme o synthase [EC:2.5.1.141]

COX15; cytochrome c oxidase assembly protein subunit 15

FXN; frataxin [EC:1.16.3.1]

128. Propanoate metabolism

mmsA; malonate-semialdehyde dehydrogenase (acetylating) / methylmalonate-semialdehyde dehydrogenase [EC:1.2.1.18 1.2.1.27]

E1.3.3.6; acyl-CoA oxidase [EC:1.3.3.6]

DLD; dihydrolipoamide dehydrogenase [EC:1.8.1.4]

ACAT; acetyl-CoA C-acetyltransferase [EC:2.3.1.9]

ACSS1_2; acetyl-CoA synthetase [EC:6.2.1.1]

LSC1; succinyl-CoA synthetase alpha subunit [EC:6.2.1.4 6.2.1.5]

LSC2; succinyl-CoA synthetase beta subunit [EC:6.2.1.4 6.2.1.5]

HIBCH; 3-hydroxyisobutyryl-CoA hydrolase [EC:3.1.2.4]

ACACA; acetyl-CoA carboxylase / biotin carboxylase 1 [EC:6.4.1.2 6.3.4.14 2.1.3.15]

ABAT; 4-aminobutyrate aminotransferase / (S)-3-amino-2-methylpropionate transaminase [EC:2.6.1.19 2.6.1.22]

FOX2; multifunctional beta-oxidation protein [EC:4.2.1.- 1.1.1.-]

GRE2; NADPH-dependent methylglyoxal reductase [EC:1.1.1.283]

HPD1; 3-hydroxyisobutyrate/3-hydroxypropionate dehydrogenase [EC:1.1.1.31 1.1.1.59]

129. Focal adhesion

PIP5K; 1-phosphatidylinositol-4-phosphate 5-kinase [EC:2.7.1.68]

PTEN; phosphatidylinositol-3,4,5-trisphosphate 3-phosphatase and dual-specificity protein phosphatase PTEN [EC:3.1.3.16 3.1.3.48 3.1.3.67]

PRKCA; classical protein kinase C alpha type [EC:2.7.11.13]

GSK3B; glycogen synthase kinase 3 beta [EC:2.7.11.26]

SOS; son of sevenless

ERK; mitogen-activated protein kinase 1/3 [EC:2.7.11.24]

RAC1; Ras-related C3 botulinum toxin substrate 1

CDC42; cell division control protein 42

PAK1; p21-activated kinase 1 [EC:2.7.11.1]

RHOA; Ras homolog gene family, member A

PPP1C; serine/threonine-protein phosphatase PP1 catalytic subunit [EC:3.1.3.16]

PDPK1; 3-phosphoinositide dependent protein kinase-1 [EC:2.7.11.1]

RAP1B; Ras-related protein Rap-1B

130. Epithelial cell signaling in Helicobacter pylori infection

ATPeV1A; V-type H+-transporting ATPase subunit A [EC:7.1.2.2]

ATPeV0D; V-type H+-transporting ATPase subunit d

ATPeV1B; V-type H+-transporting ATPase subunit B

ATPeV1C; V-type H+-transporting ATPase subunit C

ATPeV1D; V-type H+-transporting ATPase subunit D

ATPeV1G; V-type H+-transporting ATPase subunit G

ATPeV0A; V-type H+-transporting ATPase subunit a

ATPeV0C; V-type H+-transporting ATPase 16kDa proteolipid subunit

ATPeV0B; V-type H+-transporting ATPase 21kDa proteolipid subunit

RAC1; Ras-related C3 botulinum toxin substrate 1

CDC42; cell division control protein 42

PAK1; p21-activated kinase 1 [EC:2.7.11.1]

P38; p38 MAP kinase [EC:2.7.11.24]

131. Longevity regulating pathway

EIF4E; translation initiation factor 4E

katE; catalase [EC:1.11.1.6]

PKA; protein kinase A [EC:2.7.11.11]

SOD2; superoxide dismutase, Fe-Mn family [EC:1.15.1.1]

PRKAB; 5'-AMP-activated protein kinase, regulatory beta subunit

PRKAG; 5'-AMP-activated protein kinase, regulatory gamma subunit

MTOR; serine/threonine-protein kinase mTOR [EC:2.7.11.1]

RAPTOR; regulatory associated protein of mTOR

RHEB; Ras homolog enriched in brain

ADIPOR; adiponectin receptor

KRAS; GTPase KRas

ATG13; autophagy-related protein 13

ATG5; autophagy-related protein 5

132. HIF-1 signaling pathway

GAPDH; glyceraldehyde 3-phosphate dehydrogenase [EC:1.2.1.12]

PDHA; pyruvate dehydrogenase E1 component alpha subunit [EC:1.2.4.1]

PDHB; pyruvate dehydrogenase E1 component beta subunit [EC:1.2.4.1]

HK; hexokinase [EC:2.7.1.1]

pfkA; 6-phosphofructokinase 1 [EC:2.7.1.11]

PGK; phosphoglycerate kinase [EC:2.7.2.3]

ENO; enolase [EC:4.2.1.11]

PRKCA; classical protein kinase C alpha type [EC:2.7.11.13]

EIF4E; translation initiation factor 4E

ELOC; elongin-C

ERK; mitogen-activated protein kinase 1/3 [EC:2.7.11.24]

MTOR; serine/threonine-protein kinase mTOR [EC:2.7.11.1]

PDK1; pyruvate dehydrogenase kinase isoform 1 [EC:2.7.11.2]

133. Wnt signaling pathway

PRKCA; classical protein kinase C alpha type [EC:2.7.11.13]

GSK3B; glycogen synthase kinase 3 beta [EC:2.7.11.26]

SKP1; S-phase kinase-associated protein 1

CSNK2A; casein kinase II subunit alpha [EC:2.7.11.1]

CSNK2B; casein kinase II subunit beta

CUL1; cullin 1

PKA; protein kinase A [EC:2.7.11.11]

PPP3C; serine/threonine-protein phosphatase 2B catalytic subunit [EC:3.1.3.16]

RAC1; Ras-related C3 botulinum toxin substrate 1

RUVBL1; RuvB-like protein 1 (pontin 52)

TBL1; transducin (beta)-like 1

RHOA; Ras homolog gene family, member A

PPP3R; serine/threonine-protein phosphatase 2B regulatory subunit

134. Tyrosine metabolism

frmA; S-(hydroxymethyl)glutathione dehydrogenase / alcohol dehydrogenase [EC:1.1.1.284 1.1.1.1]

E1.2.1.5; aldehyde dehydrogenase (NAD(P)+) [EC:1.2.1.5]

gabD; succinate-semialdehyde dehydrogenase / glutarate-semialdehyde dehydrogenase [EC:1.2.1.16 1.2.1.79 1.2.1.20]

AOC3; primary-amine oxidase [EC:1.4.3.21]

COMT; catechol O-methyltransferase [EC:2.1.1.6]

hisC; histidinol-phosphate aminotransferase [EC:2.6.1.9]

ARO8; aromatic amino acid aminotransferase I / 2-aminoadipate transaminase [EC:2.6.1.57 2.6.1.39 2.6.1.27 2.6.1.5]

FAHD1; acylpyruvate hydrolase [EC:3.7.1.5]

ARO9; aromatic amino acid aminotransferase II [EC:2.6.1.58 2.6.1.28]

adhP; alcohol dehydrogenase, propanol-preferring [EC:1.1.1.1]

GOT1; aspartate aminotransferase, cytoplasmic [EC:2.6.1.1]

GOT2; aspartate aminotransferase, mitochondrial [EC:2.6.1.1]

135. Lysine biosynthesis

hom; homoserine dehydrogenase [EC:1.1.1.3]

asd; aspartate-semialdehyde dehydrogenase [EC:1.2.1.11]

LYS2; L-2-aminoadipate reductase [EC:1.2.1.95]

LYS1; saccharopine dehydrogenase (NAD+, L-lysine forming) [EC:1.5.1.7]

LYS9; saccharopine dehydrogenase (NADP+, L-glutamate forming) [EC:1.5.1.10]

ARO8; aromatic amino acid aminotransferase I / 2-aminoadipate transaminase [EC:2.6.1.57 2.6.1.39 2.6.1.27 2.6.1.5]

lysC; aspartate kinase [EC:2.7.2.4]

LYS21; homocitrate synthase [EC:2.3.3.14]

LYS4; homoaconitate hydratase [EC:4.2.1.36]

dapA; 4-hydroxy-tetrahydrodipicolinate synthase [EC:4.3.3.7]

LYS12; homoisocitrate dehydrogenase [EC:1.1.1.87]

ACO2; homoaconitase [EC:4.2.1.-]

136. Central carbon metabolism in cancer

G6PD; glucose-6-phosphate 1-dehydrogenase [EC:1.1.1.49 1.1.1.363]

PDHA; pyruvate dehydrogenase E1 component alpha subunit [EC:1.2.4.1]

PDHB; pyruvate dehydrogenase E1 component beta subunit [EC:1.2.4.1]

HK; hexokinase [EC:2.7.1.1]

pfkA; 6-phosphofructokinase 1 [EC:2.7.1.11]

PK; pyruvate kinase [EC:2.7.1.40]

PTEN; phosphatidylinositol-3,4,5-trisphosphate 3-phosphatase and dual-specificity protein phosphatase PTEN [EC:3.1.3.16 3.1.3.48 3.1.3.67]

PGAM; 2,3-bisphosphoglycerate-dependent phosphoglycerate mutase [EC:5.4.2.11]

ERK; mitogen-activated protein kinase 1/3 [EC:2.7.11.24]

MTOR; serine/threonine-protein kinase mTOR [EC:2.7.11.1]

KRAS; GTPase KRas

PDK1; pyruvate dehydrogenase kinase isoform 1 [EC:2.7.11.2]

137. Colorectal cancer

GSK3B; glycogen synthase kinase 3 beta [EC:2.7.11.26]

SOS; son of sevenless

ERK; mitogen-activated protein kinase 1/3 [EC:2.7.11.24]

RAC1; Ras-related C3 botulinum toxin substrate 1

RHOA; Ras homolog gene family, member A

MTOR; serine/threonine-protein kinase mTOR [EC:2.7.11.1]

KRAS; GTPase KRas

MLH1; DNA mismatch repair protein MLH1

MSH2; DNA mismatch repair protein MSH2

MSH3; DNA mismatch repair protein MSH3

MSH6; DNA mismatch repair protein MSH6

CYC; cytochrome c

138. Herpes simplex virus 1 infection

EIF2S1; translation initiation factor 2 subunit 1

EIF2B1; translation initiation factor eIF-2B subunit alpha

EIF2B5; translation initiation factor eIF-2B subunit epsilon

EIF2B3; translation initiation factor eIF-2B subunit gamma

EIF2B4; translation initiation factor eIF-2B subunit delta

EIF2B2; translation initiation factor eIF-2B subunit beta

PPP1C; serine/threonine-protein phosphatase PP1 catalytic subunit [EC:3.1.3.16]

MTOR; serine/threonine-protein kinase mTOR [EC:2.7.11.1]

RHEB; Ras homolog enriched in brain

CYC; cytochrome c

NXF; nuclear RNA export factor

EIF2AK4; eukaryotic translation initiation factor 2-alpha kinase 4 [EC:2.7.11.1]

139. beta-Alanine metabolism

ALDH; aldehyde dehydrogenase (NAD+) [EC:1.2.1.3]

E1.2.1.5; aldehyde dehydrogenase (NAD(P)+) [EC:1.2.1.5]

mmsA; malonate-semialdehyde dehydrogenase (acetylating) / methylmalonate-semialdehyde dehydrogenase [EC:1.2.1.18 1.2.1.27]

E1.3.3.6; acyl-CoA oxidase [EC:1.3.3.6]

AOC3; primary-amine oxidase [EC:1.4.3.21]

E4.1.1.15; glutamate decarboxylase [EC:4.1.1.15]

panC; pantoate--beta-alanine ligase [EC:6.3.2.1]

HIBCH; 3-hydroxyisobutyryl-CoA hydrolase [EC:3.1.2.4]

FMS1; polyamine oxidase [EC:1.5.3.17]

ABAT; 4-aminobutyrate aminotransferase / (S)-3-amino-2-methylpropionate transaminase [EC:2.6.1.19 2.6.1.22]

FOX2; multifunctional beta-oxidation protein [EC:4.2.1.- 1.1.1.-]

HPD1; 3-hydroxyisobutyrate/3-hydroxypropionate dehydrogenase [EC:1.1.1.31 1.1.1.59]

140. Axon guidance

PRKCA; classical protein kinase C alpha type [EC:2.7.11.13]

GSK3B; glycogen synthase kinase 3 beta [EC:2.7.11.26]

PPP3C; serine/threonine-protein phosphatase 2B catalytic subunit [EC:3.1.3.16]

ERK; mitogen-activated protein kinase 1/3 [EC:2.7.11.24]

RAC1; Ras-related C3 botulinum toxin substrate 1

CDC42; cell division control protein 42

PAK1; p21-activated kinase 1 [EC:2.7.11.1]

RHOA; Ras homolog gene family, member A

GNAI; guanine nucleotide-binding protein G(i) subunit alpha

PPP3R; serine/threonine-protein phosphatase 2B regulatory subunit

KRAS; GTPase KRas

PDK1; pyruvate dehydrogenase kinase isoform 1 [EC:2.7.11.2]

141. NOD-like receptor signaling pathway

trxA; thioredoxin 1

HSP90A; molecular chaperone HtpG

ERK; mitogen-activated protein kinase 1/3 [EC:2.7.11.24]

P38; p38 MAP kinase [EC:2.7.11.24]

RHOA; Ras homolog gene family, member A

MFN2; mitofusin 2 [EC:3.6.5.-]

YWHAE; 14-3-3 protein epsilon

ATG5; autophagy-related protein 5

GABARAP; GABA(A) receptor-associated protein

SUGT1; suppressor of G2 allele of SKP1

VDAC2; voltage-dependent anion channel protein 2

DNM1L; dynamin 1-like protein [EC:3.6.5.5]

142. Chemokine signaling pathway

GSK3B; glycogen synthase kinase 3 beta [EC:2.7.11.26]

SOS; son of sevenless

PKA; protein kinase A [EC:2.7.11.11]

ERK; mitogen-activated protein kinase 1/3 [EC:2.7.11.24]

RAC1; Ras-related C3 botulinum toxin substrate 1

CDC42; cell division control protein 42

PAK1; p21-activated kinase 1 [EC:2.7.11.1]

RHOA; Ras homolog gene family, member A

GNB1; guanine nucleotide-binding protein G(I)/G(S)/G(T) subunit beta-1

GNAI; guanine nucleotide-binding protein G(i) subunit alpha

KRAS; GTPase KRas

RAP1B; Ras-related protein Rap-1B

143. Galactose metabolism

HK; hexokinase [EC:2.7.1.1]

galK; galactokinase [EC:2.7.1.6]

pfkA; 6-phosphofructokinase 1 [EC:2.7.1.11]

UGP2; UTP--glucose-1-phosphate uridylyltransferase [EC:2.7.7.9]

galT; UDPglucose--hexose-1-phosphate uridylyltransferase [EC:2.7.7.12]

IMA; oligo-1,6-glucosidase [EC:3.2.1.10]

malZ; alpha-glucosidase [EC:3.2.1.20]

lacZ; beta-galactosidase [EC:3.2.1.23]

INV; beta-fructofuranosidase [EC:3.2.1.26]

galM; aldose 1-epimerase [EC:5.1.3.3]

pgm; phosphoglucomutase [EC:5.4.2.2]

E3.2.1.22B; alpha-galactosidase [EC:3.2.1.22]

144. Phenylalanine metabolism

E1.2.1.5; aldehyde dehydrogenase (NAD(P)+) [EC:1.2.1.5]

AOC3; primary-amine oxidase [EC:1.4.3.21]

hisC; histidinol-phosphate aminotransferase [EC:2.6.1.9]

ARO8; aromatic amino acid aminotransferase I / 2-aminoadipate transaminase [EC:2.6.1.57 2.6.1.39 2.6.1.27 2.6.1.5]

E3.5.1.4; amidase [EC:3.5.1.4]

ARO9; aromatic amino acid aminotransferase II [EC:2.6.1.58 2.6.1.28]

PHAA; phenylacetate 2-hydroxylase [EC:1.14.14.54]

ARO10; phenylpyruvate decarboxylase [EC:4.1.1.-]

GOT1; aspartate aminotransferase, cytoplasmic [EC:2.6.1.1]

GOT2; aspartate aminotransferase, mitochondrial [EC:2.6.1.1]

HPA3; D-amino-acid N-acetyltransferase [EC:2.3.1.36]

145. Choline metabolism in cancer

PIP5K; 1-phosphatidylinositol-4-phosphate 5-kinase [EC:2.7.1.68]

PCYT1; choline-phosphate cytidylyltransferase [EC:2.7.7.15]

PLD1_2; phospholipase D1/2 [EC:3.1.4.4]

PRKCA; classical protein kinase C alpha type [EC:2.7.11.13]

SOS; son of sevenless

ERK; mitogen-activated protein kinase 1/3 [EC:2.7.11.24]

RAC1; Ras-related C3 botulinum toxin substrate 1

PDPK1; 3-phosphoinositide dependent protein kinase-1 [EC:2.7.11.1]

MTOR; serine/threonine-protein kinase mTOR [EC:2.7.11.1]

RHEB; Ras homolog enriched in brain

KRAS; GTPase KRas

146. Valine, leucine and isoleucine degradation

ALDH; aldehyde dehydrogenase (NAD+) [EC:1.2.1.3]

mmsA; malonate-semialdehyde dehydrogenase (acetylating) / methylmalonate-semialdehyde dehydrogenase [EC:1.2.1.18 1.2.1.27]

ACADM; acyl-CoA dehydrogenase [EC:1.3.8.7]

DLD; dihydrolipoamide dehydrogenase [EC:1.8.1.4]

ACAT; acetyl-CoA C-acetyltransferase [EC:2.3.1.9]

E2.6.1.42; branched-chain amino acid aminotransferase [EC:2.6.1.42]

E2.3.3.10; hydroxymethylglutaryl-CoA synthase [EC:2.3.3.10]

HIBCH; 3-hydroxyisobutyryl-CoA hydrolase [EC:3.1.2.4]

ACAA1; acetyl-CoA acyltransferase 1 [EC:2.3.1.16]

ABAT; 4-aminobutyrate aminotransferase / (S)-3-amino-2-methylpropionate transaminase [EC:2.6.1.19 2.6.1.22]

HPD1; 3-hydroxyisobutyrate/3-hydroxypropionate dehydrogenase [EC:1.1.1.31 1.1.1.59]

147. T cell receptor signaling pathway

GSK3B; glycogen synthase kinase 3 beta [EC:2.7.11.26]

SOS; son of sevenless

PPP3C; serine/threonine-protein phosphatase 2B catalytic subunit [EC:3.1.3.16]

ERK; mitogen-activated protein kinase 1/3 [EC:2.7.11.24]

CDC42; cell division control protein 42

PAK1; p21-activated kinase 1 [EC:2.7.11.1]

P38; p38 MAP kinase [EC:2.7.11.24]

RHOA; Ras homolog gene family, member A

PPP3R; serine/threonine-protein phosphatase 2B regulatory subunit

PDPK1; 3-phosphoinositide dependent protein kinase-1 [EC:2.7.11.1]

KRAS; GTPase KRas

148. Sulfur metabolism

cysJ; sulfite reductase (NADPH) flavoprotein alpha-component [EC:1.8.1.2]

cysI; sulfite reductase (NADPH) hemoprotein beta-component [EC:1.8.1.2]

cysH; phosphoadenosine phosphosulfate reductase [EC:1.8.4.8 1.8.4.10]

metX; homoserine O-acetyltransferase/O-succinyltransferase [EC:2.3.1.31 2.3.1.46]

cysC; adenylylsulfate kinase [EC:2.7.1.25]

sat; sulfate adenylyltransferase [EC:2.7.7.4]

APA1_2; sulfate adenylyltransferase (ADP) / ATP adenylyltransferase [EC:2.7.7.5 2.7.7.53]

cysQ; 3'(2'), 5'-bisphosphate nucleotidase [EC:3.1.3.7]

cysK; cysteine synthase [EC:2.5.1.47]

metB; cystathionine gamma-synthase [EC:2.5.1.48]

MET17; O-acetylhomoserine/O-acetylserine sulfhydrylase [EC:2.5.1.49 2.5.1.47]

149. Hepatitis C

GSK3B; glycogen synthase kinase 3 beta [EC:2.7.11.26]

SOS; son of sevenless

EIF2S1; translation initiation factor 2 subunit 1

PPP2R1; serine/threonine-protein phosphatase 2A regulatory subunit A

PPP2R2; serine/threonine-protein phosphatase 2A regulatory subunit B

ERK; mitogen-activated protein kinase 1/3 [EC:2.7.11.24]

PIAS1; E3 SUMO-protein ligase PIAS1 [EC:2.3.2.-]

YWHAE; 14-3-3 protein epsilon

KRAS; GTPase KRas

CYC; cytochrome c

EIF2AK4; eukaryotic translation initiation factor 2-alpha kinase 4 [EC:2.7.11.1]

150. Cardiac muscle contraction

UQCRFS1; ubiquinol-cytochrome c reductase iron-sulfur subunit [EC:7.1.1.8]

CYC1; ubiquinol-cytochrome c reductase cytochrome c1 subunit

QCR1; ubiquinol-cytochrome c reductase core subunit 1

QCR2; ubiquinol-cytochrome c reductase core subunit 2

QCR7; ubiquinol-cytochrome c reductase subunit 7

QCR8; ubiquinol-cytochrome c reductase subunit 8

COX5B; cytochrome c oxidase subunit 5b

COX6A; cytochrome c oxidase subunit 6a

COX6B; cytochrome c oxidase subunit 6b

COX7C; cytochrome c oxidase subunit 7c

SLC9A6_7; solute carrier family 9 (sodium/hydrogen exchanger), member 6/7

151. Bacterial invasion of epithelial cells

RAC1; Ras-related C3 botulinum toxin substrate 1

CDC42; cell division control protein 42

RHOA; Ras homolog gene family, member A

CLTC; clathrin heavy chain

ARPC5; actin related protein 2/3 complex, subunit 5

ARPC4; actin related protein 2/3 complex, subunit 4

ARPC3; actin related protein 2/3 complex, subunit 3

ARPC1A_B; actin related protein 2/3 complex, subunit 1A/1B

ARPC2; actin related protein 2/3 complex, subunit 2

SEPT3_9_12; septin 3/9/12

WASL; neural Wistt-Aldrich syndrome protein

152. Yersinia infection

PIP5K; 1-phosphatidylinositol-4-phosphate 5-kinase [EC:2.7.1.68]

GSK3B; glycogen synthase kinase 3 beta [EC:2.7.11.26]

ERK; mitogen-activated protein kinase 1/3 [EC:2.7.11.24]

RAC1; Ras-related C3 botulinum toxin substrate 1

CDC42; cell division control protein 42

P38; p38 MAP kinase [EC:2.7.11.24]

RHOA; Ras homolog gene family, member A

ARF6; ADP-ribosylation factor 6

ACTR2; actin-related protein 2

ACTR3; actin-related protein 3

WASL; neural Wistt-Aldrich syndrome protein

153. cAMP signaling pathway

E1.3.3.6; acyl-CoA oxidase [EC:1.3.3.6]

PLD1_2; phospholipase D1/2 [EC:3.1.4.4]

CALM; calmodulin

PKA; protein kinase A [EC:2.7.11.11]

ERK; mitogen-activated protein kinase 1/3 [EC:2.7.11.24]

RAC1; Ras-related C3 botulinum toxin substrate 1

PAK1; p21-activated kinase 1 [EC:2.7.11.1]

RHOA; Ras homolog gene family, member A

GNAI; guanine nucleotide-binding protein G(i) subunit alpha

PPP1C; serine/threonine-protein phosphatase PP1 catalytic subunit [EC:3.1.3.16]

RAP1B; Ras-related protein Rap-1B

154. Insulin resistance

PYG; glycogen phosphorylase [EC:2.4.1.1]

GYS; glycogen synthase [EC:2.4.1.11]

glmS; glutamine---fructose-6-phosphate transaminase (isomerizing) [EC:2.6.1.16]

PTEN; phosphatidylinositol-3,4,5-trisphosphate 3-phosphatase and dual-specificity protein phosphatase PTEN [EC:3.1.3.16 3.1.3.48 3.1.3.67]

GSK3B; glycogen synthase kinase 3 beta [EC:2.7.11.26]

PPP1C; serine/threonine-protein phosphatase PP1 catalytic subunit [EC:3.1.3.16]

PDPK1; 3-phosphoinositide dependent protein kinase-1 [EC:2.7.11.1]

PRKAB; 5'-AMP-activated protein kinase, regulatory beta subunit

PRKAG; 5'-AMP-activated protein kinase, regulatory gamma subunit

MTOR; serine/threonine-protein kinase mTOR [EC:2.7.11.1]

PPP2R4; serine/threonine-protein phosphatase 2A activator

155. Apoptosis - fly

EGD1; nascent polypeptide-associated complex subunit beta

EIF5; translation initiation factor 5

PRDX2_4; peroxiredoxin 2/4 [EC:1.11.1.24]

ERK; mitogen-activated protein kinase 1/3 [EC:2.7.11.24]

ATM; serine-protein kinase ATM [EC:2.7.11.1]

MFN2; mitofusin 2 [EC:3.6.5.-]

KRAS; GTPase KRas

RHOT1; mitochondrial Rho GTPase 1 [EC:3.6.5.-]

CYC; cytochrome c

DNM1L; dynamin 1-like protein [EC:3.6.5.5]

156. Histidine metabolism

ALDH; aldehyde dehydrogenase (NAD+) [EC:1.2.1.3]

E1.2.1.5; aldehyde dehydrogenase (NAD(P)+) [EC:1.2.1.5]

hisC; histidinol-phosphate aminotransferase [EC:2.6.1.9]

HIS7; imidazole glycerol-phosphate synthase [EC:4.3.2.10]

hisB; imidazoleglycerol-phosphate dehydratase [EC:4.2.1.19]

hisA; phosphoribosylformimino-5-aminoimidazole carboxamide ribotide isomerase [EC:5.3.1.16]

E3.1.3.15B; histidinol-phosphatase (PHP family) [EC:3.1.3.15]

HIS4; phosphoribosyl-ATP pyrophosphohydrolase / phosphoribosyl-AMP cyclohydrolase / histidinol dehydrogenase [EC:3.6.1.31 3.5.4.19 1.1.1.23]

CARNMT1; carnosine N-methyltransferase [EC:2.1.1.22]

EGT2; hercynylcysteine S-oxide lyase [EC:4.4.1.36]

157. GnRH signaling pathway

PLD1_2; phospholipase D1/2 [EC:3.1.4.4]

CALM; calmodulin

PRKCA; classical protein kinase C alpha type [EC:2.7.11.13]

SOS; son of sevenless

PKA; protein kinase A [EC:2.7.11.11]

ERK; mitogen-activated protein kinase 1/3 [EC:2.7.11.24]

CDC42; cell division control protein 42

P38; p38 MAP kinase [EC:2.7.11.24]

MAPK7; mitogen-activated protein kinase 7 [EC:2.7.11.24]

KRAS; GTPase KRas

158. Valine, leucine and isoleucine biosynthesis

leuB; 3-isopropylmalate dehydrogenase [EC:1.1.1.85]

ilvC; ketol-acid reductoisomerase [EC:1.1.1.86]

E2.6.1.42; branched-chain amino acid aminotransferase [EC:2.6.1.42]

leuA; 2-isopropylmalate synthase [EC:2.3.3.13]

E2.2.1.6L; acetolactate synthase I/II/III large subunit [EC:2.2.1.6]

E2.2.1.6S; acetolactate synthase I/III small subunit [EC:2.2.1.6]

ilvD; dihydroxy-acid dehydratase [EC:4.2.1.9]

LEU1; 3-isopropylmalate dehydratase [EC:4.2.1.33]

E4.3.1.19; threonine dehydratase [EC:4.3.1.19]

SDS; L-serine/L-threonine ammonia-lyase [EC:4.3.1.17 4.3.1.19]

159. Adrenergic signaling in cardiomyocytes

CALM; calmodulin

PRKCA; classical protein kinase C alpha type [EC:2.7.11.13]

PPP2R1; serine/threonine-protein phosphatase 2A regulatory subunit A

PKA; protein kinase A [EC:2.7.11.11]

PPP2R2; serine/threonine-protein phosphatase 2A regulatory subunit B

ERK; mitogen-activated protein kinase 1/3 [EC:2.7.11.24]

P38; p38 MAP kinase [EC:2.7.11.24]

GNAI; guanine nucleotide-binding protein G(i) subunit alpha

PPP1C; serine/threonine-protein phosphatase PP1 catalytic subunit [EC:3.1.3.16]

PPP2R5; serine/threonine-protein phosphatase 2A regulatory subunit B'

160. Biosynthesis of unsaturated fatty acids

E1.3.3.6; acyl-CoA oxidase [EC:1.3.3.6]

SCD; stearoyl-CoA desaturase (Delta-9 desaturase) [EC:1.14.19.1]

ACAA1; acetyl-CoA acyltransferase 1 [EC:2.3.1.16]

ELO2; fatty acid elongase 2 [EC:2.3.1.199]

ELO3; fatty acid elongase 3 [EC:2.3.1.199]

HSD17B12; 17beta-estradiol 17-dehydrogenase / very-long-chain 3-oxoacyl-CoA reductase [EC:1.1.1.62 1.1.1.330]

FAD2; omega-6 fatty acid desaturase / acyl-lipid omega-6 desaturase (Delta-12 desaturase) [EC:1.14.19.6 1.14.19.22]

TER; very-long-chain enoyl-CoA reductase [EC:1.3.1.93]

HACD; very-long-chain (3R)-3-hydroxyacyl-CoA dehydratase [EC:4.2.1.134]

FAD3; omega-3 fatty acid desaturase (Delta-15 desaturase) [EC:1.14.19.-]

161. Thiamine metabolism

THI20; hydroxymethylpyrimidine/phosphomethylpyrimidine kinase / thiaminase [EC:2.7.1.49 2.7.4.7 3.5.99.2]

adk; adenylate kinase [EC:2.7.4.3]

thiN; thiamine pyrophosphokinase [EC:2.7.6.2]

E3.1.3.1; alkaline phosphatase [EC:3.1.3.1]

PHO; acid phosphatase [EC:3.1.3.2]

THI4; cysteine-dependent adenosine diphosphate thiazole synthase [EC:2.4.2.60]

iscS; cysteine desulfurase [EC:2.8.1.7]

THI6; thiamine-phosphate diphosphorylase / hydroxyethylthiazole kinase [EC:2.5.1.3 2.7.1.50]

ACP1; low molecular weight phosphotyrosine protein phosphatase [EC:3.1.3.2 3.1.3.48]

THI5; pyrimidine precursor biosynthesis enzyme

162. Calcium signaling pathway

CALM; calmodulin

PRKCA; classical protein kinase C alpha type [EC:2.7.11.13]

PKA; protein kinase A [EC:2.7.11.11]

PPP3C; serine/threonine-protein phosphatase 2B catalytic subunit [EC:3.1.3.16]

SPHK; sphingosine kinase [EC:2.7.1.91]

PLCD; phosphatidylinositol phospholipase C, delta [EC:3.1.4.11]

SLC25A4S; solute carrier family 25 (mitochondrial adenine nucleotide translocator), member 4/5/6/31

PPP3R; serine/threonine-protein phosphatase 2B regulatory subunit

CAMK1; calcium/calmodulin-dependent protein kinase I [EC:2.7.11.17]

VDAC2; voltage-dependent anion channel protein 2

163. Rap1 signaling pathway

CALM; calmodulin

PRKCA; classical protein kinase C alpha type [EC:2.7.11.13]

ERK; mitogen-activated protein kinase 1/3 [EC:2.7.11.24]

RAC1; Ras-related C3 botulinum toxin substrate 1

CDC42; cell division control protein 42

P38; p38 MAP kinase [EC:2.7.11.24]

RHOA; Ras homolog gene family, member A

GNAI; guanine nucleotide-binding protein G(i) subunit alpha

KRAS; GTPase KRas

RAP1B; Ras-related protein Rap-1B

164. Drug metabolism - other enzymes

IMPDH; IMP dehydrogenase [EC:1.1.1.205]

GST; glutathione S-transferase [EC:2.5.1.18]

udk; uridine kinase [EC:2.7.1.48]

ndk; nucleoside-diphosphate kinase [EC:2.7.4.6]

ITPA; inosine triphosphate pyrophosphatase [EC:3.6.1.-]

dut; dUTP pyrophosphatase [EC:3.6.1.23]

guaA; GMP synthase (glutamine-hydrolysing) [EC:6.3.5.2]

RRM1; ribonucleoside-diphosphate reductase subunit M1 [EC:1.17.4.1]

RRM2; ribonucleoside-diphosphate reductase subunit M2 [EC:1.17.4.1]

CMPK1; UMP-CMP kinase [EC:2.7.4.14]

165. Long-term potentiation

CALM; calmodulin

PRKCA; classical protein kinase C alpha type [EC:2.7.11.13]

PKA; protein kinase A [EC:2.7.11.11]

PPP3C; serine/threonine-protein phosphatase 2B catalytic subunit [EC:3.1.3.16]

ERK; mitogen-activated protein kinase 1/3 [EC:2.7.11.24]

PPP3R; serine/threonine-protein phosphatase 2B regulatory subunit

PPP1C; serine/threonine-protein phosphatase PP1 catalytic subunit [EC:3.1.3.16]

KRAS; GTPase KRas

RAP1B; Ras-related protein Rap-1B

166. Riboflavin metabolism

ribE; riboflavin synthase [EC:2.5.1.9]

RFK; riboflavin kinase [EC:2.7.1.26]

FLAD1; FAD synthetase [EC:2.7.7.2]

PHO; acid phosphatase [EC:3.1.3.2]

ribA; GTP cyclohydrolase II [EC:3.5.4.25]

ribB; 3,4-dihydroxy 2-butanone 4-phosphate synthase [EC:4.1.99.12]

ACP1; low molecular weight phosphotyrosine protein phosphatase [EC:3.1.3.2 3.1.3.48]

RIB7; 2,5-diamino-6-(ribosylamino)-4(3H)-pyrimidinone 5'-phosphate reductase [EC:1.1.1.302]

RIB2; tRNA pseudouridine32 synthase / 2,5-diamino-6-(5-phospho-D-ribitylamino)-pyrimidin-4(3H)-one deaminase [EC:5.4.99.28]

167. Growth hormone synthesis, secretion and action

PRKCA; classical protein kinase C alpha type [EC:2.7.11.13]

GSK3B; glycogen synthase kinase 3 beta [EC:2.7.11.26]

SOS; son of sevenless

PKA; protein kinase A [EC:2.7.11.11]

ERK; mitogen-activated protein kinase 1/3 [EC:2.7.11.24]

P38; p38 MAP kinase [EC:2.7.11.24]

GNAI; guanine nucleotide-binding protein G(i) subunit alpha

MTOR; serine/threonine-protein kinase mTOR [EC:2.7.11.1]

KRAS; GTPase KRas

168. cGMP-PKG signaling pathway

CALM; calmodulin

PPP3C; serine/threonine-protein phosphatase 2B catalytic subunit [EC:3.1.3.16]

ERK; mitogen-activated protein kinase 1/3 [EC:2.7.11.24]

RHOA; Ras homolog gene family, member A

GNAI; guanine nucleotide-binding protein G(i) subunit alpha

SLC25A4S; solute carrier family 25 (mitochondrial adenine nucleotide translocator), member 4/5/6/31

PPP3R; serine/threonine-protein phosphatase 2B regulatory subunit

PPP1C; serine/threonine-protein phosphatase PP1 catalytic subunit [EC:3.1.3.16]

VDAC2; voltage-dependent anion channel protein 2

169. VEGF signaling pathway

PRKCA; classical protein kinase C alpha type [EC:2.7.11.13]

PPP3C; serine/threonine-protein phosphatase 2B catalytic subunit [EC:3.1.3.16]

ERK; mitogen-activated protein kinase 1/3 [EC:2.7.11.24]

RAC1; Ras-related C3 botulinum toxin substrate 1

CDC42; cell division control protein 42

P38; p38 MAP kinase [EC:2.7.11.24]

SPHK; sphingosine kinase [EC:2.7.1.91]

PPP3R; serine/threonine-protein phosphatase 2B regulatory subunit

KRAS; GTPase KRas

170. Legionellosis

EEF1A; elongation factor 1-alpha

HSPA1s; heat shock 70kDa protein 1/2/6/8

groEL; chaperonin GroEL

RAB1A; Ras-related protein Rab-1A

ARF1_2; ADP-ribosylation factor 1/2

SAR1; GTP-binding protein SAR1 [EC:3.6.5.-]

SEC22; vesicle transport protein SEC22

CYC; cytochrome c

HBS1; elongation factor 1 alpha-like protein

171. Fatty acid degradation

frmA; S-(hydroxymethyl)glutathione dehydrogenase / alcohol dehydrogenase [EC:1.1.1.284 1.1.1.1]

ALDH; aldehyde dehydrogenase (NAD+) [EC:1.2.1.3]

E1.3.3.6; acyl-CoA oxidase [EC:1.3.3.6]

ACADM; acyl-CoA dehydrogenase [EC:1.3.8.7]

ACAT; acetyl-CoA C-acetyltransferase [EC:2.3.1.9]

ACSL; long-chain acyl-CoA synthetase [EC:6.2.1.3]

ACAA1; acetyl-CoA acyltransferase 1 [EC:2.3.1.16]

ECI2; Delta3-Delta2-enoyl-CoA isomerase [EC:5.3.3.8]

adhP; alcohol dehydrogenase, propanol-preferring [EC:1.1.1.1]

172. Amyotrophic lateral sclerosis (ALS)

katE; catalase [EC:1.11.1.6]

PPP3C; serine/threonine-protein phosphatase 2B catalytic subunit [EC:3.1.3.16]

RAC1; Ras-related C3 botulinum toxin substrate 1

P38; p38 MAP kinase [EC:2.7.11.24]

SOD1; superoxide dismutase, Cu-Zn family [EC:1.15.1.1]

CCS; copper chaperone for superoxide dismutase

PPP3R; serine/threonine-protein phosphatase 2B regulatory subunit

CYC; cytochrome c

TOM40; mitochondrial import receptor subunit TOM40

173. Renal cell carcinoma

E4.2.1.2B; fumarate hydratase, class II [EC:4.2.1.2]

SOS; son of sevenless

ELOC; elongin-C

ERK; mitogen-activated protein kinase 1/3 [EC:2.7.11.24]

RAC1; Ras-related C3 botulinum toxin substrate 1

CDC42; cell division control protein 42

PAK1; p21-activated kinase 1 [EC:2.7.11.1]

KRAS; GTPase KRas

RAP1B; Ras-related protein Rap-1B

174. PD-L1 expression and PD-1 checkpoint pathway in cancer

PTEN; phosphatidylinositol-3,4,5-trisphosphate 3-phosphatase and dual-specificity protein phosphatase PTEN [EC:3.1.3.16 3.1.3.48 3.1.3.67]

CSNK2A; casein kinase II subunit alpha [EC:2.7.11.1]

CSNK2B; casein kinase II subunit beta

PPP3C; serine/threonine-protein phosphatase 2B catalytic subunit [EC:3.1.3.16]

ERK; mitogen-activated protein kinase 1/3 [EC:2.7.11.24]

P38; p38 MAP kinase [EC:2.7.11.24]

PPP3R; serine/threonine-protein phosphatase 2B regulatory subunit

MTOR; serine/threonine-protein kinase mTOR [EC:2.7.11.1]

KRAS; GTPase KRas

175. One carbon pool by folate

MTHFD; methylenetetrahydrofolate dehydrogenase (NADP+) / methenyltetrahydrofolate cyclohydrolase / formyltetrahydrofolate synthetase [EC:1.5.1.5 3.5.4.9 6.3.4.3]

MTD1; methylenetetrahydrofolate dehydrogenase (NAD+) [EC:1.5.1.15]

metF; methylenetetrahydrofolate reductase (NADPH) [EC:1.5.1.20]

thyA; thymidylate synthase [EC:2.1.1.45]

glyA; glycine hydroxymethyltransferase [EC:2.1.2.1]

E2.1.2.2; phosphoribosylglycinamide formyltransferase [EC:2.1.2.2]

purH; phosphoribosylaminoimidazolecarboxamide formyltransferase / IMP cyclohydrolase [EC:2.1.2.3 3.5.4.10]

MTFMT; methionyl-tRNA formyltransferase [EC:2.1.2.9]

gcvT; aminomethyltransferase [EC:2.1.2.10]

176. Measles

GSK3B; glycogen synthase kinase 3 beta [EC:2.7.11.26]

CSNK2A; casein kinase II subunit alpha [EC:2.7.11.1]

CSNK2B; casein kinase II subunit beta

EIF2S1; translation initiation factor 2 subunit 1

EIF3H; translation initiation factor 3 subunit H

HSPA1s; heat shock 70kDa protein 1/2/6/8

CYC; cytochrome c

RACK1; guanine nucleotide-binding protein subunit beta-2-like 1 protein

EIF2AK4; eukaryotic translation initiation factor 2-alpha kinase 4 [EC:2.7.11.1]

177. Fatty acid biosynthesis

fabG; 3-oxoacyl-[acyl-carrier protein] reductase [EC:1.1.1.100]

fabD; [acyl-carrier-protein] S-malonyltransferase [EC:2.3.1.39]

FAS2; fatty acid synthase subunit alpha, fungi type [EC:2.3.1.86]

FAS1; fatty acid synthase subunit beta, fungi type [EC:2.3.1.86]

ACSL; long-chain acyl-CoA synthetase [EC:6.2.1.3]

MECR; mitochondrial enoyl-[acyl-carrier protein] reductase / trans-2-enoyl-CoA reductase [EC:1.3.1.- 1.3.1.38]

fabF; 3-oxoacyl-[acyl-carrier-protein] synthase II [EC:2.3.1.179]

ACACA; acetyl-CoA carboxylase / biotin carboxylase 1 [EC:6.4.1.2 6.3.4.14 2.1.3.15]

HTD2; 3-hydroxyacyl-thioester dehydratase, fungi type [EC:4.2.1.-]

178. Fluid shear stress and atherosclerosis

GST; glutathione S-transferase [EC:2.5.1.18]

argG; argininosuccinate synthase [EC:6.3.4.5]

CALM; calmodulin

trxA; thioredoxin 1

HSP90A; molecular chaperone HtpG

RAC1; Ras-related C3 botulinum toxin substrate 1

P38; p38 MAP kinase [EC:2.7.11.24]

MAPK7; mitogen-activated protein kinase 7 [EC:2.7.11.24]

RHOA; Ras homolog gene family, member A

179. Folate biosynthesis

E3.1.3.1; alkaline phosphatase [EC:3.1.3.1]

GCH1; GTP cyclohydrolase IA [EC:3.5.4.16]

ribA; GTP cyclohydrolase II [EC:3.5.4.25]

PCBD; 4a-hydroxytetrahydrobiopterin dehydratase [EC:4.2.1.96]

FPGS; folylpolyglutamate synthase [EC:6.3.2.17]

pabC; 4-amino-4-deoxychorismate lyase [EC:4.1.3.38]

FOL1; dihydroneopterin aldolase / 2-amino-4-hydroxy-6-hydroxymethyldihydropteridine diphosphokinase / dihydropteroate synthase [EC:4.1.2.25 2.7.6.3 2.5.1.15]

pabAB; para-aminobenzoate synthetase [EC:2.6.1.85]

DHFS; dihydrofolate synthase [EC:6.3.2.12]

180. Butanoate metabolism

BDH; (R,R)-butanediol dehydrogenase / meso-butanediol dehydrogenase / diacetyl reductase [EC:1.1.1.4 1.1.1.- 1.1.1.303]

L2HGDH; 2-hydroxyglutarate dehydrogenase [EC:1.1.99.2]

gabD; succinate-semialdehyde dehydrogenase / glutarate-semialdehyde dehydrogenase [EC:1.2.1.16 1.2.1.79 1.2.1.20]

ACAT; acetyl-CoA C-acetyltransferase [EC:2.3.1.9]

E4.1.1.15; glutamate decarboxylase [EC:4.1.1.15]

E2.3.3.10; hydroxymethylglutaryl-CoA synthase [EC:2.3.3.10]

E2.2.1.6L; acetolactate synthase I/II/III large subunit [EC:2.2.1.6]

E2.2.1.6S; acetolactate synthase I/III small subunit [EC:2.2.1.6]

ABAT; 4-aminobutyrate aminotransferase / (S)-3-amino-2-methylpropionate transaminase [EC:2.6.1.19 2.6.1.22]

181. GABAergic synapse

E4.1.1.15; glutamate decarboxylase [EC:4.1.1.15]

PRKCA; classical protein kinase C alpha type [EC:2.7.11.13]

PKA; protein kinase A [EC:2.7.11.11]

GNB1; guanine nucleotide-binding protein G(I)/G(S)/G(T) subunit beta-1

GNAI; guanine nucleotide-binding protein G(i) subunit alpha

NSF; vesicle-fusing ATPase [EC:3.6.4.6]

GABARAP; GABA(A) receptor-associated protein

ABAT; 4-aminobutyrate aminotransferase / (S)-3-amino-2-methylpropionate transaminase [EC:2.6.1.19 2.6.1.22]

SLC32A; solute carrier family 32 (vesicular inhibitory amino acid transporter)

182. Pentose and glucuronate interconversions

AKR1A1; alcohol dehydrogenase (NADP+) [EC:1.1.1.2]

SORD; L-iditol 2-dehydrogenase [EC:1.1.1.14]

xylB; xylulokinase [EC:2.7.1.17]

UGP2; UTP--glucose-1-phosphate uridylyltransferase [EC:2.7.7.9]

rpe; ribulose-phosphate 3-epimerase [EC:5.1.3.1]

E1.1.1.9; D-xylulose reductase [EC:1.1.1.9]

ARD; D-arabinitol 2-dehydrogenase [EC:1.1.1.250]

XR; D-xylose reductase [EC:1.1.1.307]

GAAB; L-galactonate dehydratase [EC:4.2.1.146]

183. Cushing syndrome

E4.2.1.2B; fumarate hydratase, class II [EC:4.2.1.2]

GSK3B; glycogen synthase kinase 3 beta [EC:2.7.11.26]

PKA; protein kinase A [EC:2.7.11.11]

ERK; mitogen-activated protein kinase 1/3 [EC:2.7.11.24]

GNAI; guanine nucleotide-binding protein G(i) subunit alpha

RAP1B; Ras-related protein Rap-1B

USP8; ubiquitin carboxyl-terminal hydrolase 8 [EC:3.4.19.12]

RBBP5; COMPASS component SWD1

WDR5; COMPASS component SWD3

184. Rheumatoid arthritis

ATPeV1A; V-type H+-transporting ATPase subunit A [EC:7.1.2.2]

ATPeV0D; V-type H+-transporting ATPase subunit d

ATPeV1B; V-type H+-transporting ATPase subunit B

ATPeV1C; V-type H+-transporting ATPase subunit C

ATPeV1D; V-type H+-transporting ATPase subunit D

ATPeV1G; V-type H+-transporting ATPase subunit G
ATPeV0A; V-type H+-transporting ATPase subunit a
ATPeV0C; V-type H+-transporting ATPase 16kDa proteolipid subunit
ATPeV0B; V-type H+-transporting ATPase 21kDa proteolipid subunit

185. Collecting duct acid secretion

ATPeV1A; V-type H+-transporting ATPase subunit A [EC:7.1.2.2]
ATPeV0D; V-type H+-transporting ATPase subunit d
ATPeV1B; V-type H+-transporting ATPase subunit B
ATPeV1C; V-type H+-transporting ATPase subunit C
ATPeV1D; V-type H+-transporting ATPase subunit D
ATPeV1G; V-type H+-transporting ATPase subunit G
ATPeV0A; V-type H+-transporting ATPase subunit a
ATPeV0C; V-type H+-transporting ATPase 16kDa proteolipid subunit

186. lutamatergic synapse

PLD1_2; phospholipase D1/2 [EC:3.1.4.4]
PRKCA; classical protein kinase C alpha type [EC:2.7.11.13]
PKA; protein kinase A [EC:2.7.11.11]
PPP3C; serine/threonine-protein phosphatase 2B catalytic subunit [EC:3.1.3.16]
ERK; mitogen-activated protein kinase 1/3 [EC:2.7.11.24]
GNB1; guanine nucleotide-binding protein G(I)/G(S)/G(T) subunit beta-1
GNAI; guanine nucleotide-binding protein G(i) subunit alpha
PPP3R; serine/threonine-protein phosphatase 2B regulatory subunit

187. Gastric cancer

GSK3B; glycogen synthase kinase 3 beta [EC:2.7.11.26]
SOS; son of sevenless
ERK; mitogen-activated protein kinase 1/3 [EC:2.7.11.24]
ABCB1; ATP-binding cassette, subfamily B (MDR/TAP), member 1 [EC:7.6.2.2]
MTOR; serine/threonine-protein kinase mTOR [EC:2.7.11.1]
KRAS; GTPase KRas

MLH1; DNA mismatch repair protein MLH1

TERT; telomerase reverse transcriptase [EC:2.7.7.49]

188. Transcriptional misregulation in cancer

ATM; serine-protein kinase ATM [EC:2.7.11.1]

HDAC1_2; histone deacetylase 1/2 [EC:3.5.1.98]

H3; histone H3

SUPT3H; transcription initiation protein SPT3

DOT1L; [histone H3]-lysine79 N-trimethyltransferase [EC:2.1.1.360]

SIN3A; paired amphipathic helix protein Sin3a

DDX5; ATP-dependent RNA helicase DDX5/DBP2 [EC:3.6.4.13]

TAF15; transcription initiation factor TFIID subunit 15

189. Apoptosis

ENDOG; endonuclease G, mitochondrial

EIF2S1; translation initiation factor 2 subunit 1

ERK; mitogen-activated protein kinase 1/3 [EC:2.7.11.24]

ATM; serine-protein kinase ATM [EC:2.7.11.1]

PDPK1; 3-phosphoinositide dependent protein kinase-1 [EC:2.7.11.1]

KRAS; GTPase KRas

CYC; cytochrome c

ERN1; serine/threonine-protein kinase/endoribonuclease IRE1 [EC:2.7.11.1 3.1.26.-]

190. Adherens junction

CSNK2A; casein kinase II subunit alpha [EC:2.7.11.1]

CSNK2B; casein kinase II subunit beta

ERK; mitogen-activated protein kinase 1/3 [EC:2.7.11.24]

RAC1; Ras-related C3 botulinum toxin substrate 1

CDC42; cell division control protein 42

RHOA; Ras homolog gene family, member A

ACP1; low molecular weight phosphotyrosine protein phosphatase [EC:3.1.3.2 3.1.3.48]

WASL; neural Wistt-Aldrich syndrome protein

191. Natural killer cell mediated cytotoxicity

PRKCA; classical protein kinase C alpha type [EC:2.7.11.13]

SOS; son of sevenless

PPP3C; serine/threonine-protein phosphatase 2B catalytic subunit [EC:3.1.3.16]

ERK; mitogen-activated protein kinase 1/3 [EC:2.7.11.24]

RAC1; Ras-related C3 botulinum toxin substrate 1

PAK1; p21-activated kinase 1 [EC:2.7.11.1]

PPP3R; serine/threonine-protein phosphatase 2B regulatory subunit

KRAS; GTPase KRas

192. EGFR tyrosine kinase inhibitor resistance

PTEN; phosphatidylinositol-3,4,5-trisphosphate 3-phosphatase and dual-specificity protein phosphatase PTEN [EC:3.1.3.16 3.1.3.48 3.1.3.67]

PRKCA; classical protein kinase C alpha type [EC:2.7.11.13]

GSK3B; glycogen synthase kinase 3 beta [EC:2.7.11.26]

SOS; son of sevenless

EIF4E; translation initiation factor 4E

ERK; mitogen-activated protein kinase 1/3 [EC:2.7.11.24]

MTOR; serine/threonine-protein kinase mTOR [EC:2.7.11.1]

KRAS; GTPase KRas

193. Estrogen signaling pathway

CALM; calmodulin

SOS; son of sevenless

HSPA1s; heat shock 70kDa protein 1/2/6/8

HSP90A; molecular chaperone HtpG

PKA; protein kinase A [EC:2.7.11.11]

ERK; mitogen-activated protein kinase 1/3 [EC:2.7.11.24]

GNAI; guanine nucleotide-binding protein G(i) subunit alpha

KRAS; GTPase KRas

194. Relaxin signaling pathway

PRKCA; classical protein kinase C alpha type [EC:2.7.11.13]

SOS; son of sevenless

PKA; protein kinase A [EC:2.7.11.11]

ERK; mitogen-activated protein kinase 1/3 [EC:2.7.11.24]

P38; p38 MAP kinase [EC:2.7.11.24]

GNB1; guanine nucleotide-binding protein G(I)/G(S)/G(T) subunit beta-1

GNAI; guanine nucleotide-binding protein G(i) subunit alpha

KRAS; GTPase KRas

195. Glioma

PTEN; phosphatidylinositol-3,4,5-trisphosphate 3-phosphatase and dual-specificity protein phosphatase PTEN [EC:3.1.3.16 3.1.3.48 3.1.3.67]

CALM; calmodulin

PRKCA; classical protein kinase C alpha type [EC:2.7.11.13]

SOS; son of sevenless

ERK; mitogen-activated protein kinase 1/3 [EC:2.7.11.24]

MTOR; serine/threonine-protein kinase mTOR [EC:2.7.11.1]

KRAS; GTPase KRas

CAMK1; calcium/calmodulin-dependent protein kinase I [EC:2.7.11.17]

196. C-type lectin receptor signaling pathway

CALM; calmodulin

PPP3C; serine/threonine-protein phosphatase 2B catalytic subunit [EC:3.1.3.16]

ERK; mitogen-activated protein kinase 1/3 [EC:2.7.11.24]

PAK1; p21-activated kinase 1 [EC:2.7.11.1]

P38; p38 MAP kinase [EC:2.7.11.24]

RHOA; Ras homolog gene family, member A

PPP3R; serine/threonine-protein phosphatase 2B regulatory subunit

KRAS; GTPase KRas

197. Prostate cancer

PTEN; phosphatidylinositol-3,4,5-trisphosphate 3-phosphatase and dual-specificity protein phosphatase PTEN [EC:3.1.3.16 3.1.3.48 3.1.3.67]

GSK3B; glycogen synthase kinase 3 beta [EC:2.7.11.26]

SOS; son of sevenless

HSP90A; molecular chaperone HtpG

ERK; mitogen-activated protein kinase 1/3 [EC:2.7.11.24]

PDPK1; 3-phosphoinositide dependent protein kinase-1 [EC:2.7.11.1]

MTOR; serine/threonine-protein kinase mTOR [EC:2.7.11.1]

KRAS; GTPase KRas

198. Vasopressin-regulated water reabsorption

PKA; protein kinase A [EC:2.7.11.11]

DCTN1; dynactin 1

NSF; vesicle-fusing ATPase [EC:3.6.4.6]

RAB5C; Ras-related protein Rab-5C

RAB11B; Ras-related protein Rab-11B

DYNC1H; dynein heavy chain 1, cytosolic

DYNC1I; dynein intermediate chain, cytosolic

ARHGDI; Rho GDP-dissociation inhibitor

199. PPAR signaling pathway

E1.3.3.6; acyl-CoA oxidase [EC:1.3.3.6]

ACADM; acyl-CoA dehydrogenase [EC:1.3.8.7]

SCD; stearoyl-CoA desaturase (Delta-9 desaturase) [EC:1.14.19.1]

E2.3.3.10; hydroxymethylglutaryl-CoA synthase [EC:2.3.3.10]

ACSL; long-chain acyl-CoA synthetase [EC:6.2.1.3]

PDPK1; 3-phosphoinositide dependent protein kinase-1 [EC:2.7.11.1]

ACAA1; acetyl-CoA acyltransferase 1 [EC:2.3.1.16]

UBC; ubiquitin C

200. Hepatitis B

PRKCA; classical protein kinase C alpha type [EC:2.7.11.13]

SOS; son of sevenless

ERK; mitogen-activated protein kinase 1/3 [EC:2.7.11.24]

P38; p38 MAP kinase [EC:2.7.11.24]

PCNA; proliferating cell nuclear antigen

KRAS; GTPase KRas

CYC; cytochrome c

DDX3X; ATP-dependent RNA helicase DDX3X [EC:3.6.4.13]

201. Systemic lupus erythematosus

SNRPB; small nuclear ribonucleoprotein B and B'

SNRPD1; small nuclear ribonucleoprotein D1

SNRPD3; small nuclear ribonucleoprotein D3

LA; lupus La protein

H2A; histone H2A

H2B; histone H2B

H3; histone H3

H4; histone H4

202. Endocrine and other factor-regulated calcium reabsorption

PRKCA; classical protein kinase C alpha type [EC:2.7.11.13]

PKA; protein kinase A [EC:2.7.11.11]

CLTC; clathrin heavy chain

AP2A; AP-2 complex subunit alpha

AP2B1; AP-2 complex subunit beta-1

AP2M1; AP-2 complex subunit mu-1

AP2S1; AP-2 complex subunit sigma-1

203. Gap junction

PRKCA; classical protein kinase C alpha type [EC:2.7.11.13]

SOS; son of sevenless

PKA; protein kinase A [EC:2.7.11.11]

ERK; mitogen-activated protein kinase 1/3 [EC:2.7.11.24]

MAPK7; mitogen-activated protein kinase 7 [EC:2.7.11.24]

GNAI; guanine nucleotide-binding protein G(i) subunit alpha

KRAS; GTPase KRas

204. Quorum sensing

ribA; GTP cyclohydrolase II [EC:3.5.4.25]

E4.1.1.15; glutamate decarboxylase [EC:4.1.1.15]

E2.5.1.54; 3-deoxy-7-phosphoheptulonate synthase [EC:2.5.1.54]

trpE; anthranilate synthase component I [EC:4.1.3.27]

ACSL; long-chain acyl-CoA synthetase [EC:6.2.1.3]

SRP54; signal recognition particle subunit SRP54 [EC:3.6.5.4]

yidC; YidC/Oxa1 family membrane protein insertase

205. ErbB signaling pathway

PRKCA; classical protein kinase C alpha type [EC:2.7.11.13]

GSK3B; glycogen synthase kinase 3 beta [EC:2.7.11.26]

SOS; son of sevenless

ERK; mitogen-activated protein kinase 1/3 [EC:2.7.11.24]

PAK1; p21-activated kinase 1 [EC:2.7.11.1]

MTOR; serine/threonine-protein kinase mTOR [EC:2.7.11.1]

KRAS; GTPase KRas

206. Non-homologous end-joining

FEN1; flap endonuclease-1 [EC:3.-.-.-]

LIG4; DNA ligase 4 [EC:6.5.1.1]

MRE11; double-strand break repair protein MRE11

RAD50; DNA repair protein RAD50 [EC:3.6.-.-]

XRCC6; ATP-dependent DNA helicase 2 subunit 1

XRCC5; ATP-dependent DNA helicase 2 subunit 2

DCLRE1C; DNA cross-link repair 1C protein [EC:3.1.-.-]

207. Parathyroid hormone synthesis, secretion and action

PLD1_2; phospholipase D1/2 [EC:3.1.4.4]

PRKCA; classical protein kinase C alpha type [EC:2.7.11.13]

EGD2; nascent polypeptide-associated complex subunit alpha

PKA; protein kinase A [EC:2.7.11.11]

ERK; mitogen-activated protein kinase 1/3 [EC:2.7.11.24]

RHOA; Ras homolog gene family, member A

GNAI; guanine nucleotide-binding protein G(i) subunit alpha

208. Carbon fixation pathways in prokaryotes

IDH1; isocitrate dehydrogenase [EC:1.1.1.42]

metF; methylenetetrahydrofolate reductase (NADPH) [EC:1.5.1.20]

ACAT; acetyl-CoA C-acetyltransferase [EC:2.3.1.9]

E4.2.1.2B; fumarate hydratase, class II [EC:4.2.1.2]

ACO; aconitate hydratase [EC:4.2.1.3]

ACSS1_2; acetyl-CoA synthetase [EC:6.2.1.1]

PC; pyruvate carboxylase [EC:6.4.1.1]

209. Pancreatic cancer

PLD1_2; phospholipase D1/2 [EC:3.1.4.4]

ERK; mitogen-activated protein kinase 1/3 [EC:2.7.11.24]

RAC1; Ras-related C3 botulinum toxin substrate 1

CDC42; cell division control protein 42

RAD51; DNA repair protein RAD51

MTOR; serine/threonine-protein kinase mTOR [EC:2.7.11.1]

KRAS; GTPase KRas

210. Amphetamine addiction

CALM; calmodulin

PRKCA; classical protein kinase C alpha type [EC:2.7.11.13]

PKA; protein kinase A [EC:2.7.11.11]

PPP3C; serine/threonine-protein phosphatase 2B catalytic subunit [EC:3.1.3.16]

HDAC1_2; histone deacetylase 1/2 [EC:3.5.1.98]

PPP3R; serine/threonine-protein phosphatase 2B regulatory subunit

PPP1C; serine/threonine-protein phosphatase PP1 catalytic subunit [EC:3.1.3.16]

211. AGE-RAGE signaling pathway in diabetic complications

PRKCA; classical protein kinase C alpha type [EC:2.7.11.13]

ERK; mitogen-activated protein kinase 1/3 [EC:2.7.11.24]

RAC1; Ras-related C3 botulinum toxin substrate 1

CDC42; cell division control protein 42

P38; p38 MAP kinase [EC:2.7.11.24]

PLCD; phosphatidylinositol phospholipase C, delta [EC:3.1.4.11]

KRAS; GTPase KRas

212. Endometrial cancer

PTEN; phosphatidylinositol-3,4,5-trisphosphate 3-phosphatase and dual-specificity protein phosphatase PTEN [EC:3.1.3.16 3.1.3.48 3.1.3.67]

GSK3B; glycogen synthase kinase 3 beta [EC:2.7.11.26]

SOS; son of sevenless

ERK; mitogen-activated protein kinase 1/3 [EC:2.7.11.24]

PDPK1; 3-phosphoinositide dependent protein kinase-1 [EC:2.7.11.1]

KRAS; GTPase KRas

MLH1; DNA mismatch repair protein MLH1

213. Axon regeneration

PTEN; phosphatidylinositol-3,4,5-trisphosphate 3-phosphatase and dual-specificity protein phosphatase PTEN [EC:3.1.3.16 3.1.3.48 3.1.3.67]

PKA; protein kinase A [EC:2.7.11.11]

RAC1; Ras-related C3 botulinum toxin substrate 1

P38; p38 MAP kinase [EC:2.7.11.24]

DUSP; dual specificity MAP kinase phosphatase [EC:3.1.3.16 3.1.3.48]

RHOA; Ras homolog gene family, member A

MTOR; serine/threonine-protein kinase mTOR [EC:2.7.11.1]

214. Ubiquinone and other terpenoid-quinone biosynthesis

COQ3; polyprenyldihydroxybenzoate methyltransferase / 3-demethylubiquinol 3-O-methyltransferase [EC:2.1.1.114 2.1.1.64]

ARO8; aromatic amino acid aminotransferase I / 2-aminoadipate transaminase [EC:2.6.1.57 2.6.1.39 2.6.1.27 2.6.1.5]

wrbA; NAD(P)H dehydrogenase (quinone) [EC:1.6.5.2]

COQ2; 4-hydroxybenzoate polyprenyltransferase [EC:2.5.1.39]

COQ6; ubiquinone biosynthesis monooxygenase Coq6 [EC:1.14.13.-]

COQ5; 2-methoxy-6-polyprenyl-1,4-benzoquinol methylase [EC:2.1.1.201]

COQ7; 3-demethoxyubiquinol 3-hydroxylase [EC:1.14.99.60]

215. Vitamin B6 metabolism

pdxH; pyridoxamine 5'-phosphate oxidase [EC:1.4.3.5]

serC; phosphoserine aminotransferase [EC:2.6.1.52]

pdxK; pyridoxine kinase [EC:2.7.1.35]

thrC; threonine synthase [EC:4.2.3.1]

E1.1.1.65; pyridoxine 4-dehydrogenase [EC:1.1.1.65]

pdxS; pyridoxal 5'-phosphate synthase pdxS subunit [EC:4.3.3.6]

pdxT; 5'-phosphate synthase pdxT subunit [EC:4.3.3.6]

216. Melanogenesis

CALM; calmodulin

PRKCA; classical protein kinase C alpha type [EC:2.7.11.13]

GSK3B; glycogen synthase kinase 3 beta [EC:2.7.11.26]

PKA; protein kinase A [EC:2.7.11.11]

ERK; mitogen-activated protein kinase 1/3 [EC:2.7.11.24]

GNAI; guanine nucleotide-binding protein G(i) subunit alpha

KRAS; GTPase KRas

217. Platelet activation

PKA; protein kinase A [EC:2.7.11.11]

ERK; mitogen-activated protein kinase 1/3 [EC:2.7.11.24]

P38; p38 MAP kinase [EC:2.7.11.24]

RHOA; Ras homolog gene family, member A

GNAI; guanine nucleotide-binding protein G(i) subunit alpha

PPP1C; serine/threonine-protein phosphatase PP1 catalytic subunit [EC:3.1.3.16]

RAP1B; Ras-related protein Rap-1B

218. B cell receptor signaling pathway

GSK3B; glycogen synthase kinase 3 beta [EC:2.7.11.26]

SOS; son of sevenless

PPP3C; serine/threonine-protein phosphatase 2B catalytic subunit [EC:3.1.3.16]

ERK; mitogen-activated protein kinase 1/3 [EC:2.7.11.24]

RAC1; Ras-related C3 botulinum toxin substrate 1

PPP3R; serine/threonine-protein phosphatase 2B regulatory subunit

KRAS; GTPase KRas

219. Fatty acid elongation

PPT; palmitoyl-protein thioesterase [EC:3.1.2.22]

MECR; mitochondrial enoyl-[acyl-carrier protein] reductase / trans-2-enoyl-CoA reductase [EC:1.3.1.- 1.3.1.38]

ELO2; fatty acid elongase 2 [EC:2.3.1.199]

ELO3; fatty acid elongase 3 [EC:2.3.1.199]

HSD17B12; 17beta-estradiol 17-dehydrogenase / very-long-chain 3-oxoacyl-CoA reductase [EC:1.1.1.62 1.1.1.330]

TER; very-long-chain enoyl-CoA reductase [EC:1.3.1.93]

HACD; very-long-chain (3R)-3-hydroxyacyl-CoA dehydratase [EC:4.2.1.134]

220. Hippo signaling pathway - multiple species

CSNK1; casein kinase 1 [EC:2.7.11.1]

PAK1; p21-activated kinase 1 [EC:2.7.11.1]

CDC15; cell division control protein CDC15 [EC:2.7.11.1]

DBF2; cell cycle protein kinase DBF2 [EC:2.7.11.-]

MOB1; MOB kinase activator 1

TEAD; transcriptional enhancer factor

NUD1; protein NUD1

221. Hippo signaling pathway

GSK3B; glycogen synthase kinase 3 beta [EC:2.7.11.26]

PPP2R1; serine/threonine-protein phosphatase 2A regulatory subunit A

PPP2R2; serine/threonine-protein phosphatase 2A regulatory subunit B

PPP1C; serine/threonine-protein phosphatase PP1 catalytic subunit [EC:3.1.3.16]

YWHAE; 14-3-3 protein epsilon

MOB1; MOB kinase activator 1

TEAD; transcriptional enhancer factor

222. IL-17 signaling pathway

GSK3B; glycogen synthase kinase 3 beta [EC:2.7.11.26]

APC5; anaphase-promoting complex subunit 5

HSP90A; molecular chaperone HtpG

ERK; mitogen-activated protein kinase 1/3 [EC:2.7.11.24]

P38; p38 MAP kinase [EC:2.7.11.24]

MAPK7; mitogen-activated protein kinase 7 [EC:2.7.11.24]

USP25; ubiquitin carboxyl-terminal hydrolase 25 [EC:3.4.19.12]

223. Leucyte transendothelial migration

PRKCA; classical protein kinase C alpha type [EC:2.7.11.13]

RAC1; Ras-related C3 botulinum toxin substrate 1

CDC42; cell division control protein 42

P38; p38 MAP kinase [EC:2.7.11.24]

RHOA; Ras homolog gene family, member A

GNAI; guanine nucleotide-binding protein G(i) subunit alpha

RAP1B; Ras-related protein Rap-1B

224. Tropane, piperidine and pyridine alkaloid biosynthesis

AOC3; primary-amine oxidase [EC:1.4.3.21]

hisC; histidinol-phosphate aminotransferase [EC:2.6.1.9]

ARO8; aromatic amino acid aminotransferase I / 2-aminoadipate transaminase [EC:2.6.1.57 2.6.1.39 2.6.1.27 2.6.1.5]

ARO9; aromatic amino acid aminotransferase II [EC:2.6.1.58 2.6.1.28]

GOT1; aspartate aminotransferase, cytoplasmic [EC:2.6.1.1]

GOT2; aspartate aminotransferase, mitochondrial [EC:2.6.1.1]

225. Serotonergic synapse

PRKCA; classical protein kinase C alpha type [EC:2.7.11.13]

PKA; protein kinase A [EC:2.7.11.11]

ERK; mitogen-activated protein kinase 1/3 [EC:2.7.11.24]

GNB1; guanine nucleotide-binding protein G(I)/G(S)/G(T) subunit beta-1

GNAI; guanine nucleotide-binding protein G(i) subunit alpha

KRAS; GTPase KRas

226. Breast cancer

PTEN; phosphatidylinositol-3,4,5-trisphosphate 3-phosphatase and dual-specificity protein phosphatase PTEN [EC:3.1.3.16 3.1.3.48 3.1.3.67]

GSK3B; glycogen synthase kinase 3 beta [EC:2.7.11.26]

SOS; son of sevenless

ERK; mitogen-activated protein kinase 1/3 [EC:2.7.11.24]

MTOR; serine/threonine-protein kinase mTOR [EC:2.7.11.1]

KRAS; GTPase KRas

227. Vascular smooth muscle contraction

CALM; calmodulin

PRKCA; classical protein kinase C alpha type [EC:2.7.11.13]

PKA; protein kinase A [EC:2.7.11.11]

ERK; mitogen-activated protein kinase 1/3 [EC:2.7.11.24]

RHOA; Ras homolog gene family, member A

PPP1C; serine/threonine-protein phosphatase PP1 catalytic subunit [EC:3.1.3.16]

228. Toxoplasmosis

HSPA1s; heat shock 70kDa protein 1/2/6/8

ERK; mitogen-activated protein kinase 1/3 [EC:2.7.11.24]

P38; p38 MAP kinase [EC:2.7.11.24]

GNAI; guanine nucleotide-binding protein G(i) subunit alpha

PDPK1; 3-phosphoinositide dependent protein kinase-1 [EC:2.7.11.1]

CYC; cytochrome c

229. Cyanoamino acid metabolism

glyA; glycine hydroxymethyltransferase [EC:2.1.2.1]

ggt; gamma-glutamyltranspeptidase / glutathione hydrolase [EC:2.3.2.2 3.4.19.13]

E3.5.1.1; L-asparaginase [EC:3.5.1.1]

E3.5.1.49; formamidase [EC:3.5.1.49]

E3.5.5.1; nitrilase [EC:3.5.5.1]

bglX; beta-glucosidase [EC:3.2.1.21]

230. Non-small cell lung cancer

FHIT; bis(5'-adenosyl)-triphosphatase [EC:3.6.1.29]

PRKCA; classical protein kinase C alpha type [EC:2.7.11.13]

SOS; son of sevenless

ERK; mitogen-activated protein kinase 1/3 [EC:2.7.11.24]

PDPK1; 3-phosphoinositide dependent protein kinase-1 [EC:2.7.11.1]

KRAS; GTPase KRas

231. Prolactin signaling pathway

galT; UDPglucose--hexose-1-phosphate uridylyltransferase [EC:2.7.7.12]

GSK3B; glycogen synthase kinase 3 beta [EC:2.7.11.26]

SOS; son of sevenless

ERK; mitogen-activated protein kinase 1/3 [EC:2.7.11.24]

P38; p38 MAP kinase [EC:2.7.11.24]

KRAS; GTPase KRas

232. Circadian entrainment

CALM; calmodulin

PRKCA; classical protein kinase C alpha type [EC:2.7.11.13]

PKA; protein kinase A [EC:2.7.11.11]

ERK; mitogen-activated protein kinase 1/3 [EC:2.7.11.24]

GNB1; guanine nucleotide-binding protein G(I)/G(S)/G(T) subunit beta-1

GNAI; guanine nucleotide-binding protein G(i) subunit alpha

233. Th17 cell differentiation

HSP90A; molecular chaperone HtpG

PPP3C; serine/threonine-protein phosphatase 2B catalytic subunit [EC:3.1.3.16]

ERK; mitogen-activated protein kinase 1/3 [EC:2.7.11.24]

P38; p38 MAP kinase [EC:2.7.11.24]

PPP3R; serine/threonine-protein phosphatase 2B regulatory subunit

MTOR; serine/threonine-protein kinase mTOR [EC:2.7.11.1]

234. Nitrogen metabolism

E1.4.1.4; glutamate dehydrogenase (NADP+) [EC:1.4.1.4]

ncd2; nitronate monooxygenase [EC:1.13.12.16]

E3.5.1.49; formamidase [EC:3.5.1.49]

E3.5.5.1; nitrilase [EC:3.5.5.1]

cynT; carbonic anhydrase [EC:4.2.1.1]

GDH2; glutamate dehydrogenase [EC:1.4.1.2]

235. Hippo signaling pathway - fly

PPP2R1; serine/threonine-protein phosphatase 2A regulatory subunit A

PPP2R2; serine/threonine-protein phosphatase 2A regulatory subunit B

YWHAE; 14-3-3 protein epsilon

MOB1; MOB kinase activator 1

TEAD; transcriptional enhancer factor

ZDHHC9_14_18; palmitoyltransferase ZDHHC9/14/18 [EC:2.3.1.225]

236. Antifolate resistance

metF; methylenetetrahydrofolate reductase (NADPH) [EC:1.5.1.20]

thyA; thymidylate synthase [EC:2.1.1.45]

glyA; glycine hydroxymethyltransferase [EC:2.1.2.1]

purH; phosphoribosylaminoimidazolecarboxamide formyltransferase / IMP cyclohydrolase [EC:2.1.2.3 3.5.4.10]

FPGS; folylpolyglutamate synthase [EC:6.3.2.17]

ABCC1; ATP-binding cassette, subfamily C (CFTR/MRP), member 1 [EC:7.6.2.3]

237. Ferroptosis

ACSL; long-chain acyl-CoA synthetase [EC:6.2.1.3]

ATG7; ubiquitin-like modifier-activating enzyme ATG7

ATG5; autophagy-related protein 5

GCLC; glutamate--cysteine ligase catalytic subunit [EC:6.3.2.2]

VDAC2; voltage-dependent anion channel protein 2

GSS; glutathione synthase [EC:6.3.2.3]

238. Ether lipid metabolism

EPT1; ethanolaminephosphotransferase [EC:2.7.8.1]

PLD1_2; phospholipase D1/2 [EC:3.1.4.4]

AYR1; 1-acylglycerone phosphate reductase [EC:1.1.1.101]

LPT1; lysophospholipid acyltransferase [EC:2.3.1.51 2.3.1.23 2.3.1.-]

TGL4; TAG lipase / steryl ester hydrolase / phospholipase A2 / LPA acyltransferase [EC:3.1.1.3 3.1.1.13 3.1.1.4 2.3.1.51]

PAFAH1B1; platelet-activating factor acetylhydrolase IB subunit alpha

239. Cholinergic synapse

PRKCA; classical protein kinase C alpha type [EC:2.7.11.13]

PKA; protein kinase A [EC:2.7.11.11]

ERK; mitogen-activated protein kinase 1/3 [EC:2.7.11.24]

GNB1; guanine nucleotide-binding protein G(I)/G(S)/G(T) subunit beta-1

GNAI; guanine nucleotide-binding protein G(i) subunit alpha
KRAS; GTPase KRas

240. Endocrine resistance
SOS; son of sevenless
PKA; protein kinase A [EC:2.7.11.11]
ERK; mitogen-activated protein kinase 1/3 [EC:2.7.11.24]
P38; p38 MAP kinase [EC:2.7.11.24]
MTOR; serine/threonine-protein kinase mTOR [EC:2.7.11.1]
KRAS; GTPase KRas

241. RIG-I-like receptor signaling pathway
DAK; triose/dihydroxyacetone kinase / FAD-AMP lyase (cyclizing) [EC:2.7.1.28 2.7.1.29 4.6.1.15]
P38; p38 MAP kinase [EC:2.7.11.24]
ATG5; autophagy-related protein 5
PIN1; peptidyl-prolyl cis-trans isomerase NIMA-interacting 1 [EC:5.2.1.8]
DDX3X; ATP-dependent RNA helicase DDX3X [EC:3.6.4.13]

242. MAPK signaling pathway - plant
ndk; nucleoside-diphosphate kinase [EC:2.7.4.6]
CALM; calmodulin
katE; catalase [EC:1.11.1.6]
PR1; pathogenesis-related protein 1
copA; P-type Cu+ transporter [EC:7.2.2.8]

243. Long-term depression
PRKCA; classical protein kinase C alpha type [EC:2.7.11.13]
PPP2R1; serine/threonine-protein phosphatase 2A regulatory subunit A
ERK; mitogen-activated protein kinase 1/3 [EC:2.7.11.24]
GNAI; guanine nucleotide-binding protein G(i) subunit alpha
KRAS; GTPase KRas

244. Pancreatic secretion

PRKCA; classical protein kinase C alpha type [EC:2.7.11.13]

RAC1; Ras-related C3 botulinum toxin substrate 1

RHOA; Ras homolog gene family, member A

RAP1B; Ras-related protein Rap-1B

RAB8A; Ras-related protein Rab-8A

245. Morphine addiction

PRKCA; classical protein kinase C alpha type [EC:2.7.11.13]

PKA; protein kinase A [EC:2.7.11.11]

GNB1; guanine nucleotide-binding protein G(I)/G(S)/G(T) subunit beta-1

GNAI; guanine nucleotide-binding protein G(i) subunit alpha

SLC32A; solute carrier family 32 (vesicular inhibitory amino acid transporter)

246. Antigen processing and presentation

HSPA1s; heat shock 70kDa protein 1/2/6/8

HSP90A; molecular chaperone HtpG

CANX; calnexin

NFYB; nuclear transcription Y subunit beta

NFYC; nuclear transcription factor Y, gamma

247. Signaling pathways regulating pluripotency of stem cells

GSK3B; glycogen synthase kinase 3 beta [EC:2.7.11.26]

ERK; mitogen-activated protein kinase 1/3 [EC:2.7.11.24]

P38; p38 MAP kinase [EC:2.7.11.24]

KRAS; GTPase KRas

SMARCAD1; SWI/SNF-related matrix-associated actin-dependent regulator of chromatin subfamily A containing DEAD/H box 1 [EC:3.6.4.12]

248. Sulfur relay system

iscS; cysteine desulfurase [EC:2.8.1.7]

MOCS3; adenylyltransferase and sulfurtransferase [EC:2.7.7.80 2.8.1.11]

CTU1; cytoplasmic tRNA 2-thiolation protein 1 [EC:2.7.7.-]

CTU2; cytoplasmic tRNA 2-thiolation protein 2

AHP1; alkyl hydroperoxide reductase 1 [EC:1.11.1.24]

249. Amoebiasis

E3.5.3.1; arginase [EC:3.5.3.1]

PRKCA; classical protein kinase C alpha type [EC:2.7.11.13]

PKA; protein kinase A [EC:2.7.11.11]

RAB5C; Ras-related protein Rab-5C

RAB7A; Ras-related protein Rab-7A

250. Renin secretion

CALM; calmodulin

PKA; protein kinase A [EC:2.7.11.11]

PPP3C; serine/threonine-protein phosphatase 2B catalytic subunit [EC:3.1.3.16]

GNAI; guanine nucleotide-binding protein G(i) subunit alpha

PPP3R; serine/threonine-protein phosphatase 2B regulatory subunit

251. p53 signaling pathway

PTEN; phosphatidylinositol-3,4,5-trisphosphate 3-phosphatase and dual-specificity protein phosphatase PTEN [EC:3.1.3.16 3.1.3.48 3.1.3.67]

ATM; serine-protein kinase ATM [EC:2.7.11.1]

CYC; cytochrome c

RRM2; ribonucleoside-diphosphate reductase subunit M2 [EC:1.17.4.1]

AIFM2; apoptosis-inducing factor 2

252. TGF-beta signaling pathway

SKP1; S-phase kinase-associated protein 1

CUL1; cullin 1

PPP2R1; serine/threonine-protein phosphatase 2A regulatory subunit A

ERK; mitogen-activated protein kinase 1/3 [EC:2.7.11.24]

RHOA; Ras homolog gene family, member A

253. Plant-pathogen interaction

CALM; calmodulin

tuf; elongation factor Tu

HSP90A; molecular chaperone HtpG

SUGT1; suppressor of G2 allele of SKP1

PR1; pathogenesis-related protein 1

254. ABC transporters

ABCB1; ATP-binding cassette, subfamily B (MDR/TAP), member 1 [EC:7.6.2.2]

ATM; ATP-binding cassette, subfamily B, mitochondrial transporter ATM

ABCC1; ATP-binding cassette, subfamily C (CFTR/MRP), member 1 [EC:7.6.2.3]

ABCG2.PDR; ATP-binding cassette, subfamily G (WHITE), member 2, PDR

PXA; ATP-binding cassette, subfamily D (ALD), peroxisomal long-chain fatty acid import protein

255. Inflammatory mediator regulation of TRP channels

CALM; calmodulin

PRKCA; classical protein kinase C alpha type [EC:2.7.11.13]

PKA; protein kinase A [EC:2.7.11.11]

P38; p38 MAP kinase [EC:2.7.11.24]

PPP1C; serine/threonine-protein phosphatase PP1 catalytic subunit [EC:3.1.3.16]

256. Pertussis

CALM; calmodulin

ERK; mitogen-activated protein kinase 1/3 [EC:2.7.11.24]

P38; p38 MAP kinase [EC:2.7.11.24]

RHOA; Ras homolog gene family, member A

GNAI; guanine nucleotide-binding protein G(i) subunit alpha

257. Metabolism of xenobiotics by cytochrome P450

frmA; S-(hydroxymethyl)glutathione dehydrogenase / alcohol dehydrogenase [EC:1.1.1.284 1.1.1.1]

E1.2.1.5; aldehyde dehydrogenase (NAD(P)+) [EC:1.2.1.5]

GST; glutathione S-transferase [EC:2.5.1.18]

adhP; alcohol dehydrogenase, propanol-preferring [EC:1.1.1.1]

258. Toll and Imd signaling pathway

P38; p38 MAP kinase [EC:2.7.11.24]

UBE2D; ubiquitin-conjugating enzyme E2 D [EC:2.3.2.23]

UBE2N; ubiquitin-conjugating enzyme E2 N [EC:2.3.2.23]

UBE2V; ubiquitin-conjugating enzyme E2 variant

259. Other glycan degradation

lacZ; beta-galactosidase [EC:3.2.1.23]

MAN2C1; alpha-mannosidase [EC:3.2.1.24]

E3.2.1.25; beta-mannosidase [EC:3.2.1.25]

HEXA_B; hexosaminidase [EC:3.2.1.52]

260. Hedgehog signaling pathway

GSK3B; glycogen synthase kinase 3 beta [EC:2.7.11.26]

CUL1; cullin 1

CUL3; cullin 3

PKA; protein kinase A [EC:2.7.11.11]

261. Taurine and hypotaurine metabolism

CDO1; cysteine dioxygenase [EC:1.13.11.20]

ggt; gamma-glutamyltranspeptidase / glutathione hydrolase [EC:2.3.2.2 3.4.19.13]

E4.1.1.15; glutamate decarboxylase [EC:4.1.1.15]

GDH2; glutamate dehydrogenase [EC:1.4.1.2]

262. Monobactam biosynthesis

asd; aspartate-semialdehyde dehydrogenase [EC:1.2.1.11]

lysC; aspartate kinase [EC:2.7.2.4]

sat; sulfate adenylyltransferase [EC:2.7.7.4]

dapA; 4-hydroxy-tetrahydrodipicolinate synthase [EC:4.3.3.7]

263. JAK-STAT signaling pathway

SOS; son of sevenless

STAM; signal transducing adaptor molecule

PIAS1; E3 SUMO-protein ligase PIAS1 [EC:2.3.2.-]

MTOR; serine/threonine-protein kinase mTOR [EC:2.7.11.1]

264. NF-kappa B signaling pathway

CSNK2A; casein kinase II subunit alpha [EC:2.7.11.1]

CSNK2B; casein kinase II subunit beta

ATM; serine-protein kinase ATM [EC:2.7.11.1]

UBE2I; ubiquitin-conjugating enzyme E2 I

265. Circadian rhythm

SKP1; S-phase kinase-associated protein 1

CUL1; cullin 1

PRKAB; 5'-AMP-activated protein kinase, regulatory beta subunit

PRKAG; 5'-AMP-activated protein kinase, regulatory gamma subunit

266. Aldosterone synthesis and secretion

CALM; calmodulin

PRKCA; classical protein kinase C alpha type [EC:2.7.11.13]

PKA; protein kinase A [EC:2.7.11.11]

CAMK1; calcium/calmodulin-dependent protein kinase I [EC:2.7.11.17]

267. Type II diabetes mellitus

HK; hexokinase [EC:2.7.1.1]

PK; pyruvate kinase [EC:2.7.1.40]

ERK; mitogen-activated protein kinase 1/3 [EC:2.7.11.24]

MTOR; serine/threonine-protein kinase mTOR [EC:2.7.11.1]

268. Streptomycin biosynthesis

HK; hexokinase [EC:2.7.1.1]

rfbB; dTDP-glucose 4,6-dehydratase [EC:4.2.1.46]

pgm; phosphoglucomutase [EC:5.4.2.2]

INO1; myo-inositol-1-phosphate synthase [EC:5.5.1.4]

269. Drug metabolism - cytochrome P450

frmA; S-(hydroxymethyl)glutathione dehydrogenase / alcohol dehydrogenase [EC:1.1.1.284 1.1.1.1]

E1.2.1.5; aldehyde dehydrogenase (NAD(P)+) [EC:1.2.1.5]

GST; glutathione S-transferase [EC:2.5.1.18]

adhP; alcohol dehydrogenase, propanol-preferring [EC:1.1.1.1]

270. Other types of O-glycan biosynthesis

POMT; dolichyl-phosphate-mannose-protein mannosyltransferase [EC:2.4.1.109]

MNN1; alpha 1,3-mannosyltransferase [EC:2.4.1.-]

KTR1_3; alpha 1,2-mannosyltransferase [EC:2.4.1.-]

MNN25; alpha 1,2-mannosyltransferase [EC:2.4.1.-]

271. Steroid hormone biosynthesis

COMT; catechol O-methyltransferase [EC:2.1.1.6]

E3.1.6.1; arylsulfatase [EC:3.1.6.1]

HSD17B12; 17beta-estradiol 17-dehydrogenase / very-long-chain 3-oxoacyl-CoA reductase [EC:1.1.1.62 1.1.1.330]

SRD5A3; 3-oxo-5-alpha-steroid 4-dehydrogenase 3 / polyprenol reductase [EC:1.3.1.22 1.3.1.94]

272. Isoquinoline alkaloid biosynthesis

AOC3; primary-amine oxidase [EC:1.4.3.21]

ARO8; aromatic amino acid aminotransferase I / 2-aminoadipate transaminase [EC:2.6.1.57 2.6.1.39 2.6.1.27 2.6.1.5]

GOT1; aspartate aminotransferase, cytoplasmic [EC:2.6.1.1]

GOT2; aspartate aminotransferase, mitochondrial [EC:2.6.1.1]

273. Prion diseases

PKA; protein kinase A [EC:2.7.11.11]

ERK; mitogen-activated protein kinase 1/3 [EC:2.7.11.24]

SOD1; superoxide dismutase, Cu-Zn family [EC:1.15.1.1]

STIP1; stress-induced-phosphoprotein 1

274. Acute myeloid leukemia

SOS; son of sevenless

ERK; mitogen-activated protein kinase 1/3 [EC:2.7.11.24]

MTOR; serine/threonine-protein kinase mTOR [EC:2.7.11.1]

KRAS; GTPase KRas

275. Cholesterol metabolism

SOAT; sterol O-acyltransferase [EC:2.3.1.26]

LIPA; lysosomal acid lipase/cholesteryl ester hydrolase [EC:3.1.1.13]

NPC1; Niemann-Pick C1 protein

VDAC2; voltage-dependent anion channel protein 2

276. Biotin metabolism

fabG; 3-oxoacyl-[acyl-carrier protein] reductase [EC:1.1.1.100]

bioB; biotin synthase [EC:2.8.1.6]

HLCS; biotin---protein ligase [EC:6.3.4.9 6.3.4.10 6.3.4.11 6.3.4.15]

fabF; 3-oxoacyl-[acyl-carrier-protein] synthase II [EC:2.3.1.179]

277. Gastric acid secretion

CALM; calmodulin

PRKCA; classical protein kinase C alpha type [EC:2.7.11.13]

PKA; protein kinase A [EC:2.7.11.11]

GNAI; guanine nucleotide-binding protein G(i) subunit alpha

278. Degradation of aromatic compounds

AKR1A1; alcohol dehydrogenase (NADP+) [EC:1.1.1.2]

frmA; S-(hydroxymethyl)glutathione dehydrogenase / alcohol dehydrogenase [EC:1.1.1.284 1.1.1.1]

E1.14.13.1; salicylate hydroxylase [EC:1.14.13.1]

adhP; alcohol dehydrogenase, propanol-preferring [EC:1.1.1.1]

279. Th1 and Th2 cell differentiation

PPP3C; serine/threonine-protein phosphatase 2B catalytic subunit [EC:3.1.3.16]

ERK; mitogen-activated protein kinase 1/3 [EC:2.7.11.24]

P38; p38 MAP kinase [EC:2.7.11.24]

PPP3R; serine/threonine-protein phosphatase 2B regulatory subunit

280. Aldosterone-regulated sodium reabsorption

PRKCA; classical protein kinase C alpha type [EC:2.7.11.13]

ERK; mitogen-activated protein kinase 1/3 [EC:2.7.11.24]

PDPK1; 3-phosphoinositide dependent protein kinase-1 [EC:2.7.11.1]

KRAS; GTPase KRas

281. Naphthalene degradation

frmA; S-(hydroxymethyl)glutathione dehydrogenase / alcohol dehydrogenase [EC:1.1.1.284 1.1.1.1]

E1.14.13.1; salicylate hydroxylase [EC:1.14.13.1]

adhP; alcohol dehydrogenase, propanol-preferring [EC:1.1.1.1]

282. Chloroalkane and chloroalkene degradation

frmA; S-(hydroxymethyl)glutathione dehydrogenase / alcohol dehydrogenase [EC:1.1.1.284 1.1.1.1]

ALDH; aldehyde dehydrogenase (NAD+) [EC:1.2.1.3]

adhP; alcohol dehydrogenase, propanol-preferring [EC:1.1.1.1]

283. Salivary secretion

CALM; calmodulin

PRKCA; classical protein kinase C alpha type [EC:2.7.11.13]

PKA; protein kinase A [EC:2.7.11.11]

284. Bile secretion

HMGCR; hydroxymethylglutaryl-CoA reductase (NADPH) [EC:1.1.1.34]

PKA; protein kinase A [EC:2.7.11.11]

ABCB1; ATP-binding cassette, subfamily B (MDR/TAP), member 1 [EC:7.6.2.2]

285. Styrene degradation

E3.5.1.4; amidase [EC:3.5.1.4]

E3.5.5.1; nitrilase [EC:3.5.5.1]

PHAA; phenylacetate 2-hydroxylase [EC:1.14.14.54]

286. Fat digestion and absorption

ACAT; acetyl-CoA C-acetyltransferase [EC:2.3.1.9]

AGPAT1_2; lysophosphatidate acyltransferase [EC:2.3.1.51]

GOT2; aspartate aminotransferase, mitochondrial [EC:2.6.1.1]

287. alpha-Linolenic acid metabolism

E1.3.3.6; acyl-CoA oxidase [EC:1.3.3.6]

ACAA1; acetyl-CoA acyltransferase 1 [EC:2.3.1.16]

TGL4; TAG lipase / steryl ester hydrolase / phospholipase A2 / LPA acyltransferase [EC:3.1.1.3 3.1.1.13 3.1.1.4 2.3.1.51]

288. Dorso-ventral axis formation

SOS; son of sevenless

ERK; mitogen-activated protein kinase 1/3 [EC:2.7.11.24]

KRAS; GTPase KRas

289. Thyroid cancer

ERK; mitogen-activated protein kinase 1/3 [EC:2.7.11.24]

KRAS; GTPase KRas

TPR; nucleoprotein TPR

290. Toll-like receptor signaling pathway

ERK; mitogen-activated protein kinase 1/3 [EC:2.7.11.24]

RAC1; Ras-related C3 botulinum toxin substrate 1

P38; p38 MAP kinase [EC:2.7.11.24]

291. Regulation of lipolysis in adipocytes

MGLL; acylglycerol lipase [EC:3.1.1.23]

PKA; protein kinase A [EC:2.7.11.11]

GNAI; guanine nucleotide-binding protein G(i) subunit alpha

292. Olfactory transduction

CALM; calmodulin

PKA; protein kinase A [EC:2.7.11.11]

GNB1; guanine nucleotide-binding protein G(I)/G(S)/G(T) subunit beta-1

293. GnRH secretion

PRKCA; classical protein kinase C alpha type [EC:2.7.11.13]

ERK; mitogen-activated protein kinase 1/3 [EC:2.7.11.24]

KRAS; GTPase KRas

294. Lipoic acid metabolism

lipA; lipoyl synthase [EC:2.8.1.8]

LIPT2; lipoyl(octanoyl) transferase 2 [EC:2.3.1.181]

LIP3; octanoyl-CoA:protein transferase [EC:2.3.1.-]

295. Phosphonate and phosphinate metabolism

PCYT2; ethanolamine-phosphate cytidylyltransferase [EC:2.7.7.14]

PCYT1; choline-phosphate cytidylyltransferase [EC:2.7.7.15]

EPT1; ethanolaminephosphotransferase [EC:2.7.8.1]

296. C5-Branched dibasic acid metabolism

leuB; 3-isopropylmalate dehydrogenase [EC:1.1.1.85]

E2.2.1.6L; acetolactate synthase I/II/III large subunit [EC:2.2.1.6]

E2.2.1.6S; acetolactate synthase I/III small subunit [EC:2.2.1.6]

297. TNF signaling pathway

ERK; mitogen-activated protein kinase 1/3 [EC:2.7.11.24]

P38; p38 MAP kinase [EC:2.7.11.24]

DNM1L; dynamin 1-like protein [EC:3.6.5.5]

298. Leishmaniasis

EEF1A; elongation factor 1-alpha

ERK; mitogen-activated protein kinase 1/3 [EC:2.7.11.24]

P38; p38 MAP kinase [EC:2.7.11.24]

299. Chemical carcinogenesis

frmA; S-(hydroxymethyl)glutathione dehydrogenase / alcohol dehydrogenase [EC:1.1.1.284 1.1.1.1]

E1.2.1.5; aldehyde dehydrogenase (NAD(P)+) [EC:1.2.1.5]

GST; glutathione S-transferase [EC:2.5.1.18]

300. Primary immunodeficiency

add; adenosine deaminase [EC:3.5.4.4]

UNG; uracil-DNA glycosylase [EC:3.2.2.27]

DCLRE1C; DNA cross-link repair 1C protein [EC:3.1.-.-]

301. Aminobenzoate degradation

E3.1.3.41; 4-nitrophenyl phosphatase [EC:3.1.3.41]

E3.5.1.4; amidase [EC:3.5.1.4]

E3.5.5.1; nitrilase [EC:3.5.5.1]

302. Phototransduction - fly

CALM; calmodulin

PRKCA; classical protein kinase C alpha type [EC:2.7.11.13]

303. Insulin secretion

PRKCA; classical protein kinase C alpha type [EC:2.7.11.13]

PKA; protein kinase A [EC:2.7.11.11]

304. Betalain biosynthesis

COMT; catechol O-methyltransferase [EC:2.1.1.6]

DOPA; 4,5-DOPA dioxygenase extradiol [EC:1.13.11.-]

305. Circadian rhythm - plant

CSNK2A; casein kinase II subunit alpha [EC:2.7.11.1]

CSNK2B; casein kinase II subunit beta

306. Prodigiosin biosynthesis

fabG; 3-oxoacyl-[acyl-carrier protein] reductase [EC:1.1.1.100]

fabD; [acyl-carrier-protein] S-malonyltransferase [EC:2.3.1.39]

307. Bacterial secretion system

SRP54; signal recognition particle subunit SRP54 [EC:3.6.5.4]

yidC; YidC/Oxa1 family membrane protein insertase

308. Atrazine degradation

E6.3.4.6; urea carboxylase [EC:6.3.4.6]

DDI2_3; cyanamide hydratase [EC:4.2.1.69]

309. Synthesis and degradation of ketone bodies

ACAT; acetyl-CoA C-acetyltransferase [EC:2.3.1.9]

E2.3.3.10; hydroxymethylglutaryl-CoA synthase [EC:2.3.3.10]

310. Glycosphingolipid biosynthesis - globo and isoglobo series

E3.2.1.22B; alpha-galactosidase [EC:3.2.1.22]

HEXA_B; hexosaminidase [EC:3.2.1.52]

311. African trypanosomiasis

IDO; indoleamine 2,3-dioxygenase [EC:1.13.11.52]

PRKCA; classical protein kinase C alpha type [EC:2.7.11.13]

312. Phototransduction

CALM; calmodulin

GNB1; guanine nucleotide-binding protein G(I)/G(S)/G(T) subunit beta-1

313. Mineral absorption

SLC31A1; solute carrier family 31 (copper transporter), member 1

SLC30A1; solute carrier family 30 (zinc transporter), member 1

314. Hypertrophic cardiomyopathy (HCM)

PRKAB; 5'-AMP-activated protein kinase, regulatory beta subunit

PRKAG; 5'-AMP-activated protein kinase, regulatory gamma subunit

315. Glycosaminoglycan degradation

nagZ; beta-N-acetylhexosaminidase [EC:3.2.1.52]

HEXA_B; hexosaminidase [EC:3.2.1.52]

316. Circadian rhythm - fly

GSK3B; glycogen synthase kinase 3 beta [EC:2.7.11.26]

LSM12; protein LSM12

317. Novobiocin biosynthesis

hisC; histidinol-phosphate aminotransferase [EC:2.6.1.9]

ARO8; aromatic amino acid aminotransferase I / 2-aminoadipate transaminase [EC:2.6.1.57 2.6.1.39 2.6.1.27 2.6.1.5]

318. Apoptosis - multiple species

BECN; beclin

CYC; cytochrome c

319. Biofilm formation - Pseudomonas aeruginosa

trpE; anthranilate synthase component I [EC:4.1.3.27]

E4.6.1.1; adenylate cyclase [EC:4.6.1.1]

320. Arachidonic acid metabolism

LTA4H; leutriene-A4 hydrolase [EC:3.3.2.6]

TGL4; TAG lipase / steryl ester hydrolase / phospholipase A2 / LPA acyltransferase [EC:3.1.1.3 3.1.1.13 3.1.1.4 2.3.1.51]

321. Retinol metabolism

frmA; S-(hydroxymethyl)glutathione dehydrogenase / alcohol dehydrogenase [EC:1.1.1.284 1.1.1.1]

adhP; alcohol dehydrogenase, propanol-preferring [EC:1.1.1.1]

322. Sesquiterpenoid and triterpenoid biosynthesis

SQLE; squalene monooxygenase [EC:1.14.14.17]

FDFT1; farnesyl-diphosphate farnesyltransferase [EC:2.5.1.21]

323. Penicillin and cephalosporin biosynthesis

DAO; D-amino-acid oxidase [EC:1.4.3.3]

IAL; isopenicillin-N N-acyltransferase like protein

324. Carbapenem biosynthesis

proA; glutamate-5-semialdehyde dehydrogenase [EC:1.2.1.41]

proB; glutamate 5-kinase [EC:2.7.2.11]

325. Glycosphingolipid biosynthesis - ganglio series

SLC33A1; MFS transporter, PAT family, solute carrier family 33 (acetyl-CoA transportor), member 1 [EC:2.3.1.-]

HEXA_B; hexosaminidase [EC:3.2.1.52]

326. Biosynthesis of vancomycin group antibiotics

rfbB; dTDP-glucose 4,6-dehydratase [EC:4.2.1.46]

327. Dilated cardiomyopathy (DCM)

PKA; protein kinase A [EC:2.7.11.11]

328. Vitamin digestion and absorption

ABCC1; ATP-binding cassette, subfamily C (CFTR/MRP), member 1 [EC:7.6.2.3]

329. Biosynthesis of ansamycins

E2.2.1.1; transketolase [EC:2.2.1.1]

330. Mannose type O-glycan biosynthesis

POMT; dolichyl-phosphate-mannose-protein mannosyltransferase [EC:2.4.1.109]

331. Acarbose and validamycin biosynthesis

rfbB; dTDP-glucose 4,6-dehydratase [EC:4.2.1.46]

332. Plant hormone signal transduction

PR1; pathogenesis-related protein 1

333. Phenazine biosynthesis

trpE; anthranilate synthase component I [EC:4.1.3.27]

334. Nicotine addiction

SLC32A; solute carrier family 32 (vesicular inhibitory amino acid transporter)

335. Caprolactam degradation

AKR1A1; alcohol dehydrogenase (NADP+) [EC:1.1.1.2]

336. Cortisol synthesis and secretion

PKA; protein kinase A [EC:2.7.11.11]

337. Proximal tubule bicarbonate reclamation

SLC25A10; solute carrier family 25 (mitochondrial dicarboxylate transporter), member 10

338. Renin-angiotensin system

CTSA; cathepsin A (carboxypeptidase C) [EC:3.4.16.5]

339. Polyketide sugar unit biosynthesis

rfbB; dTDP-glucose 4,6-dehydratase [EC:4.2.1.46]

340. D-Arginine and D-ornithine metabolism

DAO; D-amino-acid oxidase [EC:1.4.3.3]

341. Polycyclic aromatic hydrocarbon degradation

E1.14.13.1; salicylate hydroxylase [EC:1.14.13.1]

342. Chlorocyclohexane and chlorobenzene degradation

E3.1.1.45; carboxymethylenebutenolidase [EC:3.1.1.45]

343. Ascorbate and aldarate metabolism

ALDH; aldehyde dehydrogenase (NAD+) [EC:1.2.1.3]

344. beta-Lactam resistance

nagZ; beta-N-acetylhexosaminidase [EC:3.2.1.52]

345. Limonene and pinene degradation

ALDH; aldehyde dehydrogenase (NAD+) [EC:1.2.1.3]

346. Caffeine metabolism

uaZ; urate oxidase [EC:1.7.3.3]

347. Neomycin, kanamycin and gentamicin biosynthesis

HK; hexokinase [EC:2.7.1.1]

348. Taste transduction

PKA; protein kinase A [EC:2.7.11.11]

349. Carbohydrate digestion and absorption

HK; hexokinase [EC:2.7.1.1]

350. Ovarian steroidogenesis

PKA; protein kinase A [EC:2.7.11.11]

351. Basal cell carcinoma

GSK3B; glycogen synthase kinase 3 beta [EC:2.7.11.26]

352. Benzoate degradation

ACAT; acetyl-CoA C-acetyltransferase [EC:2.3.1.9]

353. Protein digestion and absorption

SLC36A; solute carrier family 36 (proton-coupled amino acid transporter)

354. Toluene degradation

E3.1.1.45; carboxymethylenebutenolidase [EC:3.1.1.45]

355. Insect hormone biosynthesis

ALDH; aldehyde dehydrogenase (NAD+) [EC:1.2.1.3]

356. Biofilm formation - Escherichia coli

PYG; glycogen phosphorylase [EC:2.4.1.1]

357. Zeatin biosynthesis

miaA; tRNA dimethylallyltransferase [EC:2.5.1.75]

358. Aflatoxin biosynthesis

ACACA; acetyl-CoA carboxylase / biotin carboxylase 1 [EC:6.4.1.2 6.3.4.14 2.1.3.15]

359. Glucosinolate biosynthesis

E2.6.1.42; branched-chain amino acid aminotransferase [EC:2.6.1.42]

360. Linoleic acid metabolism

TGL4; TAG lipase / steryl ester hydrolase / phospholipase A2 / LPA acyltransferase [EC:3.1.1.3 3.1.1.13 3.1.1.4 2.3.1.51]

361. Dioxin degradation

E1.14.13.1; salicylate hydroxylase [EC:1.14.13.1]

362. Phenylpropanoid biosynthesis

bglX; beta-glucosidase [EC:3.2.1.21]

363. Cell cycle - Caulobacter

lon; ATP-dependent Lon protease [EC:3.4.21.53]

364. Fluorobenzoate degradation

E3.1.1.45; carboxymethylenebutenolidase [EC:3.1.1.45]
